# Total Synthesis of Jerangolid B via sp^3^–sp^2^ Stille Coupling

**DOI:** 10.1021/acs.orglett.5c02569

**Published:** 2025-07-05

**Authors:** Janick Schug, Bernd Morgenstern, Johann Jauch

**Affiliations:** Organic Chemistry II, Saarland University, Campus C4.2, 66123 Saarbrücken, Germany

## Abstract

First isolated from So ce 307, jerangolids are a class of natural products with high antifungal activity and minimal toxicity toward mammals. Comprised of a skipped diene substructure with a chiral center in between a δ-lactone and a pyran substituent, they present an intriguing synthetic challenge. We herein report the first synthesis of jerangolid B through a modular approach that incorporates sp^3^–sp^2^ Stille coupling as the key step to generate the skipped diene structure. By comparing our synthetic jerangolid B to the data published in the literature, we could show that the configuration at C14 is R.

## Graphical abstract

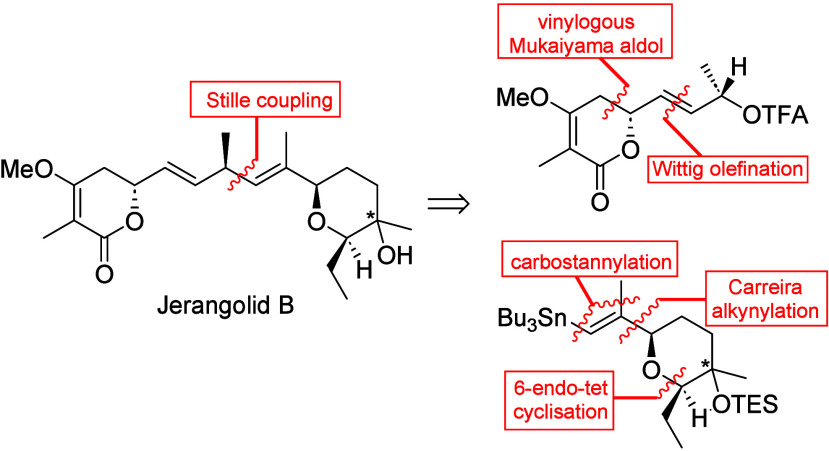

Jerangolids are a class of polyketides that were first isolated by Höfle et al. in 1995 from the myxobacterium So ce 307.^1^ They consist of a skipped 1,4-pentadiene core motif, which is connected to an δ-lactone and a pyran substituent (Figure 1).

**Figure 1:**
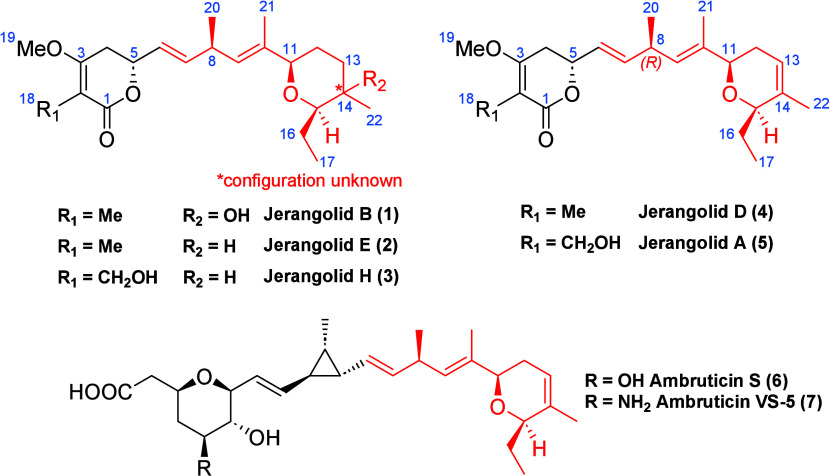
Structures of known jerangolids and selected ambruticins.

Structurally, they are closely related to ambruticins, which were isolated from var. and So ce 10 by Strandtmann et al.^2^ Their highly potent antifungal activity paired with minimal toxicity toward mammals make the jerangolids an interesting target for total synthesis.^1a,2e^ Both classes target the high osmolarity glycerol (HOG) signaling pathway in histidine kinase 1 (Hik1) expressing cells, leading to an intracellular accumulation of free fatty acids and glycerol resulting in cell death.^3^ Because both have the skipped diene and the pyran motif (C6–C17) in common, it has been proposed that this structure constitutes the pharmacophore responsible for biological activity.^4^ Their unique mode of action and challenging structural motif make the jerangolids an interesting synthetic target.

To date, several syntheses of natural occurring jerangolids are known.^5^ Jerangolid D (**4**, 22 steps, 6.1% yield) was the first to be synthesized by Markó et al.,^5a^ followed by jerangolid A (**5**, 23 steps, 1.9% yield) by Hanessian et al.^5b^ and recently the synthesis of jerangolid E (**2**, 23 steps, 4.0% yield) by Hahn et al. in 2018.^5c^ Their key strategy relies on using olefination reactions to generate the skipped diene structure from three respective building blocks. Several syntheses of truncated non-natural analogues of jerangolids and ambruticins have been described as well, though jerangolids B (**1**) and H (**3**) remain elusive.^4a,6^ Although vast efforts have been made, there still exists no general method that allows all natural jerangolids and their potential derivatives to be accessed.

In our quest to find a common synthetic strategy, we turned our attention to the synthesis of jerangolid B (**1**) (Scheme 1). We envisioned building the skipped diene at C8 through sp^3^–sp^2^ Stille coupling of chiral allylic trifluoroacetate **6** with vinylstannane **7**. The idea behind this is that, in palladium-catalyzed allylic substitution reactions, hard nucleophiles, such as vinylstannane **7**, attack the generated π-allylpalladium complex directly via an inner sphere mechanism, leading to overall inversion of configuration.^7^ The required stereocenter at C8 can be traced back to methyl l-(−)-lactate (**9**). Stannane **7** could be synthesized by Carreira alkynylation of aldehyde **10**, followed by 6-*endo*-tet cyclization and carbostannylation of the generated terminal alkyne. Using only two building blocks, which can be coupled late in the synthesis, should allow us to easily generate derivatives through modification of either building block.

**Scheme 1:**
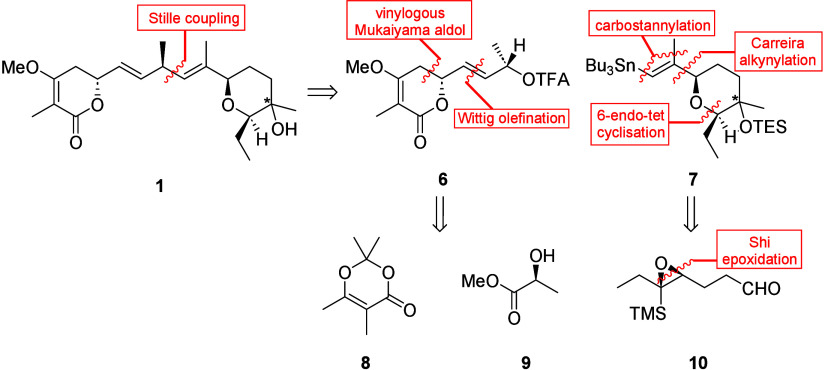
Retrosynthetic Analysis of Jerangolid B (**1**)

Synthesis of the lactone building block (Scheme 2) started with the preparation of aldehyde **14** according to the procedure described by Könning et al.^8^ Methyl l-(−)-lactate (**9**) was protected as TBS ether **11** followed by DIBALH reduction to aldehyde, and subsequent Wittig olefination yielded the unsaturated ester **12** quantitatively as a 1.5:1 *Z*/*E* mixture. After a second DIBALH reduction, allylic alcohol **13** was then subjected to the one-pot oxidation/isomerization protocol, which afforded aldehyde **14** in quantitative yields.^8^

Next we subjected aldehyde **14** to a vinylogous Mukaiyama aldol reaction (VMAR)^9^ with freshly prepared silyl ketene acetal **15**.^10^ We were initially delighted to find that using (*S*)-BINOL as the chiral ligand (Table 1, entry 1) produced allylic alcohol **16** in good yields with a 5:95 dr.^11^ However, through Mosher ester analysis, we discovered that the product was the undesired diastereomer. Simply changing to (*R*)-BINOL provided the desired isomer, albeit in lower yields and dr (Table 1, entry 2). To explain the lower selectivity and yield, we assume that (*S*)-**14** and (*R*)-BINOL form a mismatched pair, whereas (*S*)-**14** and (*S*)-BINOL form a matched pair. To increase the selectivity in the mismatched pair, we lowered the reaction temperature to −60 °C (Table 1, entry 3) and obtained 79% compound **16** with an acceptable dr of 90:10. Further decreases in the temperature proved to be detrimental (Table 1, entry 4). Additionally, other conditions described by Carreira et al. were tested (Table 1, entry 5) but were unsuccessful.^12^

**Table 1:** Optimization of the VMAR for the Synthesis of Compound **16**

	conditions^a^	yield (%)	dr (*anti*/*syn*)
1^a^	0.5 equiv of (*S*)-BINOL, 20 °C, and 4 h	80	5:95
2	0.5 equiv of (*R*)-BINOL, –20 °C, and 4 h	60	83:17
3	0.5 equiv of (*R*)-BINOL, –60 °C, and 3 days	79	90:10
4	0.5 equiv of (*R*)-BINOL, –78 °C, and 3 days	55	87:13
5	Cu(OTf)_2_, *S*-(Tol-BINAP), PH_3_SiF_2_(Bu_4_N), –78 °C, and 24 h	59	51:49

a All reactions were conducted in THF­(abs) using 1.5 equiv of compound **15**.

b A total of 0.5 equiv of Ti­(OiPr)_4_ was used in entries 1–4.

Lactone **17** was obtained by cyclization under mildly basic conditions with subsequent O-methylation using Me_2_SO_4_ in a good yield.^10b,13^ The use of TMSCHN_2_ also worked as a methylating reagent, albeit in only a 45% yield.^14^ It is noteworthy that the lactone subunit, in general, is quite vulnerable. Catalytic amounts of Lewis acid in the presence of water led to enol ether cleavage after extended periods of time, while basic conditions caused deprotonation at C4, resulting in the formation of trienic acid.^15^ Considering this, it is generally advantageous to introduce this subunit at a later stage in the synthesis. Careful deprotection (*vide supra*) of compound **17** using catalytical amounts of Bi­(OTf)_3_ in water/acetonitrile,^16^ followed by trifluoroacylation, finally led to the desired lactone **6** in 84% yield over two steps (Scheme 2).

**Scheme 2:**
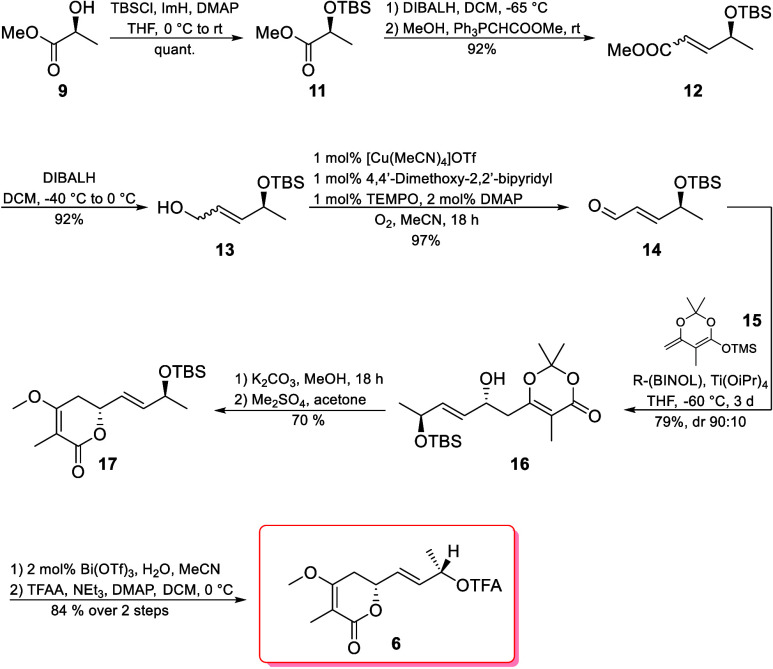
Construction of the Lactone **6** Building Block

Next, we turned our attention to the synthesis of vinylstannane **7** (Scheme 3). Our synthesis started with alkene **18**, which was prepared in two steps from commercially available 5-trimethylsilyl-pent-4-yn-1-ol as described previously.^6a^ Alkene **18** was then cleanly transformed into epoxide **20** through Shi epoxidation, using catalyst **19** derived from l-sorbose, with 75% yield and 92% ee.^17^ Subsequent DMP oxidation delivered aldehyde **10** in 83% yield. Because **10** decomposes rather quickly even at −18 °C, it was used immediately after purification in the following step.

**Scheme 3:**
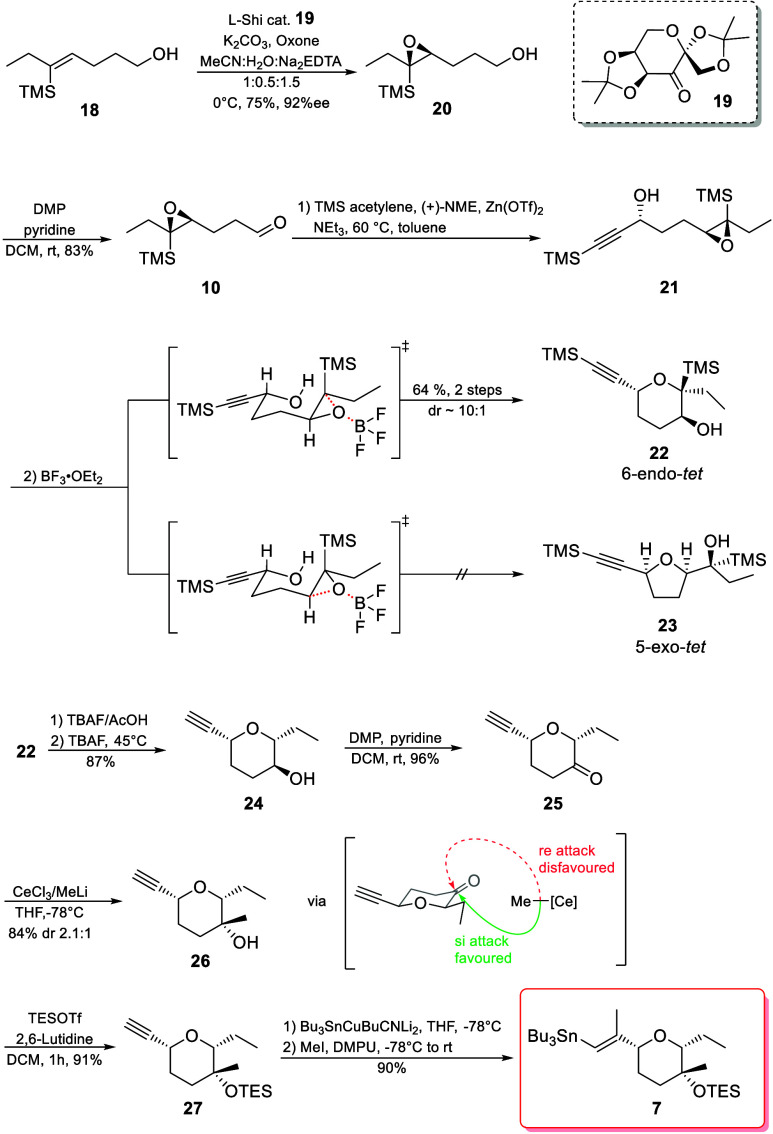
Synthesis of Pyran Fragment **7**

We then generated the stereocenter at C11 via Carreira alkynylation of compound **10**.^18^ Our initial runs using standard Carreira conditions led to complete decomposition of the starting material. Compound **10** is an aliphatic aldehyde, which can easily react with itself via aldol addition under these conditions.^19^ It was therefore crucial to add aldehyde **10** very slowly via a syringe pump. Additionally, TMS acetylene needed to be used in great excess due to its high volatility. Our best results were achieved by using 1.1 equiv of Zn­(OTf)_2_, 1.2 equiv of NEt_3_, and 1.2 equiv of (+)-NME at 60 °C and adding aldehyde **10** over 10 h, obtaining propagyl alcohol **21**, which was directly cyclized using BF_3_·OEt_2_ without purification. This led to 6-*endo*-tet cyclization, forming compound **22** as a 10:1 mixture of separable diastereomers. Usually, the spiro transition state leading to 5-*exo*-tet product **23** is favored over the fused transition state leading to the preferred 6-*endo*-tet product **22** in epoxide openings.^20^ The installation of a TMS group at the axial position was therefore pivotal for the success of this step, as it facilitates the 6-*endo*-tet cyclization by selectively weakening the adjacent C–O bond through a favorable σ_C–Si_–2p_O_ orbital interaction and stabilizing the developing positive charge in the transition state.^21^

To obtain the right configuration of the tertiary alcohol at C14 in jerangolid B (**1**), the TMS group at C15 first needed to be cleaved for the Si site to become accessible for methylation.^22^ Therefore, through protiodesilylation of the TMS groups with TBAF, we obtained compound **24** in an 87% yield. The acetylenic TMS group had to be cleaved first with a mixture of TBAF/AcOH, as just using an excess amount of TBAF sometimes leads to major formation of the Peterson olefination product.^21d,23^ Subsequent oxidation afforded ketone **25** in near-quantitative yields.

Both terminal acetylene and the α positions in ketone **25** are CH-acidic, which prompted us to investigate methods for clean stereoselective methylation without inducing deprotonation and subsequent side reactions.^24^ We first explored the ZnCl_2_-catalyzed addition of Grignard reagents to ketones developed by Hatano et al. (Table 2, entries 1–3).^25^ Using MeMgBr and a catalytic amount of ZnCl_2_, we attained high yields in overall methylation, but it unfortunately resulted primarily in the unwanted 14-(*S*) epimer in a 39:61 fashion (Table 2, entry 1). We rationalized that increasing the steric demand in the *in situ* generated R_3_ZnMgBr ate complex might favor attack from the Si site. However, using catalytic amounts of TMSCH_2_MgCl to introduce the TMSCH_2_ group as a non-transferable dummy ligand to increase steric demand of the ate complex neither increased the yield nor improved the diastereomeric ratio (Table 2, entry 2). Using stochiometric amounts of (TMSCH_2_)_2_ZnMeLi had no noticeable effect either (Table 2, entry 3).

**Table 2:** Optimization of the C14 Methylation of Compound **25** ^a^

	Me[M]	[M]X_*n*_	yield (%)	dr (*R*/*S*)
1	1.1 equiv of MeMgBr	0.1 equiv of ZnCl_2_	70	39:61
2	1.1 equiv of MeMgBr and 0.2 equiv of TMSCH_2_MgCl	0.1 equiv of ZnCl_2_ and 1.1 equiv of LiCl	74	45:55
3	1.5 equiv of MeLi and 3.0 equiv of TMSCH_2_MgCl	1.5 equiv of ZnCl_2_ and 1.1 equiv of LiCl	72	37:63
4	1.0 equiv of MeMgBr	1.0 equiv of CeCl_3_	76	40:60
5	1.0 equiv of MeLi	1.0 equiv of CeCl_3_	52	83:17
6	2.0 equiv of MeLi	1.0 equiv of CeCl_3_	69	61:39
7	3.0 equiv of MeLi	1.0 equiv of CeCl_3_	75	75:25
8	4.5 equiv of MeLi	1.5 equiv of CeCl_3_	84	68:32
9^b^	4.5 equiv of MeLi	1.5 equiv of CeCl_3_	30	86:14

a Reactions were conducted in THF on a 0.25 mmol scale at −78 °C.

b A total of 1.5 equiv of (*R*)-BINOL was used.

Changing to Imamoto's procedure using MeMgBr/CeCl_3_ unsurprisingly led to the same results (Table 2, entry 4).^26^ To our delight, using 1.0 equiv of MeLi and 1.0 equiv of CeCl_3_ resulted in the desired epimer **26** in an 83:17 ratio, albeit in a lower overall yield (Table 2, entry 5). This change in selectivity might in part be explained by the fact that MeLi reacts with CeCl_3_ to form an organocerium species, whereas with MeMgBr, alkylation is facilitated by the coordination of CeCl_3_ to the carbonyl group. While classical drying procedures state that CeCl_3_·7H_2_O can be mildly dehydrated by stepwise heating under high vacuum affording anhydrous CeCl_3_,^26a,27^ Evans et al. have shown that the material obtained is the solvated species [CeCl_3_(H_2_O)]_*n*_.^28^ Treating this compound in THF with MeLi leads to a complicated organometallic reagent in the form of [CeCl_*a*_Me_*b*_(OH)_*c*_O_*d*_Li_*e*_]_*f*_. The formation of this highly complex methylating reagent may also account for the encountered difficulty in reproducing diastereoselectivities (Table 2, entries 4–9). Overall, we found that a mixture of 4.5 equiv of MeLi and 1.5 equiv of CeCl_3_ maximized overall yields to 84% while still delivering an acceptable diastereomeric ratio of 68:32 (Table 2, entry 8). As both epimers could be separated by flash chromatography, no further optimizations were conducted.

TES protection of tertiary alcohol **26** using TESOTf afforded compound **27** in 91% yield. With stannylcupration of alkyne **27** with subsequent trapping of vinylcuprate with MeI,^29^ we obtained the desired vinylstannane **7** in near-quantitative yields after reversed-phase chromatography.

With compounds **6** and **7** synthesized, the stage was set for the sp^3^–sp^2^ Stille coupling of both building blocks. π-Allyl Stille couplings are staples in organic synthesis and have been extensively used in natural product syntheses to generate skipped 1,4-dienes. However, to our knowledge, no example of a coupling involving a chiral 2° or 3° allylic ester with a vinylstannane has been published in the field of total synthesis to date.^30^ This is surprising, given the close mechanistic relationship to the Tsuji–Trost reaction. It is well-established that, in reactions of unsymmetrical 1,3-disubstituted allylic substrates, such as compound **6**, the Tsuji–Trost reaction proceeds stereoselectively with retention of configuration at the stereocenter.^31^ This outcome results from a double inversion pathway involving oxidative addition and subsequent nucleophilic attack at the allyl position. Because an epimerization via η^3^–η^1^–η^3^ isomerization is not possible, the catalyst can only influence the regioselectivity, while the configuration is determined by the substrate. In our case, we therefore expect inversion of configuration at C8, as transmetalation of vinylstannane **7** to the Pd­(II) center precedes C–C bond formation (Scheme 4).

**Scheme 4:**
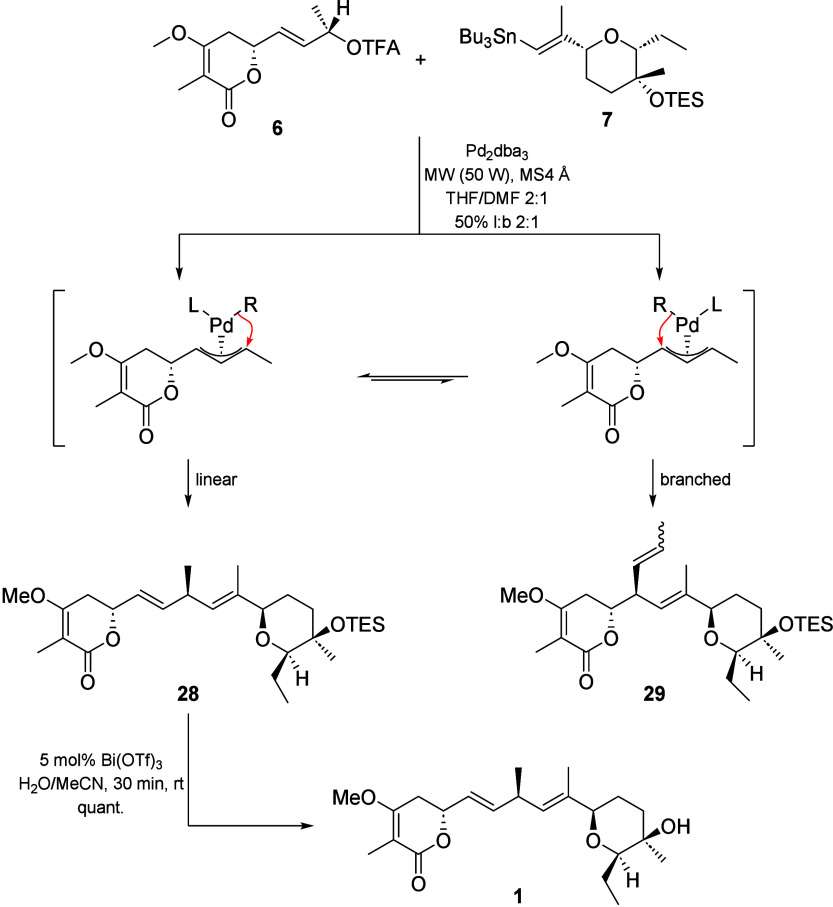
Final Steps in the Synthesis of Jerangolid B **1**

Through extensive experimentation, we found that the best yields for the coupling of compound **6** with compound **7** were achieved by utilizing Pd_2_dba_3_ as a catalyst, along with molecular sieves 4Å (to ensure anhydrous conditions) under microwave irradiation in a 2:1 mixture of THF and DMF. After 10 cycles of pulsed microwave irradiation (50 W, 1 min of irradiation, and 1 min of cooldown), we obtained 50% of the coupling product as a 2:1 linear/branched (l/b) mixture, which afforded 33% of pure compound **28** after chromatographic purification. ^13^C NMR indicated no epimerization at the central carbon center at C8. Removal of the TES group under mild conditions using 5 mol % Bi­(OTf)_3_ ultimately afforded jerangolid B (**1**) in quantitative yields. The use of TES was deliberate, as the TBS ether could not be cleaved under standard conditions without major decomposition of the lactone unit in compound **1**. By comparison of the NMR data of 14-*epi*-jerangolid B (**1**)^22^ to the data of compound **1** and natural jerangolid B, we could show that C14 has a *R* configuration.

In summary, we achieved the synthesis of jerangolid B (**1**) through a modular synthesis in 20 steps, with the longest linear sequence of 12 steps and 5.7% yield. Key steps included the stereoselective formation of the lactone core **17** through vinylogous Mukaiyama aldol reaction, the formation of the pyran fragment through Carreira alkynylation of aldehyde **10** with subsequent cyclization, and finally stereoselective π-allyl Stille coupling of building blocks **6** and **7** to generate the core jerangolid structure in a single step. Syntheses of other jerangolids and derivates are currently in progress and will be published in due course.

## Supplementary Material



## Data Availability

The data underlying this study are available in the published article and its Supporting Information.

## Notes

Experimental details, characterization data for all new compounds, ^1^H and ^13^C NMR spectra, and crystallographic data (PDF)

## Notes

This article is dedicated to Prof. Dr. Wolfang Steglich on the occasion of his 92nd birthday and to Prof. Dr. Volker Schurig on the occasion of his 85th birthday.

## References

[1] b Reichenbach, H. ; Höfle, G. ; Gerth, K. ; Washausen, P. Heterocyclic Compounds Obtainable from *Sorangium cellulosum* Bacteria, their Preparation Process, and Agents Containing These Compounds. German Patent 19607702 A1, Sept 4, 1997.

[2] Ringel S. M., Greenough R. C., Roemer S., Connor D., Gutt A. L., Blair B., Kanter G., Strandtmann M. V. (1977). Ambruticin
(W7783), a new antifungal antibiotic. J. Antibiot..

[3] Shubitz L. F., Galgiani J. N., Tian Z.-Q., Zhong Z., Timmermans P., Katz L. (2006). Efficacy of Ambruticin Analogs in
a Murine Model of Invasive Pulmonary Aspergillosis. Antimicrob. Agents Chemother..

[4] Hanessian S., Focken T., Mi X., Oza R., Chen B., Ritson D., Beaudegnies R. (2010). Total Synthesis
of (+)-Ambruticin S: Probing the Pharmacophoric Subunit. J. Org. Chem..

[5] Pospíšil J., Markó I. E. (2007). Total Synthesis
of Jerangolid D. J. Am. Chem. Soc..

[6] Lenhof J., Hutter M., Huch V., Jauch J. (2020). Towards the
Total Synthesis of Jerangolids—Synthesis of an Advanced Intermediate
for the Pharmacophore Substructure. Eur. J.
Org. Chem..

[7] Matsushita H., Negishi E. (1982). *anti*-Stereospecificity
in the Palladium-catalysed Reactions of Alkenyl- or Aryl-metal Derivatives
with Allylic Electrophilest. J. Chem. Soc.,
Chem. Commun..

[8] Könning D., Hiller W., Christmann M. (2012). One-Pot Oxidation/Isomerization of
Z-Allylic Alcohols with Oxygen as Stoichiometric Oxidant. Org. Lett..

[9] Casiraghi G., Zanardi F., Appendino G., Rassu G. (2000). The Vinylogous Aldol
Reaction: A Valuable, Yet Understated Carbon–Carbon Bond-Forming
Maneuver. Chem. Rev..

[10] Sato M., Ogasawara H., Oi K., Kato T. (1983). Synthesis of 1, 3-dioxin-4-one derivatives. Chem. Pharm. Bull..

[11] Dalby S. M., Goodwin-Tindall J., Paterson I. (2013). Total Synthesis of (−)-Rhizopodin. Angew. Chem., Int. Ed..

[12] Carreira E.
M., Singer R. A., Lee W. (1994). Catalytic,
Enantioselective Aldol Additions with Methyl and Ethyl Acetate *O*-Silyl Enolates: A Chiral Tridentate Chelate as a Ligand
for Titanium­(IV). J. Am. Chem. Soc..

[13] Smith T. E., Djang M., Velander A. J., Downey C. W., Carroll K. A., van Alphen S. (2004). Versatile Asymmetric Synthesis of the Kavalactones:
First Synthesis of (+)-Kavain. Org. Lett..

[14] Voight E. A., Seradj H., Roethle P. A., Burke S. D. (2004). Synthesis of the
Bryostatin 1 Northern Hemisphere (C1–C16) via Desymmetrization
by Ketalization/Ring-Closing Metathesis. Org.
Lett..

[15] Nakata T., Noriaki H., Katsumi I., Takeshi O. (1987). Determination of stereostructure
of naturally occurring
α, β-unsaturated δ-lactone derivatives through a
stereoselective synthesis. Tetrahedron Lett..

[16] Firouzabadi H., Mohammadpoor-Baltork I., Kolagar S. (2001). A rapid, selective, and efficient
method for deprotection
of silyl ethers catalyzed by bismuth­(III) salts. Syn. Commun..

[17] Tu Y., Wang Z.-X., Shi Y. (1996). An Efficient
Asymmetric Epoxidation Method for *trans*-Olefins Mediated
by a Fructose-Derived Ketone. J. Am. Chem. Soc..

[18] Frantz D. E., Fässler R., Carreira E. M. (1999). Catalytic *in Situ* Generation of Zn­(II)-Alkynilides
under Mild Conditions: A Novel CN Addition Process Utilizing Terminal
Acetylenes. J. Am. Chem. Soc..

[19] García-Fortanet J., Murga J., Carda M., Marco J. A. (2004). Stereoselective synthesis of hyptolide and 6-*epi*-hyptolide. Tetrahedron.

[20] Baldwin J.
E. (1976). Rules for
ring closure. J. Chem. Soc., Chem. Commun..

[21] Vilotijevic I., Jamison T. F. (2009). Epoxide-Opening Cascades in the Synthesis
of Polycyclic Polyether Natural Products. Angew.
Chem., Int. Ed..

[22] Through the same sequence of TES protection and carbostannylation of compound epi-**26**, we were able to synthesize the epimer of jerangolid B (**1**) with a *S* configuration at C14 (see the Supporting Information).

[23] Hudrlik P. F., Hudrlik A. M., Kulkarni A. K. (1982). Protodesilylation
reactions of simple β-hydroxysilanes (and α-hydroxysilanes).
Homo-Brook rearrangements. J. Am. Chem. Soc..

[24] Bartoli G., Marcantoni E., Marcolini M., Sambri L. (2010). Applications of CeCl_3_ as an Environmental
Friendly Promoter in Organic Chemistry. Chem.
Rev..

[25] Hatano M., Suzuki S., Ishihara K. (2006). Highly Efficient
Alkylation to Ketones and Aldimines with Grignard Reagents Catalyzed
by Zinc­(II) Chloride. J. Am. Chem. Soc..

[26] Imamoto T., Kusumoto T., Tawarayama Y., Sugiura Y., Mita T., Hatanaka Y., Yokoyama M. (1984). Carbon-carbon
bond-forming reactions using cerium metal or organocerium­(III) reagents. J. Org. Chem..

[27] Takeda N., Imamoto T. (1999). Use of Cerium­(III)
Chloride in the Reactions of Carbonyl Compounds with Organolithiums
or Grignard Reagents for the Suppression of Abnormal Reactions:1-Butyl-1,2,3,4-Tetrahydro-1-Naphthol. Org. Synth..

[28] Evans W. J., Feldman J. D., Ziller J. W. (1996). The Presence of Water in the Common
CeCl_3_/RLi Alkylation System. J. Am.
Chem. Soc..

[29] Lipshutz B. H., Ellsworth E. L., Dimock S. H., Reuter D. C. (1989). Transmetalation
reactions of higher
order cyanocuprates: Direct formation of trialkyltin cuprates from
tin hydrides which bypasses organolithium intermediates. Tetrahedron Lett..

[30] Heravi M. M., Mohammadkhani L. (2018). Recent applications of Stille reaction
in total synthesis
of natural products: An update. J. Organomet.
Chem..

[31] c Transition Metal Catalyzed Enantioselective Allylic Substitution in Organic Synthesis; Kazmaier, U. , Ed.; Springer: Berlin, Germany, 2012; Topics in Organometallic Chemistry, Vol. 38, 10.1007/978-3-642-22749-3. Only in rare cases is it possible to convert a racemic substrate to a single enantiomer via a dynamic kinetic resolution utilizing a Pd^0^-catalyzed allyl exchange. For selected examples, see

