# Supplementary material for: Total Synthesis of Jerangolid B via sp3–sp2 Stille Coupling
Source: Org Lett. 2025 Jul 5;27(28):7719–26. doi: 10.1021/acs.orglett.5c02569 (PMC12281574; doi:10.1021/acs.orglett.5c02569)
Supplement: Supplementary file 1 [file ol5c02569_si_001.pdf]

Supporting Information

Total synthesis of Jerangolid B via sp^3-sp^2 Stille coupling

Janick Schug, Bernd Morgenstern, Johann Jauch*

Organic Chemistry II, Saarland University, D-66123 Saarbrücken, Germany;
*E-Mail: j.jauch@mx.uni-saarland.de

Table of Contents

Table of Contents 2
Abbreviation list 3
General Information...4
Synthetic procedures..5
NMR spectra 41
X-Ray crystallographic data 75
Literature 81

Abbreviation list

[α]	optical rotation
Ac	acyl
Ar	aryl
BINOL	1,1'-Bi-2-naphthol
b.p.	boiling point
brsm	based on recovered starting material
dba	Dibenzylideneacetone
DCC	N,N'-Dicyclohexylcarbodimiide
DCM	Dichloromethane
DIBALH	Diisobutylaluminiumhydride
DIPA	Diisopropylamine
DMAP	4-Dimethylaminopyridine
DMF	N,N-Dimethylformamide
DMP	Dess-Martin periodinane
DMPU	N,N'-Dimethylpropylenurea
EDTA	Ethylenediaminetetraacetic acid
eq.	equivalents
Et	ethyl
HPLC	high performance liquid chromatography
HRMS	high resolution mass spectrometry
ImH	Imidazole
*i*Pr	isopropyl
LDA	Lithiumdiisopropylamide
Me	methyl
MS	molecular sieves
MTPA	α-Methoxy-α-trifluoromethylphenylacetic acid
MW	microwave
*n*Bu	*n*-butyl
*n*Hex	*n*Hexyl
NME	N-Methylephedrine
NMR	nuclear magnetic resonance
Ph	phenyl
PNB	paranitrobenzoyl
ppm	part per million
R_f	retention factor
r.t.	room temperature
sat.	saturated
TBAF	tetrabutylammoniumfluoride
TBS	*tert*-butyldimethylsilyl
TES	triethylsilyl
Tf	triflyl
TFA	trifluoroacyl
TFAA	trifluoroacetic anhydride
THF	Tetrahydrofurane
TMS	trimethylsilyl
TLC	thin layer chromatography

General Information

All reactions were run under an inert atmosphere in dried (heat gun) glassware unless stated otherwise. Anhydrous solvents used in reactions were purchased in HPLC grade quality and were additionally freshly distilled under N_2 atmosphere. **THF**, **Et_2O** and **toluene** were distilled from sodium/benzophenone while **DCM** and **DIPA** were distilled from CaH_2. Solvents used for flash chromatography and for the extraction of aqueous phases were distilled prior to their use. All other chemicals were used as purchased without further purification. All reactions were heated using an oil bath unless stated otherwise.

Silica gel for **flash chromatography** was purchased from Merck, Darmstadt, Germany (Silica 60, particle size 40–63 µm). **TLC** plates for reaction monitoring were purchased from Merck, Darmstadt, Germany ($Si60_{254}$ glass plates 50 × 100 mm). Visualization was achieved with a combination of UV detection (254 nm), iodine vapor and an anisaldehyde solution (85 mL MeOH, 10 mL HOAc, 5 mL concentrated H_2SO_4, 0.5 mL p-anisaldehyde) or $KMNO_4$ solution (12.5 g $KMNO_4$, 62.5 g, Na_2CO_3, 1.25 L H_2O)

Reversed-phase flash chromatography was performed using a Büchi Reveleris® Prep Chromatography System with Büchi FlashPure Select C18 30 µm spherical cartridges.

NMR spectra were recorded with a BRUKER AV II 400 NMR spectrometer (^1H = 400 MHz, ^{13}C = 100 MHz) or an AV 500 (^1H = 500 MHz, ^{13}C = 125 MHz). Chemical shifts (δ) are given in ppm. Deuterated chloroform $CDCl_3$ was used as the solvent with its chemical shifts at $\delta(^1H)$ = 7.26 ppm and $\delta(^{13}C)$ = 77.0 ppm being used as the internal standard. Multiplicities of the splitting patterns were abbreviated as follows: s (singlet), d (doublet), tr (triplet), q (quartet), quint (quintet), sext (sextet), sept (septet), m (multiplet), b (broad). All coupling constants are given in the unit Hertz (Hz). Structural assignments were made with additional information from gCOSY, gHSQC-DEPT, and gHMBC experiments. NMR spetra were analyzed using MestReNova Version 14.2.0-26256 from Mestrelab Research S.L.

Melting points were measured with a BÜCHI150 melting point device and are uncorrected.

Chiral **high-performance liquid chromatography (HPLC)** analysis was performed on a Merck Hitachi system consisting of a Merck Hitachi pump L-7100, Merck Hitachi autosampler L-7250, Merck Hitachi column oven L-7300, Merck Hitachi UV detector L-7455 and a Merck Hitachi experimental part 50 interface D-7000. The chiral chromatography columns used for normal phase chromatography were CHIRALCEL OD-H (250 x 4.6 mm) and CHIRALCEL OB-H (250 x 4.6 mm) from DAICEL CHEMI-CAL INDUSTRIES.

Specific optical rotations for enantiomerically pure or enriched substances were measured on a 241 MC polarimeter from PERKIN-ELMER with a sodium vapor lamp (λ = 589.3 nm) and are given in 10^{-1} $deg \cdot cm^2 \cdot g^{-1}$. The cuvettes used have a length of 1.0 dm. The concentrations c of the samples are given in g/100 mL. $CHCl_3$ used as solvent refers to $CHCl_3$ with amylene as stabilizer.

High-resolution mass spectra (HRMS) were measured at the Service Center for Mass Spectroscopy at Saarland University by Dr. Klaus Hollemeyer on a SolariX 7 Tesla MALDI/ESI/APPI/FTICR Imaging MS from Bruker Daltonics GmbH using a quadrupole mass analyzer.

Single crystal X-ray diffraction analysis was carried out by the service center for X-ray diffraction of Saarland University on a Bruker D8 Advance diffractometer.

Synthetic procedures

Methyl (S)-2-((*tert*-butyldimethylsilyl)oxy)propanoate 11[1]

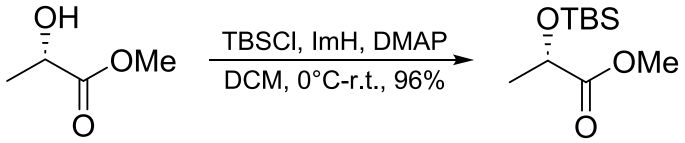

In a 250 mL round bottom flask under N_2 methyl (S)-2-hydroxypropanoate **9** (5.21 g, 50 mmol, 1.0 eq.) is dissolved in 50 mL anhydrous DCM and cooled to 0 °C. Imidazole (5.11 g, 75 mmol, 1.5 eq.) and DMAP (611 mg, 5.0 mmol, 0.1 eq.) are successively added to this solution followed by TBSCl (8.29 g, 55 mmol, 1.1 eq.). The cooling bath is removed and the resulting colorless suspension is stirred for 5 h. The suspension is quenched by adding sat. NH_4Cl solution. The phases are separated and the aqueous phase is extracted three times with 50 mL DCM. The combined organic phases are dried with $MgSO_4$. Filtration over a 2-3 cm pad of silica gel 60 and concentration in vacuo affords the product **11** as a colorless oil (10.47 g, 48 mmol, 96%) which used without further need for purification.

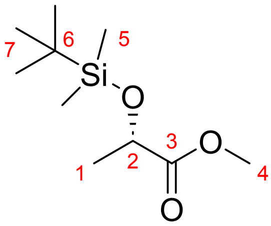

$C_{10}H_{22}O_3Si$
M = 218.37 g•mol^{-1}

TLC: R_f (pentane/Et_2O 1/1) = 0.82

^{1}H-NMR (400 MHz, $CDCl_3$, δ in ppm): 4.33 (q, J = 6.7 Hz, 1H, H2), 3.72 (s, 3H, H4), 1.40 (d, J = 6.7 Hz, 3H, H1), 0.90 (s, 9H, H7), 0.10 (s, 3H, H5), 0.07 (s, 3H, H5‘).

^{13}C-NMR (100 MHz, $CDCl_3$, δ in ppm): 174.7 (C3), 68.6 (C2), 52.0 (C4), 25.9 (C7), 21.5 (C1), 18.5 (C6), -4.8 (C5), -5.1 (C5‘).

The spectral data are in agreement with literature reports.[1]

Methyl (S)-4-((*tert*-butyldimethylsilyl)oxy)pent-2-enoate 12[1]

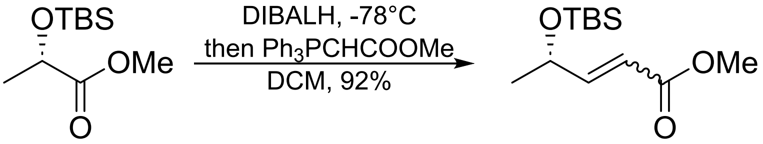

An oven dried 2L three necked round bottom flask with an addition funnel and a N_2 inlet is charged with the TBS protected (S)-2-hydroxypropanoate **11** (44.6 g, 200 mmol, 1.0 eq.). To this 400 mL of dry DCM are added and the solution is cooled down to below -70 °C (internal temperature) with a dry ice bath. Then DIBALH (0.9 M in DCM, 240 mL, 210 mmol, 1.05 eq.) is placed in the addition funnel and is subsequently added slowly to the reaction mixture over the course of 1 h while maintaining the internal temperature below -70 °C. The colorless mixture is stirred for an additional 30 min before 40 mL MeOH is added in two portions and stirred for another 30 min at below -70 °C. To the turbid mixture methyl 2-(triphenylphosphoranylidene)acetate (100.0 g, 300 mmol, 1.5 eq.) is added in one portion, the cooling bath is removed, and the resulting colorless suspension is allowed to reach room

temperature while stirring for 1 h. After complete conversion the suspension is treated with a saturated K-Na-tartrate solution. After stirring for 30 min the solution is transferred into a separating funnel. The phases are separated and the aqueous phase is extracted 3x with 200 mL DCM. The combined organic phases are dried with $MgSO_4$ and filtrated and concentrated in vacuo affording 131 g (268% crude yield) of the title compound as a colorless solid with substantial amounts of triphenylphosphine oxide as the main contaminant.

To remove the triphenylphosphine oxide impurity the crude product is dissolved in 400 mL EtOH and treated with 100 mL of a freshly prepared 2.0 M solution of $ZnCl_2$ in EtOH. After stirring for 20 min the triphenylphosphine oxide completely precipitates out of solution and is filtered off. The filtrate is concentrated in vacuo and again dissolved in 300 mL Et_2O. The organic phase is washed twice with H_2O and dried with $MgSO_4$. Filtration and evaporation of the solvent affords pure Methyl (S)-4-((*tert*-butyldimethylsilyl)oxy)pent-2-enoate **12** (44.9 g, 184 mmol, 92%, separatable 1.5:1 Z:E mixture) as a colorless oil.

Z isomer:

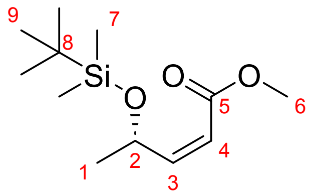

$C_{12}H_{24}O_3Si$
M = 244.41 g•mol^{-1}

TLC: R_f (pentane/Et_2O 15/1) = 0.42

^{1}H-NMR (400 MHz, $CDCl_3$, δ in ppm): 6.22 (dd, J = 11.7 Hz, 7.8 Hz, 1H, H3), 5.66 (dd, J = 11.7 Hz, 1.3 Hz, 1H, H4), 5.44 (dqd, J = 7.8 Hz, 6.3 Hz, 1.3 Hz, 1H, H2), 3.71 (s, 3H, H6), 1.25 (d, J = 6.3 Hz, 3H, H1), 0.88 (s, 9H, H9), 0.05 (s, 3H, H7), 0.03 (s, 3H, H7‘).

^{13}C-NMR (100 MHz, $CDCl_3$, δ in ppm): 166.4 (C5), 155.3 (C3), 116.5 (C3), 65.6 (C2), 51.4 (C6), 26.0 (C9), 23.7 (C1), 18.3 (C8), -4.6 (C7), -4.7 (C7‘).

E isomer:

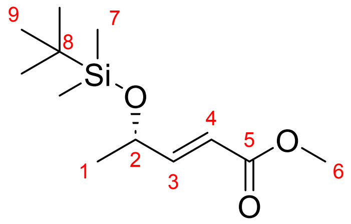

$C_{12}H_{24}O_3Si$
M = 244.41 g•mol^{-1}

TLC: R_f (pentane/Et_2O 15/1) = 0.25

^{1}H-NMR (400 MHz, $CDCl_3$, δ in ppm): 6.94 (dd, J = 15.5 Hz, 4.0 Hz, 1H, H3), 6.00 (dd, J = 15.5 Hz, 1.8 Hz, 1H, H4), 4.46 (qdd, J = 6.5 Hz, 4.0 Hz, 1.8 Hz, 1H, H2), 3.74 (s, 3H, H6), 1.26 (d, J = 6.5 Hz, 3H, H1), 0.91 (s, 9H, H9), 0.07 (s, 3H, H7), 0.06 (s, 3H, H7‘).

^{13}C-NMR (100 MHz, $CDCl_3$, δ in ppm): 167.5 (C5), 152.4 (C3), 118.7 (C4), 67.8 (C2), 51.7 (C6), 26.0 (C9), 23.7 (C1), 18.4 (C8), -4.7 (C7).

The spectral data are in agreement with literature reports.[1]

(S)-4-((*tert*-butyldimethylsilyl)oxy)pent-2-en-1-ol 13[1]

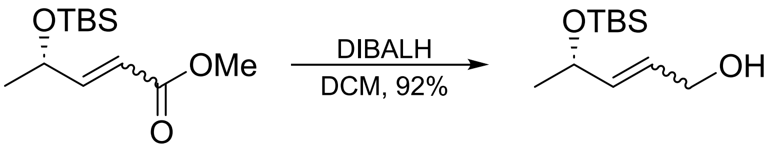

In an oven dried 1 L three necked round bottom flask with an addition funnel Methyl (S)-4-((*tert*-butyldimethylsilyl)oxy)pent-2-enoate **12** (44.9 g, 184 mmol, 1.0 eq.) is dissolved in 370 mL of anhydrous DCM and

cooled to -40 °C with a dry ice bath. DIBALH (380 mL, 1.0 M in hexane, 376 mmol, 2.05 eq.) is added into the addition funnel and slowly added dropwise to the reaction mixture over the course of 2 h. After complete addition the dry ice bath is exchanged for an ice bath and the reaction is stirred for an additional hour. The reaction is quenched slowly by dropwise addition of NH_4Cl (Caution! gas evolution with an induction period) with ice bath cooling resulting in a colorless slurry. Then 300 mL of a saturated K-Na-tartrate solution is added and the colorless slurry is stirred for 2 h. The resulting colorless biphasic mixture is transferred into a separatory funnel, the phases are separated and the aqueous phase is extracted 3x with 200 mL Et_2O. The combined organic phases are dried with $MgSO_4$. Filtration over a short pad of celite and evaporation of the solvent under vacuum affords (S)-4-((*tert*-butyldimethylsilyl)oxy)pent-2-en-1-ol **13** (36.7 g, 170 mmol, 92%) as a colorless oil, which was used without further need for purification.

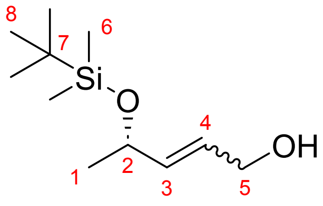

$C_{11}H_{24}O_2Si$
M = 216.40 g•mol^{-1}

TLC: R_f (pentane/Et_2O 3/1) = 0.24

Z isomer: ^{1}H-NMR (400 MHz, $CDCl_3$, δ in ppm): 5.60-5.48 (m, 2H, H3+H4), 5.48 (quin, J = 6.5 Hz, 1H, H2), 4.29-4.23 (m, 1H, H5), 4.18-4.10 (m, 1H, H5'), 1.84 (brs, 1H, OH), 1.22 (d, J = 6.3 Hz, 3H, H1), 0.89 (s, 9H, H8), 0.07 (s, 3H, H6), 0.06 (s, 3H, H6').

^{13}C-NMR (100 MHz, $CDCl_3$, δ in ppm): 137.1 (C3), 127.2 (C4), 66.7 (C2), 59.1 (C5), 26.0 (C8), 25.0 (C1), 18.4 (C7), -4.5 (C6), -4.6 (C6').

E isomer: ^{1}H-NMR (400 MHz, $CDCl_3$, δ in ppm): 5.82-5.68 (m, 2H, H3+H4), 4.36-4.30 (m, 1H, H2), 4.29-4.23 (m, 2H, H5), 1.30 (brs, 1H, OH), 1.21 (d, J = 6.4 Hz, 3H, H1), 0.90 (s, 9H, H8), 0.06 (s, 3H, H6), 0.05 (s, 3H, H6').

^{13}C-NMR (100 MHz, $CDCl_3$, δ in ppm): 136.6 (C3), 127.4 (C4), 68.6 (C2), 63.4 (C5), 26.0 (C8), 24.5 (C1), 18.4 (C7), -4.5 (C6), -4.6 (C6').

The spectral data are in agreement with literature reports.[1]

(S,E)-4-((*tert*-butyldimethylsilyl)oxy)pent-2-enal 14[1]

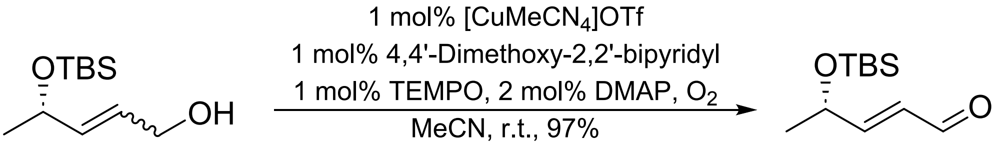

In a 500 mL Schlenk flask (S)-4-((*tert*-butyldimethylsilyl)oxy)pent-2-en-1-ol **13** (8.66 g, 40 mmol, 1.0 eq.) is dissolved in 160 mL dry MeCN. Then [Cu(MeCN)$_4$]OTf (151 mg, 0,4 mmol, 1 mol%), 4,4’-Dimethoxy-2,2’-bipyridyl (87 mg, 0,4 mmol, 1 mol%), TEMPO (63 mg, 0,4 mmol, 1 mol%) and DMAP (98 mg, 0,8 mmol, 2 mol%) are subsequently added, resulting in a dark brown solution. To the septum, an O_2 balloon fitted with a syringe reaching into the solution is connected. The mixture is then degassed and refilled with O_2 three times and stirred for 2 h while maintaining constant bubbling. The color changes from brownish red to green and finally to blue indicating complete oxidation. The reaction mixture is stirred overnight to ensure complete cis-trans isomerization. After complete reaction 200 mL H_2O are added and the mixture is transferred into a separatory funnel. The phases are separated and the aqueous phase is extracted 3x with 100 mL Et_2O. The combined organic phases are washed additionally with saturated NaCl solution and dried with $MgSO_4$. Filtration through a 2-3 cm pad of silica gel 60 and evaporation of the solvent under vacuum affords pure (S,E)-4-((*tert*-butyldimethylsilyl)oxy)pent-2-enal **14** (8.30 g, 38.7 mmol, 97% E:Z >50:1) as a colorless oil which was used without further need for purification.

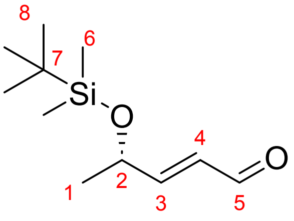

$C_{11}H_{22}O_2Si$
M = 214.38 g•mol^{-1}

TLC: R_f (pentane/Et_2O 2/1) = 0.55
$[\alpha]^D_{20}$ = +17.9 (c = 1.05, $CHCl_3$), Lit.: +17.9 (c = 1.07, $CHCl_3$)
^{1}H-NMR (400 MHz, $CDCl_3$, δ in ppm): 9.58 (d, J = 8.0 Hz, 1H, H5), 6.80 (dd, J = 15.5 Hz, 4.0 Hz, 1H, H3), 6.28 (ddd, J = 15.5 Hz, 8.0 Hz, 1.7 Hz, 1H, H4), 4.57 (qdd, J = 6.6 Hz, 4.0 Hz, 1.7 Hz, 1H, H2), 1.31 (d, J = 6.6 Hz, 3H, H1), 0.91 (s, 9H, H8), 0.08 (s, 3H, H6), 0.06 (s, 3H, H6’)
^{13}C-NMR (100 MHz, $CDCl_3$, δ in ppm): 194.0 (C5), 161.1 (C3), 130.0 (C4), 67.9 (C2), 25.9 (C9), 23.5 (C1), 18.3 (C8), -4.7 (C6), -4.8 (C6’)
The spectral data are in agreement with literature reports.[1]

tert-butyl 2-methyl-3-oxobutanoate S1[2]

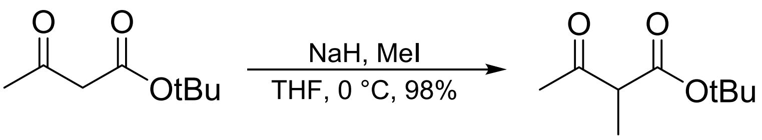

An oven dried 1L three necked round bottom flask with an addition funnel and an inlet adapter with a three-way stopcock is charged with NaH (60 w%, 16.79 g, 420 mmol, 1.05 eq). The flask is cooled to 0 °C with an ice bath and 40 mL of dry THF are added to wash the NaH. The supernatant solution is removed and the NaH slurry is

suspended in 400 mL of dry THF. To this stirred suspension *tert*-butyl 3-oxobutanoate (64.4 mL, 400 mmol, 1.0 eq.) is added dropwise at 0 °C resulting in a yellow solution. Then MeI (26.0 mL, 420 mmol, 1.05 eq.) is placed into the addition funnel and then added dropwise to the reaction over the course of an hour. After complete addition the reaction is allowed to reach room temperature resulting in a yellow suspension which is stirred for an additional hour. The reaction is quenched with a saturated NH_4Cl solution and transferred into a separatory funnel. The phases are separated and the aqueous phase is extracted 3x with 200 mL Et_2O. The combined organic phases are washed with a saturated NaCl solution and dried with $MgSO_4$. Filtration through a 2-3 cm pad of silica gel 60 and removal of the solvent in vacuo affords *tert*-butyl 2-methyl-3-oxobutanoate **S1** (67.5 g, 392 mmol, 98%) as a colorless oil, which was used without further need for purification.

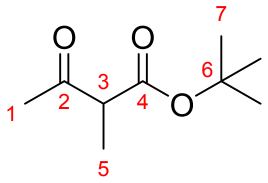

$C_9H_{16}O_3$
M = 172.22 g•mol^{-1}

TLC: R_f (pentane/Et_2O 3/1) = 0.52
^{1}H-NMR (400 MHz, $CDCl_3$, δ in ppm): 3.40 (q, J = 7.1 Hz, 1H, H3), 2.22 (s, 3H, H1), 1.46 (s, 9H, H7), 1.29 (d, J = 7.1 Hz, 3H, H5).
^{13}C-NMR (100 MHz, $CDCl_3$, δ in ppm): 204.2 (C2), 169.9 (C4), 81.9 (C6), 54.9 (C3), 28.5 (C1), 28.0 (C7), 12.8 (C5).
The spectral data are in agreement with literature reports.[2]

2,2,5,6-tetramethyl-4H-1,3-dioxin-4-one S2[3]

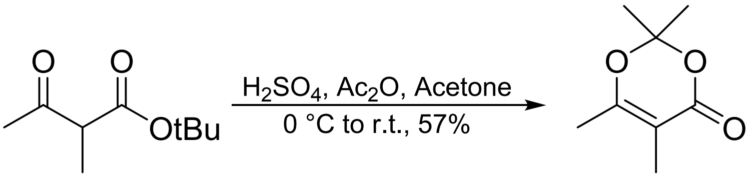

In a 250 mL round bottom flask *tert*-butyl 2-methyl-3-oxobutanoate **S1** (67.5 g, 390 mmol, 1.0 eq.) is dissolved in acetone (57 mL, 780 mmol, 2.0 eq.). The solution is cooled to 0 °C with an ice bath and Ac_2O (73 mL, 780 mmol, 2.0 eq.) is added in one portion. Then 22 mL of concentrated H_2SO_4 are added dropwise over 15 min and the reaction is stirred for 2 h at 0 °C. Afterwards the mixture is poured into a 1 L beaker filled with 200 mL of saturated $NaHCO_3$ solution and ice. The mixture is stirred until the gas evolution ceases and then transferred into a separatory funnel. The layers are separated and the aqueous layer is extracted 4x with 100 mL Et_2O. The combined organic phases are dried with $MgSO_4$, filtrated and the solvent is removed under reduced pressure. Purification of the residue by micro distillation ($T_{b.p.}$ = 84 °C, 11 Torr) affords 2,2,5,6-tetramethyl-4H-1,3-dioxin-4-one **S2** (35.0 g, 224 mmol, 57%) as a slightly yellow liquid with a pleasantly fruity odour.

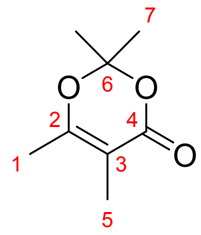

$C_8H_{12}O_3$
M = 156.18 g•mol^{-1}

TLC: R_f (pentane/Et_2O 2/1) = 0.29

^{1}H-NMR (400 MHz, $CDCl_3$, δ in ppm): 1.97 (q, J = 0.8 Hz, 3H, H1), 1.81 (q, J = 0.8 Hz, 3H, H5), 1.64 (s, 6H, H7).

^{13}C-NMR (100 MHz, $CDCl_3$, δ in ppm): 162.9 (C4), 162.8 (C2), 104.8 (C6), 100.5 (C3), 25.2 (C7), 17.6 (C1), 10.5 (C5).

The spectral data are in agreement with literature reports.[2]

Trimethyl((2,2,5-trimethyl-4-methylen-4H-1,3-dioxin-6-yl)oxy)silane 15[2]

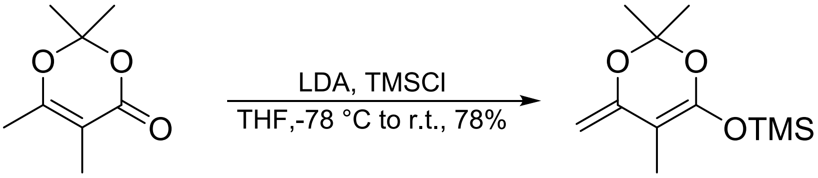

In a dried 250 mL Schlenk flask freshly distilled DIPA (16.9 mL, 120 mmol, 1.2 eq.) is dissolved in dry THF (70 mL) and the solution is cooled to -78 °C with a dry ice bath. *n*BuLi (2.5 M in hexanes, 48 mL, 120 mmol, 1.2 eq.) is then added dropwise. The colorless solution is warmed to r.t. and stirred for 30 min before being cooled again to -78 °C. Then 2,2,5,6-tetramethyl-4H-1,3-dioxin-4-one **S2** (15.6 g, 100 mmol, 1.0 eq.) dissolved in dry THF (30 mL) is added to the solution over the course of 20 min and is stirred for 1 h at -78 °C. To the resulting yellow reaction mixture TMSCl (14.0 mL, 110 mmol, 1.1 eq.) is added dropwise and is stirred for another 3 h at -78 °C before warming to room temperature. The solvent is removed under reduced pressure and a heat dried micro distill (with a tared Schlenk tube as receiving flask) under constant N_2 stream is connected to the reaction flask. The receiving flask is connected to a high vacuum pump and cooled to 0 °C. Distillation of the crude product ($T_{b.p.}$ = 59 °C, 0.6 mbar, heating the product to above 70 °C leads to decomposition) affords trimethyl((2,2,5-trimethyl-4-methylen-4H-1,3-dioxin-6-yl)oxy)silane **15** (17.73 g, 78 mmol, 78%) as a colorless oil.

The product can be stored for months at -20 °C under N_2 without decomposition.

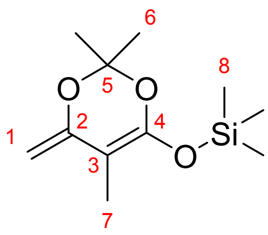

$C_{11}H_{20}O_3Si$
M = 228.36 g•mol^{-1}

^{1}H-NMR (400 MHz, $CDCl_3$, δ in ppm): 4.12 (d, J = 0.7 Hz, 1H, H1), 3.93 (d, J = 0.7 Hz, 1H, H1‘), 1.66 (s, 3H, H7), 1.51 (s, 6H, H6), 0.24 (s, 9H, H8).

^{13}C-NMR (100 MHz, $CDCl_3$, δ in ppm): 154.4 (C2), 149.6 (C4), 101.5 (C5), 84.0 (C1), 81.9 (C3), 24.7 (C6), 9.5 (C7), 0.7 (C8).

The spectral data are in agreement with literature reports.[2]

6-((2R,5S,E)-5-((*tert*-Butyldimethylsilyl)oxy)-2-hydroxyhex-3-en-1-yl)-2,2,5-trimethyl-4H-1,3-dioxin-4-one 16

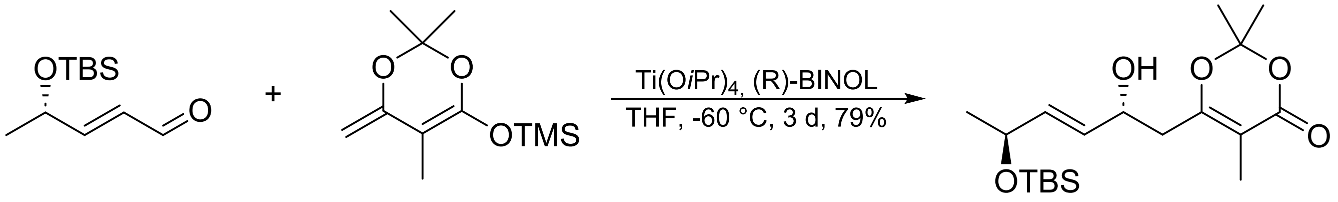

In a dried 250 mL Schlenk flask CaH_2 (215 mg) and (R)-BINOL (1.50 g, 5.25 mmol, 0.5 eq.) are mixed and cycled three times with N_2. Dry THF (31 mL) is added and the grey suspension is stirred for 5 min before Ti(O*i*Pr)$_4$ (1.55 mL, 5.25 mmol, 0.5 eq.) is added dropwise resulting in a deep orange suspension. The mixture is cooled to -60 °C with a cryostat and kept at this temperature, before dropwise addition of the Aldehyde **14** (2.24 g, 10.5 mmol, 1.0 eq.) in dry THF (11 mL). After stirring for 30 min silyl ketene acetal **15** (3.60 g, 15.8 mmol, 1.5 eq.) dissolved in dry THF (11 mL) is added dropwise and the mixture is stirred at -60 °C for 3 d. The reaction is quenched by dropwise addition of saturated NH_4Cl solution (caution! gas evolution!) until a thick yellow slurry forms. The slurry is first filtered over a small pad of celite and then washed 2x with 50 mL Et_2O. The filtrate is transferred into a separatory funnel and the phases are separated. The aqueous phase is extracted 3x with 50 mL Et_2O and the combined organic phases are dried with $MgSO_4$. After filtration and removal the solvent under reduced pressure the crude product is purified by flash chromatography (pentane:Et_2O 2:1 → 1:1 v:v) yielding 6-((2R,5S,E)-5-((*tert*-Butyldimethylsilyl)oxy)-2-hydroxyhex-3-en-1-yl)-2,2,5-trimethyl-4H-1,3-dioxin-4-one **16** (3.08 g, 8.32 mmol, 79%, 86% brsm) as a colorless oil with fruity odor.

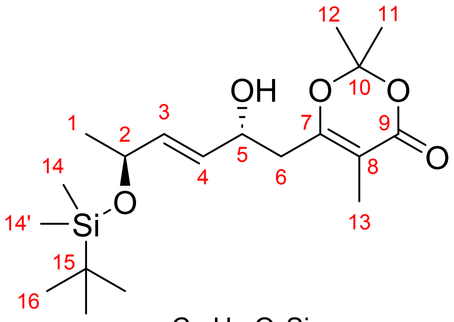

$C_{19}H_{34}O_5Si$
M = 370.56 g/mol

TLC: R_f (pentane/Et_2O 1/1) = 0.19

$[\alpha]_{20}^{D}$ = +15.3 (c = 0.98, $CHCl_3$)

^{1}H-NMR (400 MHz, $CDCl_3$, δ in ppm): 5.76 (ddd, J = 15.5 Hz, 4.5 Hz, 0.5 Hz, 1H, H3), 5.68 (ddd, J = 15.5 Hz, 6.2 Hz, 1.0 Hz, 1H, H4), 4.47-4.40 (m, 1H, H5), 4.36-4.28 (m, 1H, H2), 2.59 (dd, J = 14.1 Hz, 8.0 Hz, 1H, H6), 2.49 (dd, J = 14.1 Hz, 5.2 Hz, 1H, H6‘), 1.85 (s, 3H, H13), 1.66 (brs, 1H, OH), 1.67 (s, 3H, H12), 1.66 (s, 3H, H11), 1.20 (d, J = 6.4 Hz, 3H, H1), 0.89 (s, 9H, H16), 0.06 (s, 3H, H14), 0.05 (s, 3H, H14‘).

^{13}C-NMR (100 MHz, $CDCl_3$, δ in ppm): 162.7 (C9), 162.3 (C7), 136.9 (C3), 129.5 (C4), 105.1 (C10), 102.6 (C8), 70.0 (C5), 68.2 (C2), 38.9 (C6), 26.0 (C16), 25.7 (C12), 25.1 (C11), 24.4 (C1), 18.4 (C15), 10.6 (C13), -4.5 (C14), -4.6 (C14‘).

HRMS: calculated for $C_{19}H_{33}O_5Si$ $[M-H]^-$: 369.2103, measured for $C_{19}H_{33}O_5Si$ $[M-H]^-$: 369.2109.

Mosher ester analysis (**Table S1**) confirmed the stereocenter at C5 as *R*-configured.

Mosher ester analysis of 6-((2R,5S,E)-5-((*tert*-Butyldimethylsilyl)oxy)-2-hydroxyhex-3-en-1-yl)-2,2,5-trimethyl-4H-1,3-dioxin-4-one 16[4]

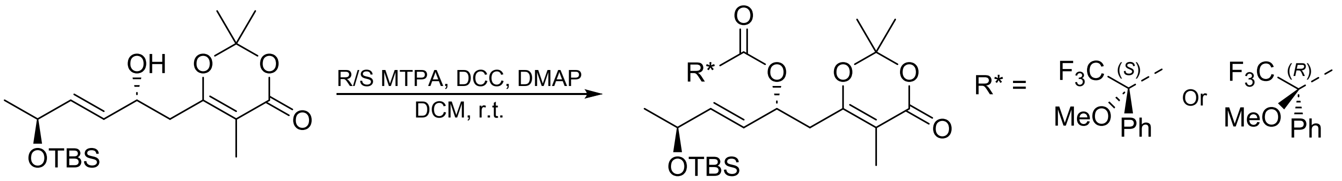

To the Alcohol **16** (10.0 mg, 27 µmol, 1.0 eq.) in a 5 mL flask is added S(-)-MTPA (19.6 mg, 84 µmol, 3.1 eq.) and cycled 3x with N_2. The mixture is dissolved in dry DCM (1 mL) followed by the subsequent addition of DCC (17.3 mg, 84µmol, 3.1 eq.) and DMAP (11.5 mg, 84 µmol, 3.1 eq.). The resulting colorless suspension is stirred overnight at room temperature. The precipitate is filtered off and washed with 10 mL Et_2O. The filtrate is concentrated under reduced pressure and the resulting crude product is purified by flash chromatography (pentane: Et_2O 3:1 v:v) yielding the S configurated ester **S3** (13.2 mg, 22.5 µmol, 83%) as a colorless oil.
Analogous esterification with R(+)-MTPA affords the corresponding R ester **S4** (12.4 mg, 21.1 µmol, 78%) as a colorless oil.

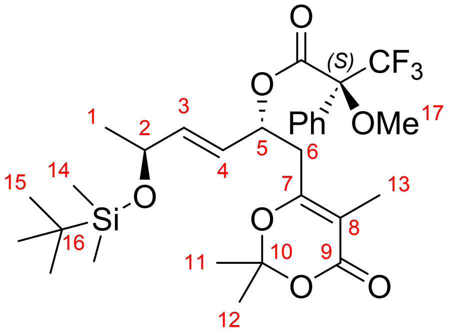

TLC: R_f (pentane/ Et_2O 2/1) = 0.31
^{1}H-NMR (400 MHz, $CDCl_3$, δ in ppm): 7.50-7.44 (m, 2H, ArH), 7.43-7.34 (m, 3H, ArH), 5.88 (ddd, J = 15.3 Hz, 4.3 Hz, 0.5 Hz, 1H, H3), 5.79-5.70 (m, 1H, H5), 5.60 (ddd, J = 15.3 Hz, 7.8 Hz, 1.5 Hz, 1H, H4), 4.30 (qdd, J = 6.5 Hz, 4.3 Hz, 1.4 Hz, 1H, H2), 3.49 (s, 3H, H17), 2.84 (dd, J = 14.5 Hz, 8.3 Hz, 1H, H6), 2.57 (dd, J = 14.5 Hz, 5.2 Hz, 1H, H6'), 1.78 (s, 3H, H13), 1.67 (s, 3H, H11), 1.59 (s, 3H, H12), 1.16 (d, J = 6.5 hz, 3H, H1), 0.87 (s, 9H, H15), 0.03 (s, 3H, H14), 0.00 (s, 3H, H14').

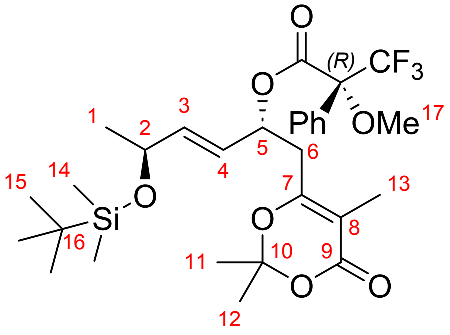

TLC: R_f (pentane/Et_2O 2/1) = 0.31
^{1}H-NMR (400 MHz, $CDCl_3$, δ in ppm): 7.51-7.45 (m, 2H, ArH), 7.41-7.34 (m, 3H, ArH), 5.95 (dd, J = 14.3 Hz, 4.2 Hz, 1H, H3), 5.74-5.69 (m, 1H, H5), 5.69 (ddd, J = 14.3 Hz, 8.0 Hz, 1.4 Hz, 1H, H4), 4.37-4.30 (m, 1H, H2), 3.50 (s, 3H, H17), 2.79 (dd, J = 14.4 Hz, 7.7 Hz, 1H, H6), 2.51 (dd, J = 14.4 Hz, 4.9 Hz, 1H, H6'), 1.69 (s, 3H, H13), 1.60 (s, 3H, H11), 1.58 (s, 3H, H12), 1.17 (d, J = 6.5 Hz, 3H, H1), 0.89 (s, 9H, H15), 0.05 (s, 3H, H14), 0.03 (s, 3H, H14').

Table S1. Mosher ester analysis of **16**.

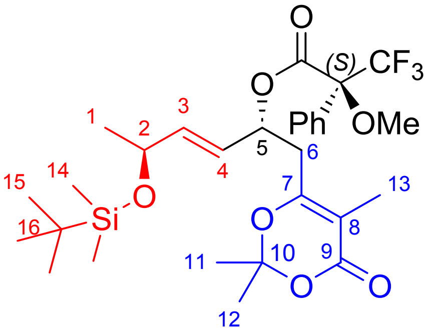

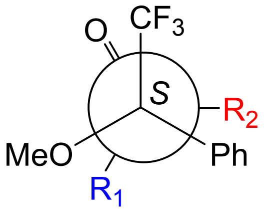

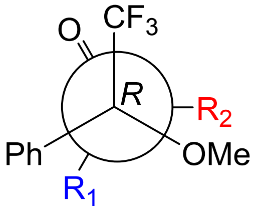

H	δ S-ester [ppm]	δ R-ester [ppm]	$\Delta\delta^{SR}$ (δ_S- δ_R) [ppm]	[Hz]
H13	1.78	1.69	+0.09	+36
H6'	2.57	2.51	+0.06	+24
H6	2.84	2.79	+0.05	+20
H11	1.63	1.60	+0.03	+12
H12	1.59	1.58	+0.01	+4
H1	1.16	1.17	-0.01	-4
H15	0.87	0.89	-0.02	-8
H14	0.03	0.05	-0.02	-8
H14'	0.00	0.03	-0.03	-12
H2	4.30	4.34	-0.04	-16
H3	5.88	5.95	-0.07	-28
H4	5.60	5.69	-0.09	-36

(R)-6-((S,E)-3-((*tert*-butyldimethylsilyl)oxy)but-1-en-1-yl)-4-methoxy-3-methyl-5,6-dihydro-2H-pyran-2-one 17

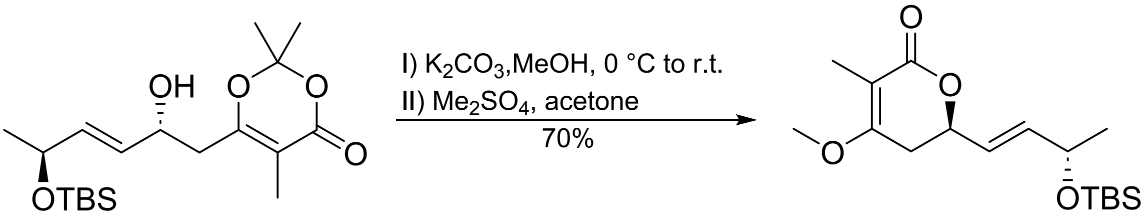

In a 250 mL round bottom flask allylalcohol **16** (3.02 g, 8.15 mmol, 1.0 eq.) is dissolved in MeOH (81 mL) and cooled to 0 °C with an ice bath. K_2CO_3 (2.25 g, 16.3 mmol, 2.0 eq.) is then added in one portion and the colorless suspension is stirred overnight resulting in a yellow solution. The reaction mixture is concentrated under reduced pressure with a rotary evaporator until a highly viscous yellow resin is obtained. The resin is suspended in acetone (81 mL) and stirred vigorously before Me_2SO_4 (1.55 mL, 16.3 mmol, 2.0 eq.) is added dropwise to the suspension. After stirring for 2 h the orange suspension is quenched with 25 w% NH_4OH solution and stirred for an additional 10 min. The biphasic mixture is transferred into a separatory funnel, the phases are separated and the aqueous phase is extracted 3x with 50 mL Et_2O. The combined organic phases are washed once with saturated NH_4Cl solution and dried with $MgSO_4$. After filtration and removal the solvent under reduced pressure the crude product is purified by flash chromatography (pentane:Et_2O 3:1 → 1:1 v:v) affording (R)-6-((S,E)-3-((*tert*-butyldimethylsilyl)oxy)but-1-en-1-yl)-4-methoxy-3-methyl-5,6-dihydro-2H-pyran-2-one **17** (1.85 g, 5.67 mmol, 70%) as a yellow oil.

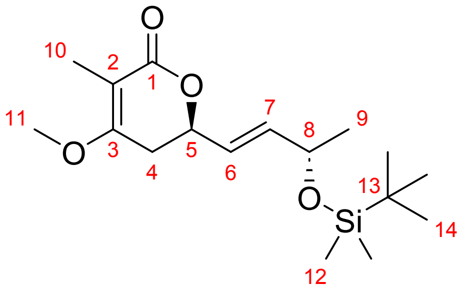

$C_{17}H_{30}O_4Si$
M = 326.51 g/mol

TLC: R_f (pentane/Et_2O 1/1) = 0.36.

$[\alpha]^D_{20}$ = +37.1 (c = 1.05, $CHCl_3$)

^{1}H-NMR (400 MHz, $CDCl_3$, δ in ppm): 5.87 (ddd, J = 15.4 Hz, 4.6 Hz, 1.0 Hz, 1H, H7), 5.76 (ddd, J = 15.4 Hz, 6.3 Hz, 1.5 Hz, 1H, H6), 4.80 (ddddd, J = 10.9 Hz, 6.3 Hz, 4.4 Hz, 1.0 Hz, 0.7 Hz, 1H, H5), 4.35 (qddd, J = 6.4 Hz, 4.6 Hz, 1.5 Hz, 0.7 Hz, 1H, H8), 3.79 (s, 3H, H11), 2.64 (ddq, J = 17.0 Hz, 4.5 Hz, 1.2 Hz, 1H, H4), 2.55 (ddq, J = 17.0 Hz, 11.0 Hz, 2.0 Hz, 1H, H4‘), 1.78 (dd, J = 2.0 Hz, 1.2 Hz, 3H, H10), 1.21 (d, J = 6.4 Hz, 3H, H9), 0.90 (s, 9H, H14), 0.06 (s, 3H, H12), 0.05 (s. 3H, H12‘).

^{13}C-NMR (100 MHz, $CDCl_3$, δ in ppm): 168.4 (C1), 165.1 (C3), 139.0 (C7), 125.0 (C6), 103.8 (C2), 74.6 (C5), 68.2 (C8), 55.6 (C11), 29.7 (C4), 26.0 (C14), 24.4 (C9), 18.4 (C13), 9.0 (C10), -4.5 (C12), -4.6 (C12‘).

HRMS: calculated for $C_{17}H_{29}O_4Si$ $[M-H]^-$: 325.1841, measured for $C_{17}H_{29}O_4Si$ $[M-H]^-$: 325.1847.

(R)-6-((S,E)-3-hydroxybut-1-en-1-yl)-4-methoxy-3-methyl-5,6-dihydro-2H-pyran-2-one S5

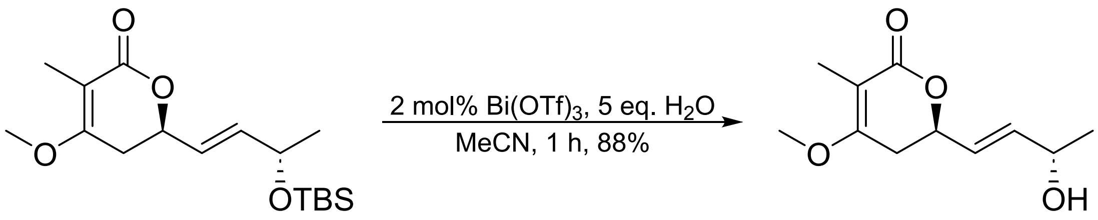

In a 50 mL round bottom flask lactone **17** (1.53 g, 4.7 mmol, 1.0 eq.) is dissolved in MeCN (24 mL) at room temperature. To this H_2O (420 µL, 23.5 mmol, 5.0 eq.) and $Bi(OTf)_3$ (62 mg, 94 µmol, 2 mol%) are added subsequently resulting in an opaque solution. After stirring the reaction for 1 h, saturated NH_4Cl solution is added and the biphasic mixture is transferred into a separatory funnel. The phases are separated and the aqueous phase is extracted 3x with 20 mL Et_2O. The combined organic phases are washed with saturated NaCl solution and dried with $MgSO_4$. After filtration and removal the solvent under reduced pressure the crude product is purified by flash chromatography (pentane:acetone 2:1 v:v) affording (R)-6-((S,E)-3-hydroxybut-1-en-1-yl)-4-methoxy-3-methyl-5,6-dihydro-2H-pyran-2-one S5 (875 mg, 4.1 mmol, 88%) as a colorless oil.

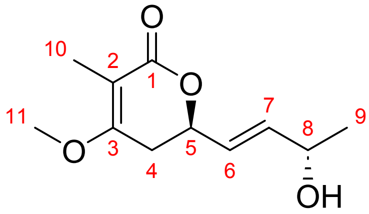

$C_{11}H_{16}O_4$
M = 212.25 g•mol^{-1}

TLC: R_f (pentane/acetone 2/1) = 0.18.
$[\mathbf{a}]^{\mathbf{D}}_{\mathbf{20}}$ = +75.2 (c = 1.09, $CHCl_3$)
^{1}H-NMR (400 MHz, $CDCl_3$, δ in ppm): 5.95 (ddd, J = 15.6 Hz, 5.5 Hz, 0.9 Hz, 1H, H7), 5.81 (ddd, J = 15.6 Hz, 6.2 Hz, 1.1 Hz, 1H, H6), 4.81 (ddddd, J = 11.1 Hz, 6.0 Hz, 4.4 Hz, 1.5 Hz, 0.8 Hz, 1H, H5), 4.37 (quint, J = 6.0 Hz, 1H, H8), 3.79 (s, 3H, H11), 2.67 (ddq, J = 17.1 Hz, 4.3 Hz, 1.2 Hz, 1H, $H4_{eq}$), 2.56 (ddq, J = 17.1 Hz, 11.2 Hz, 2.1 Hz, 1H, $H4_{ax}$), 1.78 (dd, J = 2.1 Hz, 1.2 Hz, 3H, H10), 1.64 (brs, 1H, OH), 1.29 (dd, J = 6.5 Hz, 0.8 Hz, 3H, H9).
^{13}C-NMR (100 MHz, $CDCl_3$, δ in ppm): 168.2 (C1), 165.1 (C3), 138.3 (C7), 126.2 (C6), 103.7 (C2), 74.4 (C5), 67.9 (C8), 55.6 (C11), 29.7 (C4), 23.4 (C9), 9.0 (C10).
HRMS: calculated for $C_{11}H_{15}O_4$ [M-H]$^-$: 211.0976, measured for $C_{11}H_{h15}O_4$ [M-H]$^-$: 211.0976.

(S,E)-4-((R)-4-methoxy-5-methyl-6-oxo-3,6-dihydro-2H-pyran-2-yl)but-3-en-2-yl 2,2,2-trifluoroacetate 6

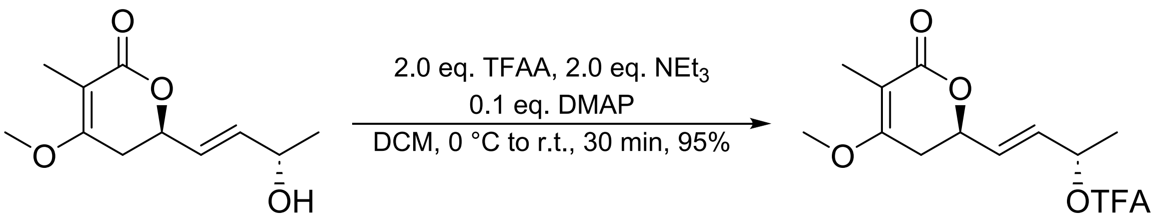

A 100 mL round bottom flask under N_2 is charged with the alcohol **S5** (1.06 g, 5.0 mmol, 1.0 eq.) and dry DCM (50 mL). DMAP (61 mg, 0.5 mmol, 0.1 eq.) and freshly distilled NEt_3 (1.39 mL, 10.0 mmol, 2.0 eq.) are subsequently added and the colorless mixture is cooled to 0 °C with an ice bath. Afterwards TFAA (1.39 mL, 10.0 mmol, 2.0 eq) is added dropwise and the yellow solution is allowed to reach room temperature. After stirring for 30 min, 1N HCl (20 mL) is added and stirred vigorously before being transferred into a separatory funnel. The phases are separated and the aqueous phase is extracted 2x with 30 mL DCM. The combined organic phases are washed with a saturated $NaHCO_3$ solution and dried with $MgSO_4$. After filtration over a 2 cm pad of silica gel 60 and removal of the solvent under reduced pressure the crude product is purified by flash chromatography (pentane:acetone 2:1 v:v) affording (S,E)-4-((R)-4-methoxy-5-methyl-6-oxo-3,6-dihydro-2H-pyran-2-yl)but-3-en-2-yl 2,2,2-trifluoro-acetate **6** (1.46 g, 4.7 mmol, 95%) as a slightly yellow oil.

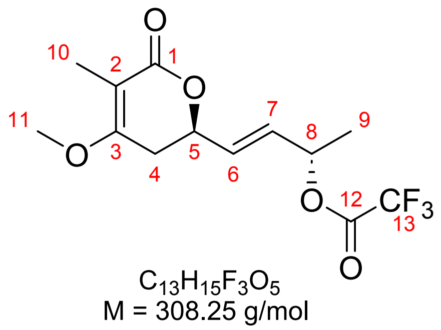

TLC: R_f (pentane/acetone 2/1) = 0.79

$[\alpha]^{D}_{20}$ = +6.2 (c = 0.97, $CHCl_3$)

^{1}H-NMR (400 MHz, $CDCl_3$, δ in ppm): 5.95 (dd, J = 15.7 Hz, 5.4 Hz, 1H, H7), 5.90 (dd, J = 15.7 Hz, 4.7 Hz, 1H, H6), 5.54 (dq, J = 6.5 Hz, 5.4 Hz, 1H, H8), 4.83 (dtr, J = 11.5 Hz, 4.7 Hz, 1H, H5), 3.80 (s, 3H, H11), 2.67 (ddq, J = 17.0 Hz, 4.4 Hz, 1.0 Hz, 1H, $H4_{eq}$), 2.54 (ddq, J = 17.0 Hz, 11.5 Hz, 2.0 Hz, 1H, $H4_{ax}$), 1.79 (dd, J = 1.9 Hz, 0.9 Hz, 3H, H10), 1.47 (d, J = 6.5 Hz, 3H, H9).

^{13}C-NMR (100 MHz, $CDCl_3$, δ in ppm): 167.7 (C1), 164.8 (C3), 156.8 (q, J = 42.5 Hz, C12), 130.9 (C7), 130.8 (C6), 114.6 (q, J = 286.2 Hz, C13), 103.9 (C2), 75.1 (C8), 73.5 (C5), 55.7 (C11), 29.5 (C4), 20.0 (C9), 9.0 (C10).

HRMS: calculated for $C_{13}H_{16}F_3O_5$ $[M+H]^+$: 309.0944, measured for $C_{13}H_{16}F_3O_5$ $[M+H]^+$: 309.0944.

(4aS,6S,7S,7aR)-2,2,2',2'-tetramethyltetrahydrospiro[furo[3,2-d][1,3]dioxine-6,4'-[1,3]dioxolan]-7-yl methanesulfonate S6[5]

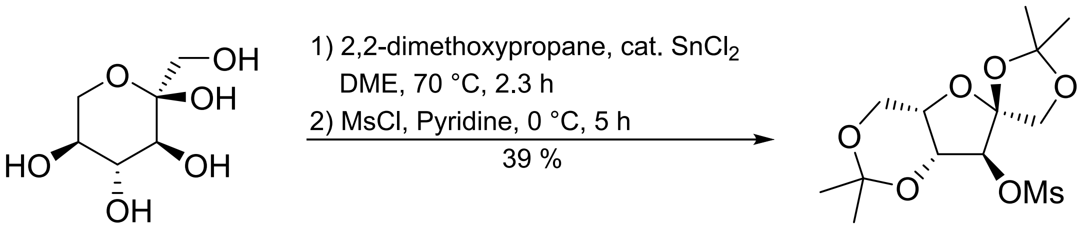

In a flame-dried 2 L three necked round bottom flask equipped with a reflux condenser and an internal thermometer, finely powdered L-sorbose (289 g, 1.60 mol, 1.0 eq.) is suspended in 2,2-dimethoxypropane (870 mL, 7.10 mol, 4.4 eq.). The suspension is heated to 70 °C under vigorous stirring. Meanwhile, $SnCl_2$ (1.44 g, 7.6 mmol, 0.46 mol%) is flame dried in a Schlenk flask and dissolved in dry DME (60 mL). The resulting $SnCl_2$ solution is added in one portion to the suspension and stirred for exactly 2.3 h. The orange reaction mixture is quenched by addition of NEt_3 (7 mL), cooled to room temperature. Unreacted L-sorbose (59 g, 21% recovered) is filtered off and washed with 100 mL ethyl acetate. The filtrate is concentrated under reduced pressure with a rotary evaporator.
The resulting orange syrup is dissolved in pyridine (700 mL), cooled to 0 °C and methanesulfonyl chloride (153 mL, 2.0 mol, 1.24 eq.) is added dropwise over 2 h. After complete addition the resulting black reaction mixture is stirred at 0 °C for 3 h (monitored by TLC) before being quenched by pouring onto 4 L of ice water. The resulting brown precipitate is filtered off and washed several times with distilled water. Recrystallization from EtOH (500 mL) affords the mesylate **S6** (213 g, 630 mmol, 39 %) as colorless needle-shaped crystals.

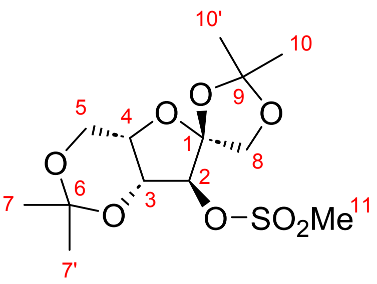

$C_{13}H_{22}O_8S$
M = 338.37 g•mol^{-1}

TLC: R_f (ethyl acetate) = 0.66
$\mathbf{T}_{\mathbf{m.p.}}$ = 123-124°C; Literature: 120-122°C
$[\alpha]^{D}_{20}$ = -30.4 (c = 1.02, $CHCl_3$); Literature: -28.0 (c = 1.06, $CHCl_3$)
^{1}H-NMR (400 MHz, $CDCl_3$, δ in ppm): 4.87 (d, J = 1.8 Hz, 1H, H2), 4.45 (dd, J = 3.2 Hz, 2.0 Hz, 1H, H3), 4.24 (d, J = 9.8 Hz, 1H, H8), 4.22 (q, J = 3.2 Hz, 1H, H4), 4.19 (d, J = 9.8 Hz, 1H, H8'), 4.00 (dd, J = 13.0 Hz, 3.2 Hz, 1H, H5), 3.91 (dd, J = 13.0 Hz, 3.1 Hz, 1H, H5'), 3.15 (s, 3H, H11), 1.54 (s, 3H, H10), 1.46 (s, 3H, H10'), 1.41 (s, 3H, H7), 1.37 (s, 3H, H7').
^{13}C-NMR (100 MHz, $CDCl_3$, δ in ppm): 111.7 (C9), 109.9 (C1), 98.4 (C6), 84.3 (C2), 73.5 (C3), 73.4 (C8), 72.2 (C4), 60.5 (C5), 39.0 (C11), 28.2 (C7), 26.1 (C10), 25.9 (C10'), 20.1 (C7').
The spectral data are in agreement with literature reports.[5]

L-Shi catalyst 19[5]

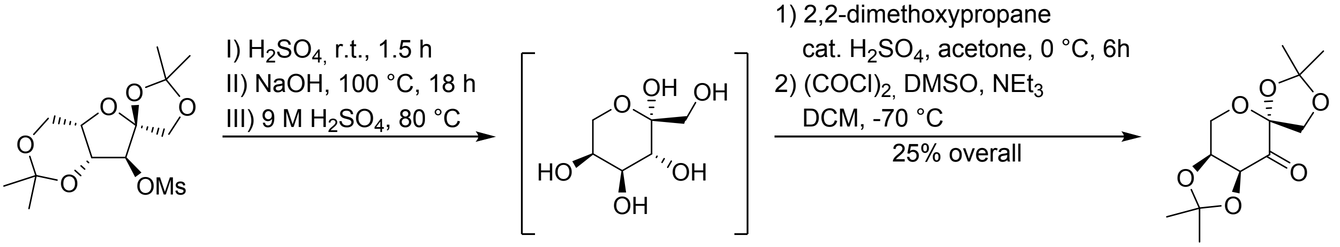

In a 2 L round bottom flask finely powdered mesylate **S6** (85.3 g, 252 mmol, 1.0 eq.) is suspended in H_2SO_4 (0.47 M, 830 mL) and stirred at room temperature for 1.5 h (until the disappearance of the spot at R_f = 0.66, ethyl acetate). The colorless suspension is then basified to pH 14 by addition of NaOH (9 M, 175 mL), resulting in a yellow solution, which is stirred overnight at 100 °C. The resulting black solution is cooled to room temperature. Meanwhile, an oil bath is preheated to 80 °C. The reaction mixture is acidified with H_2SO_4 (9 M, 40 mL) to pH 1, heated to 80 °C and monitored every 10 minutes by TLC. Once the starting material has been mostly consumed (disappearance of the spot at R_f = 0.18, ethyl acetate, caution must be taken as exceeded reactions times result in polymerization of the L-fructose), the reaction is neutralized to pH 7 by the addition of NaOH (9 M) and the solvent is removed under reduced pressure using a rotary evaporator. The brown residue is extracted five times with 300 mL of boiling EtOH. The combined organic phases are concentrated under reduced pressure affording L-fructose (55.7 g, 309 mmol, 124%) as a brown resin. To facilitate handling, the resin is subjected to lyophilization under high vacuum at -196 °C. The frozen resin is cooled to 0 °C and sequentially treated under stirring with cold acetone (420 mL), 2,2-dimethoxypropane (95 mL, 775 mmol, 3.1 eq.) and concentrated H_2SO_4 (96 w%, 3.5 mL, 65 mmol, 0.25 eq.). After stirring for 6 h the reaction is quenched by addition of NH_4OH (20 mL) and extracted with DCM (400 mL). The organic layer is washed twice with water, dried with $MgSO_4$, filtrated and concentrated under reduced pressure. The crude product is purified by flash chromatography (pentane:ethyl acetate 5:1 → 2:1 →1:1) affording (3a'S,4R,7'R,7a'R)-2,2,2',2'-tetramethyltetrahydrospiro[[1,3]dioxolane-4,6'-[1,3]dioxolo[4,5-c]pyran]-7'-ol (20.7 g, 80 mmol, 32% based on mesylate **S6**) as a colorless solid, which is used directly in the next step.

In a flame dried three necked round bottom flask equipped with a dropping funnel and an internal thermometer oxalyl chloride (7.6 mL, 88.5 mmol, 1.1 eq.) is dissolved in dry DCM (140 mL) and cooled to -70 °C. A solution of dry DMSO (13.7 mL, 192 mmol, 2.4 eq.) in dry DCM (60 mL) is added dropwise over 45 min. Afterwards a solution of acetal **S6** (20.7 g, 80.0 mmol, 1.0 eq.) in dry DCM is added dropwise over 30 min, forming a colorless suspension. After an additional 10 min, freshly distilled NEt_3 (55 mL, 400 mmol, 5.0 eq.) is added dropwise to the suspension, the cooling bath is then removed and the mixture is allowed to warm slowly to room temperature. After another 30 min the reaction is quenched by addition of distilled water. The phases are separated and the aqueous layer is extracted three times with Et_2O. The combined organic phases are dried with $MgSO_4$, filtrated and concentrated under reduced pressure. The crude product is purified by recrystallization from MeOH affording L-Shi catalyst **19** (16.4 g, 63 mmol, 79%, 25% based on mesylate **S6**) as colorless needle-shaped crystals.

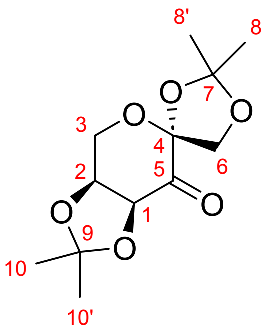

$C_{12}H_{18}O_6$
M = 258.27 g•mol^{-1}

TLC: R_f (pentane/ethyl acetate 3/1) = 0.39

T$_{m.p.}$ = 99-101 °C (Lit. 99-100 °C)

^{1}H-NMR (400 MHz, $CDCl_3$, δ in ppm): 4.72 (d, J = 5.6 Hz, 1H, H1), 4.61 (d, J = 9.5 Hz, 1H, H6), 4.55 (ddd, J = 5.5 Hz, 2.1 Hz, 0.9 Hz, 1H, H2), 4.39 (dd, J = 10.5 Hz, 2.2 Hz, 1H, H3), 4.12 (dtr, J = 13.5 Hz, 0.6 Hz, 1H, H3‘), 3.99 (d, J = 9.5 Hz, 1H, H6‘), 1.55 (s, 3H, H8), 1.46 (s, 3H, H10), 1.40 (s, 6H, H8‘+H10‘).

^{13}C-NMR (100 MHz, $CDCl_3$, δ in ppm): 197.1 (C5), 114.0 (C7), 110.8 (C9), 104.3 (C4), 78.1 (C2), 76.0 (C1), 70.1 (C6), 60.2 (C3), 27.3 (C10), 26.7 (C8), 26.2 (C10‘), 26.1 (C8‘).

The spectral data are in agreement with literature reports.[5]

(*E*)-5-iodo-5-(trimethylsilyl)pent-4-en-1-ol S7[6]

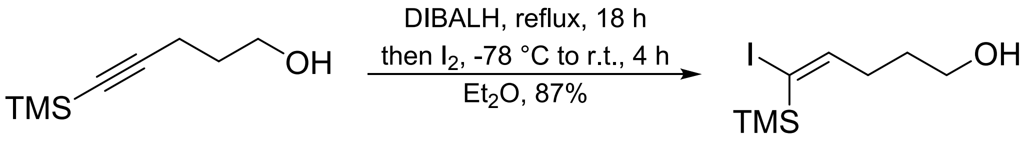

In a flame-dried 1 L three necked round bottom flask equipped with a reflux condenser and a dropping funnel, 5-(trimethylsilyl)-4-pentyn-1-ol (10.94 g, 70.0 mmol, 1.0 eq.) is dissolved in dry Et_2O (160 mL) under an inert atmosphere. A solution of DIBALH (1.0 M in hexane, 172 mL, 172 mmol, 2.45 eq.) is added dropwise at a rate sufficient to maintain steady reflux. After complete addition, the colorless reaction mixture is stirred under reflux for 18 h. The mixture is then cooled to –78 °C with a cooling bath and a solution of iodine (35.0 g, 138 mmol, 2.0 eq.) dissolved in Et_2O (200 mL) is added dropwise over the course of 1 h with vigorous stirring. The resulting dark brown, highly viscous suspension is stirred for an additional 2 h at –78 °C, then slowly warmed to room temperature by removal of the cooling bath and stirred for another 2 h. During this time, the suspension dissolves and the reaction mixture becomes colorless. The reaction is quenched by pouring the mixture onto 300 mL of ice-cold 1 N HCl with stirring. The phases are separated, and the aqueous layer is extracted three times with Et_2O. The combined organic phases are washed successively with sat. $Na_2S_2O_3$ solution, sat. $NaHCO_3$ solution, brine and dried over $MgSO_4$. After filtration and concentration under reduced pressure a yellow oil is obtained. Purification by flash chromatography (pentane:acetone 7:1 v:v) affords (E)-5-iodo-5-(trimethylsilyl)pent-4-en-1-ol **S7** (17.3 g, 61.0 mmol, 87%) as a colorless oil which crystallizes at 0 °C.

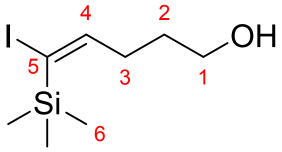

$C_8H_{17}IOSi$
M = 284.21 g•mol^{-1}

TLC: R_f (pentane/acetone 3/1) = 0.36.

^{1}H-NMR (400 MHz, $CDCl_3$, δ in ppm): 7.17 (tr, J = 8.0 Hz, 1H, H4), 3.65 (tr, J = 6.4 Hz, 2H, H1), 2.18 (dtr, J = 7.8 Hz, 7.3 Hz, 2H, H3), 1.70-1.62 (m, 2H, H2), 1.38 (brs, 1H, OH), 0.28 (s, 9H, H6).

^{13}C-NMR (100 MHz, $CDCl_3$, δ in ppm): 155.6 (C4), 107.5 (C5), 62.1 (C1), 32.1 (C3), 31.6 (C2), 1.3 (C6).

The spectral data are in agreement with literature reports.[6]

(Z)-5-(trimethylsilyl)hept-4-en-1-ol 18[6]

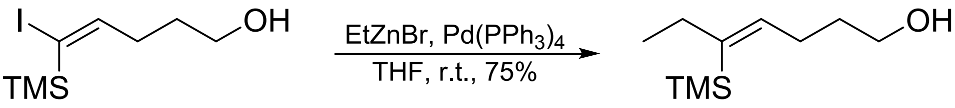

In a flame dried 250 mL three necked round bottom flask equipped with a reflux condenser and a dropping funnel Mg turnings (8.5 g, 350 mmol, 5.4 eq.) are suspended in dry THF (60 mL). A solution of EtBr (26.0 mL, 350 mmol, 5.4 eq.) in dry THF (60 mL) is added dropwise at a constant rate ensuring reflux. The resulting black suspension is refluxed for 1 h. Meanwhile $ZnCl_2$ (47.7 g, 350 mmol, 5.4 eq.) is flame dried under vacuum in a 1 l round bottom flask with a three-way stopcock. After cooling to 0 °C the $ZnCl_2$ is dissolved in dry THF (400 mL). The Grignard reagent is cooled to room temperature and added via transfer cannula to the ZnCl2 suspension at 0 °C resulting in a viscous grey suspension.

In a separate flame dried three necked round bottom flask equipped with a dropping funnel vinyl iodide **S7** (18.4 g, 65 mmol, 1.0 eq.) is dissolved in dry THF (50 mL) and $Pd(PPh_3)_4$ (2.25 g, 1.95 mmol, 0.03 eq.) is added. The previously prepared EtZnBr solution (300 mL) is added dropwise at room temperature. Towards the end of the addition a yellow suspension is formed indicating complete reaction. The reaction is quenched by addition of saturated NH_4Cl solution and the biphasic mixture is transferred into a separatory funnel. The phases are separated and the aqueous phase is extracted three times with ethyl acetate (200 mL). The combined organic phases are dried with $MgSO_4$. After filtration and removal of the solvent under reduced pressure the crude product is purified by flash chromatography (pentane:acetone 10:1 → 5:1) affording (Z)-5-(trimethylsilyl)hept-4-en-1-ol **18** (9.10 g, 49 mmol, 75%, 85 w% pure by ^{1}H-NMR) as a colorless oil

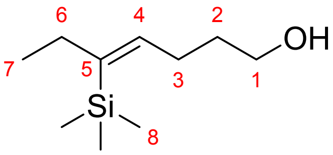

$C_{10}H_{22}OSi$
186.37 g•mol^{-1}

TLC: R_f (pentane/acetone 5/1) = 0.50

^{1}H-NMR (400 MHz, $CDCl_3$, δ in ppm): 5.93 (trtr, J = 7.5 Hz, 1.1 Hz, 1H. H4), 3.66 (brtr, J = 6.2 Hz, 1H, H1), 2.34-2.16 (m, 2H, H3), 2.11-2.01 (m, 2H, H6), 1.69-1.60 (m, 2H, H2), 1.31 (brs, 1H, OH), 0.95 (tr, J = 7.5 Hz, 3H, H7), 0.14 (s, 9H, H8).

^{13}C-NMR (100 MHz, $CDCl_3$, δ in ppm): 140.8 (C4), 129.9 (C5), 62.9 (C1), 33.3 (C2), 31.1 (C6), 28.5 (C3), 15.6 (C7), 0.5 (C8).

The spectral data are in agreement with literature reports.[6]

3-((2S,3R)-3-ethyl-3-(trimethylsilyl)oxiran-2-yl)propan-1-ol 20[6]

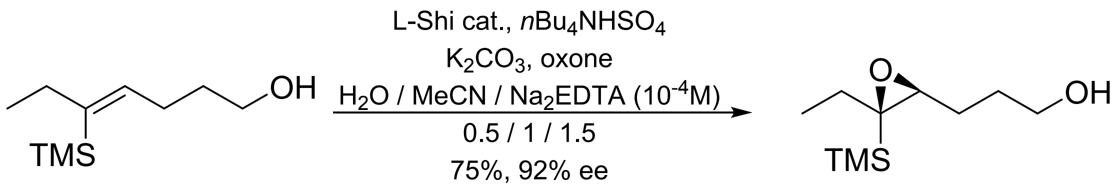

In a 1L three necked round bottom flask with two identical dropping funnels the alkene **18** (85 w% prepared according to literature procedure, 2.26g, 10.3 mmol, 1.0 eq.) is dissolved in MeCN (175 mL) and cooled to 0°C. To this solution the L-Shi catalyst **19** (prepared from L-sorbose according to Shi *et al.*, 2.20 g, 8.52 mmol, 0.7 eq.), *n*Bu_4NHSO_4 (496 mg, 1.46 mmol, 0.12 eq) and a solution of Na_2EDTA (10^{-4} M, 125 mL) are added in succession and the solution is stirred vigorously. Then a freshly prepared solution of K_2CO_3 (11.27 g, 81.5 mmol, 6.7 eq.) in H_2O (85 mL) and oxone (11.97 g, 19.5 mmol, 1.6 eq.) in Na_2EDTA (10^{-4} M, 85 mL) are filled into the respective dropping funnels. The solutions are simultaneously added dropwise to the reaction mixture (caution! a too one-sided addition of either solution reduces the yield dramatically) over the course of 1 h. After complete addition the mixture is stirred for another hour before being quenched with 100 mL pentane. The triphasic mixture is transferred into a separatory funnel and the lower aqueous layer is separated. The aqueous layer is extracted 3x with 100 mL pentane and once with 100 mL ethyl acetate. The combined organic phases are dried with $MgSO_4$, filtrated and concentrated under reduced pressure. The crude product is purified by flash chromatography (pentane:Et_2O 5:1 → 2:1 →1:1) affording 3-((2S,3R)-3-ethyl-3-(trimethylsilyl)oxiran-2-yl)propan-1-ol **20** (1.56 g, 7.1 mmol, 75%).
Derivatization to the PNB ester and HPLC analysis (Chiralcel OD-H, 1 mL/min, 25 °C, *n*Hex:*i*PrOH 95:5, 254 nm, t_{R1} = 7.03 min, t_{R2} = 8.25 min) indicated 92 % ee.
The enantiomer is synthesized with identical yield and ee using D-Shi catalyst instead.

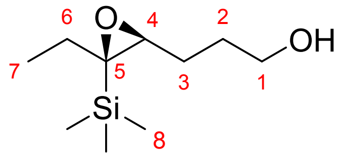

$C_{10}H_{22}O_2Si$
M = 202.37 g•mol^{-1}

TLC: R_f (pentane/Et_2O 1/1) = 0.16
^{1}H-NMR (400 MHz, $CDCl_3$, δ in ppm): 3.77-3.66 (m, 2H, H1), 2.73 (dd, J = 8.5 Hz, 3.7 Hz, 1H, H4), 1.94 (dq, J = 13.3 Hz, 7.3 Hz, 1H, H3), 1.87-1.72 (m, 4H, H3‘+H2+OH), 1.53-1.43 (m, 1H, H6), 1.16-1.06 (m, 1H, H6‘), 0.91 (tr, J = Hz, 3H, H7), 0.15 (s, 9H, H8).
^{13}C-NMR (100 MHz, $CDCl_3$, δ in ppm): 63.3 (C1), 62.9 (C4), 59.2 (C5), 30.4 (C6+C3), 27.7 (C2), 10.2 (C7), -1.0 (C8).
The spectral data are in agreement with literature reports.[6]

HPLC analysis of Epoxide 20

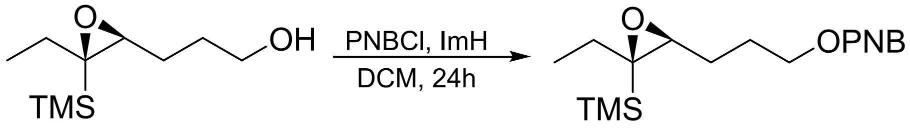

In a screw capped vial with a stirring bar 10 mg (50 µmol, 1.0 eq) of the epoxyalcohol **20** is dissolved in 2 mL DCM at room temperature. To this solution ImH (10.2 mg, 150 µmol, 3.0 eq.) and PNBCl (27.8 mg, 150 µmol, 3.0 eq.) are added successively. After stirring for 24 h, the reaction mixture is concentrated under reduced pressure and the crude product is purified by flash chromatography (pentane:Et_2O 5:1) affording the PNB ester **S8** in quantitative yield.

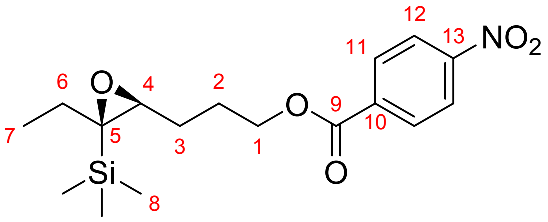

$C_{17}H_{25}NO_5Si$
M = 351.47 g•mol^{-1}

TLC: R_f (pentane/Et_2O 5/1) = 0.21

^{1}H-NMR (400 MHz, $CDCl_3$, δ in ppm): 8.29 (dtr, J = 8.8 Hz, 1.6 Hz, 2H, H12), 8.20 (dtr, J = 8.8 Hz, 1.8 Hz, 2H, H11), 4.50-4.39 (m, 2H, H1), 2.75 (dd, J = 8.0 Hz, 4.5 Hz, 1H, H4), 2.05-1.88 (m, 3H, H2+H3), 1.87-1.77 (m 1H, H3‘), 1.66-1.57 (m, 1H, H6), 1.16-1.05 (m, 1H, H6‘), 0.91 (tr, J = 7.5 Hz, 3H, H7), 0.13 (s, 9H, H8).

^{13}C-NMR (100 MHz, $CDCl_3$, δ in ppm): 164.8 (C9), 150.7 (C12), 135.8 (C10), 130.8 (C11), 123.7 (C12), 65.7 (C1), 62.7 (C4), 58.6 (C5), 30.3 (C2), 27.7 (C3), 26.6 (C6), 10.2 (C7), -1.0 (C8).

The spectral data are in agreement with literature reports.[6]

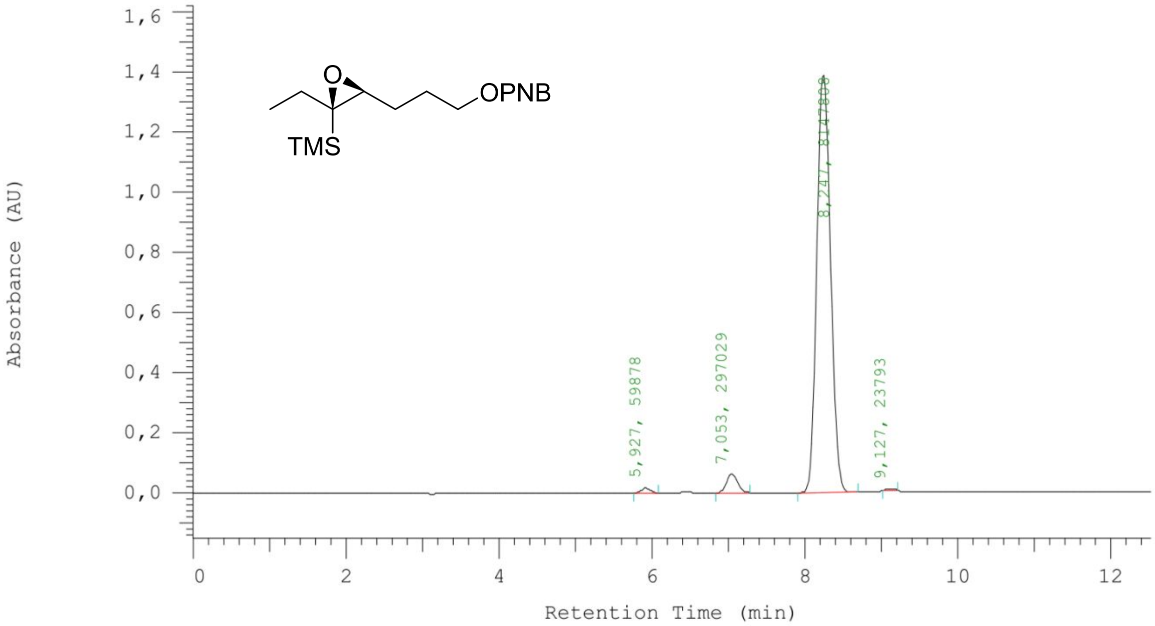

Peak	retention time [min]	area	area [%]	er
1	7.053	297029	3.5	27.4:1
2	8.247	8147808	96.5	

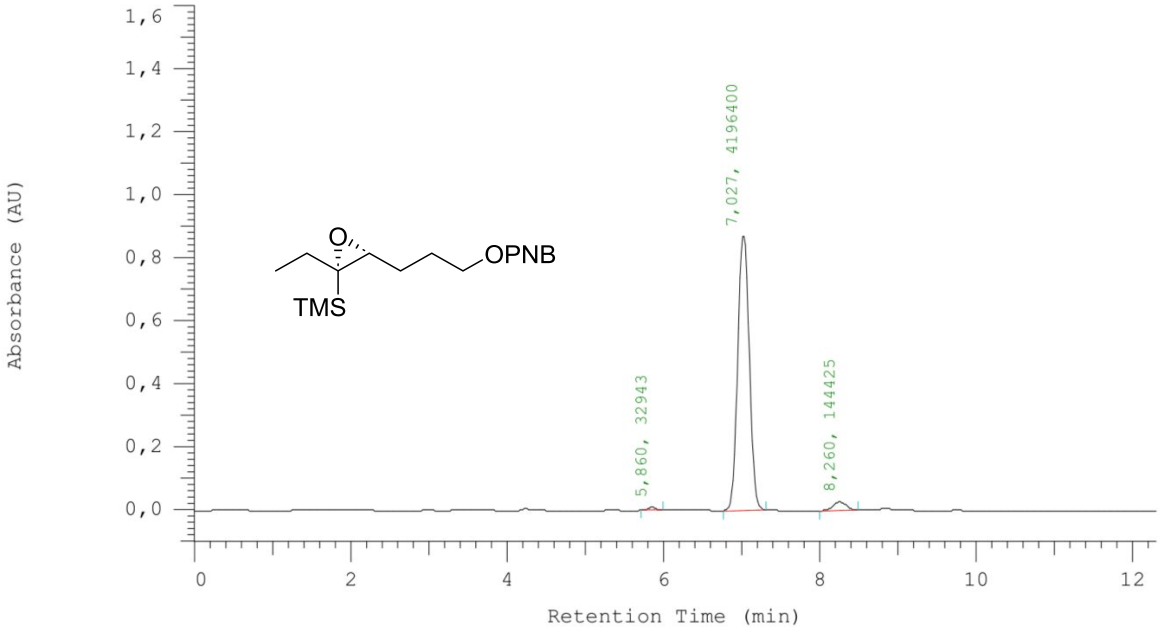

Peak	retention time [min]	area	area [%]	er
1	7.027	4196400	96.7	29.3:1
2	8.260	144425	3.3	

3-((2S,3R)-3-ethyl-3-(trimethylsilyl)oxiran-2-yl)propanal 10

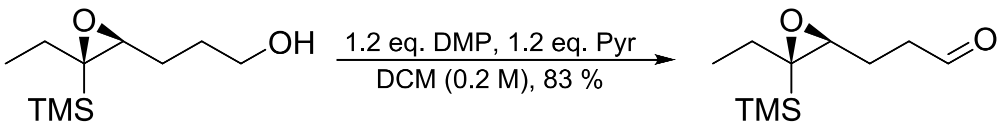

In a 50 mL round bottom flask epoxy alcohol **20** (1.20 g, 5.95 mmol, 1.0 eq.) is dissolved in 30 mL of dry DCM and cooled to 0 °C. Pyridine (575 µL, 7.14 mmol, 1.2 eq.) and DMP (3.03 g, 7.14 mmol, 1.2 eq.) are sequentially added and the yellow solution is stirred for 2 h at room temperature. The reaction is quenched by addition of both a saturated $Na_2S_2O_3$ and a $NaHCO_3$ solution and the biphasic mixture is transferred into a separatory funnel. The phases are separated and the aqueous phase is extracted 3x with 10 mL DCM. The combined organic phases are dried with $MgSO_4$. After filtration and removal of the solvent under reduced pressure the crude product is purified by flash chromatography (pentane:Et_2O 5:1) affording 3-((2S,3R)-3-ethyl-3-(trimethylsilyl)oxiran-2-yl)propanal **10** (1.01 g, 5.00 mmol, 83%) as a colorless liquid with fruity odor.

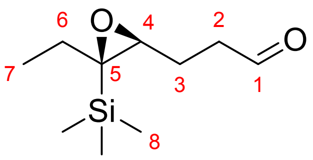

$C_{10}H_{20}O_2Si$
M = 200.35 g•mol^{-1}

TLC: R_f (pentane/Et_2O 5/1) = 0.15

^{1}H-NMR (400 MHz, $CDCl_3$, δ in ppm):9.83 (tr, J = 1.3 Hz, 1H, H1), 2.72 (dd, J = 8.5 Hz, 4.3 Hz, 1H, H4), 2.69-2.57 (m, 2H, H2), 2.04 (dddd, J = 14.6 Hz, 8.1 Hz, 6.8 Hz, 4.3 Hz, 1H, H3), 1.91 (dq, J = 13.6 Hz, 7.3 Hz, 1H, H3‘), 1.69 (dddd, J = 14.3 Hz, 8.5 Hz, 7.8 Hz, 6.6 Hz, 1H, H6), 1.15-1.05 (m, 1H, H6‘), 0.90 (tr, J = 7.5 Hz, 3H, H7), 0.14 (s, 9H, H8).

^{13}C-NMR (100 MHz, $CDCl_3$, δ in ppm): 201.4 (C1), 62.3 (C4), 59.2 (C5), 41.6 (C2), 30.3 (C6), 23.7 (C3), 10.2 (C7), -1.1 (C8).

(2S,3S,6R)-2-ethyl-2-(trimethylsilyl)-6-((trimethylsilyl)ethynyl)tetrahydro-2H-pyran-3-ol 22

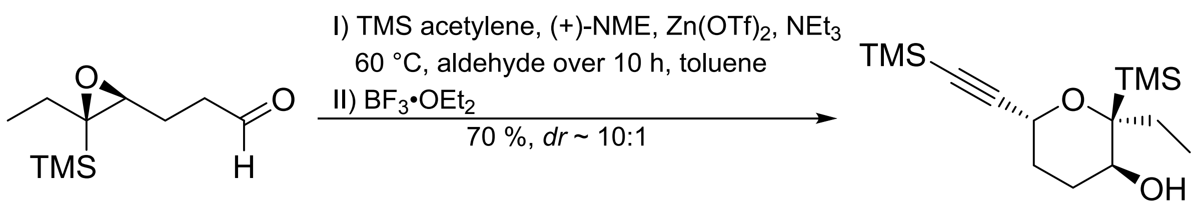

A 100 mL round bottom flask with a small reflux condenser and a three-way stopcock is charged with $Zn(OTf)_2$ (2.00 g, 5.5 mmol, 1.1 eq.) under N_2. The $Zn(OTf)_2$ is dried with a heat gun under vacuum for 10 min and the flask is purged 3 x with N_2. After cooling to room temperature (+)-NME (1.08 g, 6.0 mmol, 1.2 eq.) is added and the mixture is dissolved with dry toluene (25 mL). Then freshly distilled NEt_3 (830 µL, 6.0 mmol, 1.2 eq.) is added in one portion and the resulting biphasic colorless mixture is stirred vigorously for 2 h. Afterwards tms acetylene (3.6 mL, 25.0 mmol, 5.0 eq.) is added at once and the mixture is stirred for 15 min at room temperature before being heated to 60 °C. The aldehyde **10** (1.01 g, 5.00 mmol, 1.0 eq.) dissolved in dry toluene (25 mL) is then added

via a syringe pump over the course of 10 h (shorter addition times result in major aldol side reaction!). After the addition is complete the mixture is stirred for an additional 4 h before being quenched with saturated NH_4Cl solution. The biphasic mixture is separated and the aqueous phase is extracted 3x with 50 mL Et_2O. The combined organic phases are dried with $MgSO_4$, filtrated and concentrated under reduced pressure yielding the open chained product (2.39 g, 160 %) as a yellow oil with ~10:1 dr (separatable but not done at this point).

In a dried round bottom flask under N_2 the crude product is dissolved in dry DCM (50 mL) and cooled to 0 °C with stirring. Then BF_3•OEt_2 (634 µL 1.0 eq, 5.00 mmol) is added in one portion. After stirring for 10 min the mixture is immediately quenched with saturated $NaHCO_3$ solution and the biphasic mixture is separated. The aqueous phase is extracted 3x with 20 mL DCM and the combined organic phases are dried with $MgSO_4$. After filtration and removal of the solvent under reduced pressure the crude product is purified via flash chromatography (pentane:Et_2O 10:1) affording pure (2S,3S,6R)-2-ethyl-2-(trimethylsilyl)-6-((trimethylsilyl)ethynyl)tetrahydro-2H-pyran-3-ol **22** (952 mg, 3.18 mmol, 64%).

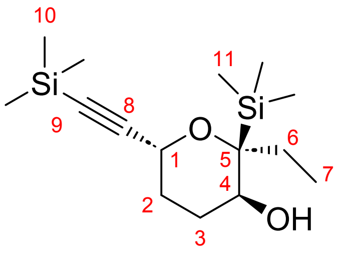

$C_{15}H_{30}O_2Si_2$
M = 298.57 g•mol^{-1}

TLC: R_f (pentane/Et_2O 10/1) = 0.18 (main product), 0.25 (epimer)

^{1}H-NMR (400 MHz, $CDCl_3$, δ in ppm): 4.24 (dd, J = 10.4 Hz, 2.3 Hz, 1H, H1), 3.70 (dtr, J = 10.2 Hz, 4.4 Hz, 1H, H4), 1.99-1.82 (m, 3H, H3+H2+H6), 1.82-1.60 (m, 3H, H2'+H3'+H6'), 1.48 (brd, J = 4.3 Hz, 1H, OH), 0.97 (tr, J = 7.4 Hz, 3H, H7), 0.20 (s, 9H, H11), 0.16 (s, 9H, H10).

^{13}C-NMR (100 MHz, $CDCl_3$, δ in ppm): 105.2 (C8), 88.5 (C9), 78.0 (C5), 71.8 (C4), 64.5 (C1), 32.0 (C2), 29.7 (C3), 29.6 (C6), 7.6 (C7), 1.0 (C11), 0.1 (C10).

HRMS: calculated for $C_{15}H_{31}O_2Si_2$ $[M+H]^+$: 299.1857, measured for $C_{15}H_{31}O_2Si_2$ $[M+H]^+$:299.1844.

(2R,3S,6R)-2-Ethyl-6-ethynyltetrahydro-2H-pyran-3-ol 24

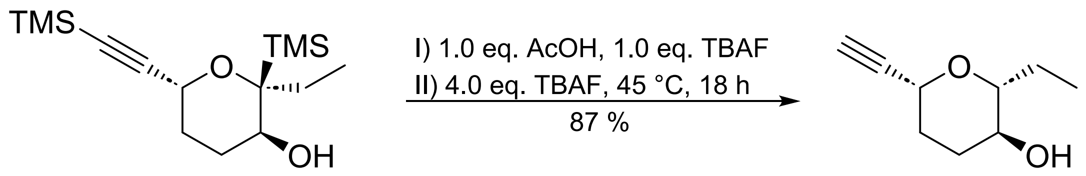

In a round bottom flask the alcohol **22** (1.05 g, 3.52 mmol, 1.0 eq.) is dissolved in THF (35 mL) at room temperature. AcOH (200 µL, 3.52 mmol, 1.0 eq) and TBAF (1.0 M in THF, 3.5 mL 3.52 mmol, 1.0 eq.) are then added sequentially and the mixture is stirred for 10 min before being quenched with saturated $NaHCO_3$ solution. The phases are separated and the aqueous phase is extracted twice with Et_2O. The organic layers are combined and dried with $MgSO_4$. After filtration and evaporation of the solvent the crude mono deprotected alcohol is transferred into a round bottom flask with a small reflux condenser and cycled with N_2 before being dissolved in dry THF (35 mL). TBAF (1.0 M in THF, 14.0 mL, 14 mmol, 4.0 eq.) is then added resulting in a slightly red solution which is stirred at 45 °C for 18 h. The wine-red mixture is quenched with saturated NH_4Cl solution and the phases are separated. The aqueous phase is extracted 3x with 20 mL Et_2O. The organic layers are combined and dried with $MgSO_4$. After filtration and removal of the solvent under reduced pressure the crude product is purified via flash

chromatography (pentane:acetone 10:1 →5:1) affording (2R,3S,6R)-2-Ethyl-6-ethynyltetrahydro-2H-pyran-3-ol **24** (474 mg, 3.07 mmol, 87%) as colorless crystals.

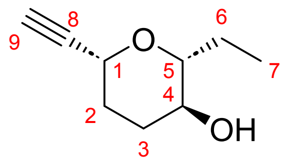

$C_9H_{14}O_2$
M = 154.21 g•mol^{-1}

TLC: R_f (pentane/acetone 5/1) = 0.18
$T_{m.p.}$ = 50-52 °C
$[\alpha]^D_{20}$ = +106.4 (c = 1.10, $CHCl_3$)
^{1}H-NMR (400 MHz, $CDCl_3$, δ in ppm): 4.08 (dtr, J = 11.4 Hz, 2.3 Hz, 1H, H1), 3.41-3.31 (m, 1H, H4), 3.00 (trd, J = 8.7 Hz, 2.6 Hz, 1H, H5), 2.45 (d, J = 2.1 Hz, 1H, H9), 2.14-2.07 (m, 1H, H3'), 1.97 (ddtr, J = 13.8 Hz, 4.9 Hz, 2.7 Hz, 1H, H2'), 1.89 (dqd, J = 14.6 Hz, 7.6 Hz, 2.8 Hz, 1H, H6'), 1.87-1.76 (m, 1H, H2), 1.56-1.43 (m, 1H, H6), 1.50-1.41 (m, 1H, H3), 1.41 (brd, J = 5.2 Hz, 1H, OH), 1.01 (tr, J = 7.5 Hz, 3H, H7).
^{13}C-NMR (100 MHz, $CDCl_3$, δ in ppm): 83.9 (C5), 82.8 (C8), 72.7 (C9), 69.5 (C4), 67.5 (C1), 32.9 (C3), 32.4 (C2), 24.9 (C6), 9.9 (C7).
HRMS: calculated for $C_9H_{15}O_2$ $[M+H]^+$: 155.1067, measured for $C_9H_{15}O_2$ $[M+H]^+$: 155.1068.

(2R,6R)-2-ethyl-6-ethynyldihydro-2H-pyran-3(4H)-one 25

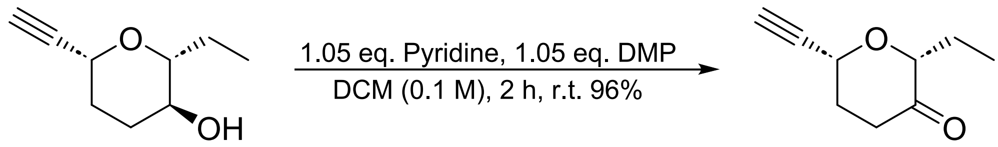

In a round bottom flask the alcohol **24** (93 mg, 600 µmol, 1.0 eq.) is dissolved in dry DCM (10 mL) and cooled to 0 °C. To this are sequentially added at once pyridine (52 µL, 630 µmol, 1.05 eq.) and DMP (267 mg, 630 µmol, 1.05 eq.) with stirring. The ice bath is removed and the reaction is stirred for an additional 2 h at room temperature. The reaction is then quenched by adding both a saturated $NaHCO_3$ and a $Na_2S_2O_3$ solution. The phases are separated and the aqueous phase is extracted 3 x with 10 mL DCM. The combined organic layers are dried with $MgSO_4$. After filtration over a 2 cm pad of silical gel 60 and removal of the solvent under reduced pressure the crude product is purified by flash chromatography (pentane:acetone 5:1) affording (2R,6R)-2-ethyl-6-ethynyldihydro-2H-pyran-3(4H)-one **25** (87.5 mg, 575 µmol, 96%) as a colorless oil, which crystallizes in the refrigerator.

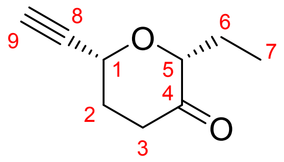

$C_9H_{12}O_2$
M = 152.19 g•mol^{-1}

TLC: R_f (pentane/acetone 5/1) = 0.36
$[\alpha]^D_{20}$ = +149.2 (c = 1.20, $CHCl_3$)
^{1}H-NMR (400 MHz, $CDCl_3$, δ in ppm): 4.52 (ddd, J = 8.0 Hz, 5.8 Hz, 2.1 Hz, 1H, H1), 3.80 (dd, J = 7.3 Hz, 4.5 Hz, 1H, H5), 2.62 (dtr, J = 16.2 Hz, 5.3 Hz, 1H, H3), 2.54 (d, J = 2.1 Hz, 1H, H9), 2.51-2.42 (m, 1H, H3'), 2.35-2.27 (m, 2H, H2), 1.89 (dqd, J = 14.9 Hz, 7.5 Hz, 4.5 Hz, 1H, H6), 1.69 (dquint, J = 14.8 Hz, 7.4 Hz, 1H, H6'), 0.98 (tr, J = 7.5 Hz, 3H, H7).
^{13}C-NMR (100 MHz, $CDCl_3$, δ in ppm): 207.5 (C4), 84.5 (C5), 81.9 (C8), 73.8 (C9), 66.0 (C1), 37.1 (C3), 32.3 (C2), 23.3 (C6), 9.9 (C7).

HRMS: calculated for $C_9H_{13}O_2$ $[M+H]^+$: 153.0910, measured for $C_9H_{13}O_2$ $[M+H]^+$ 153.0911.

(2R,3R,6R)-2-ethyl-6-ethynyl-3-methyltetrahydro-2H-pyran-3-ol 26

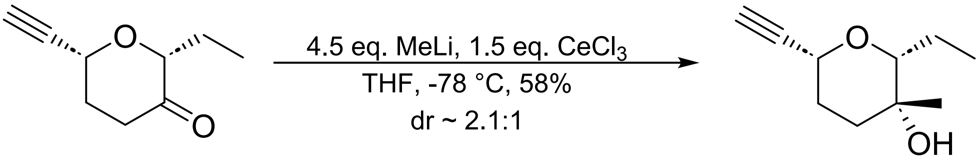

In a 25 mL Schlenk flask finely ground up $CeCl_3$•$7H_2O$ (140 mg, 375 µmol, 1.5 eq.) is stirred under high vacuum ($p < 10^{-2}$ mbar). The powder is heated stepwise (10 °C/h until 80 °C, 12 h at 80 °C, 10 °C/h until 140 °C and finally 2 h at 140 °C) to ensure that the remaining water is removed without major CeOCl formation. After cooling to room temperature the $CeCl_3$ is then suspended in dry THF (4 mL) and the resulting colorless suspension is stirred for 2 h before being cooled to -78 °C. Then MeLi (1.6 M in Et_2O, 700 µL, 1.13 mmol, 4.5 eq.) is slowly added dropwise to the reaction mixture resulting in a yellow to brown suspension. After stirring for another 30 min the ketone **25** dissolved in dry THF (2 mL) is added dropwise and the reaction mixture is stirred for an additional 10 min before quenching it with saturated NH_4Cl solution. The phases are separated, the aqueous phase is extracted 3 x with Et_2O (10 mL) and the combined organic phases are dried with $MgSO_4$. After filtration and removal of the solvent under reduced pressure the crude product is purified via flash chromatography (pentane:acetone 10:1) affording (2R,3R,6R)-2-ethyl-6-ethynyl-3-methyltetrahydro-2H-pyran-3-ol **26** (24.2 mg, 143 µmol, 58%) as the major product and (2R,3S,6R)-2-ethyl-6-ethynyl-3-methyltetrahydro-2H-pyran-3-ol **S9** (11.1 mg, 66 µmol, 26%) as a separable side product.

The absolute configuration of **26** was indirectly confirmed by the crystal structure of compound **S11**.

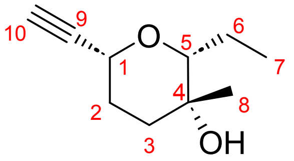

$C_{10}H_{16}O_2$
M = 168.24 g•mol^{-1}

TLC: R_f (pentane/acetone 5/1) = 0.35

[a]$^D_{20}$ = +104.5 (c = 1.26, $CHCl_3$)

^{1}H-NMR (500 MHz, $CDCl_3$, δ in ppm): 4.09 (dtr, J = 11.9 Hz, 2.3 Hz, 1H, H1), 3.05 (dd, J = 9.8 Hz, 2.7 Hz, 1H, H5), 2.49 (d, J = 2.2 Hz, 1H, H10), 2.32 (s, 1H, OH), 1.97 (dddd, J = 14.1 Hz, 13.7 Hz, 11.9 Hz, 4.3 Hz, 1H, $H2_{ax}$), 1.82-1.73 (m, 2H, $H2_{eq}$+H3), 1.62 (dqd, J = 15.0 Hz, 7.5 Hz, 2.7 Hz, 1H, H6), 1.57-1.47 (m, 2H, H3'+H6'), 1.10 (s, 3H, H8), 0.99 (tr, J = 7.5 Hz, 3H, H7).

^{13}C-NMR (100 MHz, $CDCl_3$, δ in ppm): 86.4 (C5), 82.9 (C9), 73.0 (C10), 68.3 (C1), 68.2 (C4), 37.5 (C3), 29.2 (C2), 24.5 (C8), 21.7 (C6), 11.0 (C7)

HRMS: calculated for $C_{10}H_{17}O_2$ $[M+H]^+$: 169.1223, measured for $C_{10}H_{17}O_2$ $[M+H]^+$: 169.1222.

(2R,3S,6R)-2-ethyl-6-ethynyl-3-methyltetrahydro-2H-pyran-3-ol S9

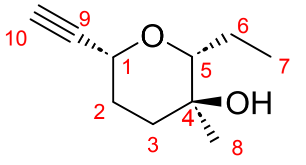

$C_{10}H_{16}O_2$
M = 168.24 g•mol^{-1}

TLC: R_f (pentane/acetone 5/1) = 0.31
$\mathbf{T_{m.p.}}$ = 62-63 °C
$[\alpha]^D_{20}$ = +109.5 (c = 0.95, $CHCl_3$)
^{1}H-NMR (400 MHz, $CDCl_3$, δ in ppm): 4.09 (ddd, J = 10.4 Hz, 3.4 Hz, 2.1 Hz, 1H, H1), 2.99 (dd, J = 10.1 Hz, 2.0 Hz, 1H, H5), 2.46 (d, J = 2.1 Hz, 1H, H10), 1.93-1.77 (m, 3H, H2+H2'+H3), 1.69 (dqd, J = 14.1 Hz, 7.6 Hz, 2.0 Hz, 1H, H6), 1.62-1.53 (m, 1H, H3'), 1.36 (ddq, J = 14.1 Hz, 10.1 Hz, 7.2 Hz, 1H, H6'), 1.44-1.31 (brs, 1H, OH), 1.21 (s, 3H, H8), 1.01 (tr, J = 7.4 Hz, 3H, H7).
^{13}C-NMR (100 MHz, $CDCl_3$, δ in ppm): 86.9 (C5), 82.9 (C9), 72.7 (C10), 69.5 (C4), 68.2 (C1), 39.8 (C3), 31.8 (C2), 21.8 (C6), 20.4 (C8), 11.4 (C7).
HRMS: calculated for $C_{10}H_{17}O_2$ $[M+H]^+$: 169.1223, measured for $C_{10}H_{17}O_2$ $[M+H]^+$: 169.1224.

triethyl(((2R,3R,6R)-2-ethyl-6-ethynyl-3-methyltetrahydro-2H-pyran-3-yl)oxy)silane 27

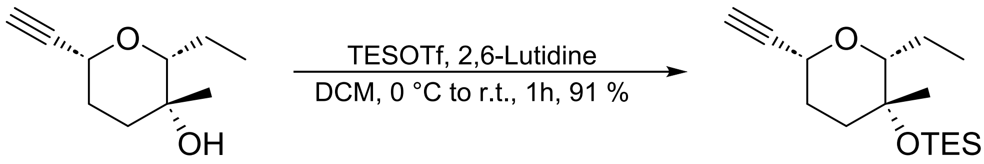

To a stirred solution of the tertiary alcohol **26** (124.7 mg, 740 µmol, 1.0 eq.) in dry DCM (7 mL) are sequentially added dry 2,6-Lutidine (340 µL, 2.96 mmol, 4.0 eq.) and TESOTf (330 µL, 1.48 mmol, 2.0 eq.) at 0 °C. The colorless solution is stirred at room temperature for one hour before being quenched with a saturated NH_4Cl solution. The phases are separated and the aqueous phase is extracted 3x with 10 mL Et_2O. The organic layers are combined and dried with $MgSO_4$. After filtration and evaporation of the solvent under reduced pressure the crude product is purified via flash chromatography (pentane:Et_2O 60:1) affording triethyl(((2R,3R,6R)-2-ethyl-6-ethynyl-3-methyltetrahydro-2H-pyran-3-yl)oxy)silane **27** (190 mg, 673 µmol, 91%) as a colorless oil.

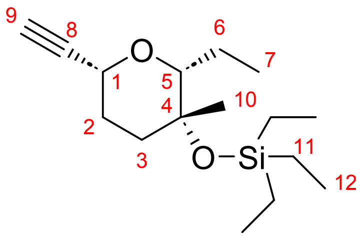

$C_{16}H_{30}O_2Si$
M = 282.50 g•mol^{-1}

TLC: R_f (pentane/Et_2O 30/1) = 0.25
$[\alpha]^D_{20}$ = +80.2 (c = 1.11, $CHCl_3$)
^{1}H-NMR (400 MHz, $CDCl_3$, δ in ppm): 4.05 (dtr, J = 11.7 Hz, 2.1 Hz, 1H, H1), 2.87 (dd, J = 9.5 Hz, 2.4 Hz, 1H, H5), 2.43 (d, J = 2.1 Hz, 1H, H9), 2.15 (trd, J = 13.4 Hz, 11.7 Hz, 3.8 Hz, 1H, H2), 1.76 (trd, J = 13.7 Hz, 3.3 Hz, 1H, H3), 1.68-1.42 (m, 4H, H2'+H3'+H6), 1.12 (s, 3H, H10), 0.98 (tr, J = 7.4 Hz, 3H, H7), 0.96 (tr, J = 7.9 Hz, 9H, H12), 0.60 (q, J = 7.9 Hz, 6H, H11).
^{13}C-NMR (100 MHz, $CDCl_3$, δ in ppm): 87.2 (C5), 83.6 (C8), 72.2 (C9), 70.5 (C4), 67.9 (C1), 38.4 (C3), 29.1 (C2), 26.8 (C10), 22.1 (C6), 11.3 (C7), 7.3 (C12), 6.9 (C11).
HRMS: calculated for $C_{16}H_{31}O_2Si$ $[M+H]^+$:283.2088 measured for $C_{16}H_{31}O_2Si$ $[M+H]^+$:283.2090.

triethyl(((2R,3S,6R)-2-ethyl-6-ethynyl-3-methyltetrahydro-2H-pyran-3-yl)oxy)silane S10

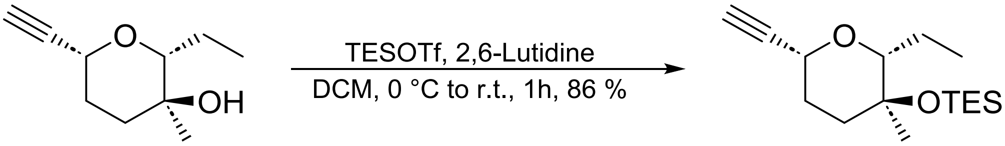

To a stirred solution of the tertiary alcohol **S9** (42 mg, 246 µmol, 1.0 eq.) in dry DCM (2.5 mL) are sequentially added 2,6-Lutidine (113 µL, 984 µmol, 4.0 eq.) and TESOTf (111 µL, 492 µmol, 2.0 eq.) at 0 °C. The colorless solution is stirred at room temperature for one hour before being quenched with a saturated NH_4Cl solution. The phases are separated and the aqueous phase is extracted 3x with 10 mL Et_2O. The organic layers are combined and dried with $MgSO_4$. After filtration and evaporation of the solvent under reduced pressure the crude product is purified via flash chromatography (pentane:Et_2O 30:1) affording triethyl(((2R,3S,6R)-2-ethyl-6-ethynyl-3-methyltetrahydro-2H-pyran-3-yl)oxy)silane **S10** (59.8 mg, 212 µmol, 86%) as a colorless oil.

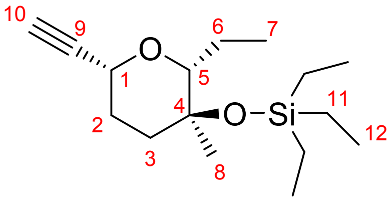

$C_{16}H_{30}O_2Si$
M = 282.50 g•mol^{-1}

TLC: R_f (pentane/Et_2O 30/1) = 0.37

$[\alpha]^{D}_{20}$ = +80.9 (c = 1.15, $CHCl_3$)

^{1}H-NMR (400 MHz, $CDCl_3$, δ in ppm): 4.12-4.05 (m, 1H, H1), 2.94 (dd, J = 10.0 Hz, 1.8 Hz, 1H, H5), 2.45 (d, J = 2.1 Hz, 1H, H10), 1.89-1.70 (m, 4H, H2+H3+H6), 1.67-1.58 (m, 1H, H3‘), 1.29 (ddq, J = 14.2 Hz, 10.0 Hz, 7.2 Hz, 1H, H6‘), 1.20 (s, 3H, H8), 0.98 (tr, J = 7.4 Hz, 3H, H7), 0.93 (tr, J = 7.8 Hz, 9H, H12), 0.56 (q, J = 7.8 Hz, 6H, H11).

^{13}C-NMR (100 MHz, $CDCl_3$, δ in ppm): 87.6 (C5), 83.2 (C9), 72.5 (C10), 71.9 (C4), 68.2 (C1), 40.3 (C3), 31.9 (C2), 21.8 (C6), 20.8 (C8), 11.6 (C7), 7.2 (C12), 7.0 (C11).

HRMS: calculated for $C_{16}H_{31}O_2Si$ $[M+H]^+$: 283.2088, measured for $C_{16}H_{31}O_2Si$ $[M+H]^+$: 283.2077.

***tert*-butyl(((2R,3S,6R)-2-ethyl-6-ethynyl-3-methyltetrahydro-2H-pyran-3-yl)oxy)dimethylsilane S11**

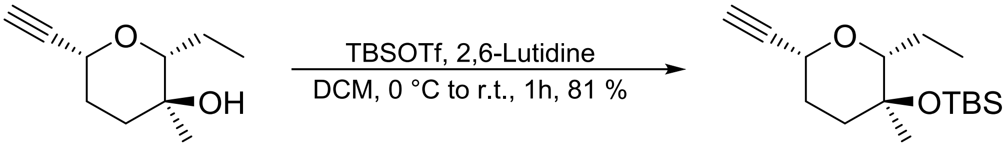

To a stirred solution of the tertiary alcohol **S9** (78.9 mg, 469 µmol, 1.0 eq.) in dry DCM (5 mL) are sequentially added 2,6-Lutidine (216 µL, 1.88 mmol, 4.0 eq.) and TBSOTf (216 µL, 938 µmol, 2.0 eq.) at 0 °C. The colorless solution is stirred at room temperature for one hour before being quenched with a saturated NH_4Cl solution. The phases are separated and the aqueous phase is extracted 3x with 10 mL Et_2O. The organic layers are combined and dried with $MgSO_4$. After filtration and evaporation of the solvent under reduced pressure the crude product is purified via flash chromatography (pentane:Et_2O 60:1) affording *tert*-butyl(((2R,3S,6R)-2-ethyl-6-ethynyl-3-

methyltetrahydro-2H-pyran-3-yl)oxy)dimethylsilane **S11** (108 mg, 380 µmol, 81%) as a colorless oil which crystallizes at -20 °C.

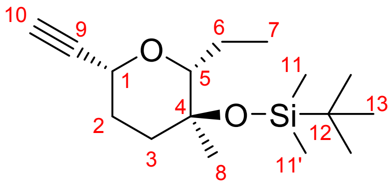

$C_{16}H_{30}O_2Si$
M = 282.50 g•mol^{-1}

TLC: R_f (pentane/Et_2O 30/1) = 0.42.

$T_{m.p.}$ = 24-25 °C

^{1}H-NMR (400 MHz, $CDCl_3$, δ in ppm): 4.09 (ddd, J = 10.7 Hz, 2.5 Hz, 2.3 Hz, 1H, H1), 2.93 (dd, J = 10.0 Hz, 1.6 Hz, 1H, H5), 2.45 (d, J = 2.2 Hz, 1H, H10), 1.89-1.78 (m, 3H, H2+H3), 1.75 (dqd, J = 14.1 Hz, 7.6 Hz, 1.6 Hz, 1H, H6), 1.67-1.57 (m, 1H, H3‘), 1.28 (ddq, J = 14.1 Hz, 10.0 Hz, 7.4 Hz, 1H, H6‘), 1.20 (s, 3H, H8), 0.98 (tr, J = 7.4 Hz, 3H, H7), 0.84 (s, 9H, H13), 0.07 (s, 3H, H11), 0.07 (s, 3H, H11‘).

^{13}C-NMR (100 MHz, $CDCl_3$, δ in ppm): 87.6 (C5), 83.2 (C9), 72.5 (C10), 72.0 (C4), 68.2 (C1), 40.3 (C3), 31.8 (C2), 25.9 (C13), 21.8 (C6), 20.6 (C8), 18.2 (C12), 11.7 (C7), -1.7 (C11), -1.9 (C11‘).

HRMS: calculated for $C_{16}H_{31}O_2Si$ $[M+H]^+$: 283.2088, measured for $C_{16}H_{31}O_2Si$ $[M+H]^+$: 283.2093.

triethyl(((2R,3R,6R)-2-ethyl-3-methyl-6-((E)-1-(tributylstannyl)prop-1-en-2-yl)tetrahydro-2H-pyran-3-yl)oxy)silane 7

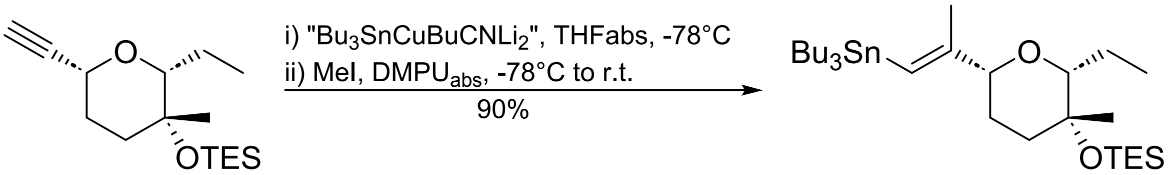

A 10 mL round bottom flask charged with CuCN (72.3 mg, 804 µmol, 1.2 eq.) is dried under vacuum with a heat gun and cycled 3x with N_2. To the dried CuCN is added dry THF (3.3 mL) and the resulting colorless suspension is cooled to -78 °C with stirring. Afterwards *n*BuLi (2.5 M in hexanes, 640 µL, 1.61 mmol, 2.4 eq.) is added dropwise. The dry ice bath is then removed and the suspension is stirred for one minute resulting in a slightly yellow solution before being cooled again to -78 °C. Then *n*Bu_3SnH (500 µL, 1.61 mmol, 2.4 eq.) is slowly added dropwise to the reaction mixture resulting in an intensive yellow solution after a few seconds. After stirring for an additional 10 min alkyne **27** (190 mg, 670 µmol, 1.0 eq.) dissolved in dry THF (1.4 mL) is added dropwise (along the walls of the flask, not directly!) to the reaction mixture. After stirring for 15 min MeI (840 µL, 13.4 mmol, 20.0 eq.) and dry DMPU (290 µL) are subsequently added dropwise resulting in a wine-red solution. The ice bath is removed and the reaction mixture allowed to warm to room temperature over the course of an hour. While warming up the color of the solution changes from wine-red to orange and finally to colorless indicating complete reaction. The reaction is quenched with a 9:1 mixture of saturated NH_4Cl and NH_4OH solution. The phases are separated and the aqueous phase is extracted 3x with 20 mL Et_2O. The organic layers are combined and dried with $MgSO_4$. After filtration and evaporation of the solvent under reduced pressure the crude product is purified via reversed phase flash chromatography on a Büchi Reveleris® chromatography System with a Büchi FlashPure Select C18 30 µm spherical cartridge (MeCN → MeCN:DCM 60:40) affording triethyl(((2R,3R,6R)-2-ethyl-3-methyl-6-((E)-1-(tributylstannyl)prop-1-en-2-yl)tetrahydro-2H-pyran-3-yl)oxy)silane **7** (356 mg, 606 µmol, 90%) as a colorless oil

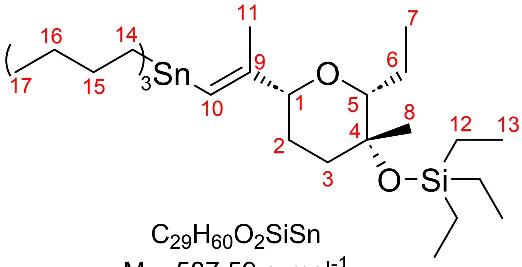

$C_{29}H_{60}O_2SiSn$
M = 587.59 g•mol^{-1}

TLC: R_f (RP-18 DCM/MeCN 40/60) = 0.32

^{1}H-NMR (400 MHz, $CDCl_3$, δ in ppm): 5.75 (s, 1H, H10), 3.67 (brd, J = 10.9 Hz, 1H, H1), 2.91 (dd, J = 9.0 Hz, 2.9 Hz, 1H, H5), 1.78 (s, 3H, H11), 1.85-1.71 (m, 2H, H2+H3), 1.64-1.44 (m, 10H, H2‘+H3‘+H6+H15), 1.37 (m, 6H, H16), 1.11 (s, 3H, H8), 0.96-0.85 (m, 27H, H7+H13+H14+H17), 0.58 (q, J = 7.8 Hz, 6H, H12).

^{13}C-NMR (100 MHz, $CDCl_3$, δ in ppm): 155.5 (C9), 121.4 (C10), 86.2 (C5), 83.3 (C1), 71.0 (C4), 39.0 (C3), 30.8 (1C, C15), 29.4 (2C, C15), 27.6 (1C, C16), 27.5 (2C, C16), 27.2 (C2), 26.9 (C8), 22.3 (C6), 21.2 (C11), 13.9 (C17), 11.3 (C7), 10.2 (2C, C14), 10.1 (1C, C14), 7.3 (C13), 7.1 (C12).

HRMS: calculated for $C_{29}H_{61}O_2SiSn$ $[M+H]^+$: 589.3457, measured for $C_{29}H_{61}O_2SiSn$ $[M+H]^+$: 589.3120.

triethyl(((2R,3S,6R)-2-ethyl-3-methyl-6-((E)-1-(tributylstannyl)prop-1-en-2-yl)tetrahydro-2H-pyran-3-yl)oxy)silane S12

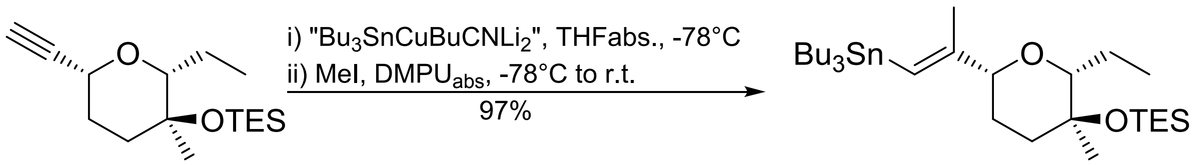

Carbostannylation is carried out analogous to the procedure described above using alkyne **S10** (58.2 mg, 206 μmol, 1.0 eq.). Purification via reversed phase flash chromatography on a Büchi Reveleris® chromatography System with a Büchi FlashPure Select C18 30 μm spherical cartridge (MeCN → MeCN:DCM 60:40) triethyl(((2R,3S,6R)-2-ethyl-3-methyl-6-((E)-1-(tributylstannyl)prop-1-en-2-yl)tetrahydro-2H-pyran-3-yl)oxy)silane **S12** (113 mg, 193 μmol, 97%) as a colorless oil.

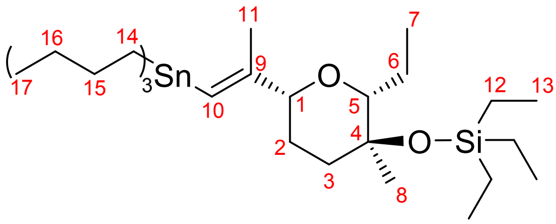

$C_{29}H_{60}O_2SiSn$
M = 587.59 g•mol^{-1}

TLC: R_f (RP-18 DCM/MeCN 40/60) = 0.30.

^{1}H-NMR (400 MHz, $CDCl_3$, δ in ppm): 5.73 (s, 1H, H10), 3.73 (brd, J = 11.0 Hz, 1H, H1), 2.99 (dd, J = 9.9 Hz, 2.0 Hz, 1H, H5), 1.86 (ddd, J = 11.5 Hz, 4.2 Hz, 2.5 Hz, 1H, H3), 1.78 (s, 3H, H11), 1.76-1.64 (m, 3H, H2+H3‘+H6), 1.57-1.41 (m, 7H, H2‘+H15), 1.36-1.25 (m, 7H, H6‘+H16), 1.17 (s, 3H, H8), 0.96-0.85 (m, 27H, H7+H13+H14+H17), 0.57 (q, J = 7.8 Hz, 6H H12).

^{13}C-NMR (100 MHz, $CDCl_3$, δ in ppm): 154.6 (C9), 121.9 (C10), 86.8 (C5), 83.4 (C1), 72.6 (C4), 40.9 (C3), 30.8 (1C, C15), 29.8 (C2), 29.4 (2C, C15), 27.6 (1C, C16), 27.5 (2C, C16), 22.0 (C6), 21.5 (C11), 21.1 (C8), 13.9 (C17), 11.6 (C7), 10.2 (2C, C14), 10.1 (1C, C14), 7.3 (C13), 7.1 (C12).

14-TES-Jerangolid B 28

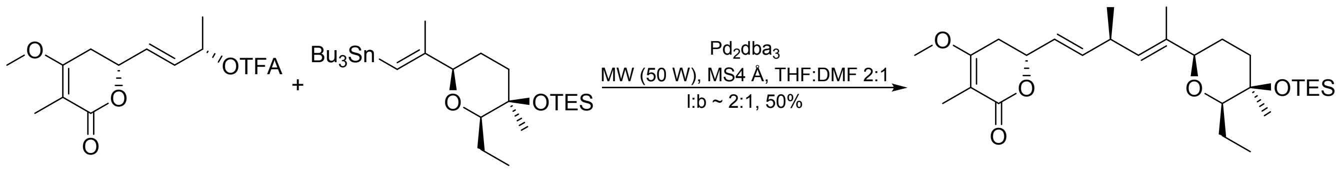

An oven dried microwave vial charged with the lactone **6** (87.7 mg, 285 µmol, 1.0 eq.) and vinylstannane **7** (176 mg, 1.05 eq. 300 µmol) is cycled three times with N_2. The mixture is dissolved in a 2:1 mixture of dry THF (2 mL) and DMF (1 mL) before 120 mg of finely ground activated MS 4 Å is added. After stirring for a few minutes Pd_2dba_3 (13.0 mg, 14 µmol, 5 mol%) is added and the resulting dark red suspension is placed into a microwave. The sample is irradiated (50 W) and cooled in pulses for 1 minute at a time. After a total of 20 min (i.e. ten full cycles) Pd black precipitates out of solution. The blackish yellow suspension is filtered over a small pad of celite and washed with Et_2O. After evaporation of the solvent the crude product is purified via flash chromatography (pentane:acetone 15:1 +2 vol% NEt_3) affording 14-TES-Jerangolid B **28** (46.9 mg, 95 µmol, 33%) as a colorless resin.

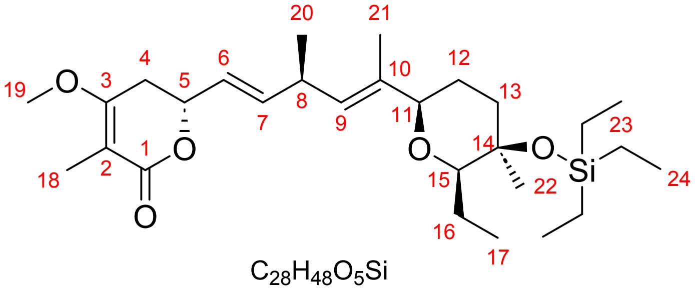

$C_{28}H_{48}O_5Si$
M = 492.77 g•mol^{-1}

TLC: R_f (pentane/acetone 10/1) = 0.14

^{1}H-NMR (500 MHz, $CDCl_3$, δ in ppm): 5.78 (ddd, J = 15.6 Hz, 6.3 Hz, 1.0 Hz, 1H, H7), 5.51 (ddd, J = 15.6 Hz, 8.9 Hz, 1.3 Hz, 1H, H6), 5.21 (dquint, J = 9.1 Hz, 1.2 Hz, 1H, H9), 4.73 (brddd, J = 11.0 Hz, 6.6 Hz, 4.2 Hz, 1H, H5), 3.77 (s, 3H, H19), 3.57 (brd, J = 1.06 Hz, 1H, H11), 3.15-3.07 (m, 1H, H8), 2.88 (dd, J = 7.1 Hz, 5.1 Hz, 1H, H15), 2.60 (ddq, J = 17.5 Hz, 4.3 Hz, 1.0 Hz, 1H, $H4_{eq}$), 2.52 (ddq, J = 17.5 Hz, 11.3 Hz, 2.0 Hz, 1H, $H4_{ax}$), 1.84-1.78 (m, 1H, H12), 1.75-1.70 (m, 1H, H13), 1.76 (dd, J = 1.6 Hz, 1.1 Hz, 3H, H18), 1.64 (d, J = 1.4 Hz, 3H, H21), 1.57-1.44 (m, 3H, H13'+H16), 1.40-1.34 (m, 1H, H12'), 1.11 (s, 3H, H22), 1.05 (d, J = 6.8 Hz, 3H, H20), 0.95 (tr, J = 7.9 Hz, 9H, H24), 0.94 (tr, J = 7.5 Hz, 3H, H17), 0.57 (q, J = 7.9 Hz, 6H, H23).

^{13}C-NMR (125 MHz, $CDCl_3$, δ in ppm): 168.5 (C1), 165.3 (C3), 139.5 (C7), 136.9 (C10), 127.4 (C9), 124.7 (C6), 103.6 (C2), 86.4 (C15), 82.0 (C11), 75.2 (C5), 71.0 (C14), 55.6 (C19), 38.8 (C13), 34.4 (C8), 29.8 (C4), 26.8 (C12), 26.6 (C22), 22.2 (C16), 20.5 (C20), 13.3 (C21), 11.3 (C17), 9.0 (C18), 7.3 (C23), 7.0 (C24).

HRMS: calculated for $C_{28}H_{49}O_5Si$ $[M+H]^+$: 493.3344, measured for $C_{28}H_{49}O_5Si$ $[M+H]^+$: 493.3355.

Additionally 23.6 mg (47.9 µmol, 17%) 6-((S,2E)-2-((2R,5R,6R)-6-ethyl-5-methyl-5-((triethylsilyl)oxy)tetrahydro-2H-pyran-2-yl)hepta-2,5-dien-4-yl)-4-methoxy-3-methyl-5,6-dihydro-2H-pyran-2-one **29** (branched side product) were isolated as a 4:1 isomeric mixture.

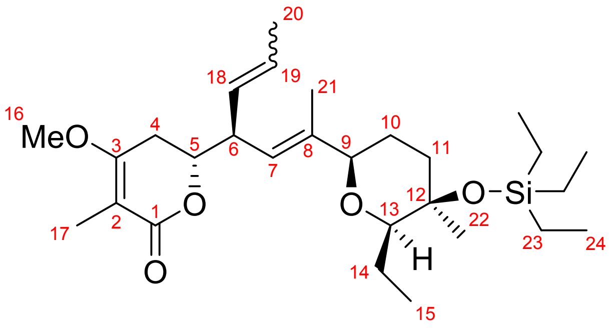

$C_{28}H_{48}O_5Si$
M = 492.77 g•mol^{-1}

TLC: R_f (pentane/acetone 10/1) = 0.20
^{1}H-NMR (400 MHz, $CDCl_3$, δ in ppm): 5.61-5.49 (m, 2H, H18+H19), 5.46 (brd, J = 9.6 Hz, 1H, H7, isomer 1), 5.40 (brd, J = 9.6 Hz, 1H, H7, isomer 2), 4.28 (dtr, J = 12.3 Hz, 4.2 Hz, 1H, H5, isomer 1), 4.22-4.15 (m, 1H, H5, isomer 2), 3.75 (s, 3H, H16), 3.60 (brd, J = 10.6 Hz, 1H, H9), 3.39-3.33 (m, 1H, H6, isomer 1), 3.26-3.19 (m, 1H, H6, isomer 2), 2.89 (brdd, J = 9.3 Hz, 3.2 Hz, 1H, H13), 2.52 (ddq, J = 17.0 Hz, 12.4 Hz, 2.0 Hz, 1H, $H4_a$), 2.40 (brdd, J = 17.0 Hz, 4.1 Hz, 1H, $H4_b$), 1.83-1.77 (m, 1H, $H10_a$), 1.76 (s, 3H, H17), 1.77-1.71 (m, 1H, $H11_a$), 1.68 (m, 3H, H20), 1.67 (m, 3H, H21), 1.63-1.42 (m, 4H, H14+$H10_b$+$H11_b$), 1.12 (s, 3H, H22), 0.95 (tr, J = 7.9 Hz, 9H, H24), 0.93 (tr, J = 7.2 Hz, 3H, H15), 0.61-0.54 (m, 6H, H23).
^{13}C-NMR (100 MHz, $CDCl_3$, δ in ppm): 168.9 (C1), 166.0 (C3), 140.3 (C8), 128.7 (C18), 127.6 (C19), 120.6 (C7), 103.3 (C2), 86.3 (C13), 81.5 (C9), 77.4 (C5), 71.0 (C12), 55.4 (C16), 44.0 (C6), 38.8 (C11), 26.80 (C10), 26.76 (C22), 26.0 (C4), 22.2 (C14), 18.3 (C20), 13.9 (C21), 11.2 (C15), 9.0 (C17), 7.3 (C24), 7.0 (C23).
HRMS: calculated for $C_{28}H_{49}O_5Si$ $[M+H]^+$: 493.3344, measured for $C_{28}H_{49}O_5Si$ $[M+H]^+$: 493.3342.

Epi-14-TES-Jerangolid B S13

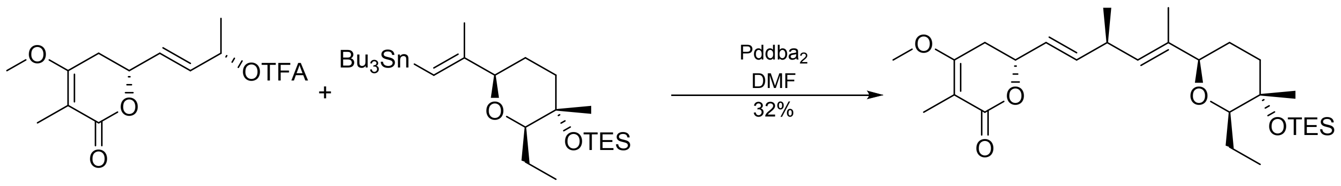

An oven dried microwave vial charged with the lactone **6** (59.0 mg, 191 µmol, 1.0 eq.) and vinylstannane **S12** (126 mg, 205 µmol. 1.10 eq.) is cycled three times with N_2. The mixture is dissolved in dry DMF (200 µL) and stirred for a few minutes before $Pddba_2$ (5.5 mg, 9.6 µmol, 5 mol%) is added. After stirring for 24 h at room temperature the reaction becomes deep red and Pd black precipitates out of solution. The blackish yellow suspension is filtered over a small pad of celite and washed with Et_2O. After evaporation of the solvent the crude product is purified via flash chromatography (pentane:acetone 10:1 +2 vol% NEt_3) affording epi-14-TES-Jerangolid B **S13** (29.9 mg, 60.7 µmol, 32%) as a slightly yellow resin.

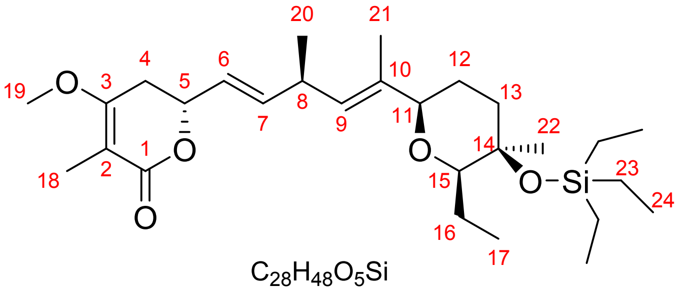

$C_{28}H_{48}O_5Si$
M = 492.77 g•mol^{-1}

TLC: R_f (pentane/acetone 10/1) = 0.19

^{1}H-NMR (400 MHz, MeOH-d$_4$, δ in ppm): 5.83 (ddd, J = 15.6 Hz, 6.5 Hz, 0.9 Hz, 1H, H7), 5.60 (ddd, J = 15.6 Hz, 6.6 Hz, 1.3 Hz, 1H, H6), 5.28 (dquint, J = 9.0 Hz, 1.1 Hz, 1H, H9), 4.85-4.78 (m, 1H, H5), 3.85 (s, 3H, H11), 3.70 (brdd, J = 11.0 Hz, 2.2 Hz, 1H, H11), 3.22-3.13 (m, 1H, H8), 3.02 (dd, J = 10.0 Hz, 1.8 Hz, 1H, H15), 2.86 (ddq, J = 17.6 Hz, 4.4 Hz, 1.1 Hz, 1H, H4$_a$), 2.64 (ddq, J = 17.6 Hz, 11.4 Hz, 1.9 Hz, 1H, H4$_b$), 1.92 (m, 1H, H13$_a$), 1.84-1.76 (m, 1H, H16$_a$), 1.75-1.70 (m, 1H, H13$_b$), 1.72 (dd, J = 1.9 Hz, 0.9 Hz, 3H, H18), 1.67 (d, J = 1.2 Hz, 3H, H21), 1.66-1.54 (m, 2H, H12), 1.31-1.21 (m, 1H, H16$_b$), 1.21 (s, 3H, H22), 1.10 (d, J = 6.8 Hz, 3H, H20), 0.99 (tr, J = 8.0 Hz, 9H, H24), 0.97 (tr, J = 7.2 Hz, 3H, H17), 0.62 (q, J = 8.0 Hz, 6H, H23).

^{13}C-NMR (100 MHz, MeOH-d$_4$, δ in ppm): 171.4 (C1), 169.4 (C3), 140.2 (C7), 137.3 (C10), 129.3 (C9), 126.4 (C6), 102.7 (C2), 88.2 (C15), 83.9 (C11), 76.9 (C5), 73.6 (C14), 56.3 (C19), 41.8 (C13), 35.9 (C8), 30.3 (C4), 30.3 (C12), 22.8 (C16), 21.4 (C22), 21.0 (C20), 13.3 (C21), 11.8 (C17), 8.8 (C18), 7.8 (C23), 7.4 (C24).

HRMS: calculated for $C_{28}H_{49}O_5Si$ [M+H]$^+$: 493.3344, measured for $C_{28}H_{49}O_5Si$ [M+H]$^+$: 493.3347.

Jerangolid B 1

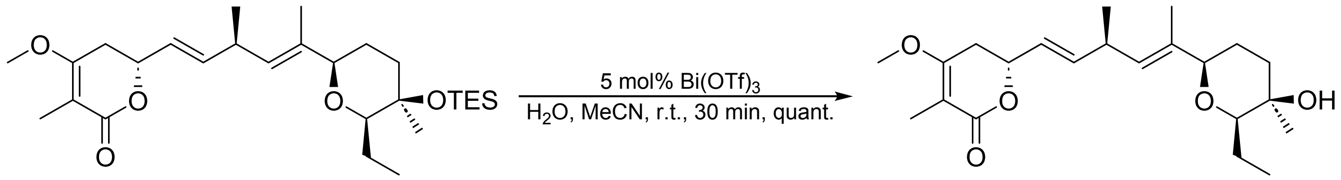

A 10 mL pear shaped flask is charged with alcohol **28** (20.6 mg, 41.6 µmol, 1.0 eq.) and dissolved in 1 mL MeCN. To this solution are subsequently added H_2O (23 µL, 1.3 mmol, 30 eq.) and $Bi(OTf)_3$ (1.3 mg, 2 µmol, 5 mol%). After stirring for 30 min (longer reaction times result in the decomposition of the lactone part, i.e. cleavage of the methylenolether and lactone elimination can be observed) the colorless suspension is quenched with saturated NH_4Cl solution. The phases are separated and the aqueous phase is extracted 3x with 10 mL Et_2O. The organic layers are combined and dried with $MgSO_4$. After filtration and evaporation of the solvent under reduced pressure the crude product is purified via flash chromatography (pentane:acetone 3:1) affording pure Jerangolid B **1** (15.6 mg, 41.2 µmol, quant.) as a colorless resin.

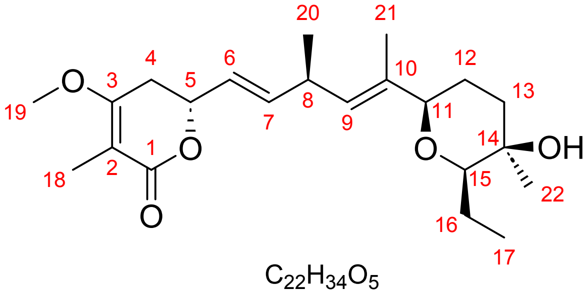

$C_{22}H_{34}O_5$
M = 378.51 g•mol^{-1}

TLC: R_f (pentane/acetone 3/1) = 0.31

^{1}H-NMR (400 MHz, MeOH-d_4, δ in ppm): 5.83 (ddd, J = 15.5 Hz, 6.4 Hz, 1.0 Hz, 1H, H7), 5.60 (ddd, J = 15.5 Hz, 6.6 Hz, 1.4 Hz, 1H, H6), 5.30 (dquint, J = 9.1 Hz, 1.3 Hz, 1H, H9), 4.80 (brddd, J = 11.2 Hz, 6.6 Hz, 4.2 Hz, 1H, H5), 3.84 (s, 3H, H19), 3.65 (brd, J = 11.1 Hz, 1H, H11), 3.22-3.14 (m, 1H, H8), 3.09 (dd, J = 9.9 Hz, 2.6 Hz, 1H, H15), 2.84 (ddq, J = 17.4 Hz, 4.2 Hz, 1.1 Hz, 1H, $H4_{eq}$), 2.63 (J = ddq, J = 17.4 Hz, 11.4 Hz, 1.9 Hz, 1H, $H4_{ax}$), 1.85-1.75 (m, 1H, H12), 1.79-1.72 (m, 1H, H13), 1.71 (dd, J = 1.9 Hz, 1.1 Hz, 3H, H18), 1.69 (d, J = 1.3 Hz, 3H, H21), 1.68-1.57 (m, 3H, H13'+H16), 1.56-1.48 (m, 1H, H12'), 1.09 (d, J = 6.9 Hz, 3H, H20), 1.09 (s, 3H, H22), 0.98 (tr, J = 7.5 Hz, 3H, H17).

^{13}C-NMR (100 MHz, MeOH-d_4, δ in ppm): 171.4 (C1), 169.4 (C3), 140.3 (C7), 137.5 (C10), 128.8 (C9), 126.4 (C6), 102.7 (C2), 86.9 (C15), 83.5 (C11), 77.0 (C5), 69.3 (C14), 56.3 (C19), 39.2 (C13), 35.9 (C8), 30.3 (C4), 27.4 (C12), 25.9 (C22), 22.7 (C16), 21.0 (C20), 13.6 (C21), 11.4 (C17), 8.8 (C18).

HRMS: calculated for $C_{22}H_{33}O_5$ [M-H]$^-$: 377.2334, measured for $C_{22}H_{33}O_5$ [M-H]$^-$: 377.2338.

Table S2. Comparison of the ^{1}H-NMR spectra with natural Jerangolid B **1**.

H	Natural[7] ^{1}H-NMR (400 MHz, MeOH-d_4)	Synthetic ^{1}H-NMR (400 MHz, MeOH-d_4)
$H4_a$	2.88 (dd, 17.4, 4.2)	2.84 (ddq, 17.4, 4.2, 1.1)
$H4_b$	2.67 (m)	2.63 (ddq, 17.4, 11.4, 1.9)
H5	4.86 (m)	4.80 (brddd, 11.2, 6.6, 4.2)
H6	5.63 (ddd, 15.5, 6.6, 1.2)	5.60 (ddd, 15.5, 6.6, 1.4)
H7	5.87 (dd, 15.6, 6.2)	5.83 (ddd, 15.5, 6.4, 1.0)
H8	3.22 (m)	3.22-3.14 (m)
H9	5.34 (dd, 9.1, 1.2, br.)	5.30 (dquint, 9.0, 1.3)
H11	3.69 (d, 10.7, br.)	3.65 (brd, 11.1)
$H12_a$	1.66 (m)	1.68-1.57 (m)
$H12_b$	1.62 (m)	1.56-1.48 (m)
$H13_a$	1.83 (m)	1.79-1.72 (m)
$H13_b$	1.69 (m)	1.68-1.57 (m)
H15	3.13 (dd, 9.9, 2.7)	3.09 (dd, 9.9, 2.6)
$H16_a$	1.87 (m)	1.68-1.57 (m)
$H16_b$	1.59 (m)	1.68-1.57 (m)
H17	1.02 (dd, 7.4, 7.4)	0.98 (tr, 7.4)
H18	1.74 (s)	1.71 (dd, 1.9, 1.1)
H19	3.88 (s)	3.84 (s)
H20	1.13 (d, 6.8)	1.09 (d, 6.9)
H21	1.73 (d, 1.2)	1.67 (d, 1.3)
H22	1.13 (s)	1.09 (s)

Table S3. Comparison of the ^{13}C-NMR spectra with natural Jerangolid B **1**.

**C**	Natural[7] ^{13}C-NMR (100 MHz, MeOH-d_4)	Synthetic ^{13}C-NMR (100 MHz, MeOH-d_4)	Δδ (ppm)
C1	171.4	171.4	0.0
C2	102.7	102.7	0.0
C3	169.3	169.4	+0.1
C4	30.3	30.3	0.0
C5	76.9	77.0	+0.1
C6	126.4	126.4	0.0
C7	140.3	140.3	0.0
C8	35.8	35.9	+0.1
C9	128.9	129.8	-0.1
C10	137.5	137.5	0.0
C11	83.5	83.5	0.0
C12	22.7	27.4	+4.7
C13	39.2	39.2	0.0
C14	69.3	69.3	0.0
C15	86.9	86.9	0.0
C16	27.4	22.7	-4.7
C17	11.4	11.4	0.0
C18	8.8	8.8	0.0
C19	56.3	56.3	0.0
C20	21.0	21.0	0.0
C21	13.6	13.6	0.0
C22	25.8	25.9	+0.1

All signals were unambiguously assigned through HMBC NMR experiments. The signals of C12 and C16 appear to have been interchanged in the original report by Höfle *et al.* As a result, both signals deviate by the same magnitude but in opposite directions: -4.7 ppm for C16 and +4.7 ppm for C12.

14-S-Jerangolid B S14

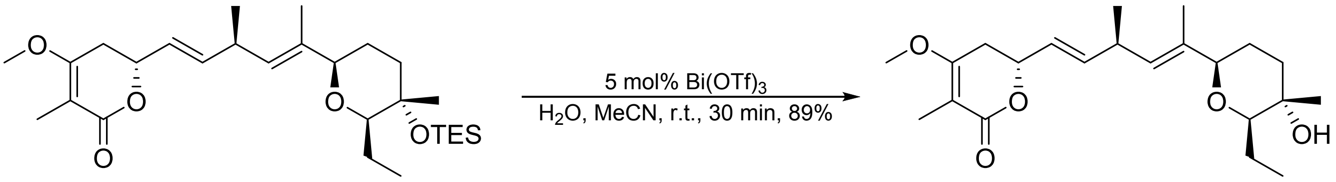

A 10 mL pear shaped flask is charged with alcohol **S14** (28.2 mg, 57.0 µmol, 1.0 eq.) and dissolved in 1.2 mL MeCN. To this solution are subsequently added H_2O (32 µL, 1.8 mmol, 30 eq.) and $Bi(OTf)_3$ (1.9 mg, 2.9 µmol, 5 mol%). After stirring for 30 min the colorless suspension is quenched with saturated NH_4Cl solution. The phases are separated and the aqueous phase is extracted 3x with 10 mL Et_2O. The organic layers are combined and dried with $MgSO_4$. After filtration and evaporation of the solvent under reduced pressure the crude product is purified via flash chromatography (pentane:acetone 3:1) affording pure Jerangolid B **1** (15.6 mg, 41.2 µmol, quant.) as a colorless resin.

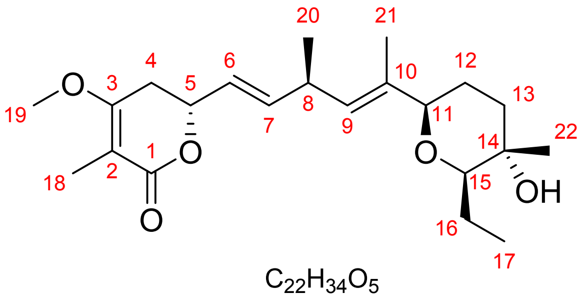

$C_{22}H_{34}O_5$
M = 378.51 g•mol^{-1}

TLC: R_f (pentane/acetone 3/1) = 0.22
$[\alpha]_{20}^{D}$ = +89.9 (c = 0.99, CH_2Cl_2)
^{1}H-NMR (400 MHz, MeOH-d$_4$, δ in ppm): 5.83 (ddd, J = 15.5 Hz, 6.5 Hz, 1.0 Hz, 1H, H7), 5.59 (ddd, J = 15.5 Hz, 6.6 Hz, 1.3 Hz, 1H, H6), 5.27 (dquint, J = 9.0 Hz, 1.2 Hz, H9), 4.81 (brddd, J = 11.2 Hz, 6.6 Hz, 4.1 Hz, 1H, H5), 3.84 (s, 3H, H19), 3.68 (brd, J = 10.6 Hz, 1H, H11), 3.23-3.12 (m, 1H, H8), 3.03 (dd, J = 10.2 Hz, 1.8 Hz, 1H, H15), 2.85 (ddq, J = 17.4 Hz, 4.2 Hz, 1.1 Hz, 1H, $H4_a$), 2.63 (ddq, J = 17.4 Hz, 11.3 Hz, 1.9 Hz, 1H, $H4_b$), 1.83-1.78 (m, 1H, $H13_a$), 1.78-1.72 (m, 1H, $H16_a$), 1.71 (dd, J = 1.9 Hz, 1.1 Hz, 3H, H18), 1.67 (d, J = 1.3 Hz, 3H, H21), 1.68-1-58 (m, 3H, $H13_b$+$H12_a$+$H12_b$), 1.34-1.22 (m, 1H, $H16_b$), 1.13 (s, 3H, H22), 1.09 (d, J = 6.9 Hz, 3H, H20), 0.97 (tr, J = 7.4 Hz, 3H, H17).
^{13}C-NMR (100 MHz, MeOH-d$_4$, δ in ppm): 171.4 (C1), 169.4 (C3), 140.2 (C7), 137.2 (C10), 129.3 (C9), 126.4 (C6), 102.7 (C2), 87.6 (C15), 83.9 (C11), 76.9 (C5), 70.2 (C14), 56.3 (C19), 40.9 (C13), 35.9 (C8), 30.3 (C12), 30.3 (C4), 22.8 (C16), 21.0 (C20), 20.0 (C22), 13.3 (C21), 11.8 (C17), 8.8 (C18).
HRMS: calculated for $C_{22}H_{33}O_5$ [M-H]$^-$: 377.2334, measured for $C_{22}H_{33}O_5$ [M-H]$^-$: 377.2338.

Table S4. Comparison of the ^{1}H-NMR spectra with synthetic 14-S-Jerangolid **S14**.

H	Natural[7]<br><br>^{1}H-NMR (400 MHz, MeOH-d_4)	Synthetic<br>14-S-Jerangolid **S14**<br><br>^{1}H-NMR (400 MHz, MeOH-d_4)
$H4_a$	2.88 (dd, 17.4, 4.2)	2.85 (ddq, 17.4, 4.2, 1.1)
$H4_b$	2.67 (m)	2.63 (ddq, 17.4, 11.3, 1.9)
H5	4.86 (m)	4.81 (brddd, 11.2, 6.6, 4.1)
H6	5.63 (ddd, 15.5, 6.6, 1.2)	5.59 (ddd, 15.5, 6.6, 1.3)
H7	5.87 (dd, 15.6, 6.2)	5.83 (ddd, 15.5, 6.5, 1.0)
H8	3.22 (m)	3.23-3.12 (m)
H9	5.34 (dd, 9.1, 1.2, br.)	5.27 (dquint, 9.0, 1.2)
H11	3.69 (d, 10.7, br.)	3.68 (brd, 10.6)
$H12_a$	1.66 (m)	1.68-1.58 (m)
$H12_b$	1.62 (m)	1.68-1.58 (m)
$H13_a$	1.83 (m)	1.83-1.78 (m)
$H13_b$	1.69 (m)	1.68-1.58 (m)
H15	3.13 (dd, 9.9, 2.7)	3.03 (dd, 10.2, 1.8)
$H16_a$	1.87 (m)	1.78-1.72 (m)
$H16_b$	1.59 (m)	1.34-1.22 (m)
H17	1.02 (dd, 7.4, 7.4)	0.97 (tr, 7.4)
H18	1.74 (s)	1.71 (dd, 1.9, 1.1)
H19	3.88 (s)	3.84 (s)
H20	1.13 (d, 6.8)	1.09 (d, 6.9)
H21	1.73 (d, 1.2)	1.67 (d, 1.3)
H22	1.13 (s)	1.13 (s)

Table S5. Comparison of the ^{13}C-NMR spectra with synthetic 14-S-Jerangolid **SX**.

C	Natural[7]<br>^{13}C-NMR (100 MHz, MeOH-d_4)	Synthetic<br>14-S Jerangolid **S14**<br>^{13}C-NMR (100 MHz, MeOH-d_4)	Δδ (ppm)
C1	171.4	171.4	0.0
C2	102.7	102.7	0.0
C3	169.3	169.4	+0.1
C4	30.3	30.3	0.0
C5	76.9	76.9	0.0
C6	126.4	126.4	0.0
C7	140.3	140.2	-0.1
C8	35.8	35.9	+0.1
C9	128.9	129.3	+0.4
C10	137.5	137.2	-0.3
C11	83.5	83.9	+0.4
C12	22.7	30.3	+7.6
C13	39.2	40.9	+1.7
C14	69.3	70.2	+0.9
C15	86.9	87.6	+0.7
C16	27.4	22.8	-4.6
C17	11.4	11.8	+0.4
C18	8.8	8.8	0.0
C19	56.3	56.3	0.0
C20	21.0	21.0	0.0
C21	13.6	13.3	-0.3
C22	25.8	20.0	-5.8

NMR spectra

Methyl (S)-2-((*tert*-butyldimethylsilyl)oxy)propanoate 11

^{1}H-NMR (400 MHz, $CDCl_3$)

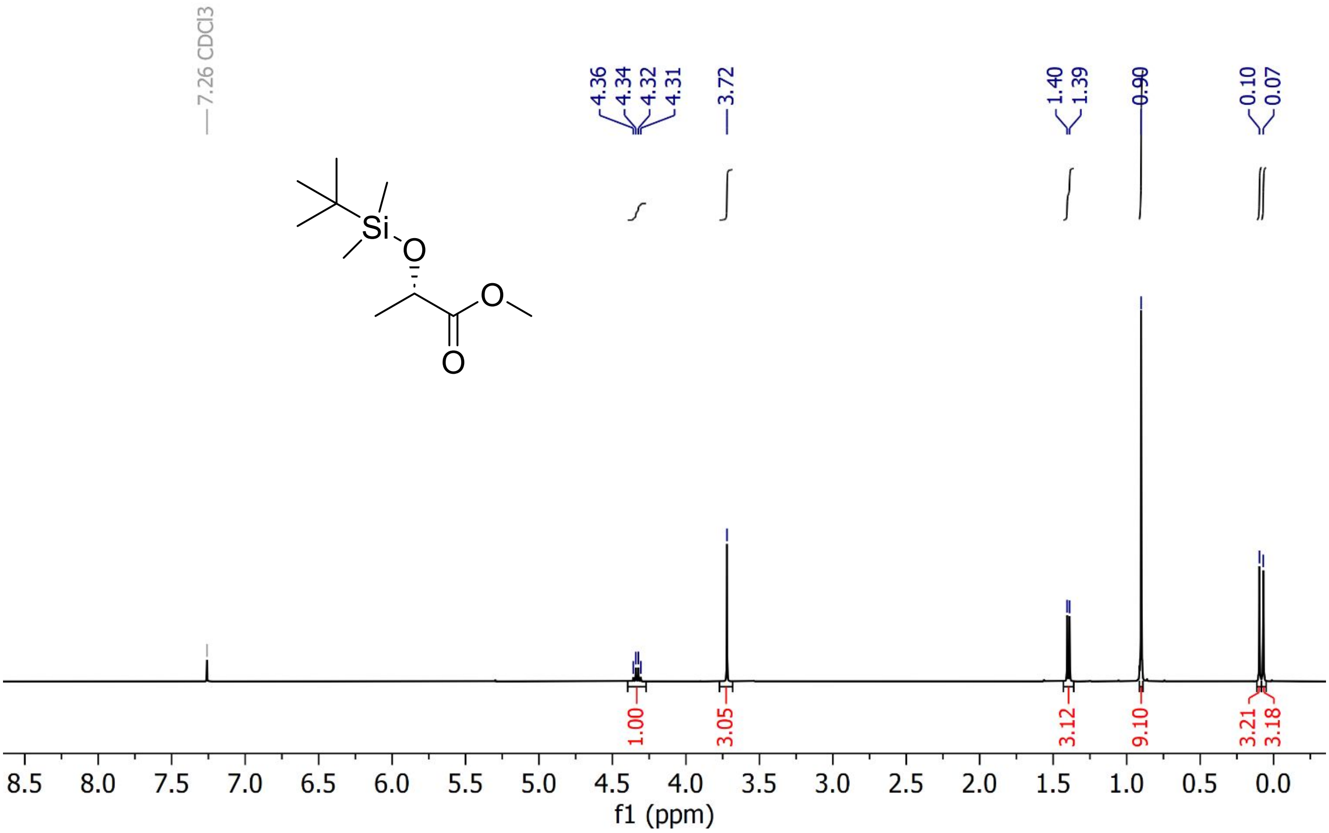

^{13}C-NMR (100 MHz, $CDCl_3$

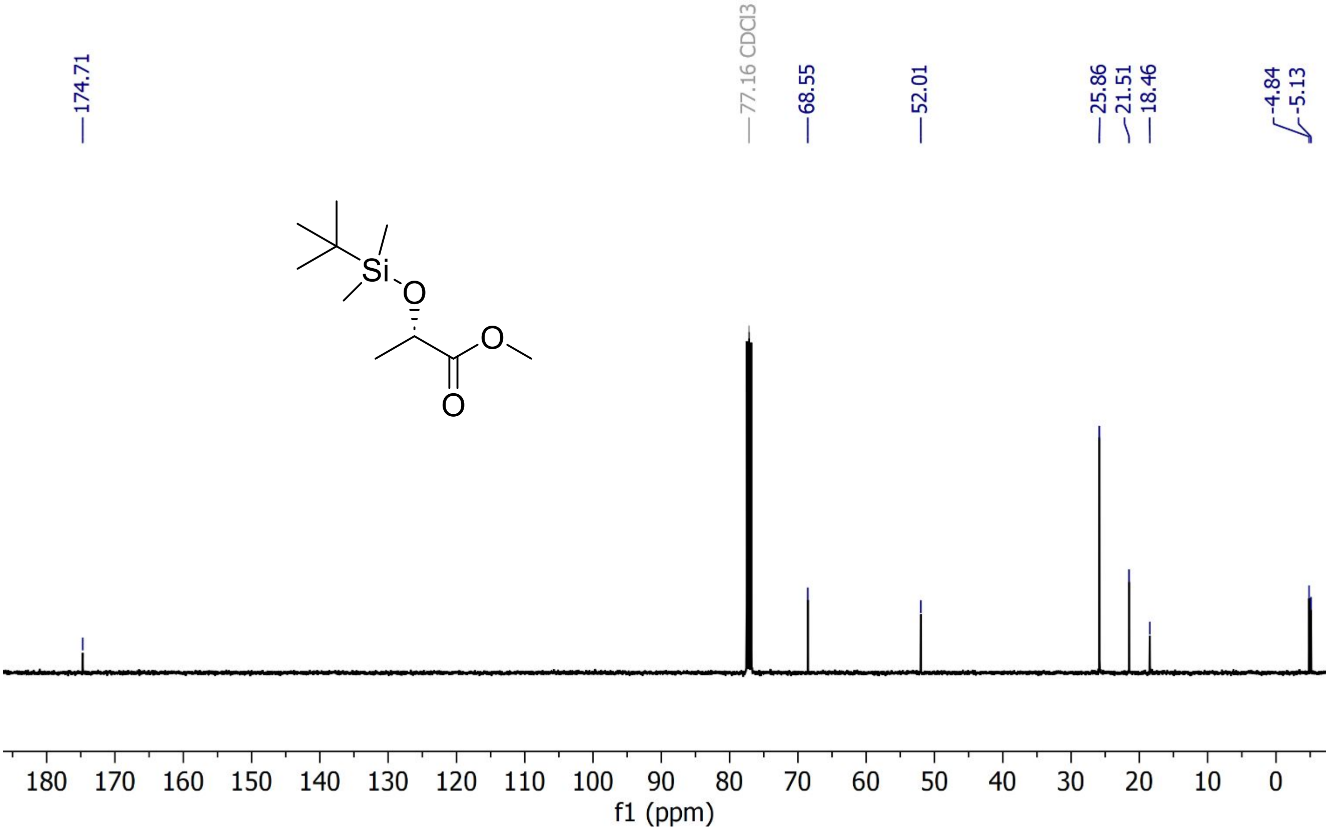

Methyl (S)-4-((*tert*-butyldimethylsilyl)oxy)pent-2-enoate 12

^{1}H-NMR (400 MHz, $CDCl_3$)

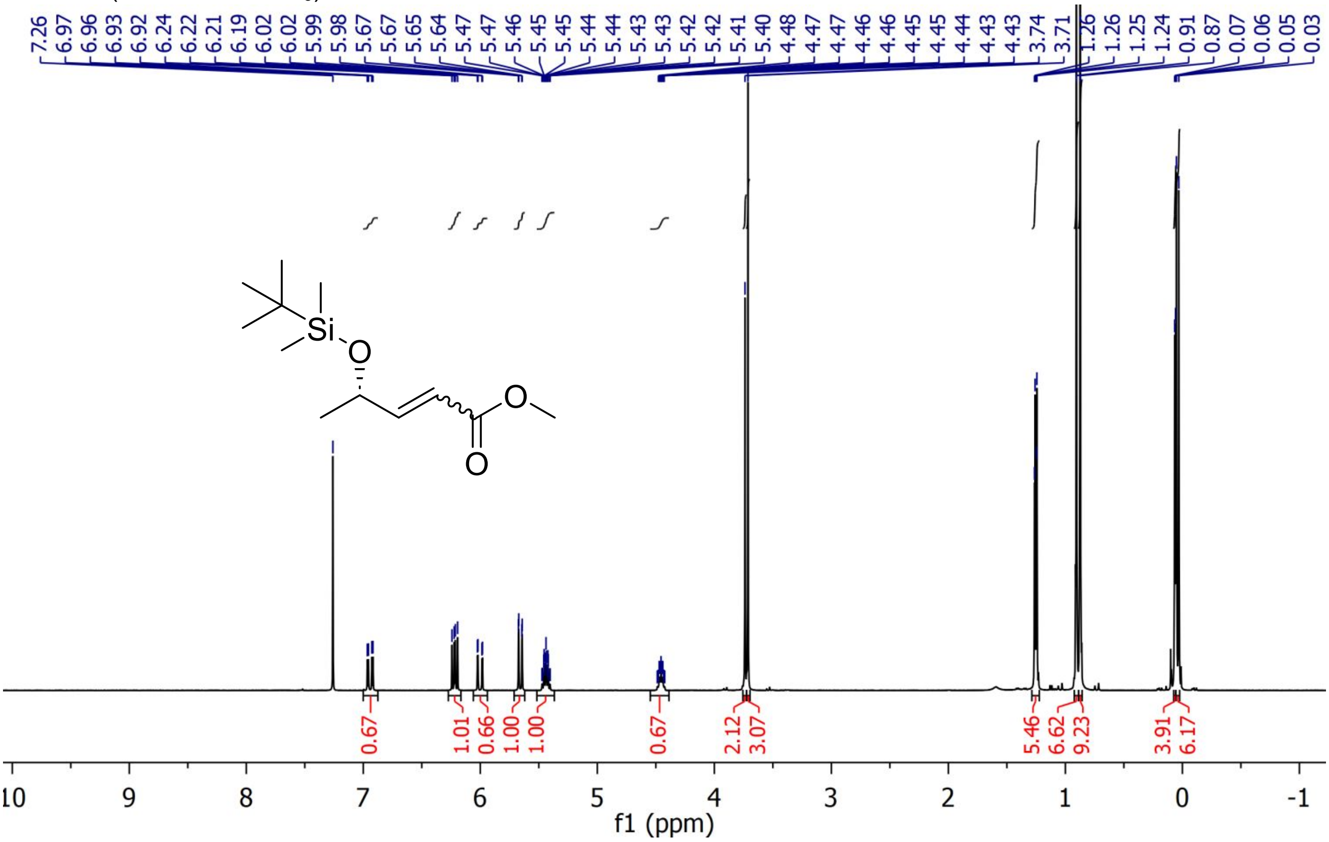

^{13}C-NMR (100 MHz, $CDCl_3$)

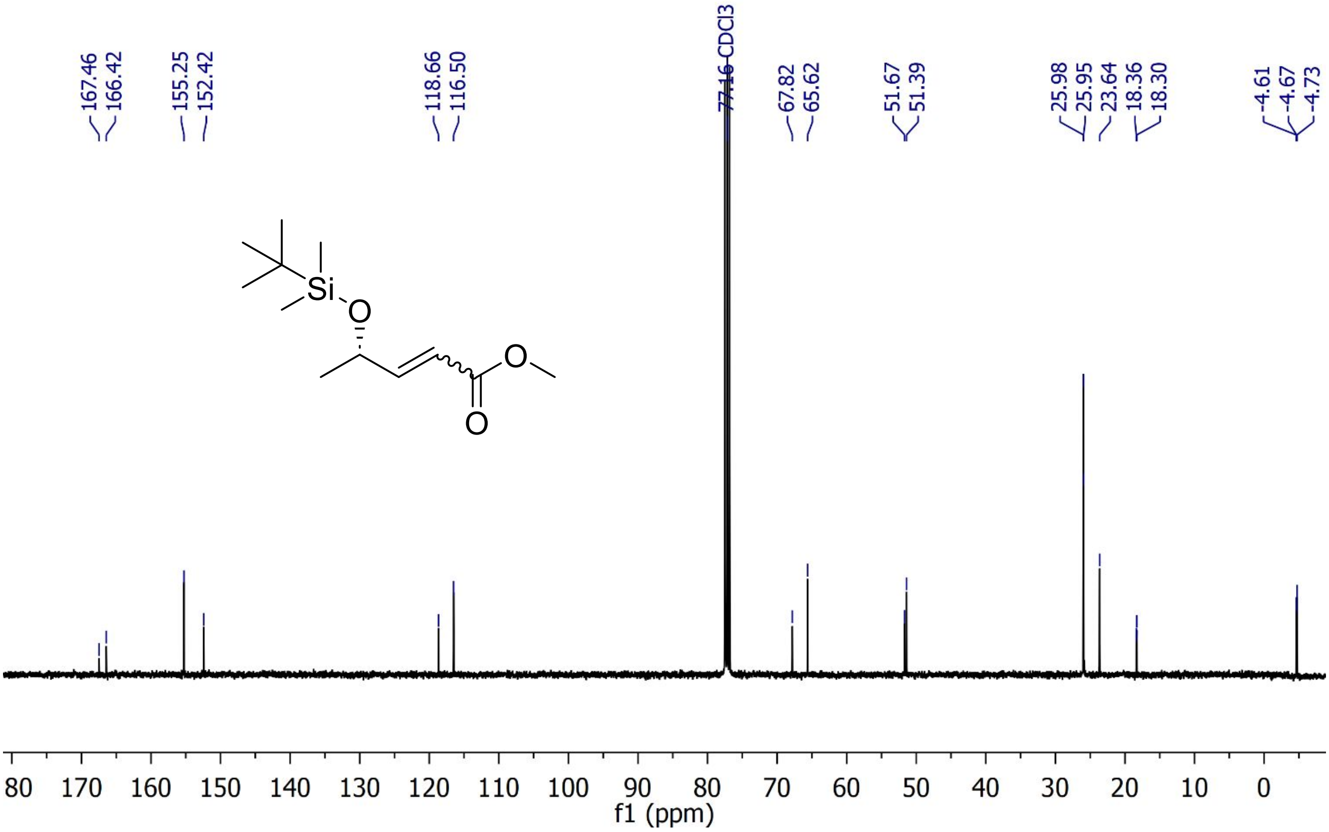

(S)-4-((*tert*-butyldimethylsilyl)oxy)pent-2-en-1-ol 13

^{1}H-NMR (400 MHz, $CDCl_3$)

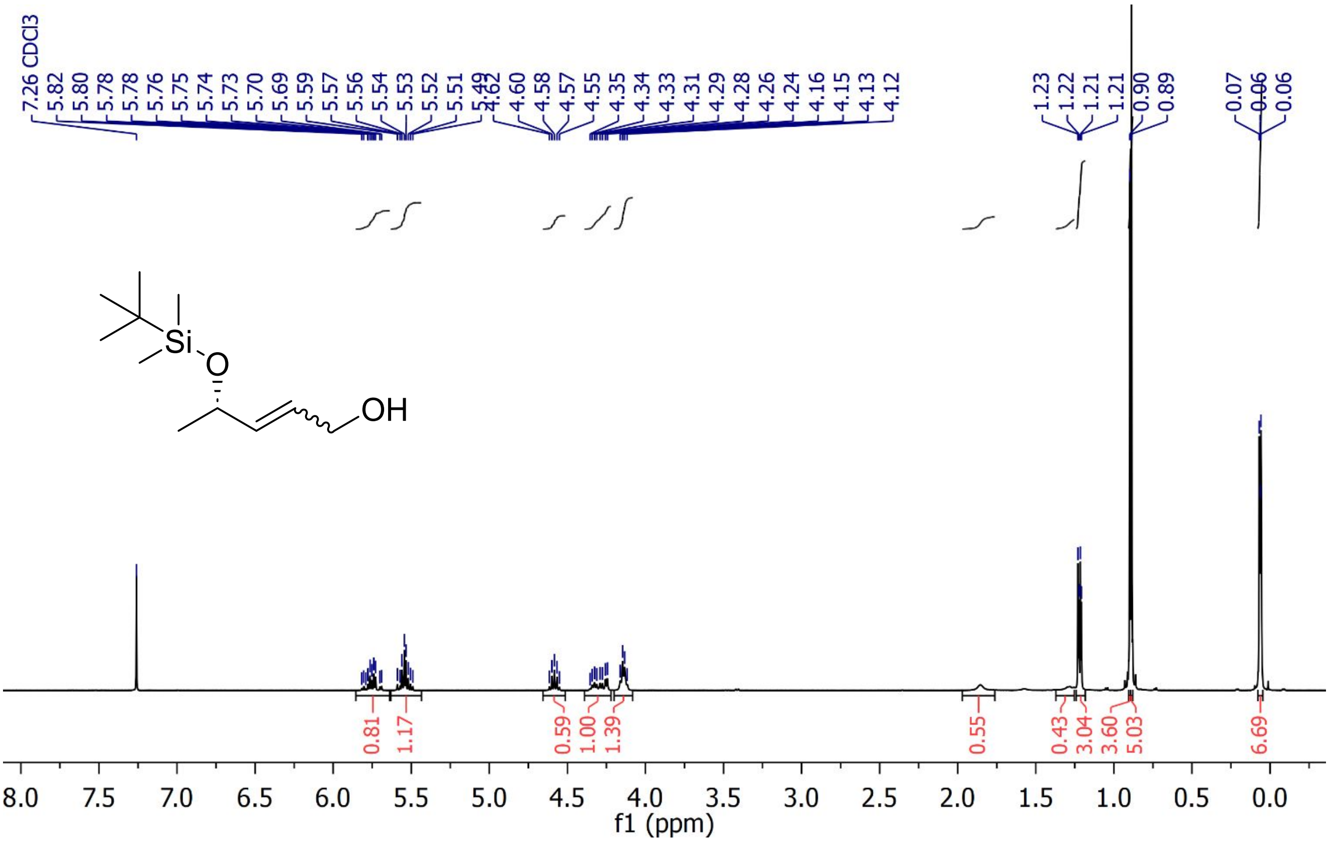

^{13}C-NMR (100 MHz, $CDCl_3$)

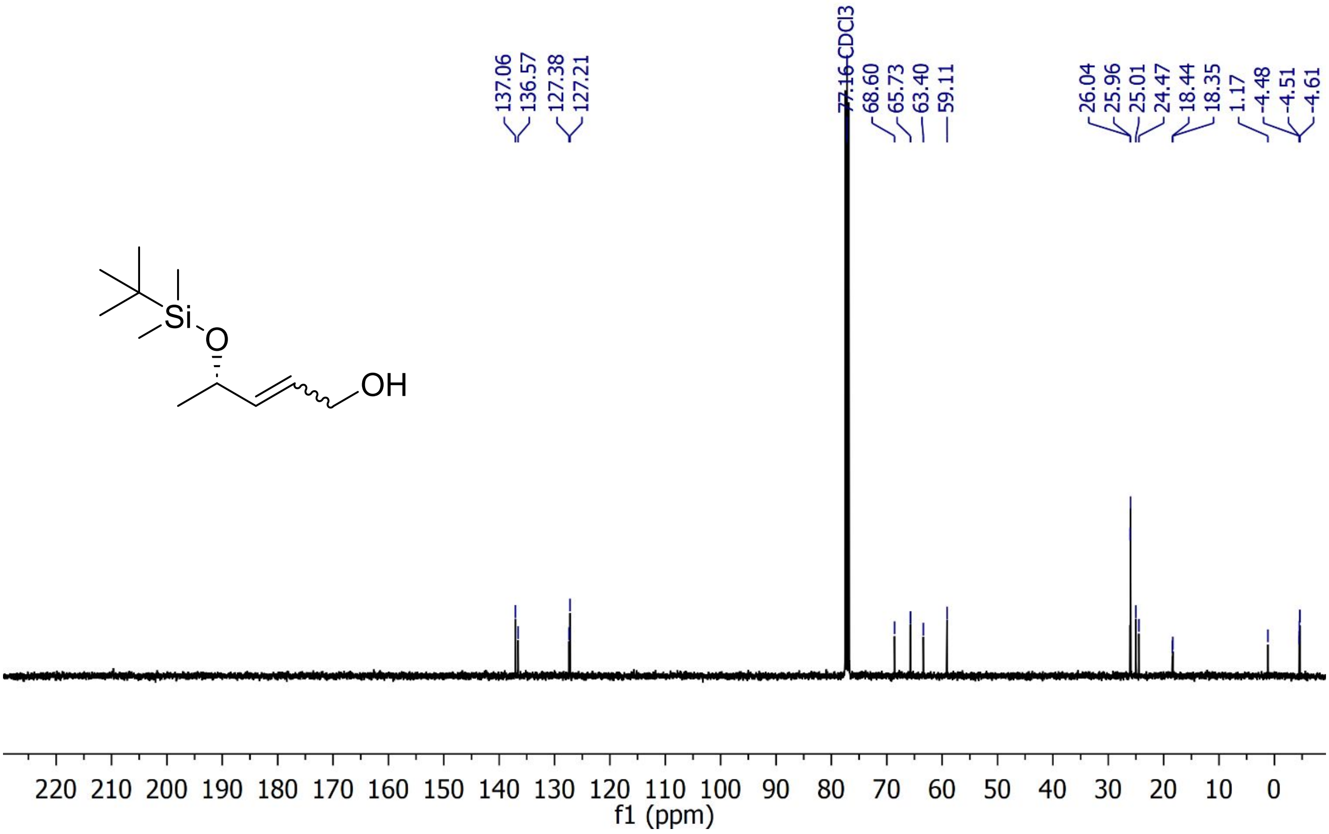

(S,E)-4-((*tert*-butyldimethylsilyl)oxy)pent-2-enal 14

^{1}H-NMR (400 MHz, $CDCl_3$)

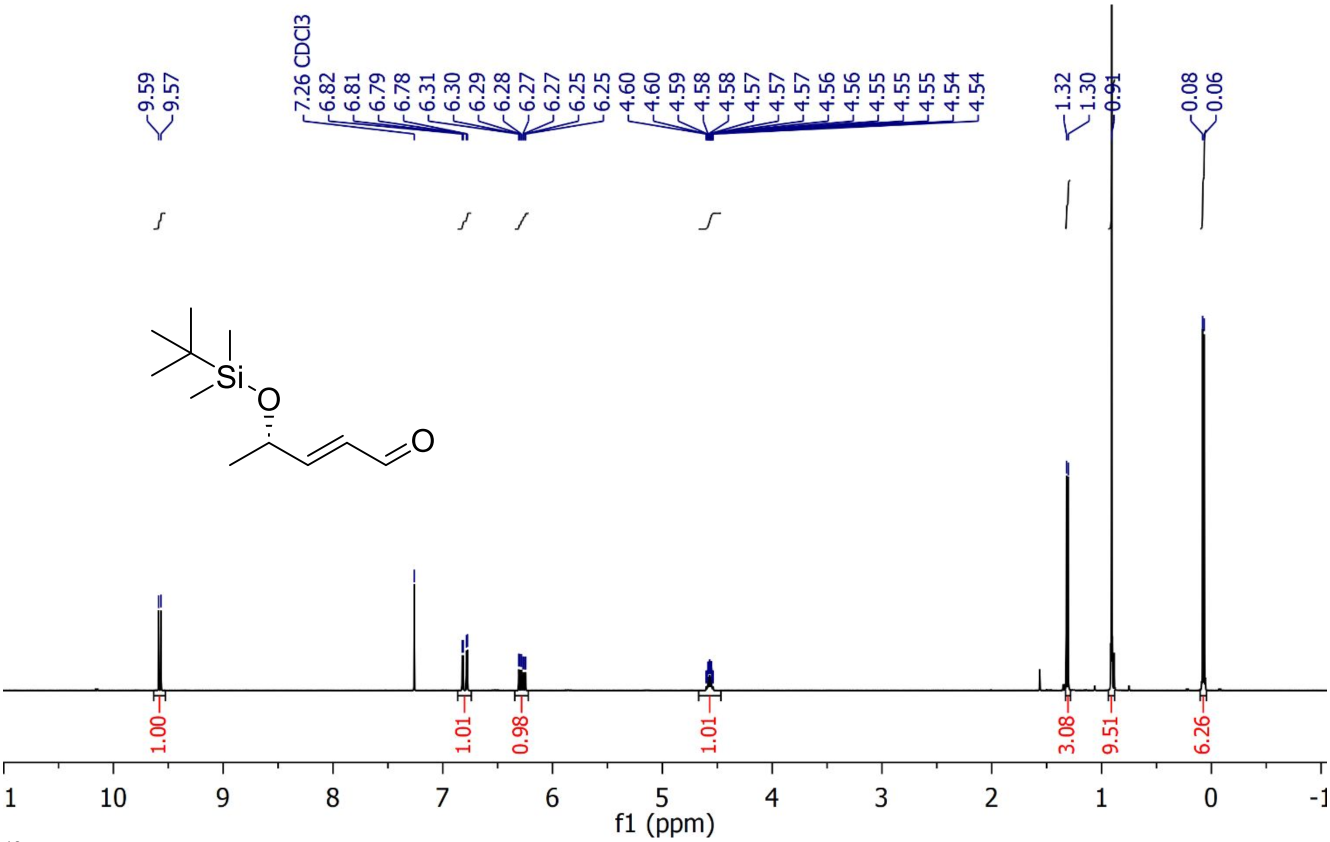

^{13}C-NMR (100 MHz, $CDCl_3$)

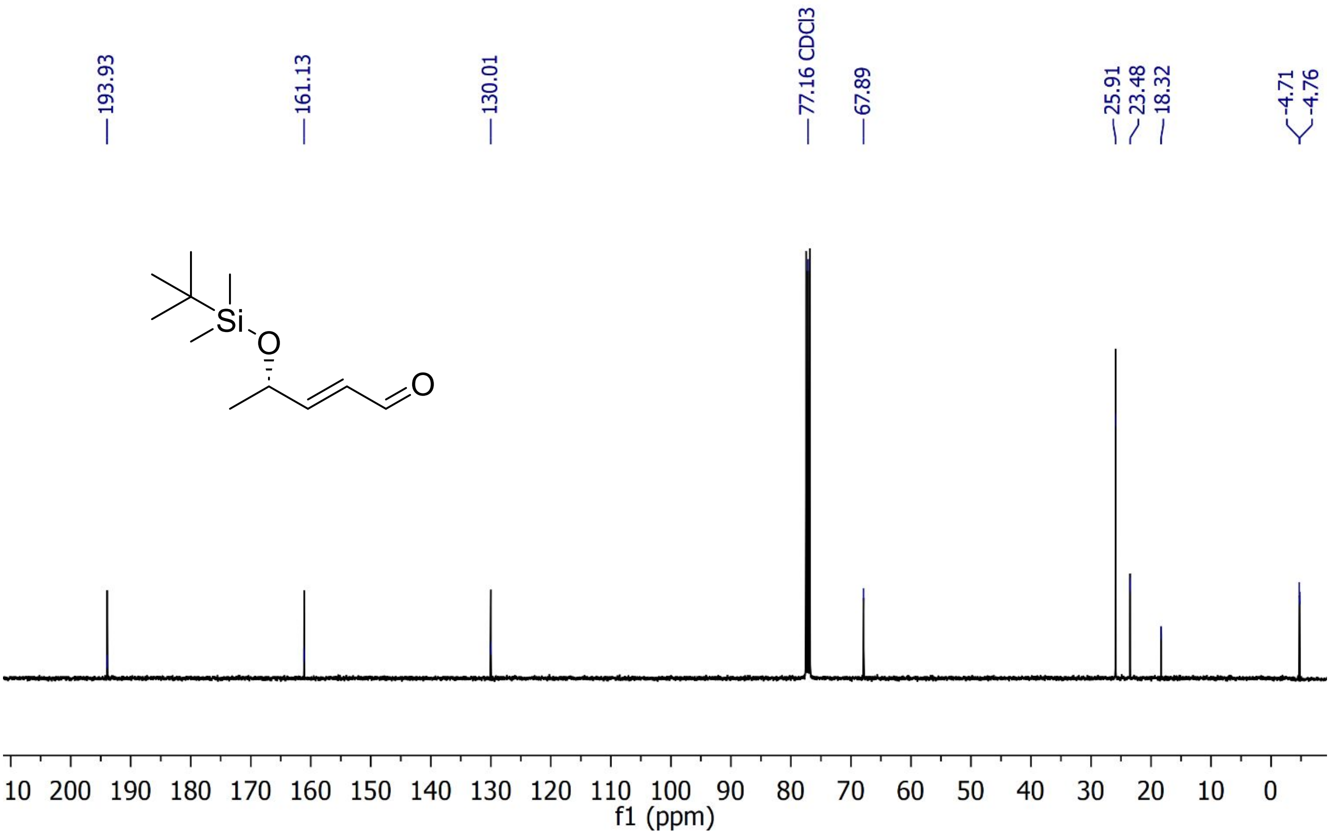

***tert*-butyl 2-methyl-3-oxobutanoate S1**

^{1}H-NMR (400 MHz, $CDCl_3$)

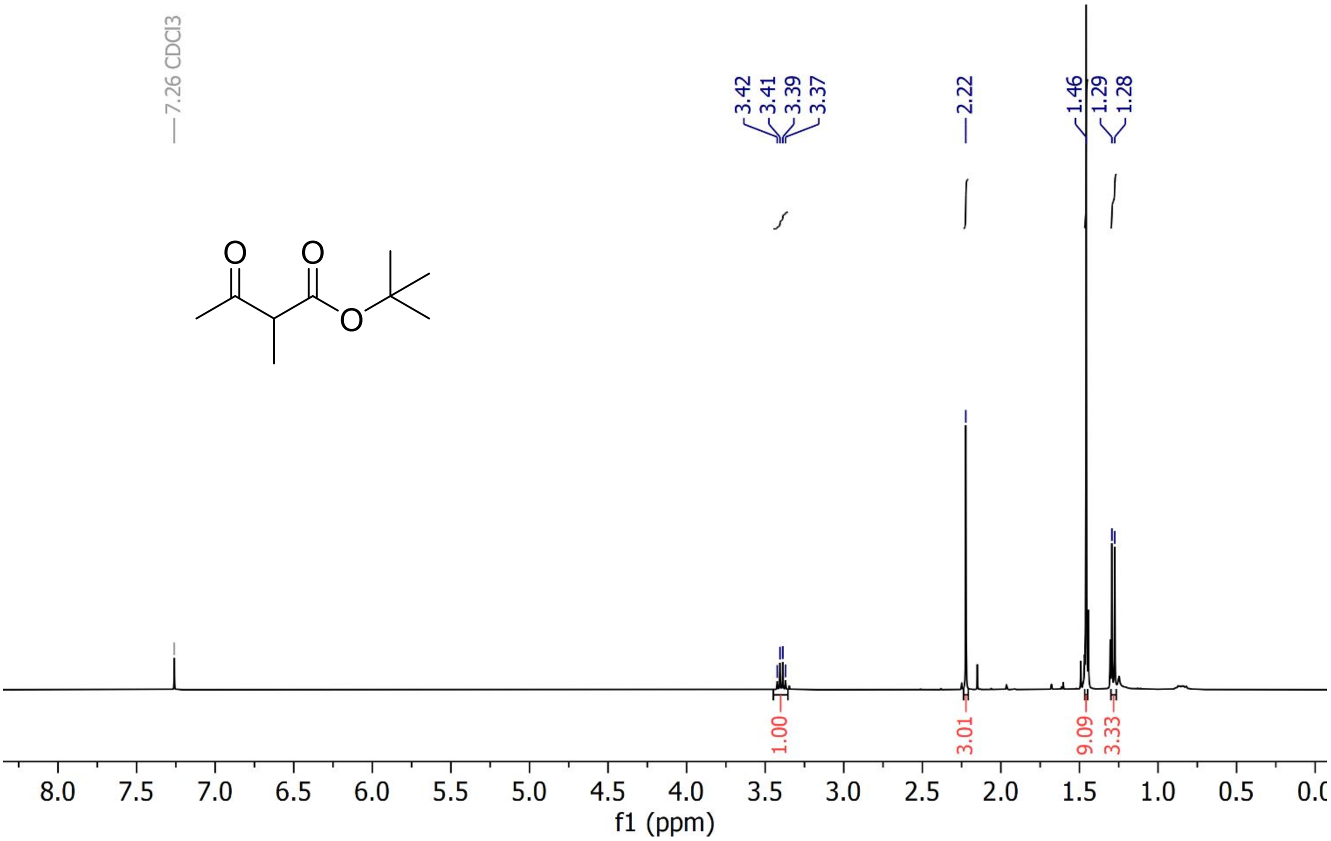

^{13}C-NMR (100 MHz, $CDCl_3$)

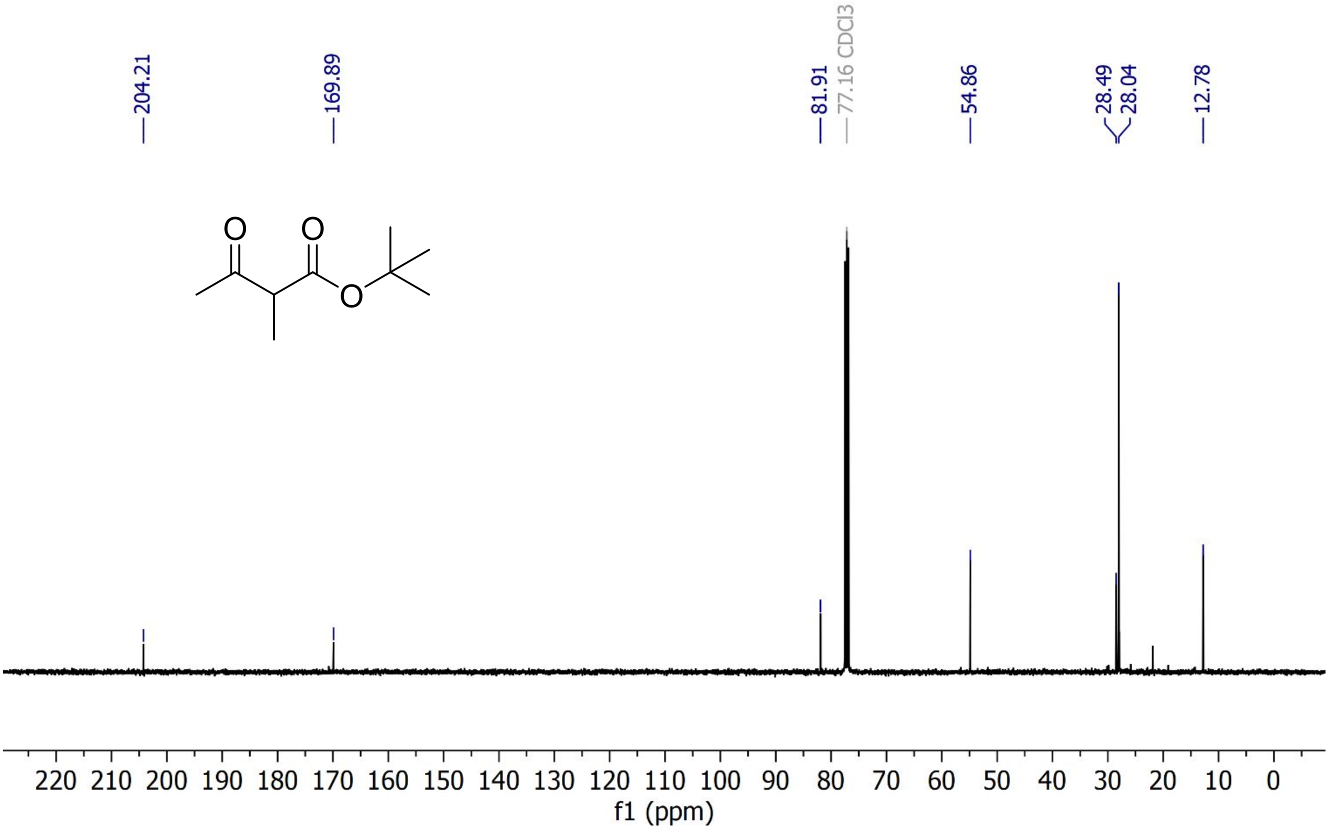

2,2,5,6-tetramethyl-4H-1,3-dioxin-4-one S2

^{1}H-NMR (400 MHz, $CDCl_3$)

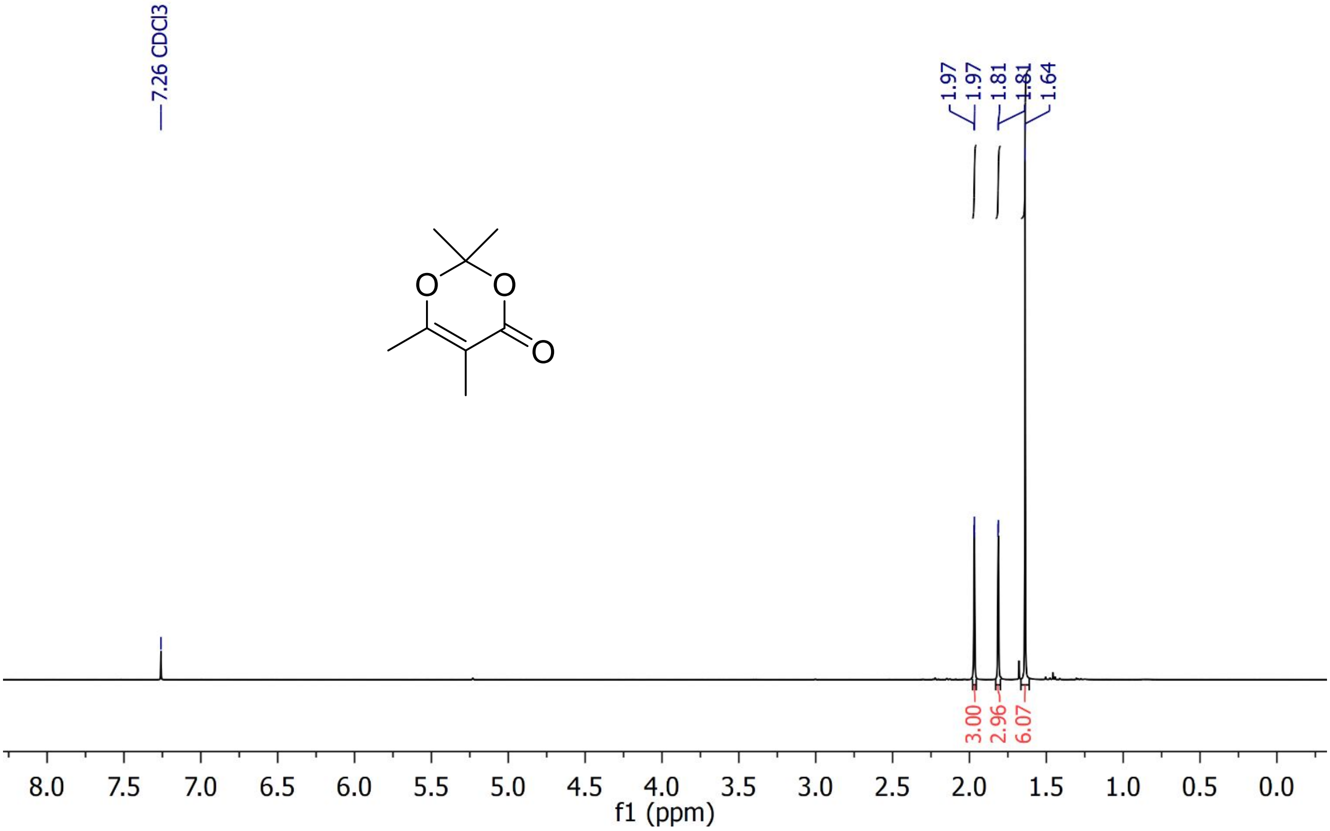

^{13}C-NMR (100 MHz, $CDCl_3$)

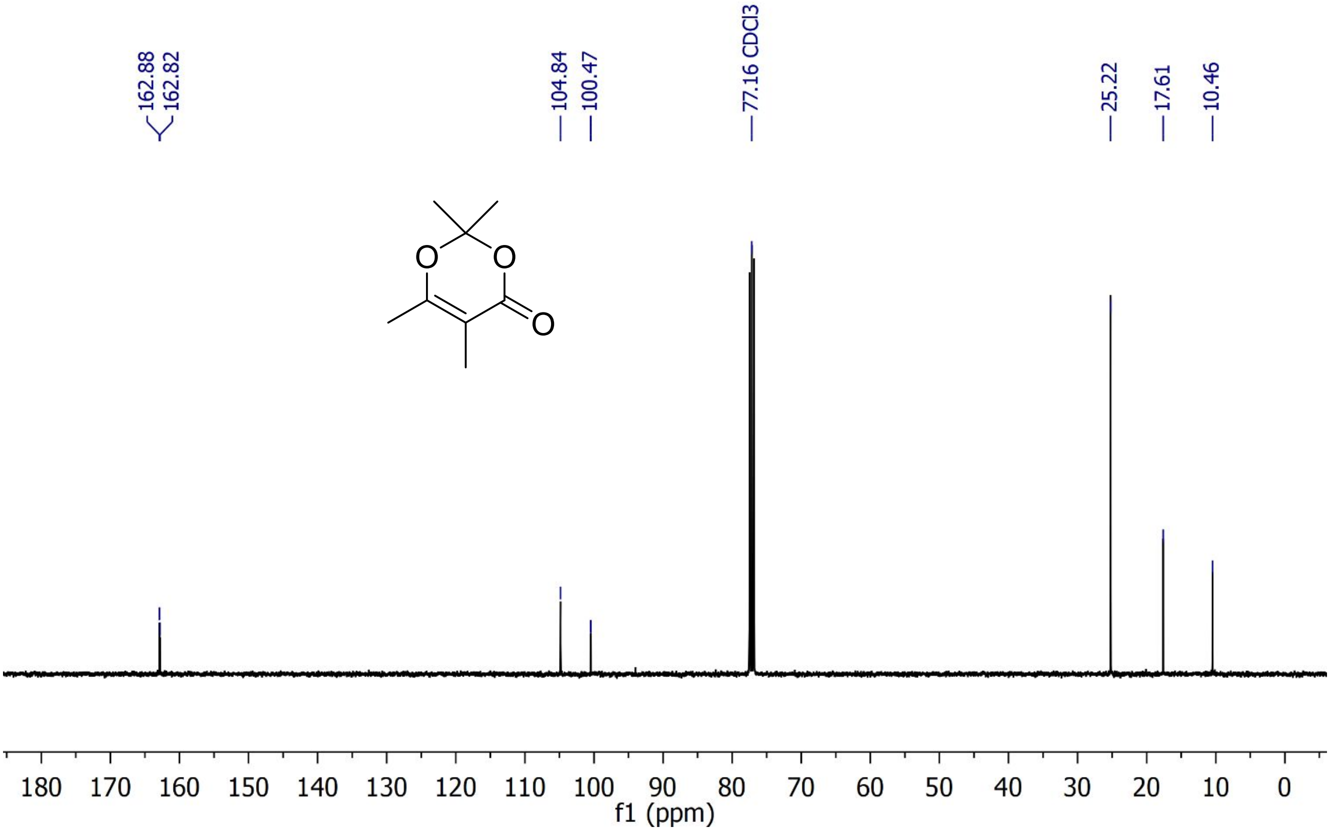

Trimethyl((2,2,5-trimethyl-4-methylen-4H-1,3-dioxin-6-yl)oxy)silane 15

^{1}H-NMR (400 MHz, $CDCl_3$)

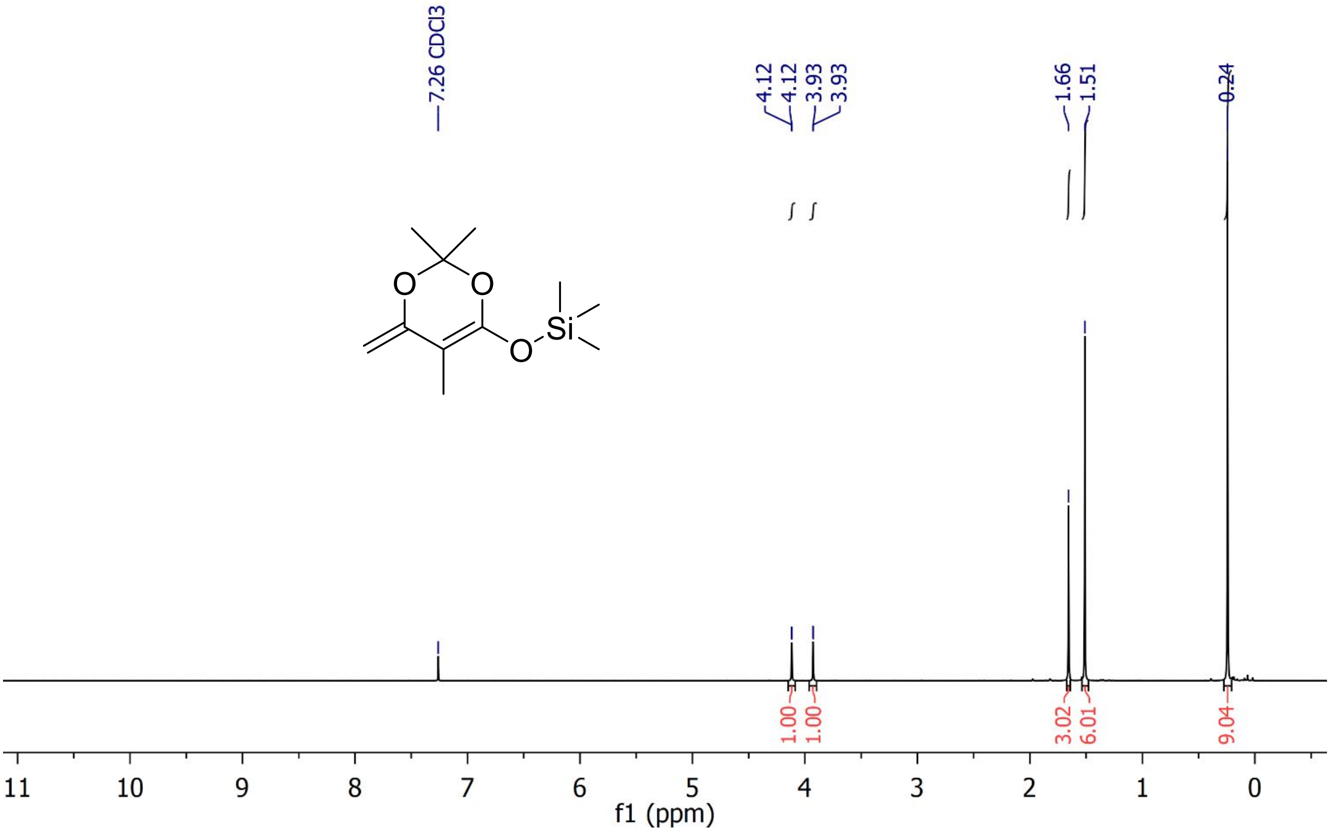

^{13}C-NMR (100 MHz, $CDCl_3$)

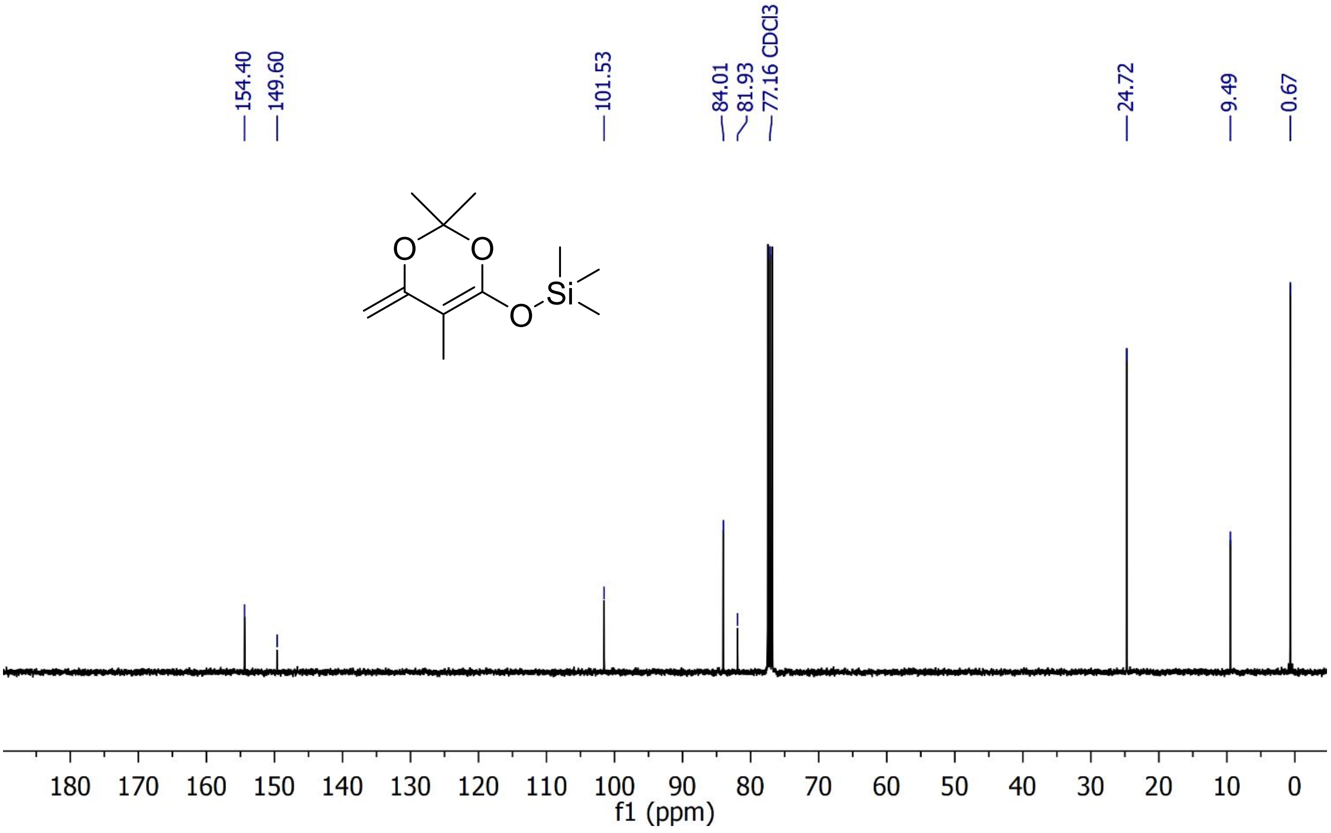

6-((2R,5S,E)-5-((*tert*-Butyldimethylsilyl)oxy)-2-hydroxyhex-3-en-1-yl)-2,2,5-trimethyl-4H-1,3-dioxin-4-one 16

^{1}H-NMR (400 MHz, $CDCl_3$)

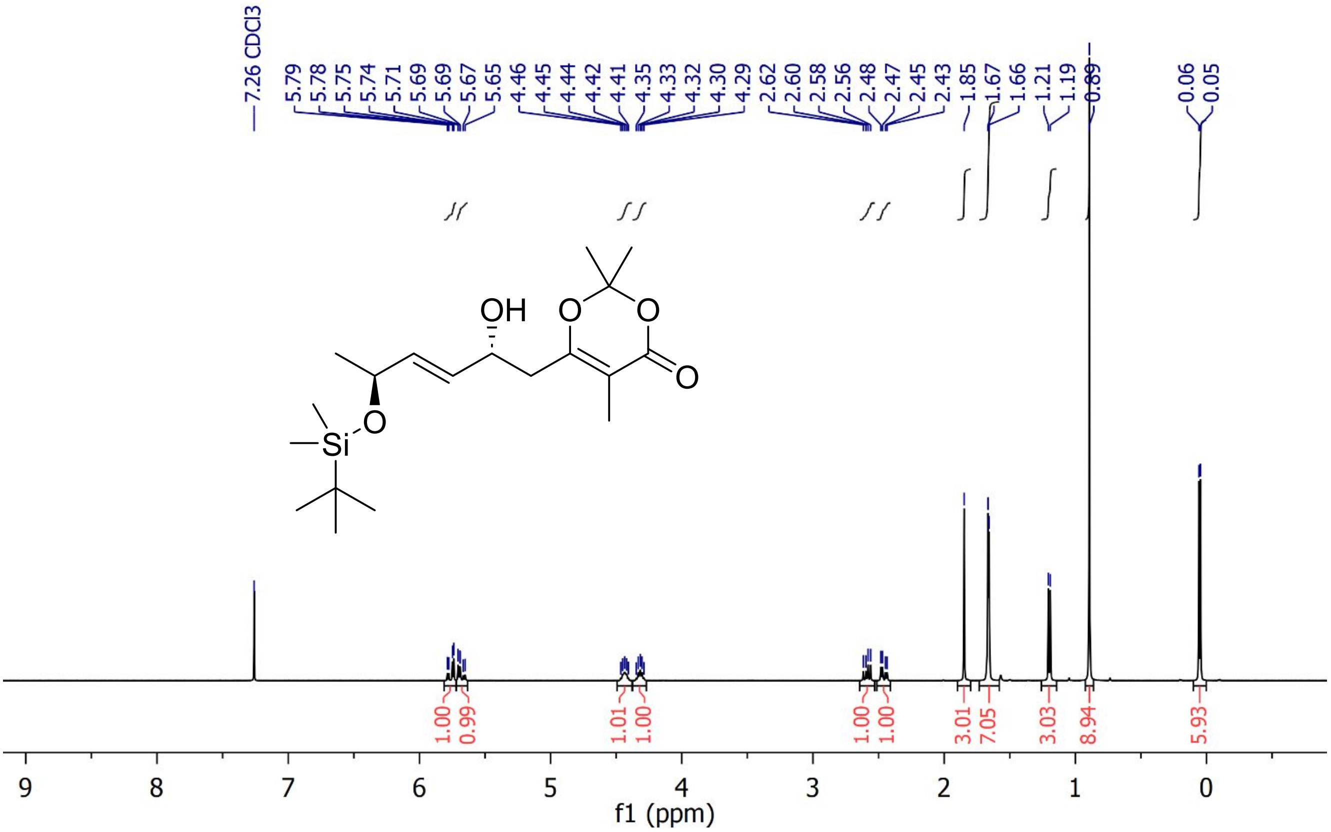

^{13}C-NMR (100 MHz, $CDCl_3$)

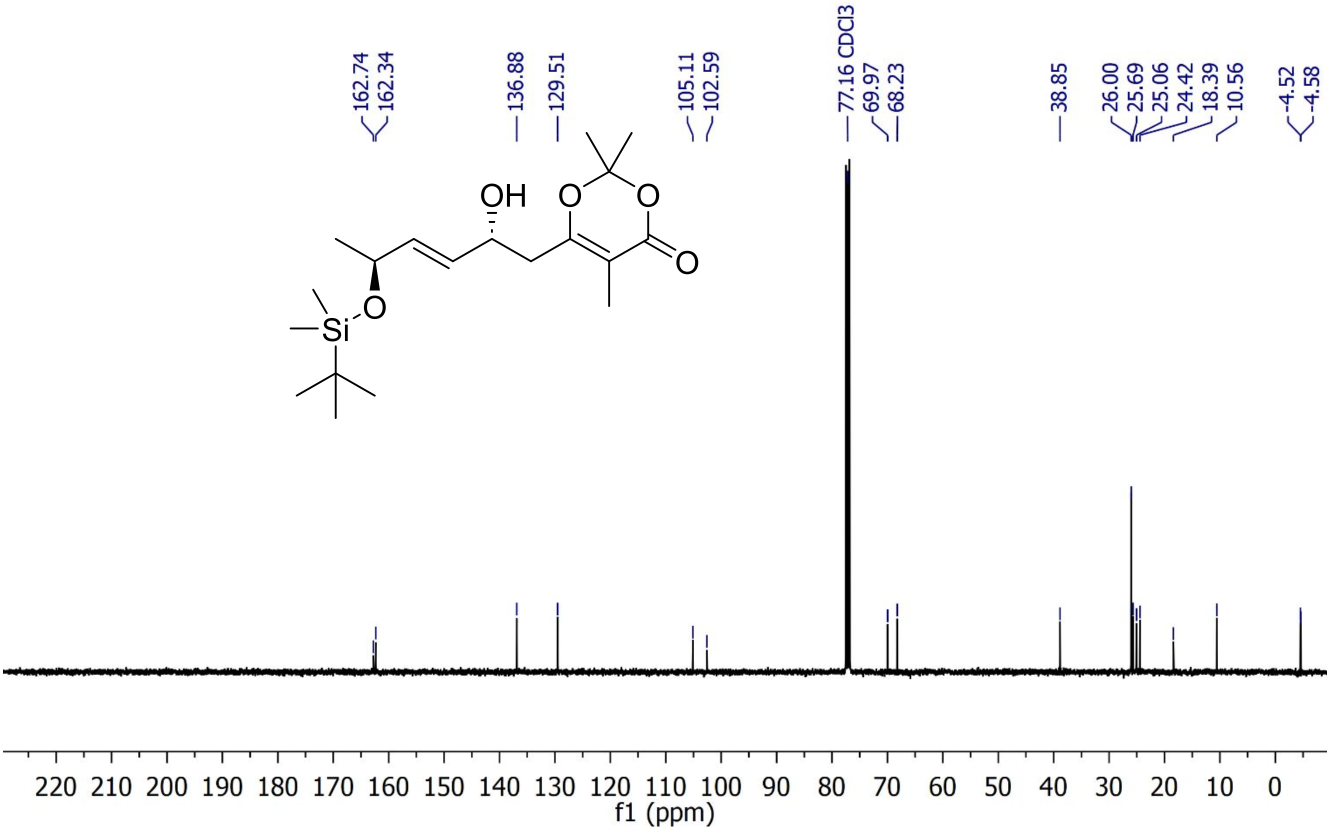

(S) Mosher ester S3

^{1}H-NMR (400 MHz, $CDCl_3$)

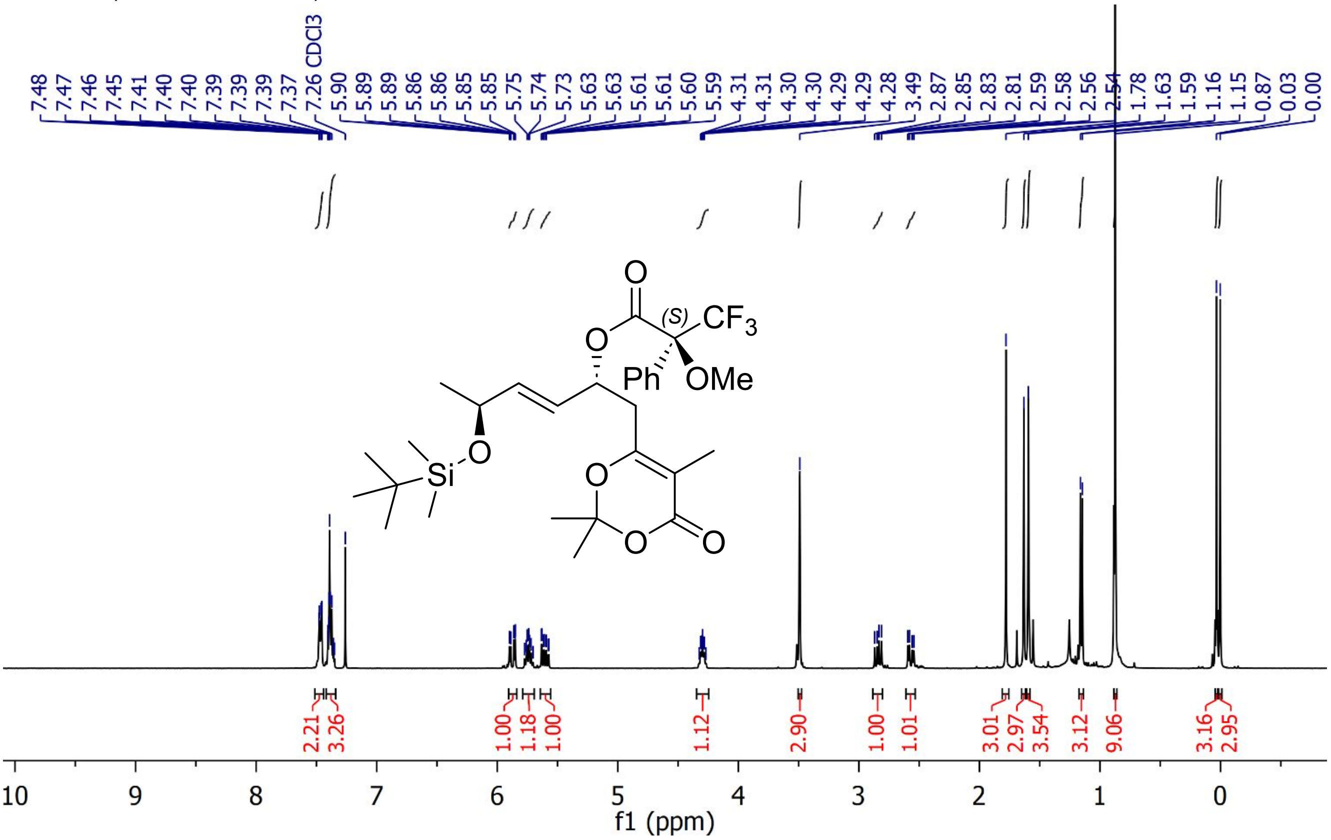

(R) Mosher ester S4

^{1}H-NMR (400 MHz, $CDCl_3$)

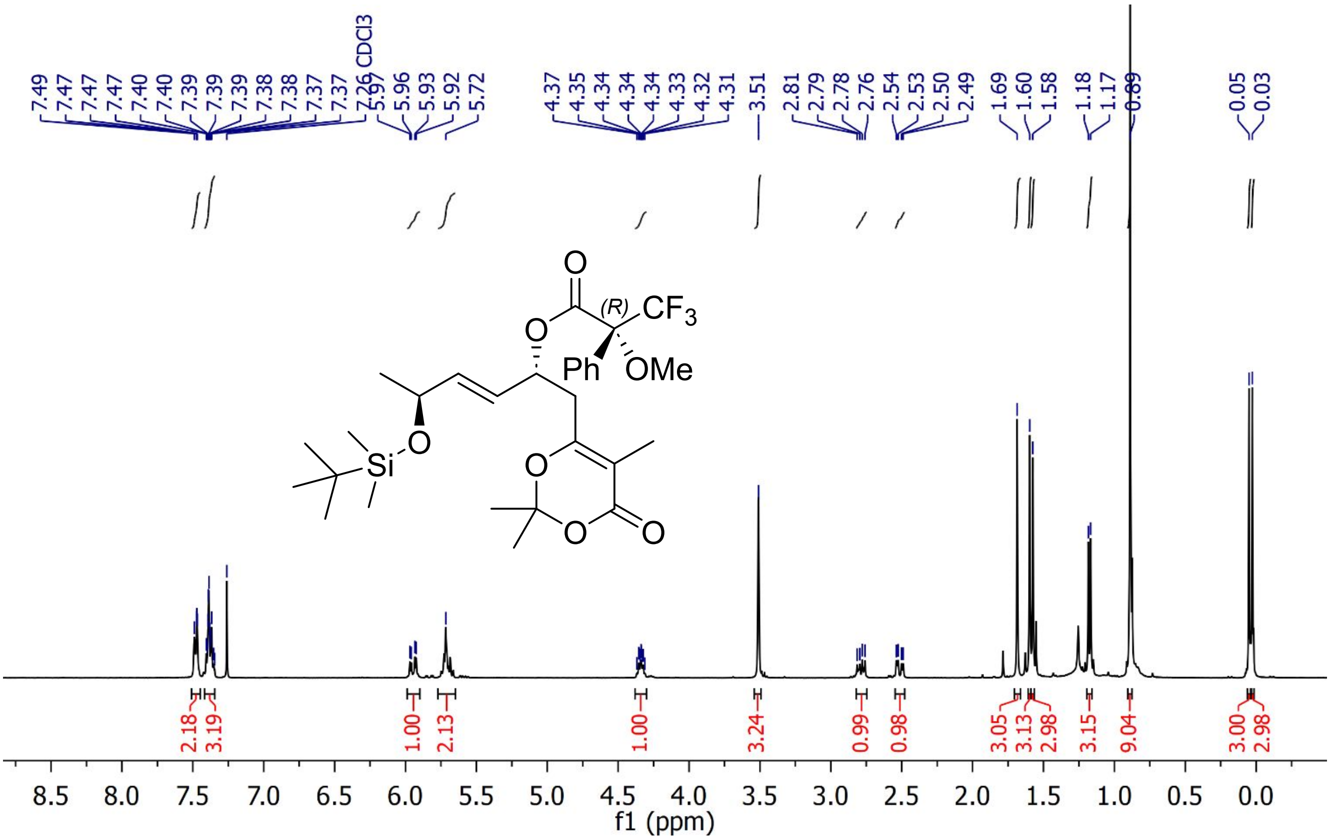

(R)-6-((S,E)-3-((*tert*-Butyldimethylsilyl)oxy)but-1-en-1-yl)-4-methoxy-3-methyl-5,6-dihydro-2H-pyran-2-one 17

^{1}H-NMR (400 MHz, $CDCl_3$)

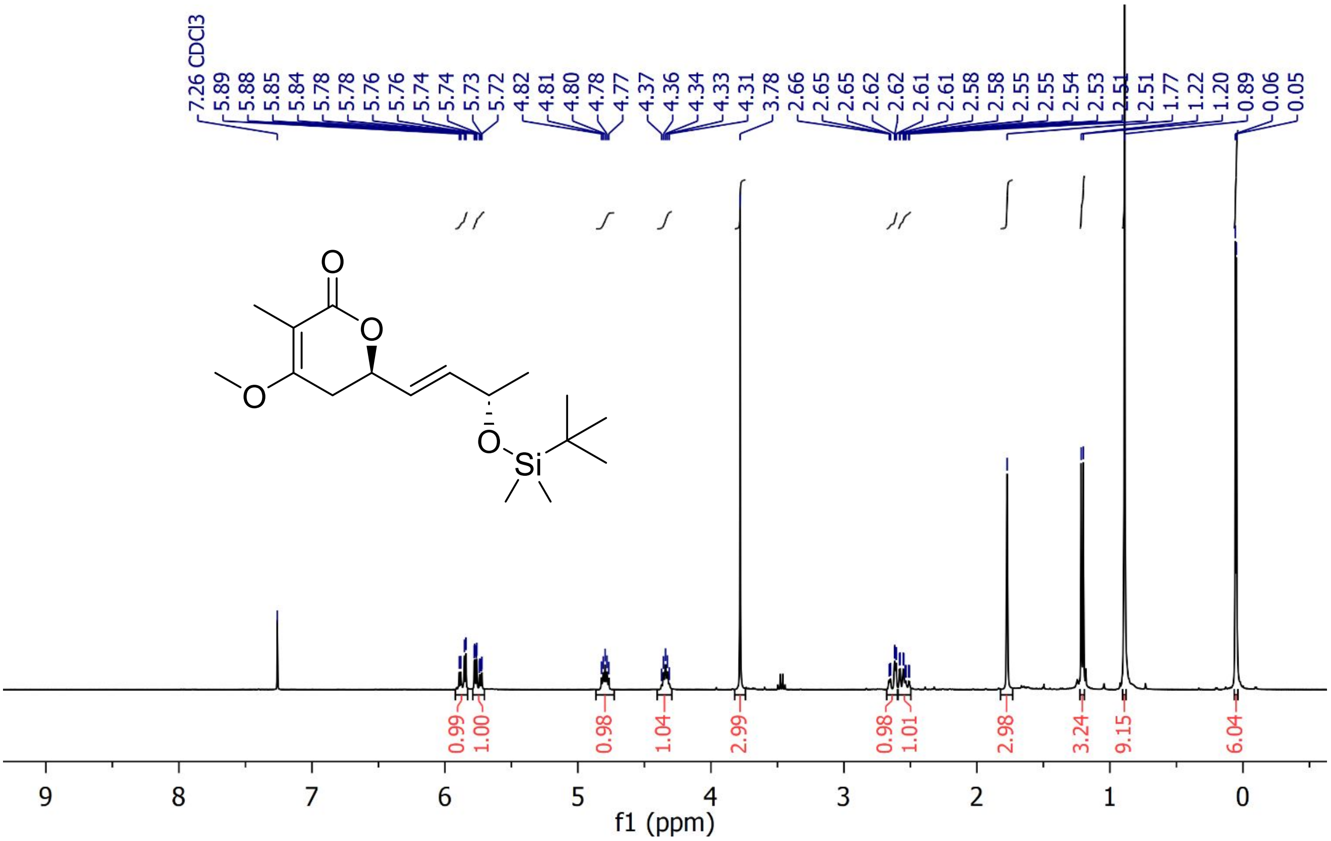

^{13}C-NMR (100 MHz, $CDCl_3$)

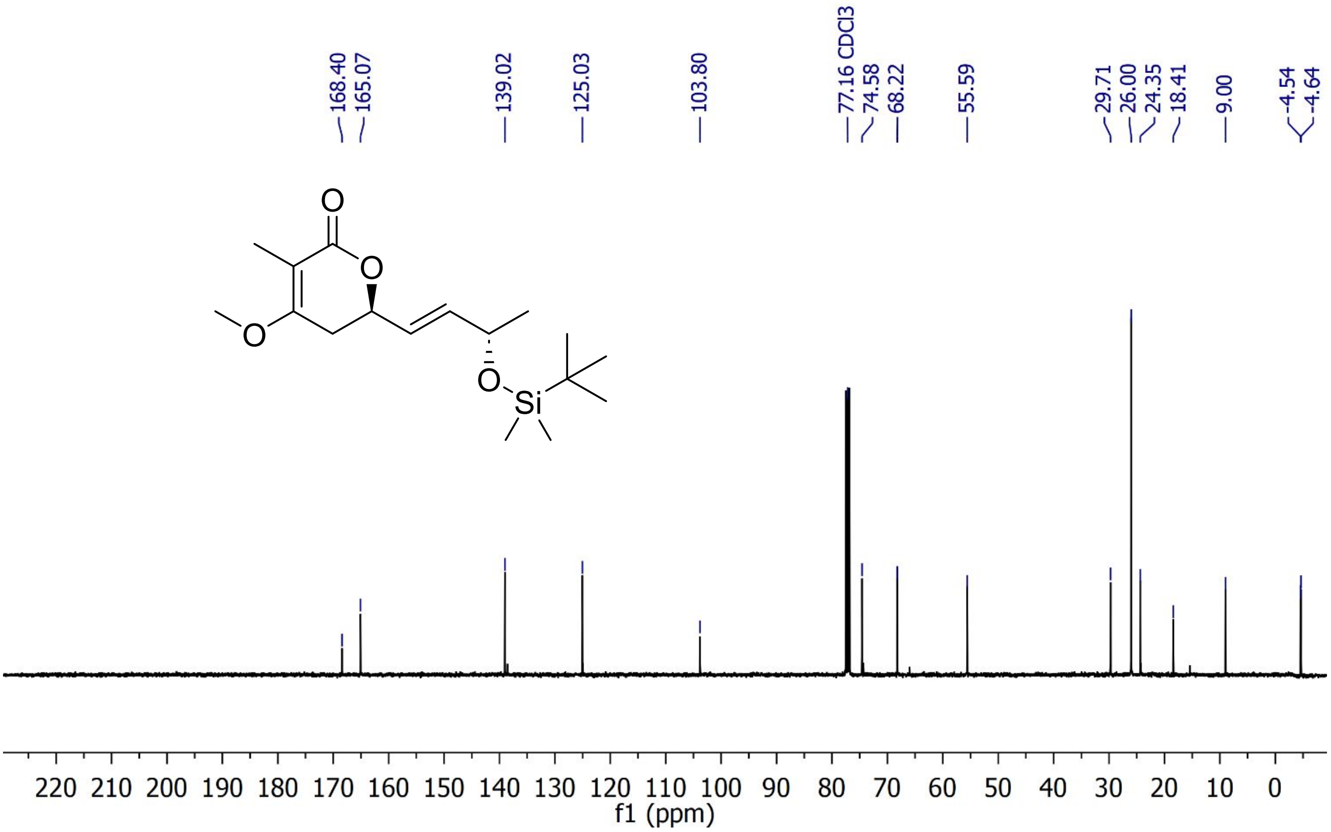

(R)-6-((S,E)-3-hydroxybut-1-en-1-yl)-4-methoxy-3-methyl-5,6-dihydro-2H-pyran-2-one S5

^{1}H-NMR (400 MHz, $CDCl_3$)

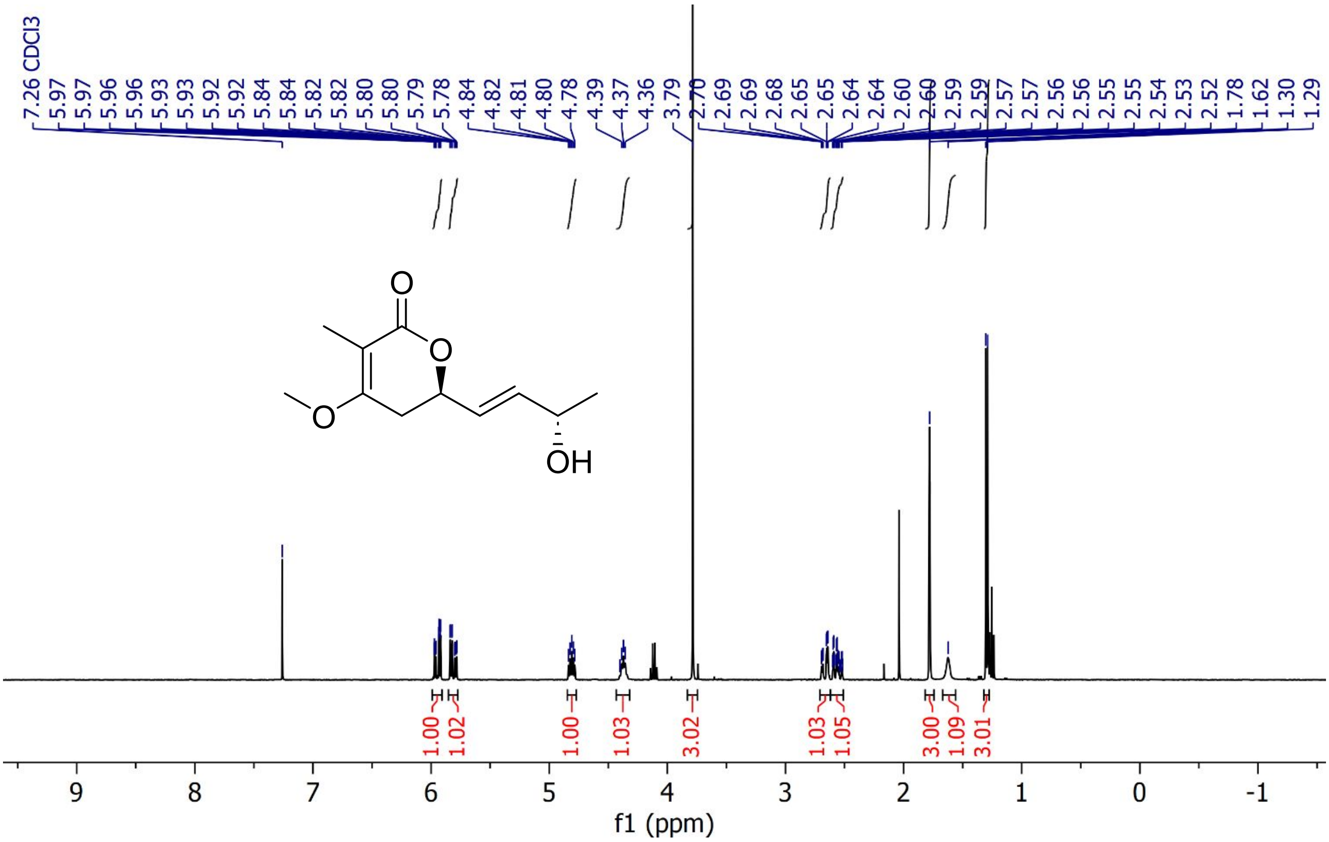

^{13}C-NMR (100 MHz, $CDCl_3$

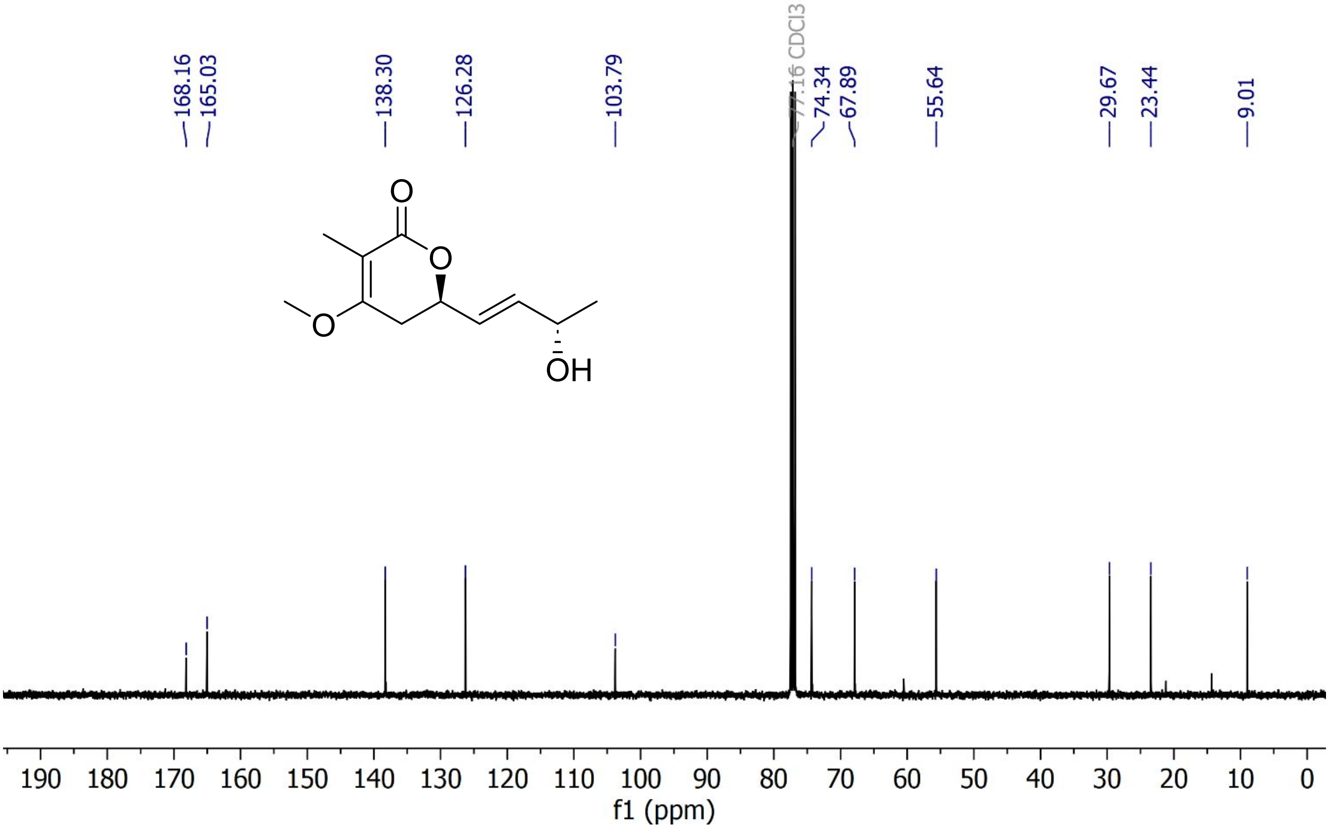

(S,E)-4-((R)-4-methoxy-5-methyl-6-oxo-3,6-dihydro-2H-pyran-2-yl)but-3-en-2-yl 2,2,2-trifluoroacetate 6

^{1}H-NMR (400 MHz, $CDCl_3$)

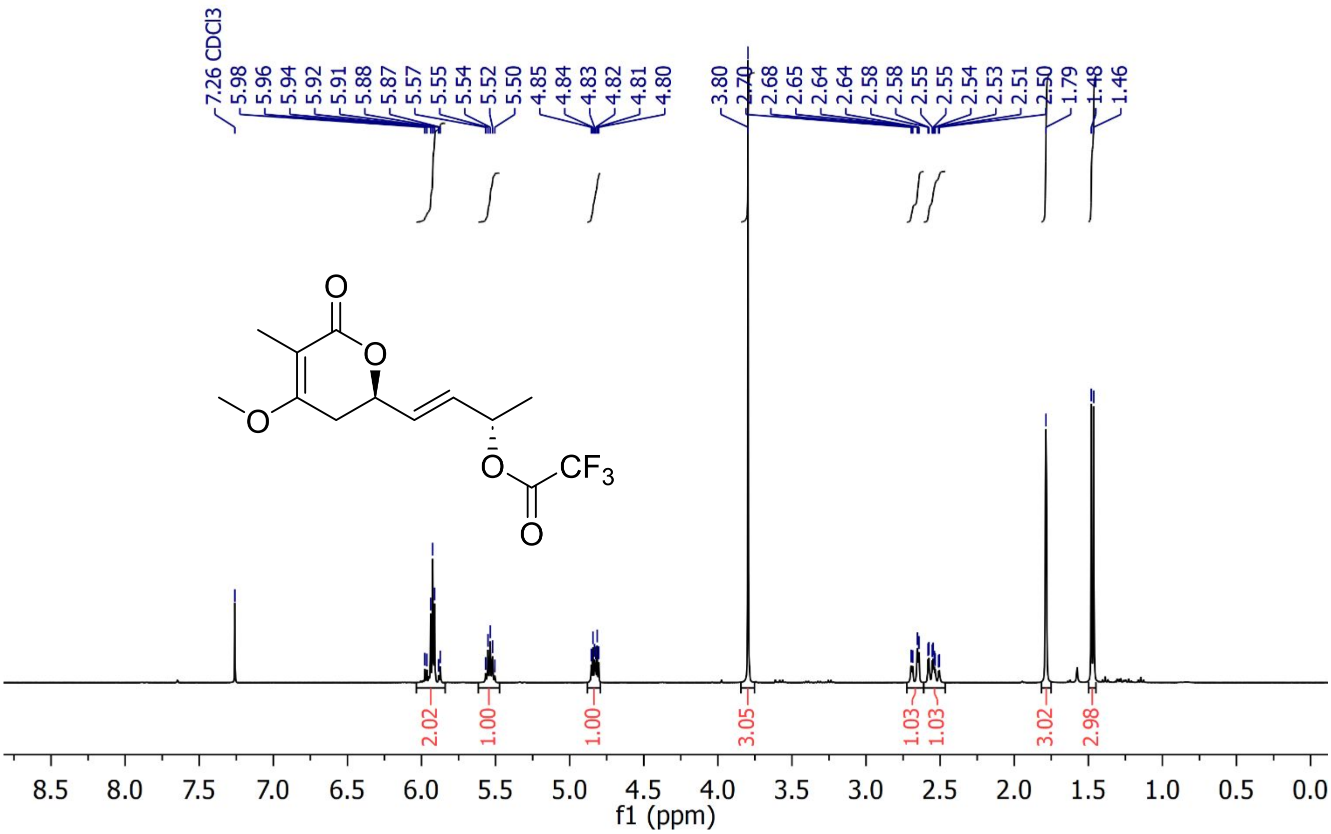

^{13}C-NMR (100 MHz, $CDCl_3$)

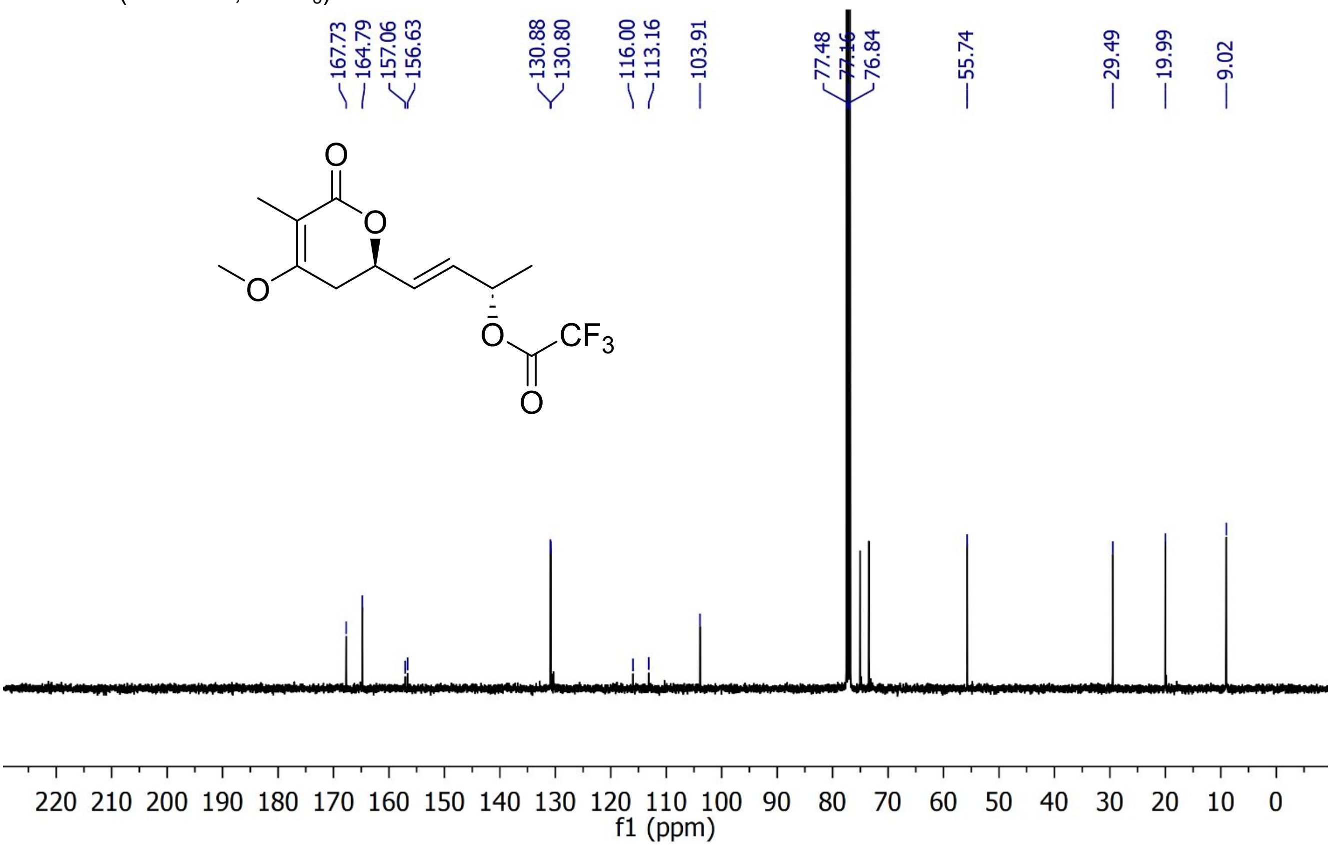

3-((2S,3R)-3-ethyl-3-(trimethylsilyl)oxiran-2-yl)propan-1-ol 20

^{1}H-NMR (400 MHz, $CDCl_3$)

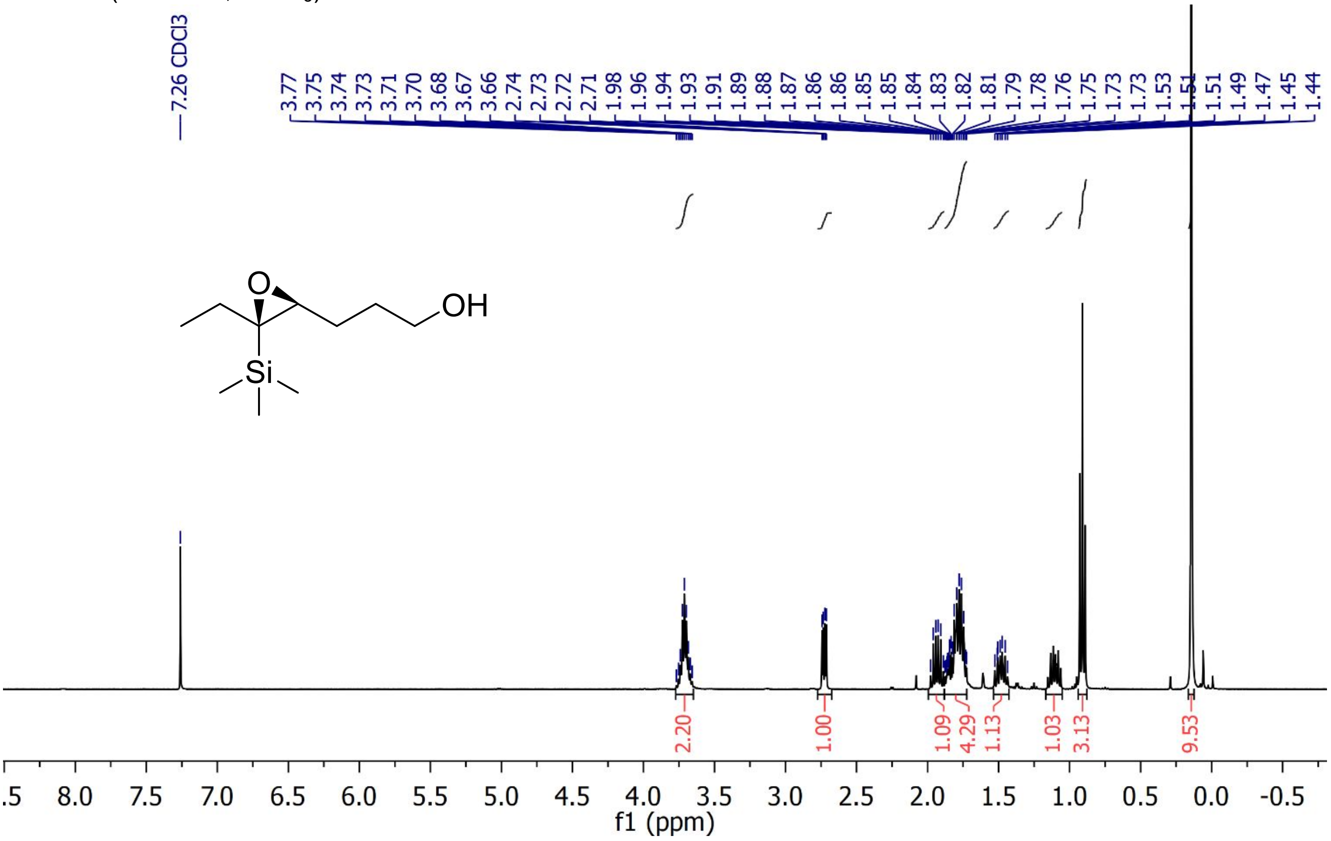

^{13}C-NMR (100 MHz, $CDCl_3$)

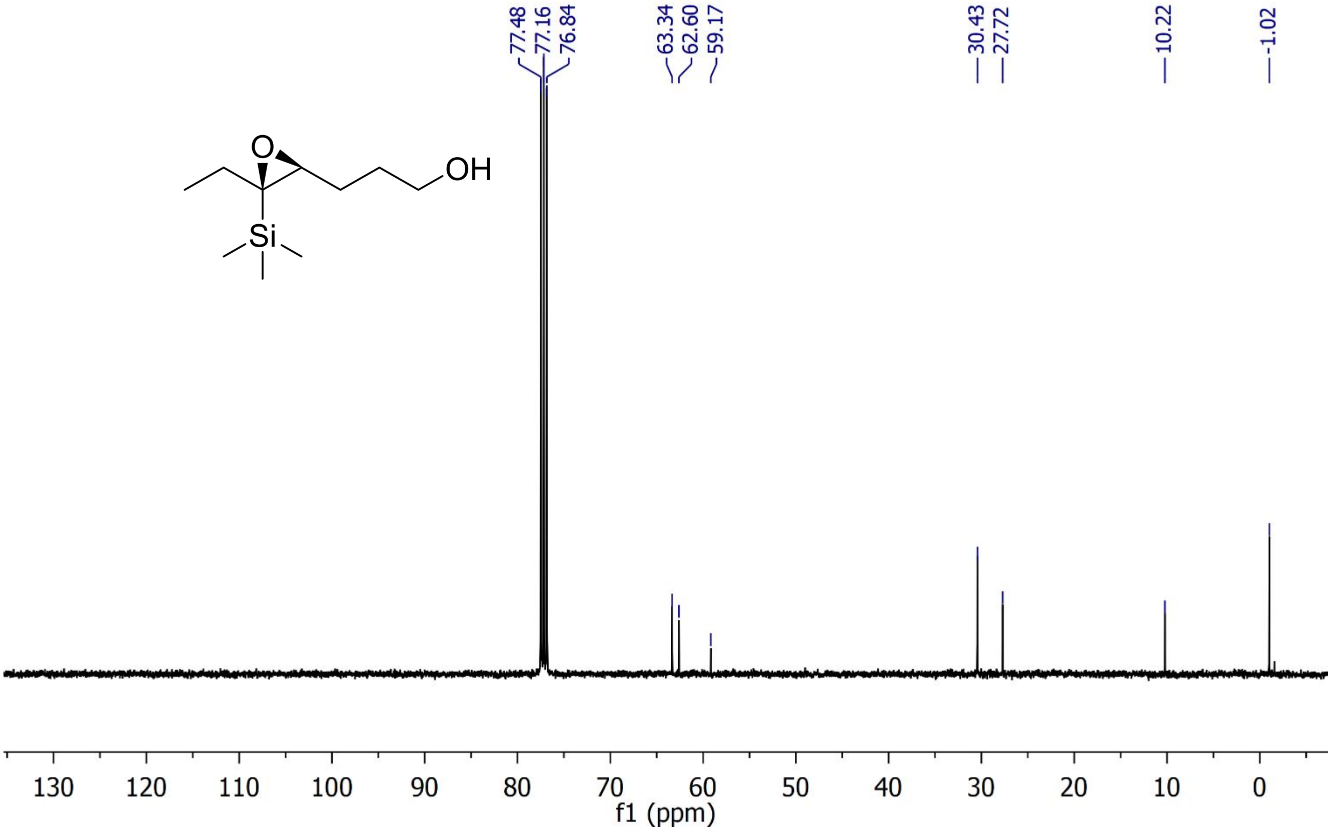

PNB ester S8

^{1}H-NMR (400 MHz, $CDCl_3$)

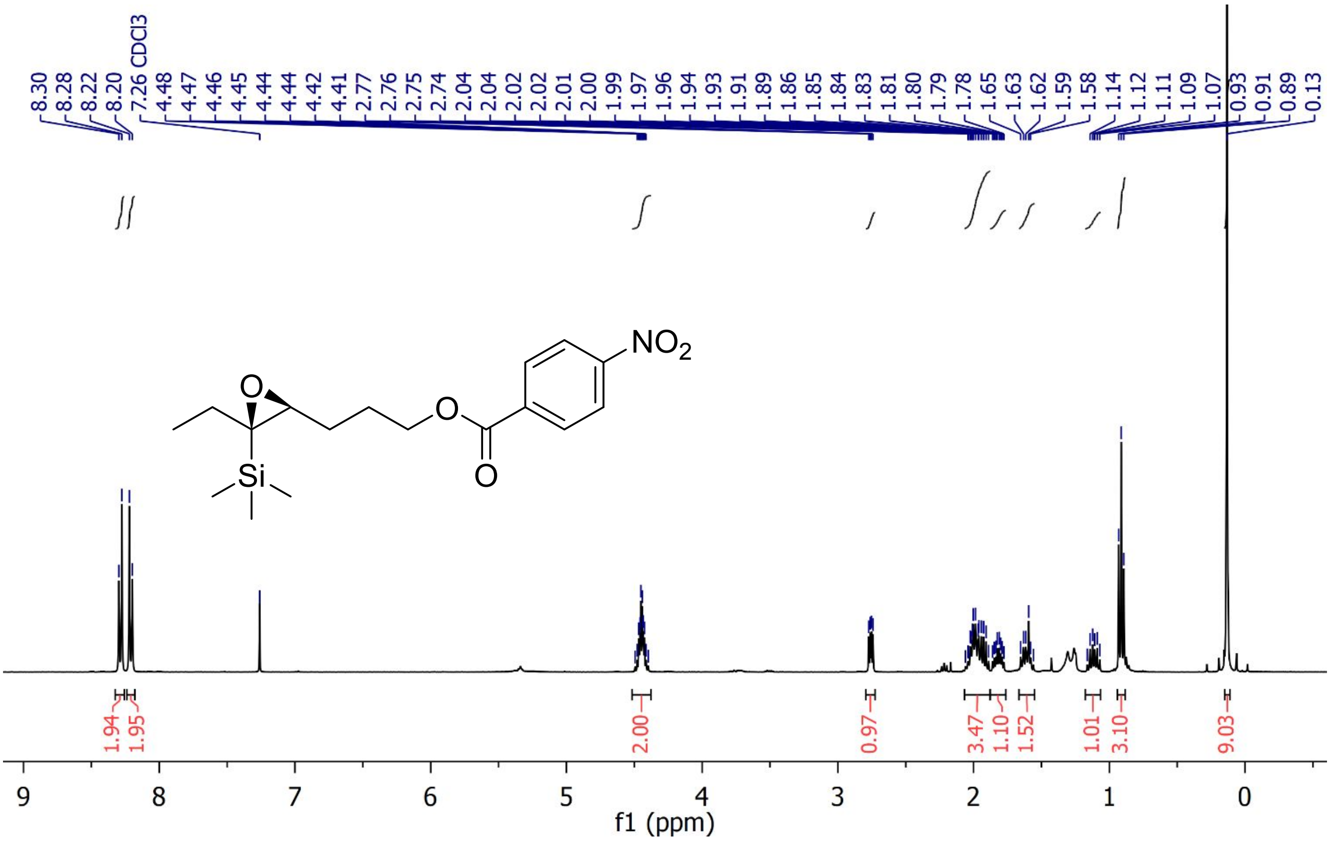

^{13}C-NMR (100 MHz, $CDCl_3$)

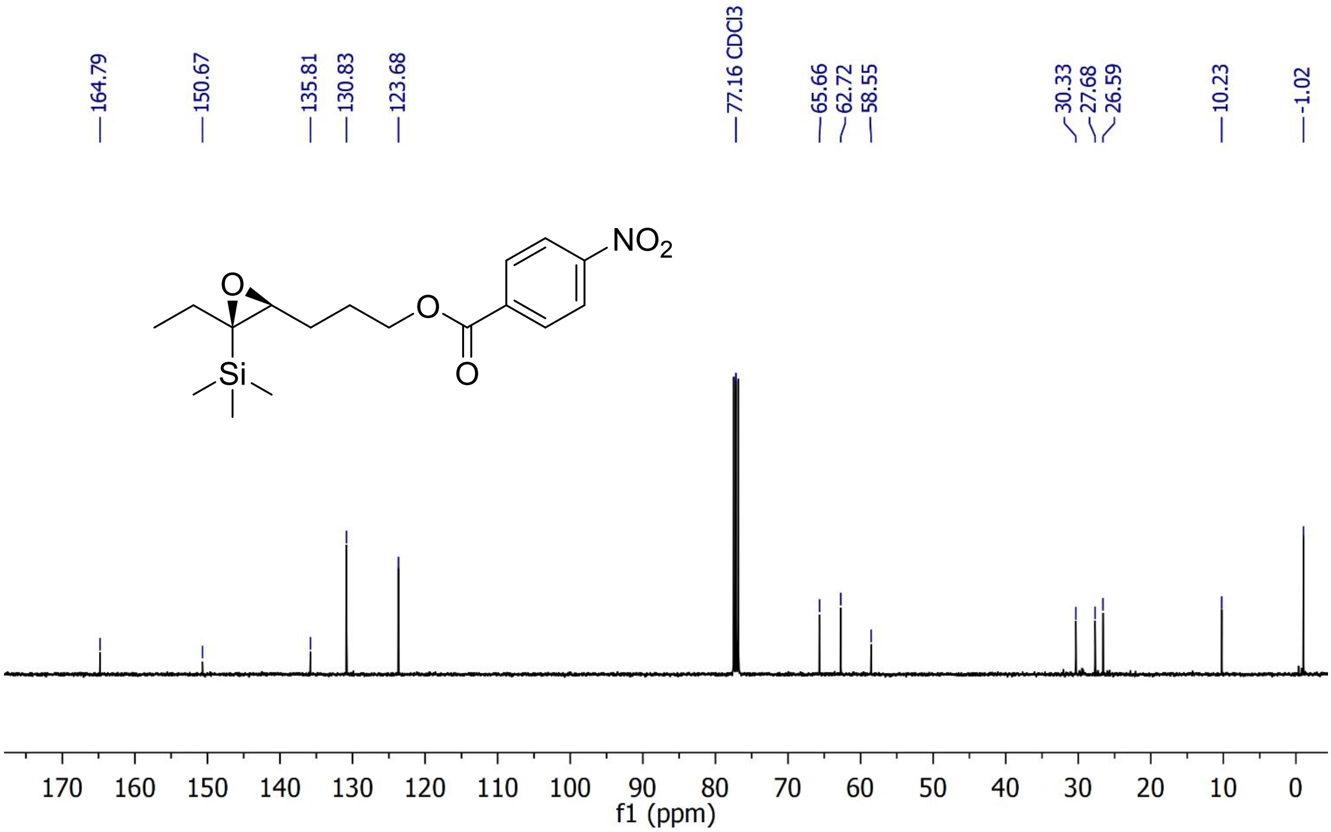

3-((2S,3R)-3-ethyl-3-(trimethylsilyl)oxiran-2-yl)propanal 10

^{1}H-NMR (400 MHz, $CDCl_3$)

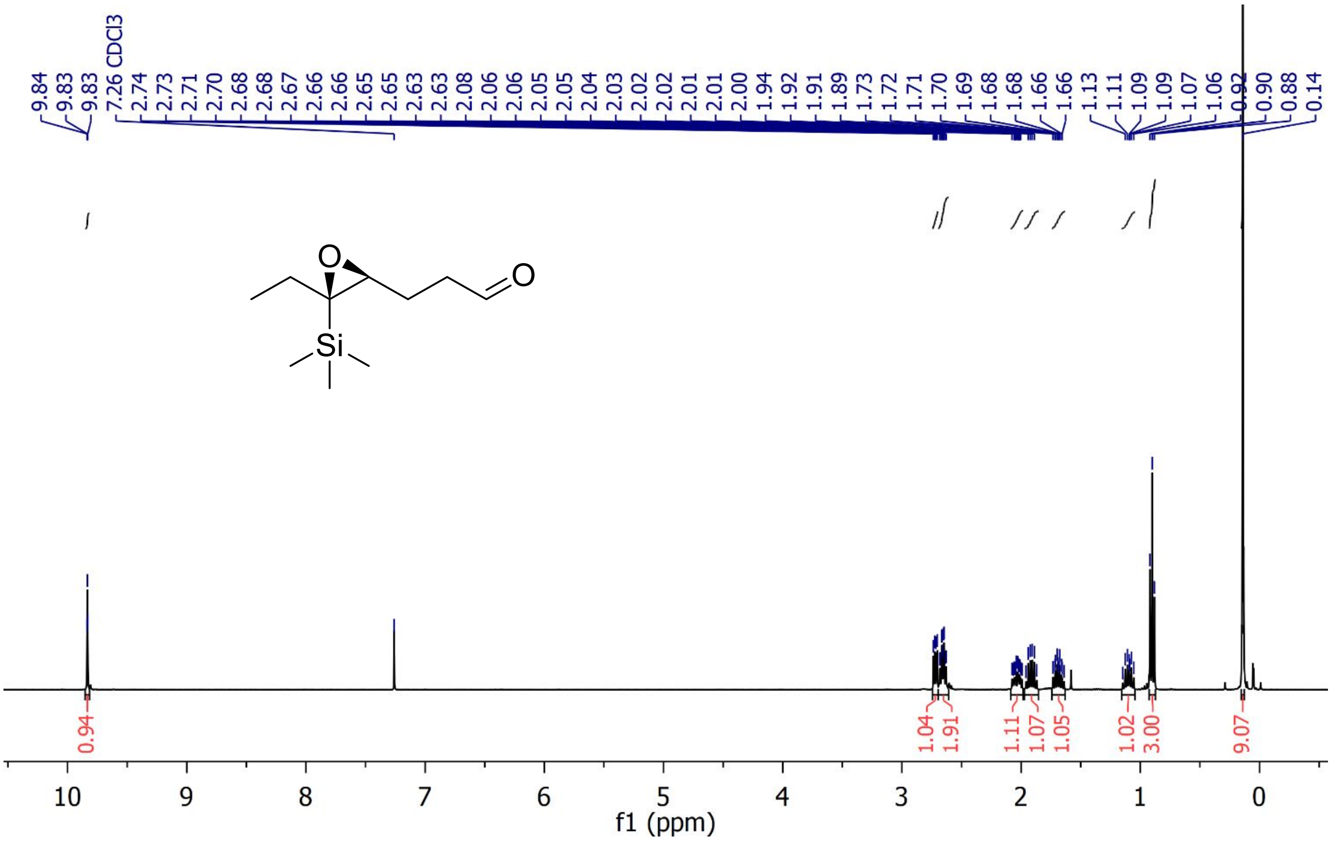

^{13}C-NMR (100 MHz, $CDCl_3$)

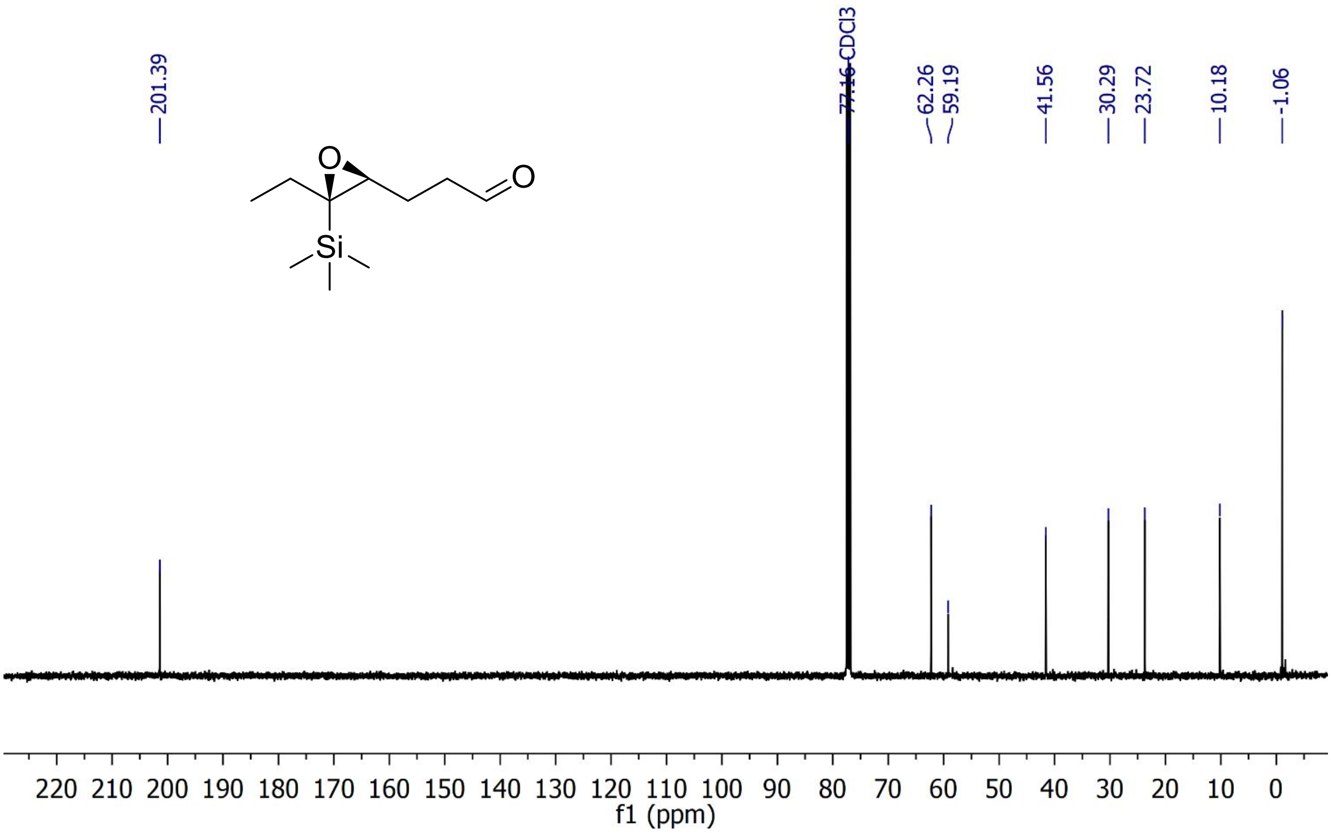

(2S,3S,6R)-2-ethyl-2-(trimethylsilyl)-6-((trimethylsilyl)ethynyl)tetrahydro-2H-pyran-3-ol 22

^{1}H-NMR (400 MHz, $CDCl_3$)

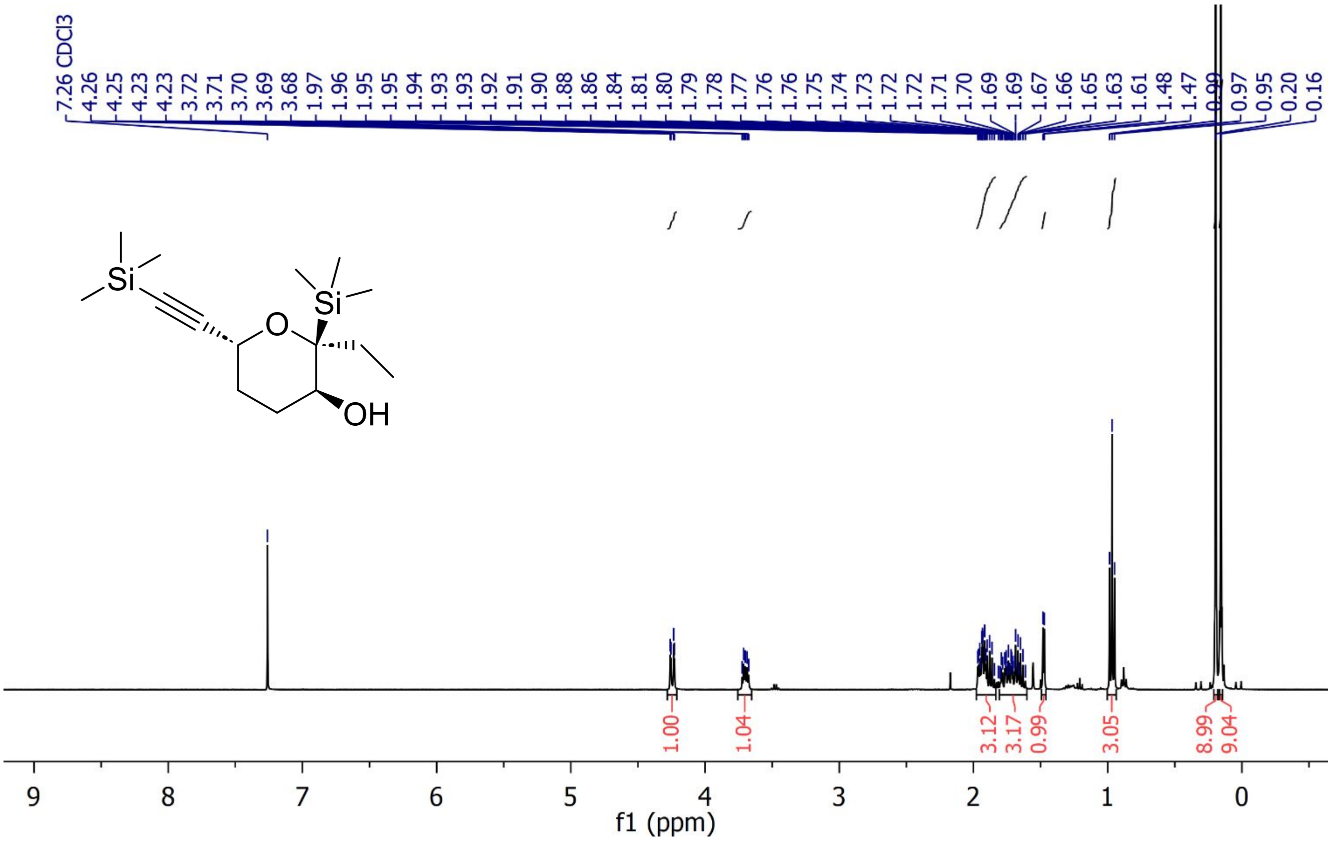

^{13}C-NMR (100 MHz, $CDCl_3$)

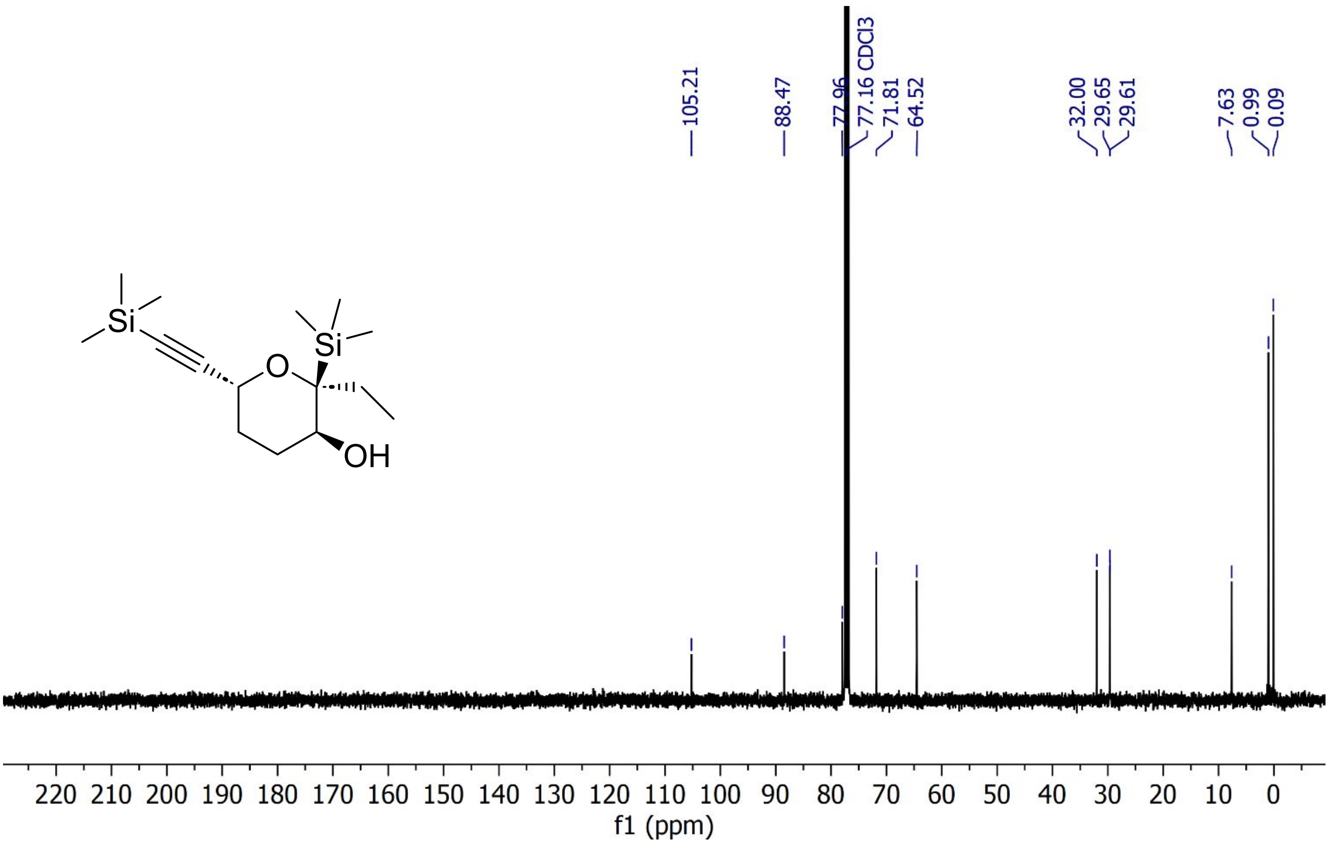

(2R,3S,6R)-2-Ethyl-6-ethynyltetrahydro-2H-pyran-3-ol 24

^{1}H-NMR (400 MHz, $CDCl_3$)

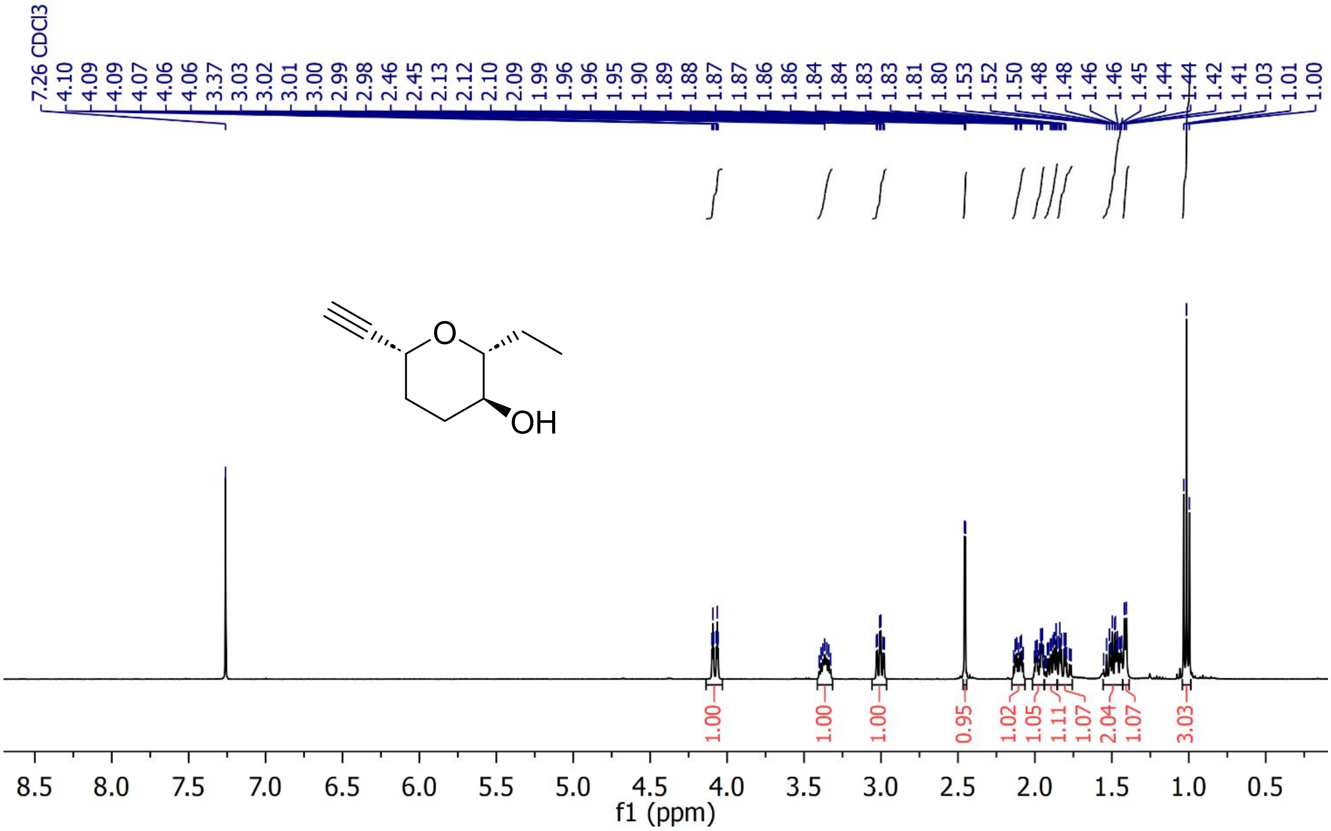

^{13}C-NMR (100 MHz, $CDCl_3$)

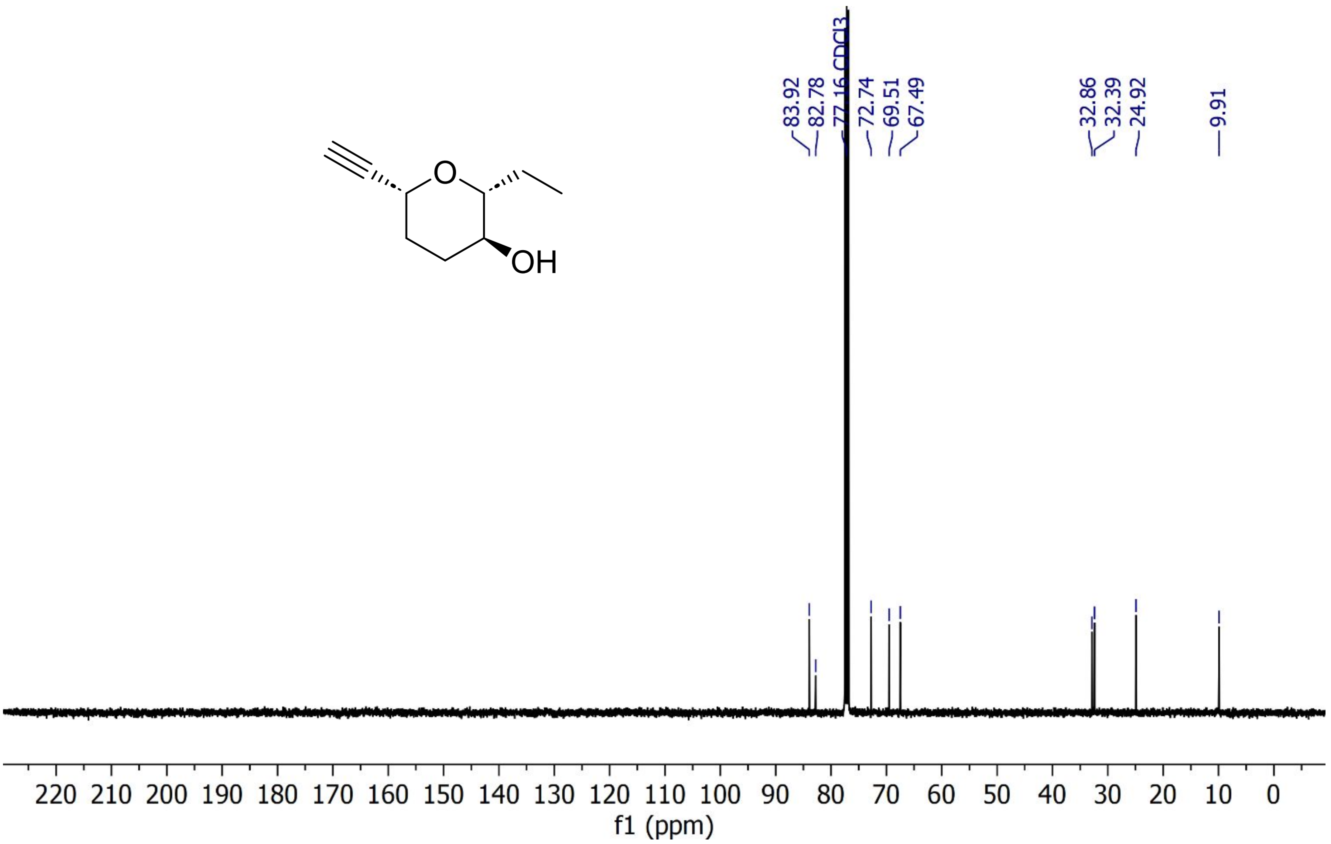

(2R,6R)-2-ethyl-6-ethynyldihydro-2H-pyran-3(4H)-one 25

^{1}H-NMR (400 MHz, $CDCl_3$)

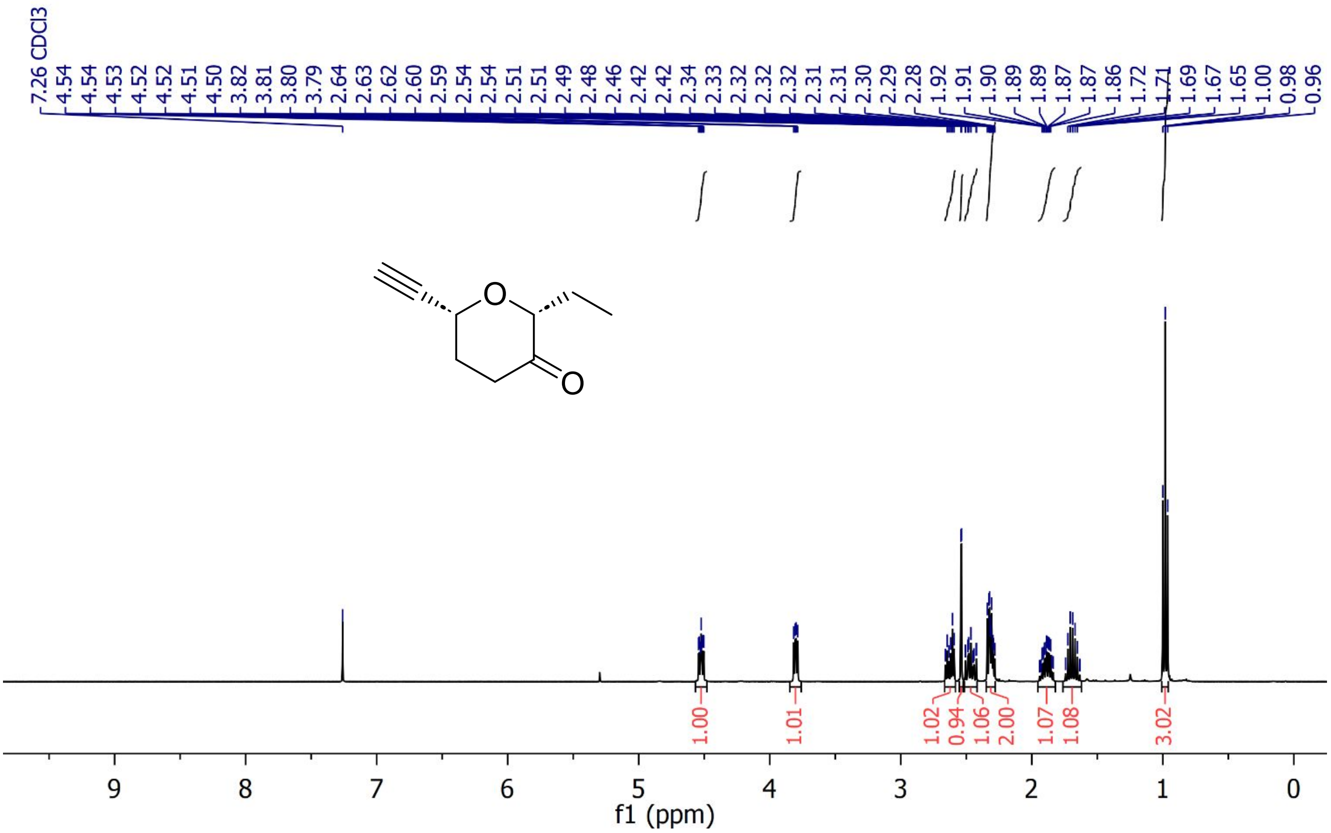

^{13}C-NMR (100 MHz, $CDCl_3$)

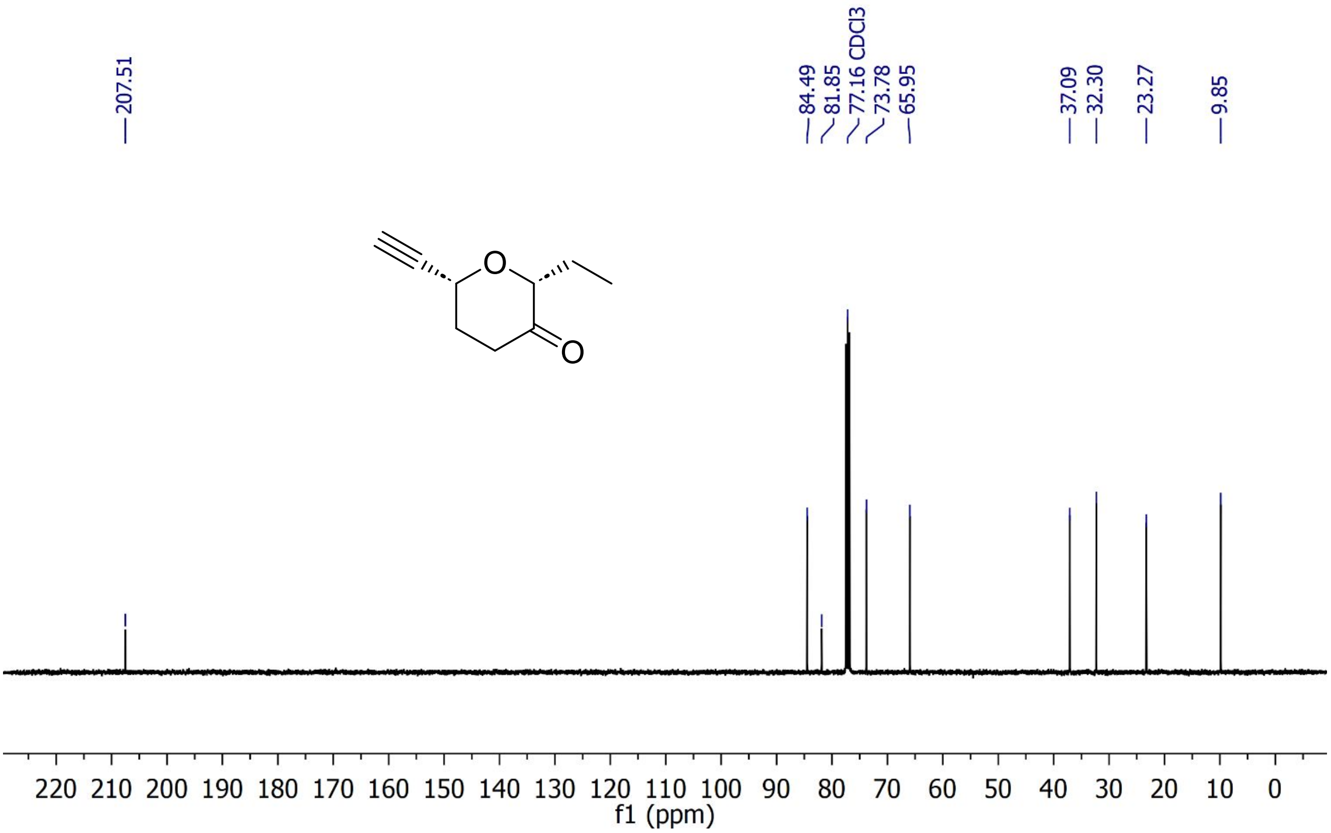

(2R,3R,6R)-2-ethyl-6-ethynyl-3-methyltetrahydro-2H-pyran-3-ol 26

^{1}H-NMR (500 MHz, $CDCl_3$)

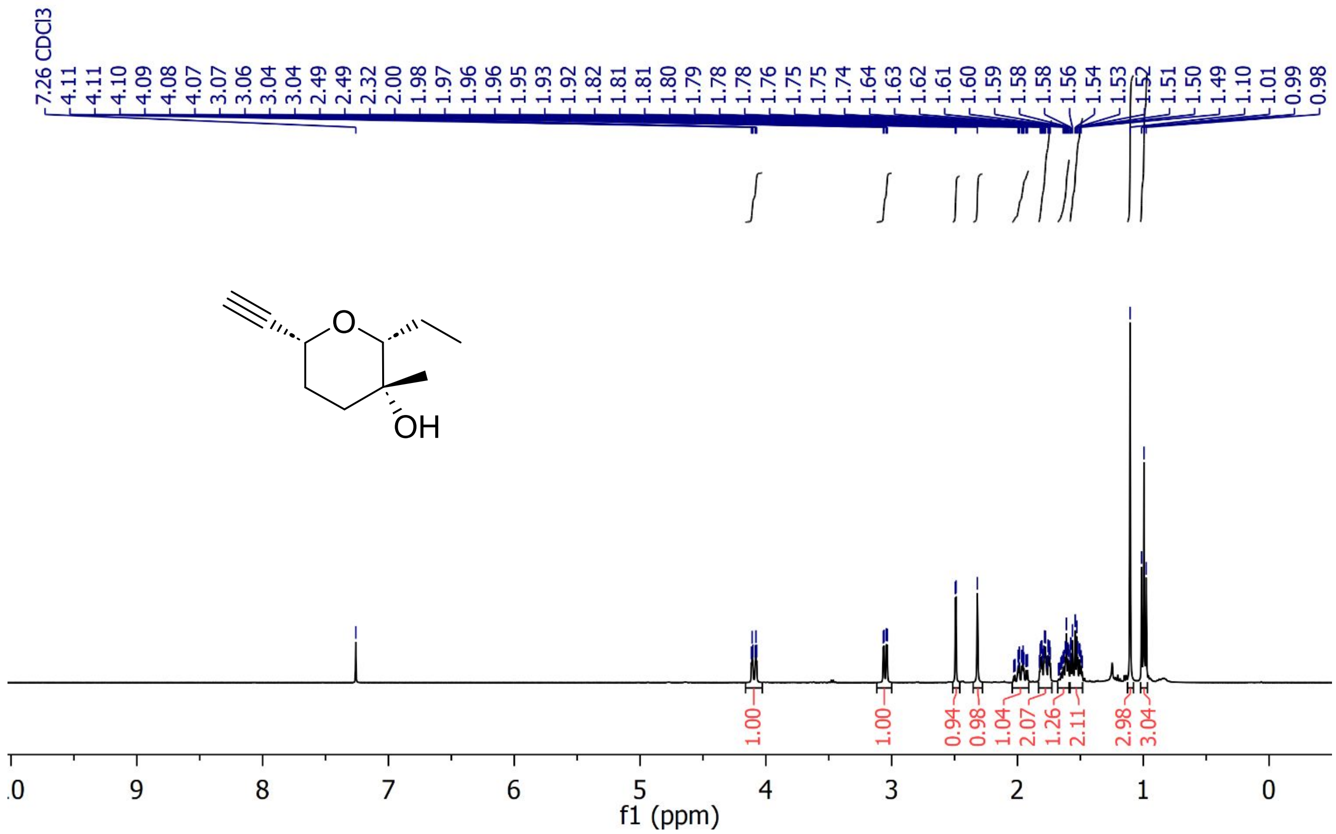

^{13}C-NMR (125 MHz, $CDCl_3$)

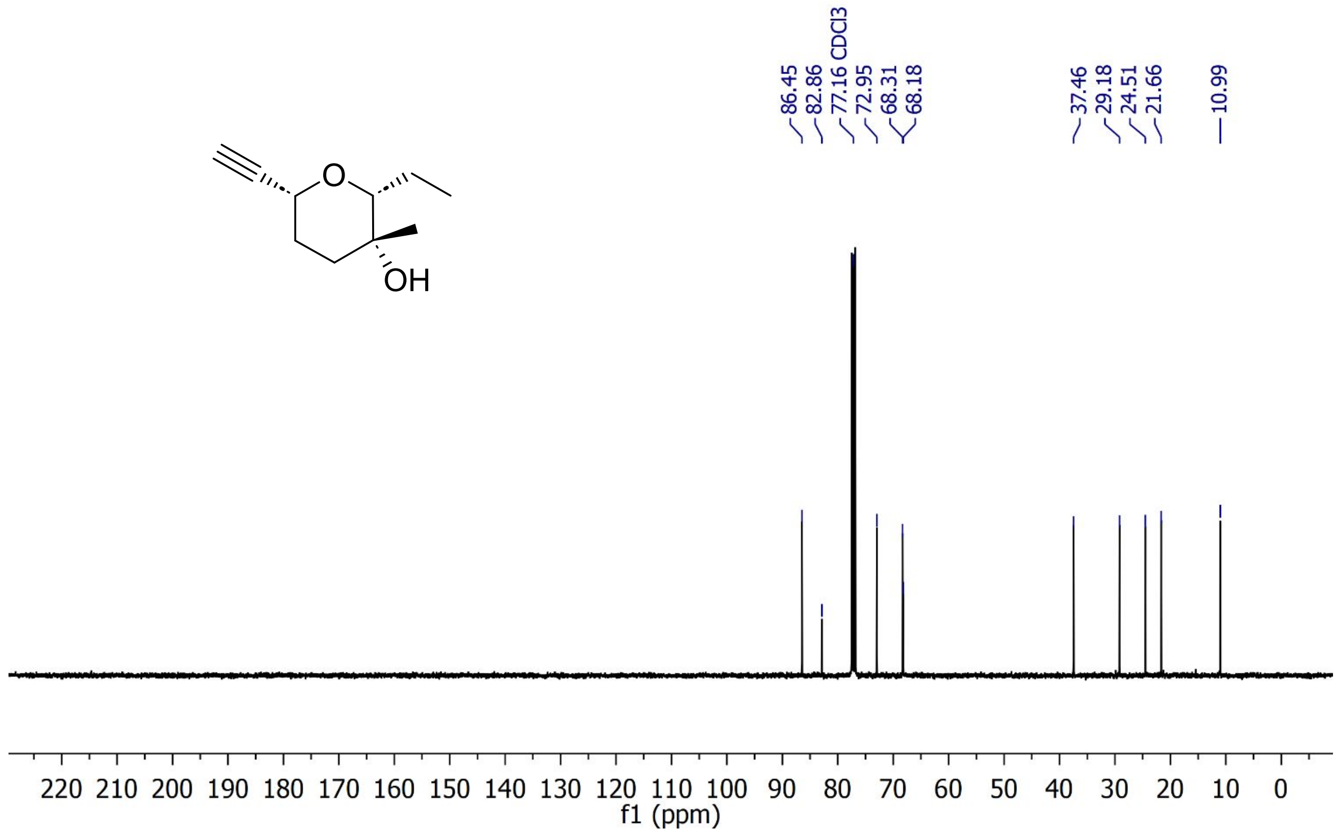

(2R,3S,6R)-2-ethyl-6-ethynyl-3-methyltetrahydro-2H-pyran-3-ol S9

^{1}H-NMR (400 MHz, $CDCl_3$)

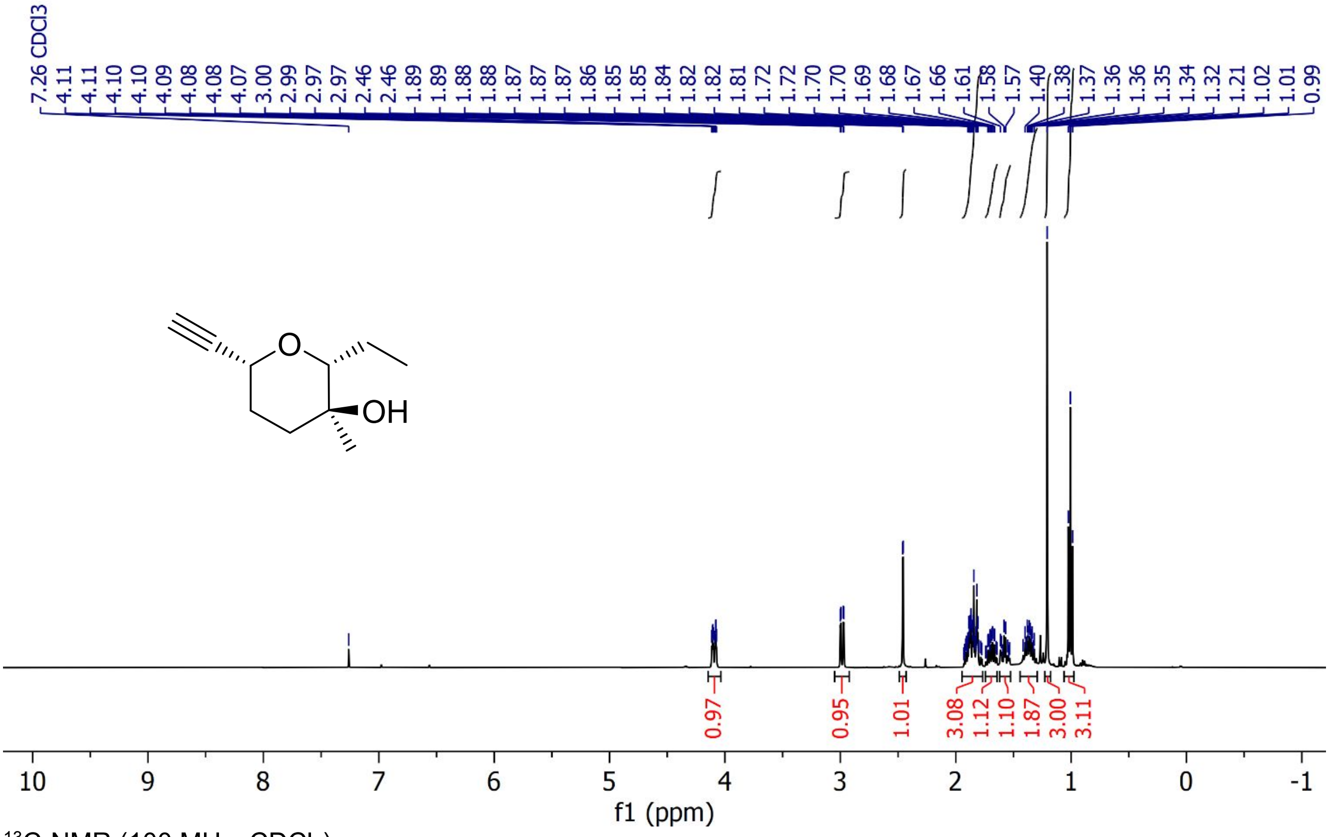

^{13}C-NMR (100 MHz, $CDCl_3$)

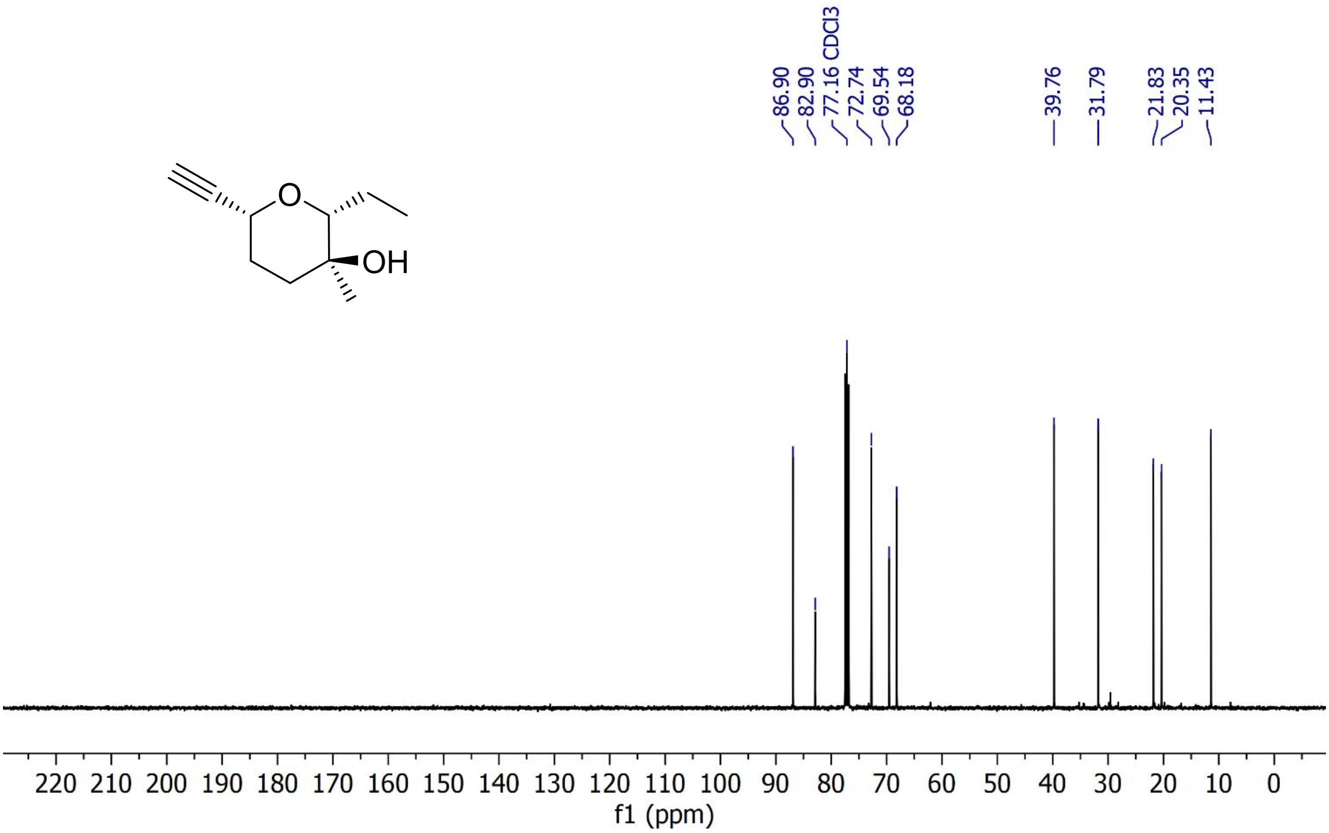

Triethyl(((2R,3R,6R)-2-ethyl-6-ethynyl-3-methyltetrahydro-2H-pyran-3-yl)oxy)silane 27

^{1}H-NMR (400 MHz, $CDCl_3$)

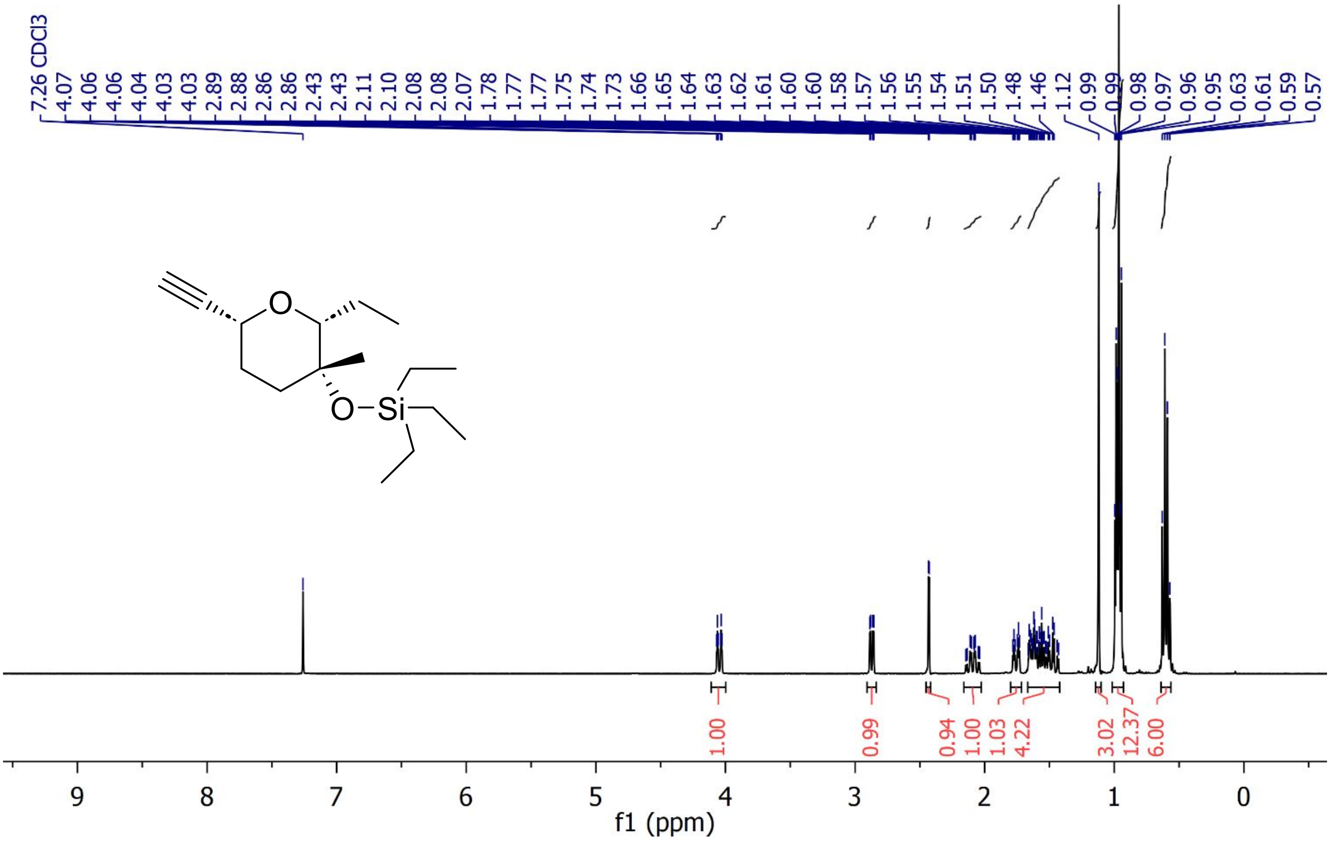

^{13}C-NMR (100 MHz, $CDCl_3$)

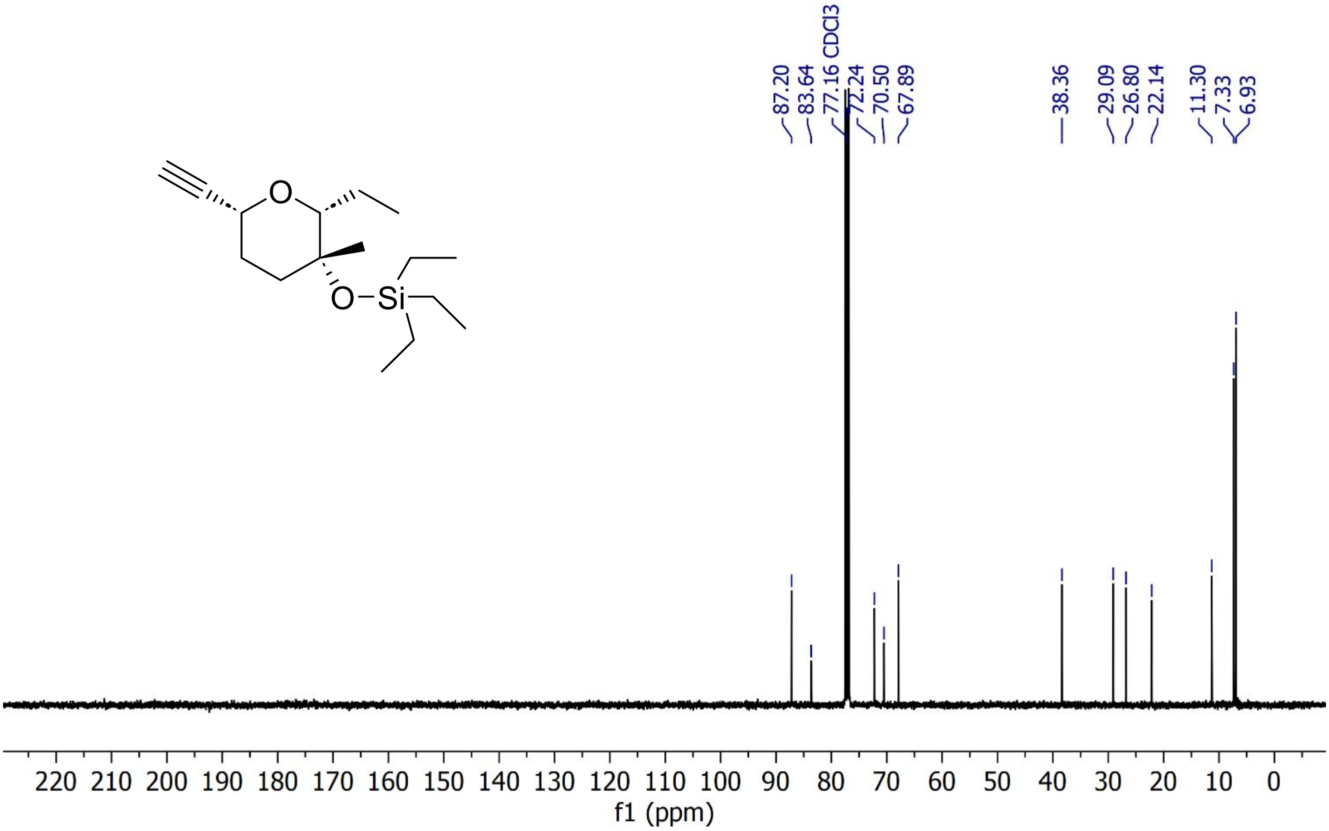

Triethyl(((2R,3S,6R)-2-ethyl-6-ethynyl-3-methyltetrahydro-2H-pyran-3-yl)oxy)silane S10

^{1}H-NMR (400 MHz, $CDCl_3$)

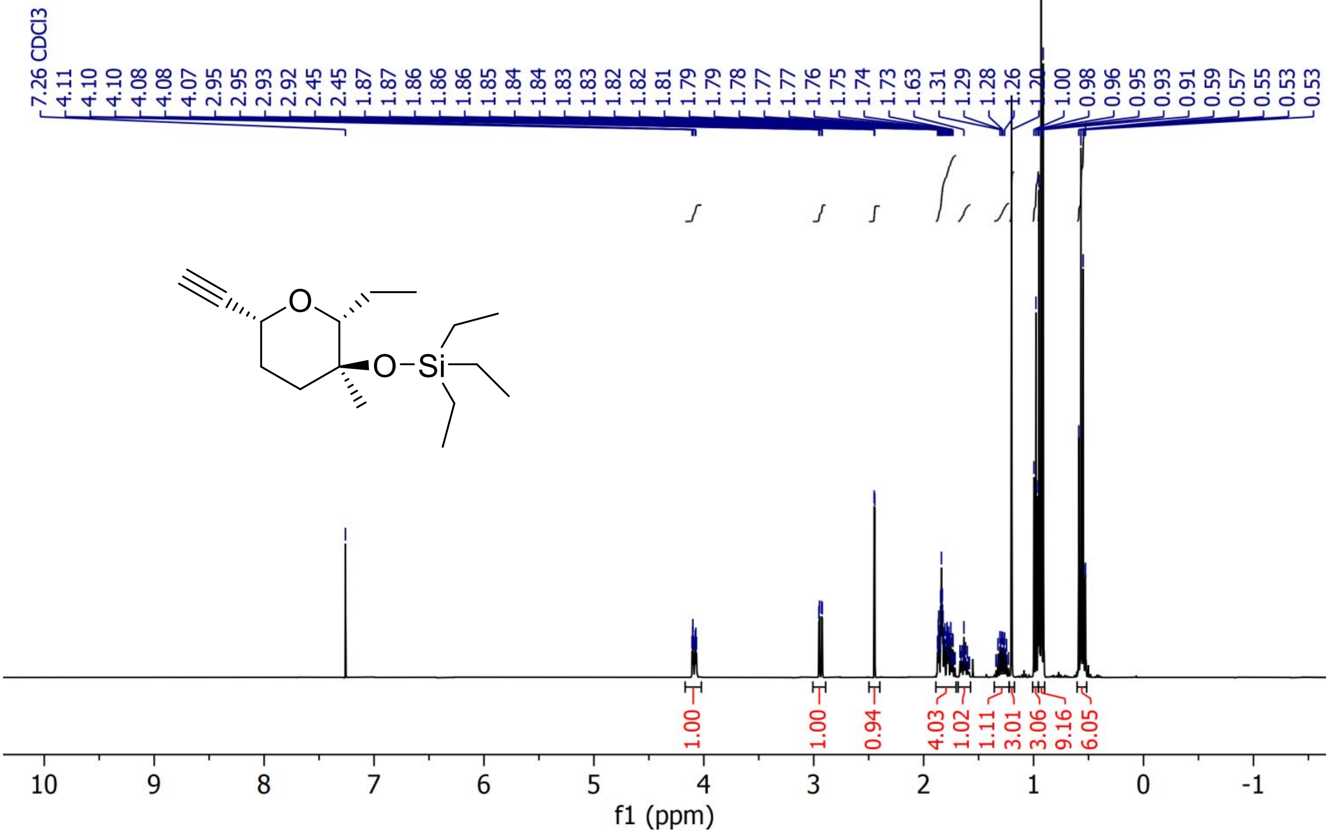

^{13}C-NMR (100 MHz, $CDCl_3$)

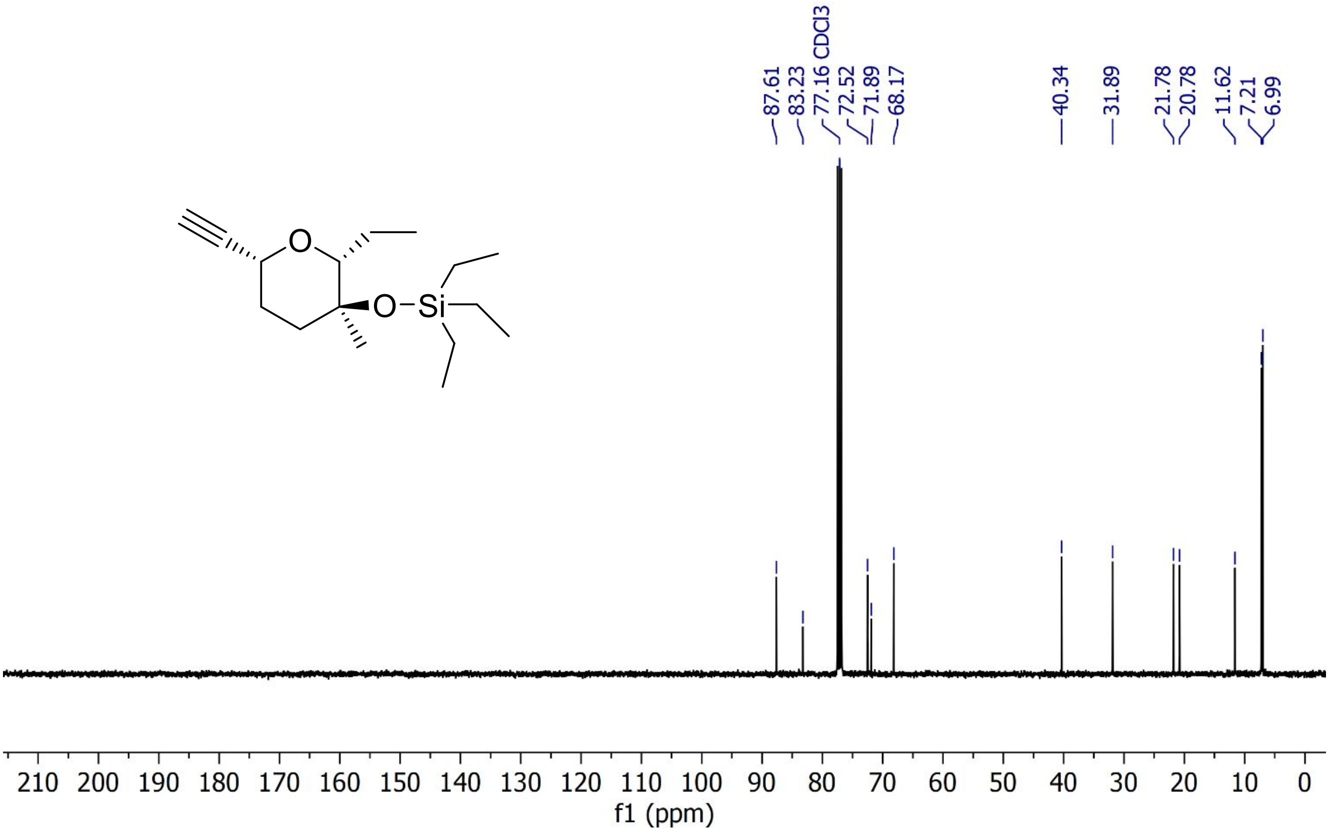

***tert*-butyl(((2R,3S,6R)-2-ethyl-6-ethynyl-3-methyltetrahydro-2H-pyran-3-yl)oxy)dimethylsilane S11**

^{1}H-NMR (400 MHz, $CDCl_3$)

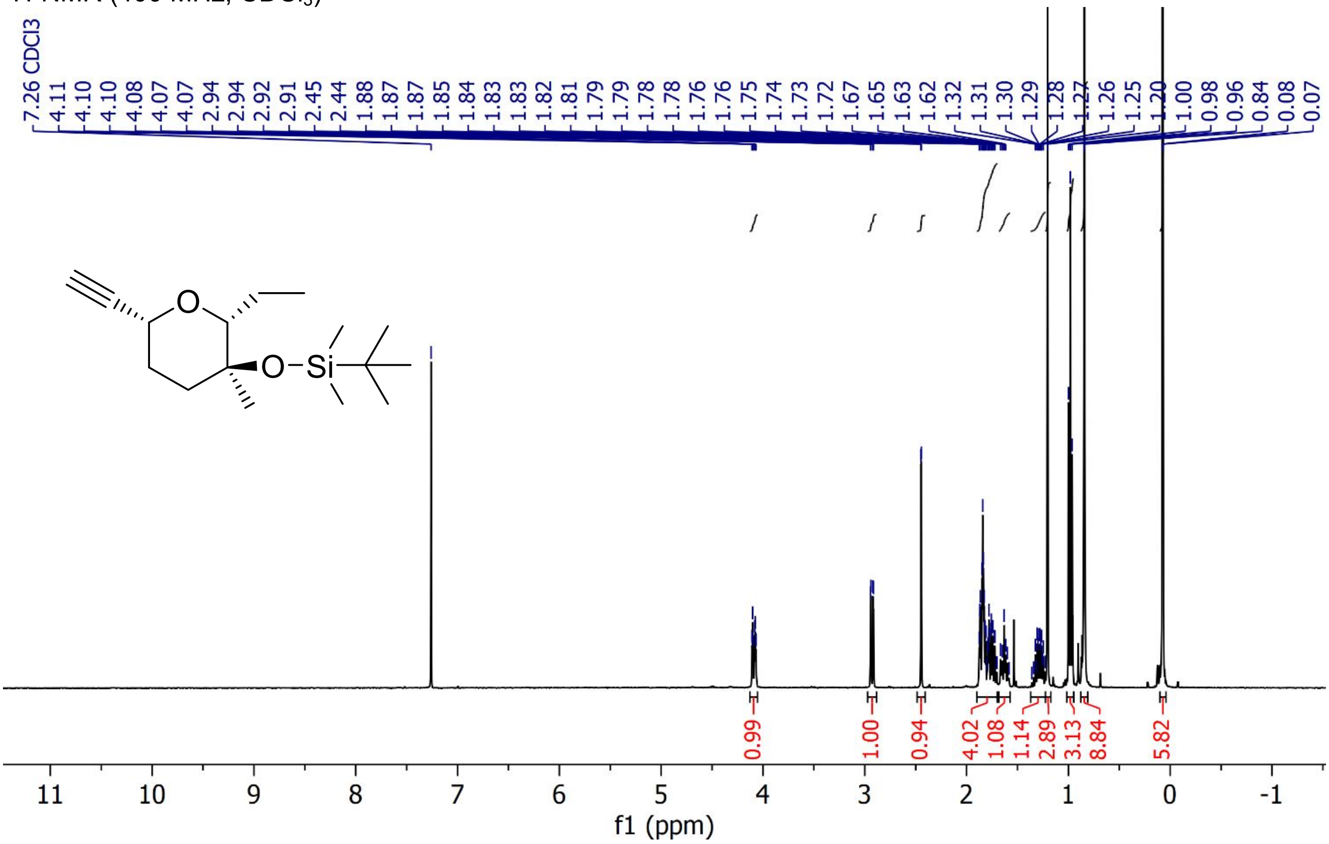

^{13}C-NMR (100 MHz, $CDCl_3$)

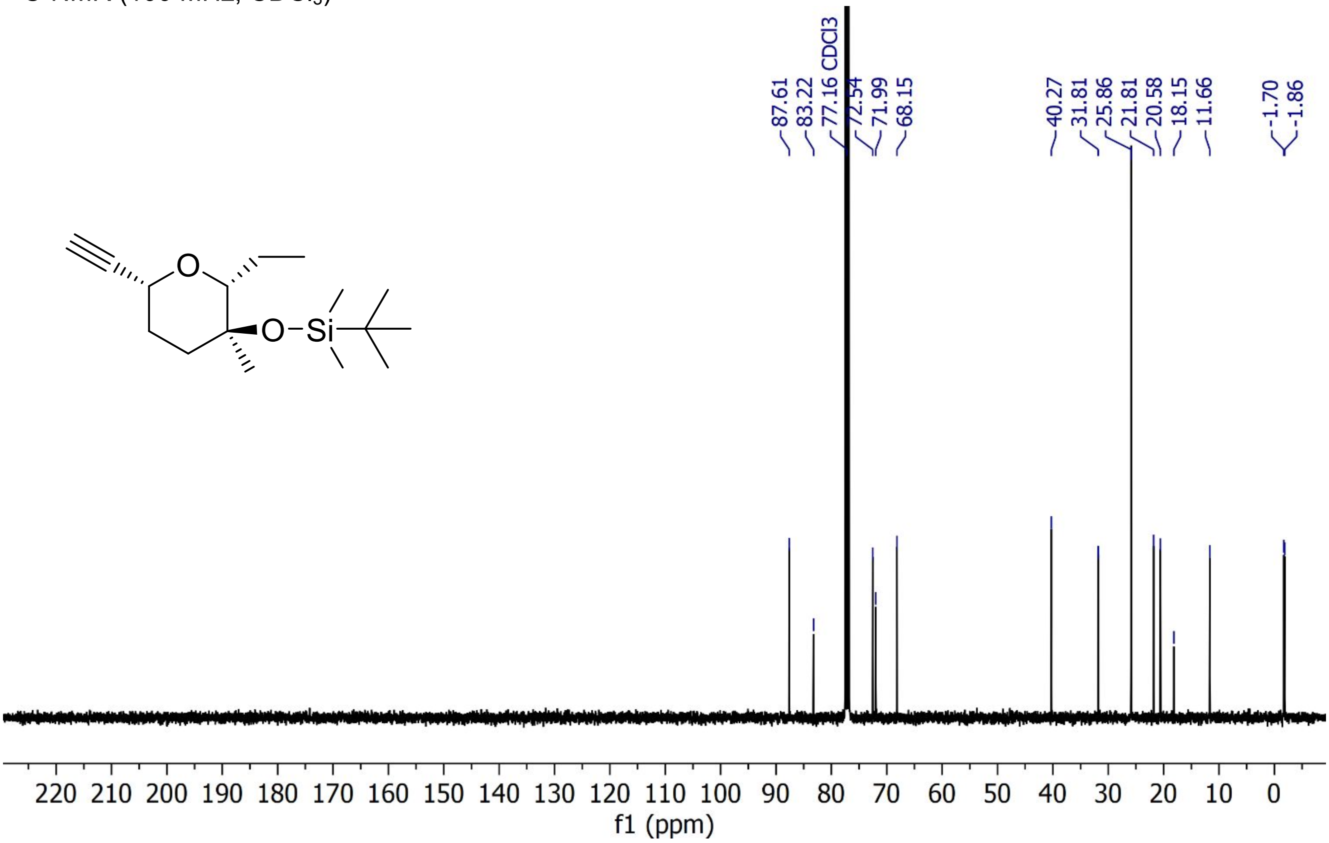

triethyl(((2R,3R,6R)-2-ethyl-3-methyl-6-((E)-1-(tributylstannyl)prop-1-en-2-yl)tetrahydro-2H-pyran-3-yl)oxy)silane 7

^{1}H-NMR (400 MHz, $CDCl_3$)

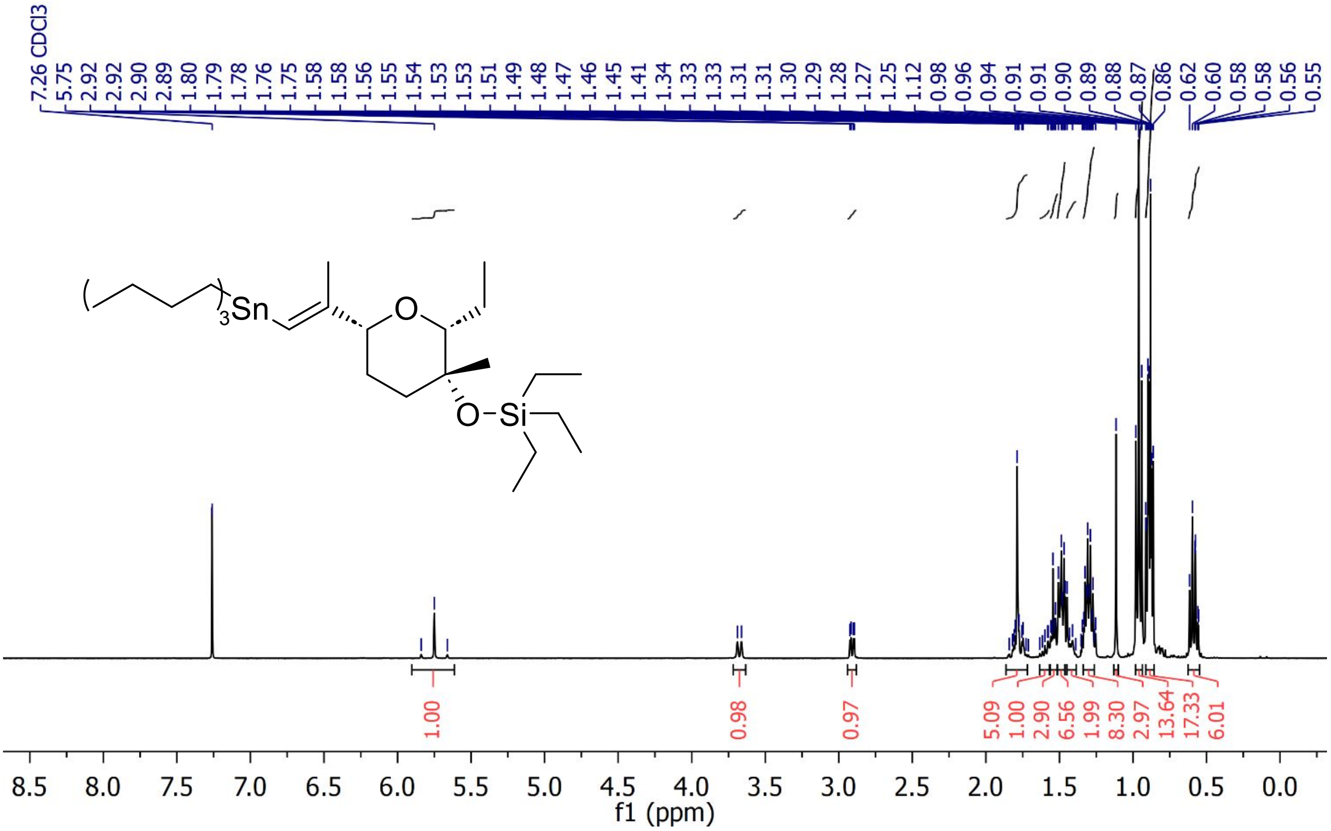

^{13}C-NMR (100 MHz, $CDCl_3$

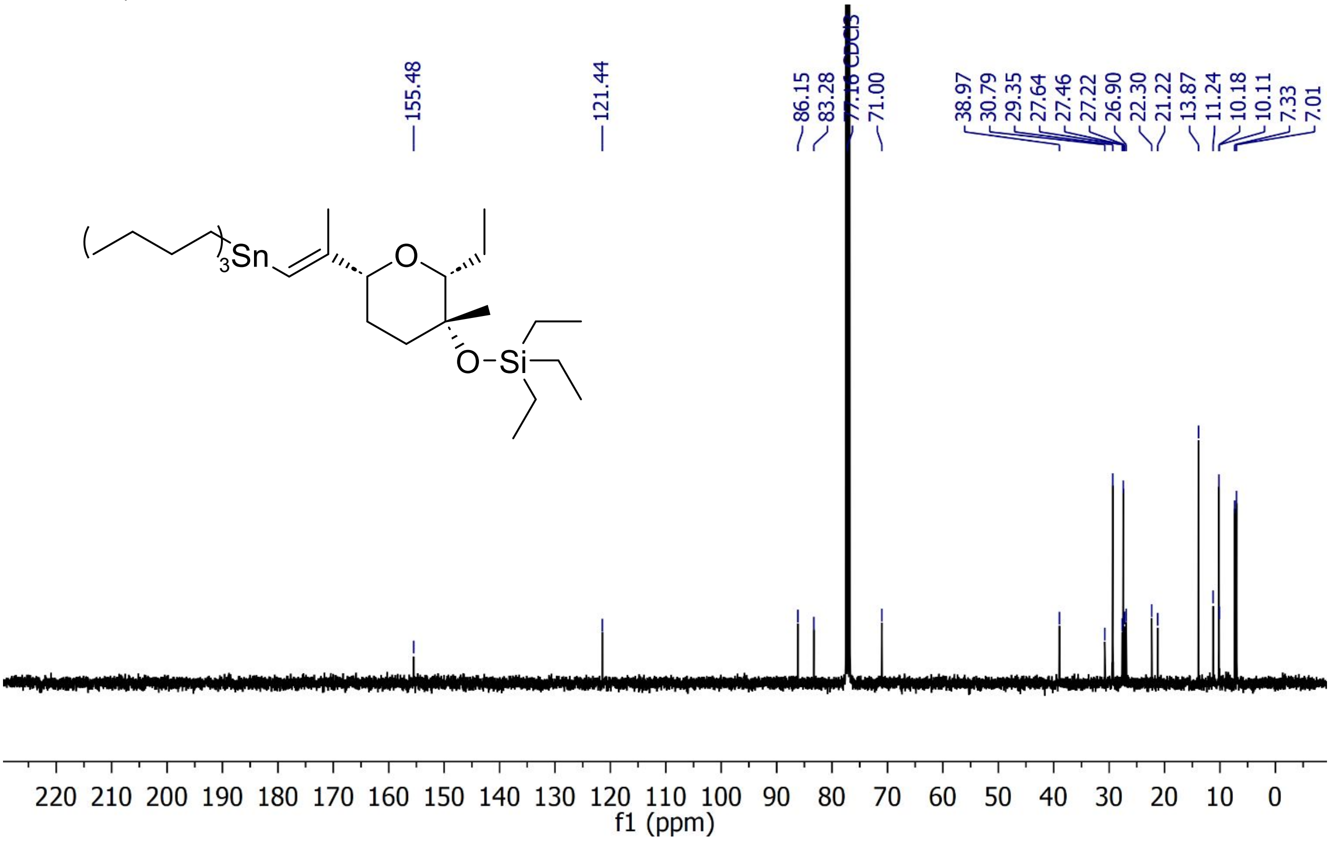

Triethyl(((2R,3S,6R)-2-ethyl-3-methyl-6-((E)-1-(tributylstannyl)prop-1-en-2-yl)tetrahydro-2H-pyran-3-yl)oxy)silane S12

^{1}H-NMR (400 MHz, $CDCl_3$)

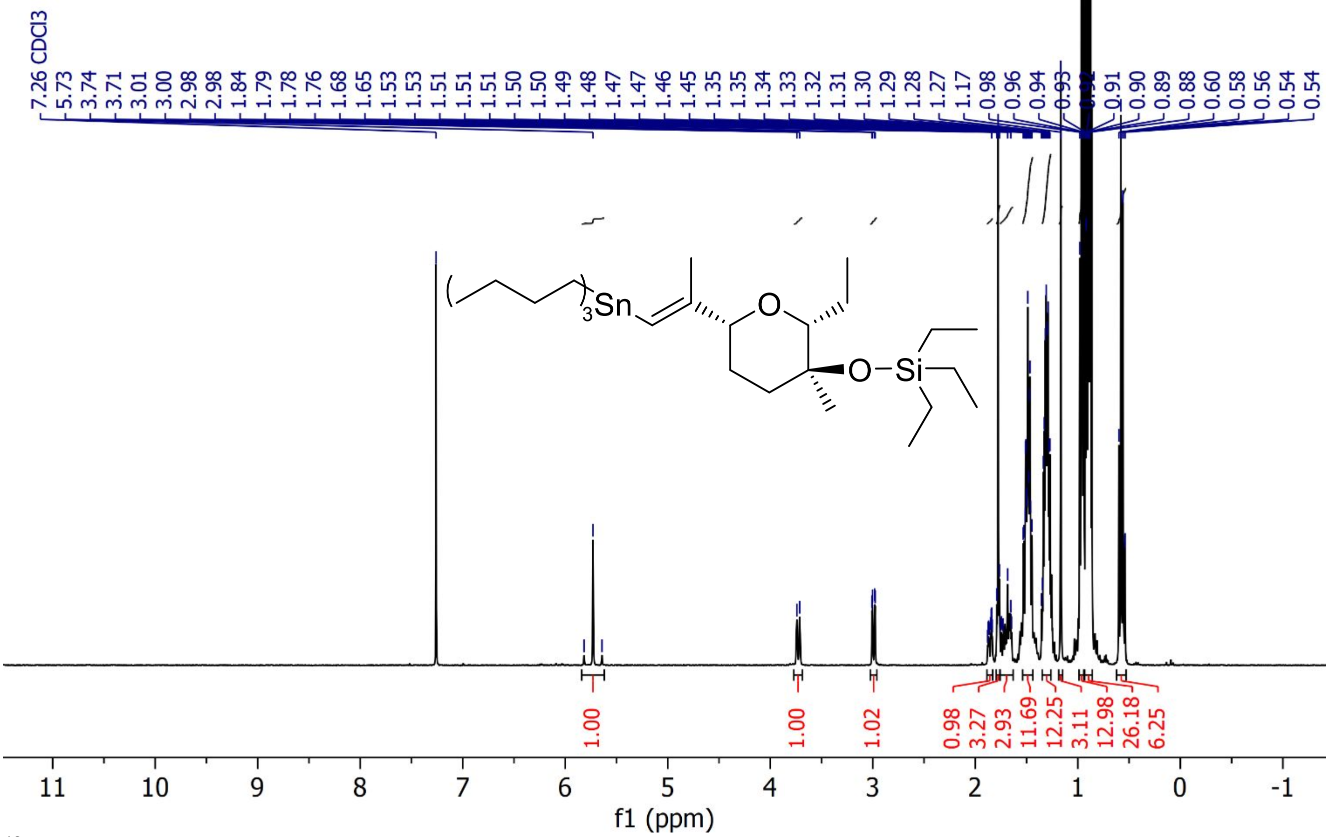

^{13}C-NMR (100 MHz, $CDCl_3$)

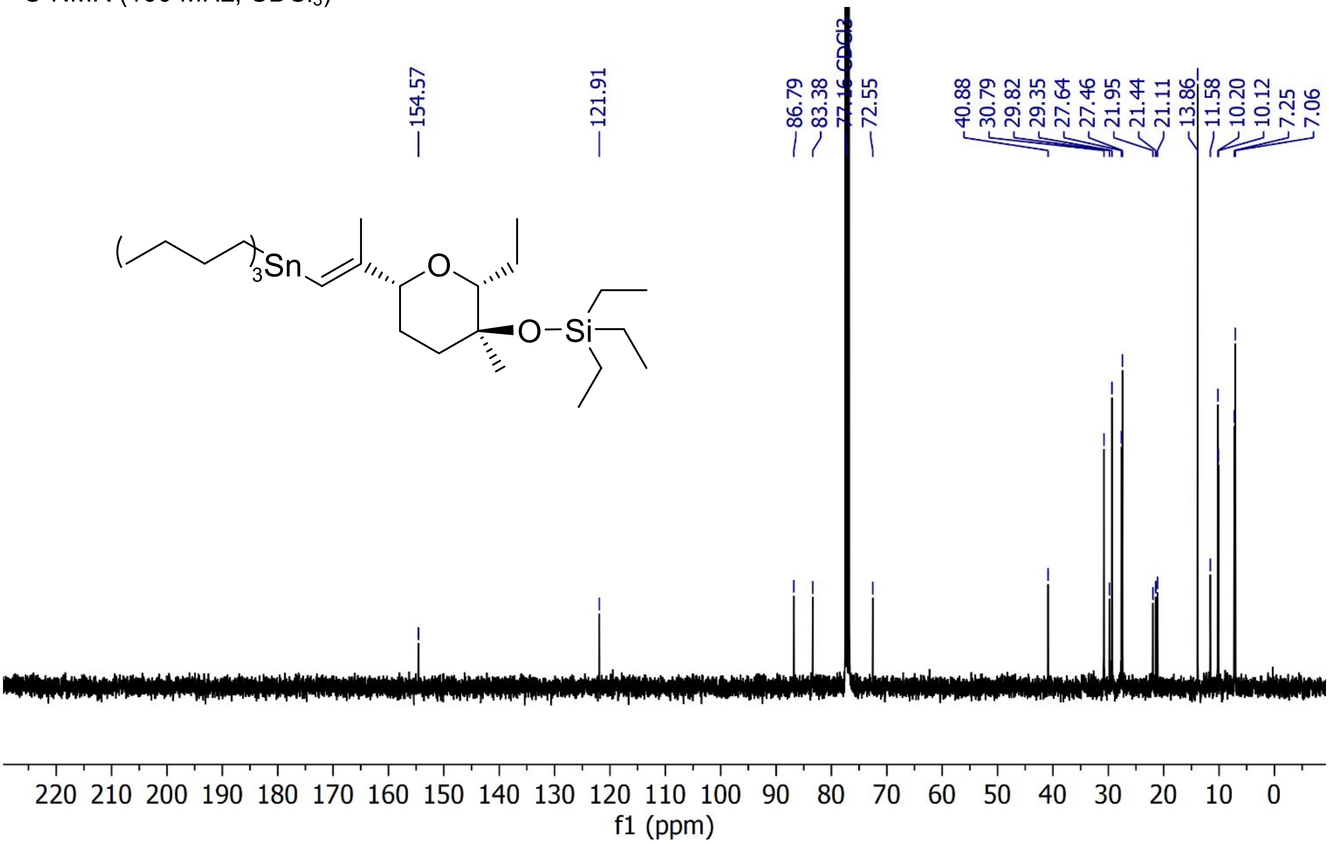

14-TES-Jerangolid B 28

^{1}H-NMR (500 MHz, $CDCl_3$)

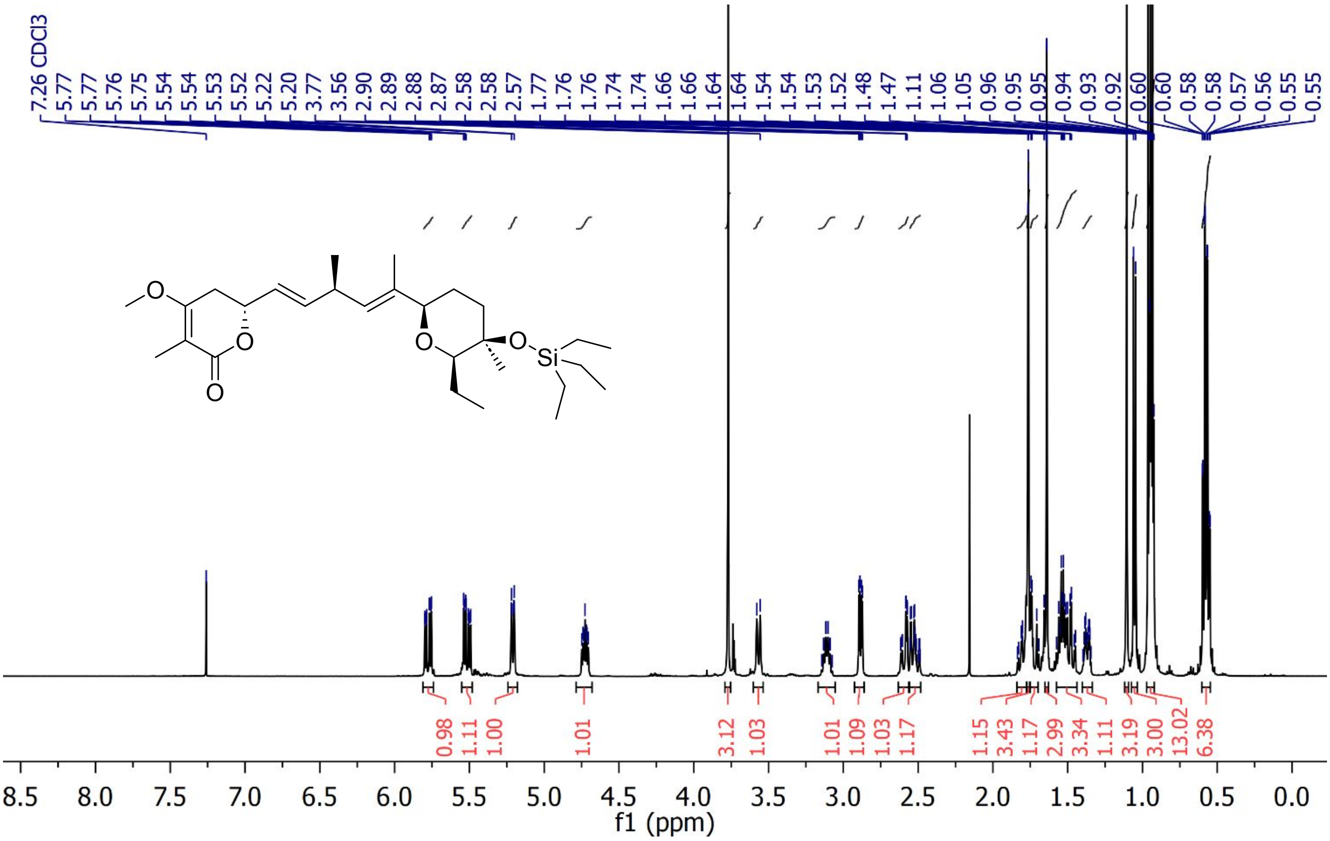

^{13}C-NMR (125 MHz, $CDCl_3$)

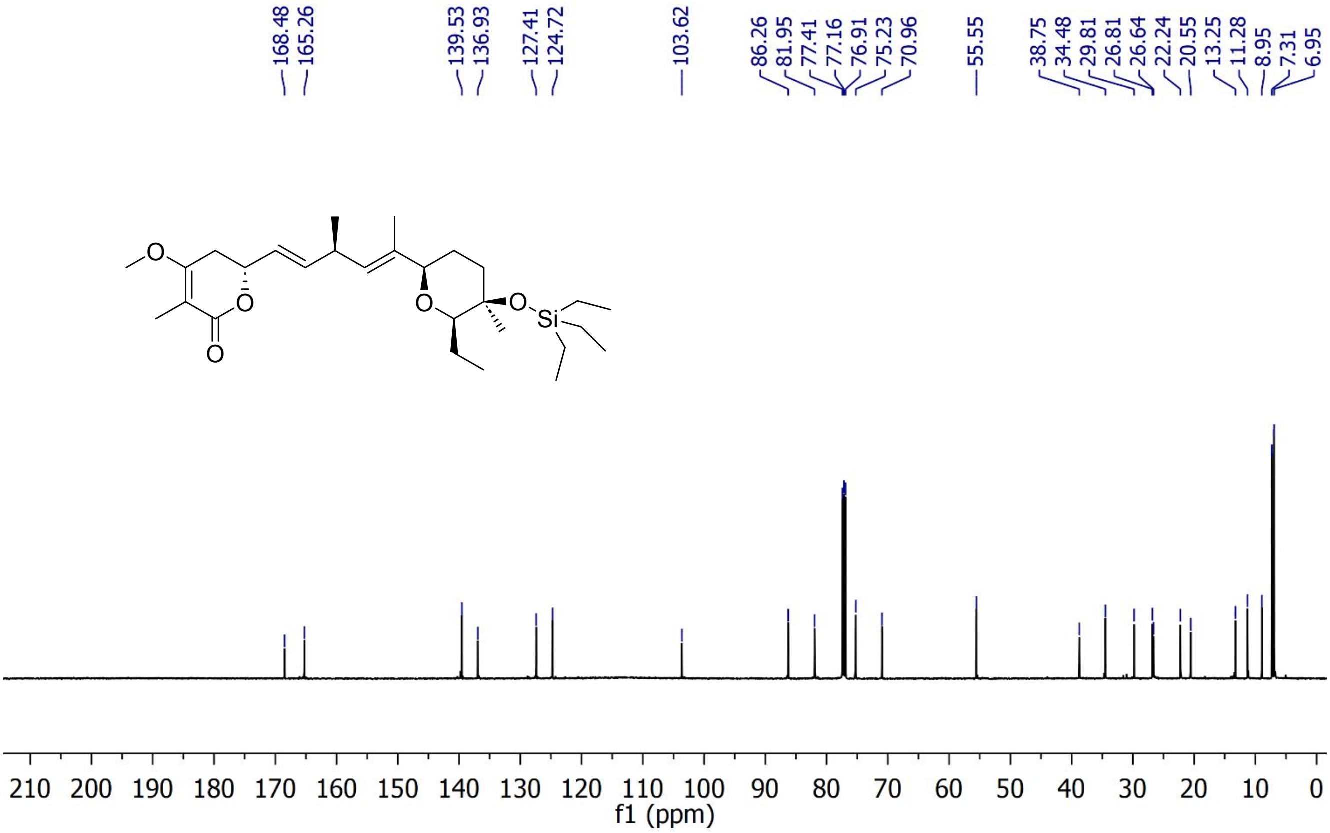

6-((S,2E)-2-((2R,5R,6R)-6-ethyl-5-methyl-5-((triethylsilyl)oxy)tetrahydro-2H-pyran-2-yl)hepta-2,5-dien-4-yl)-4-methoxy-3-methyl-5,6-dihydro-2H-pyran-2-one 29

^{1}H-NMR (400 MHz, $CDCl_3$)

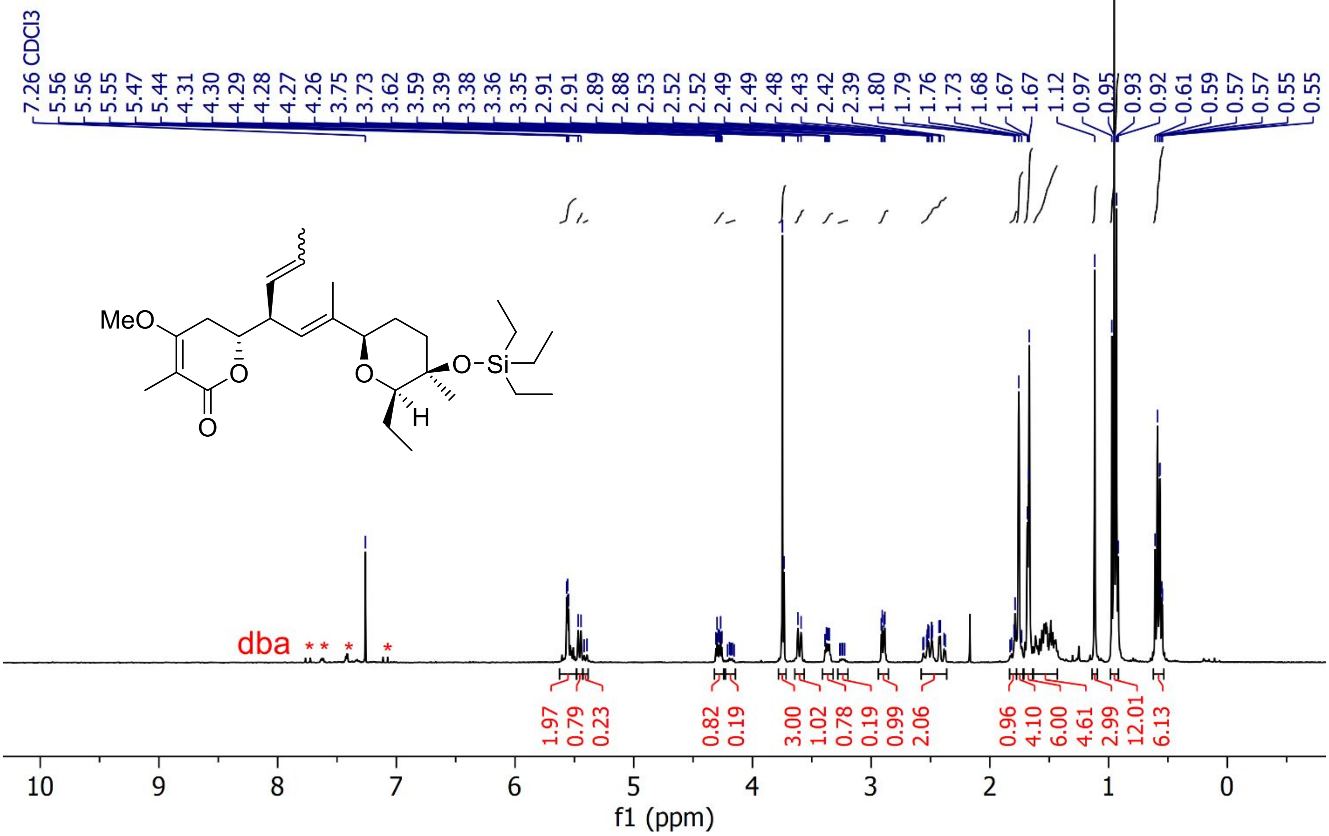

^{13}C-NMR (100 MHz, $CDCl_3$)

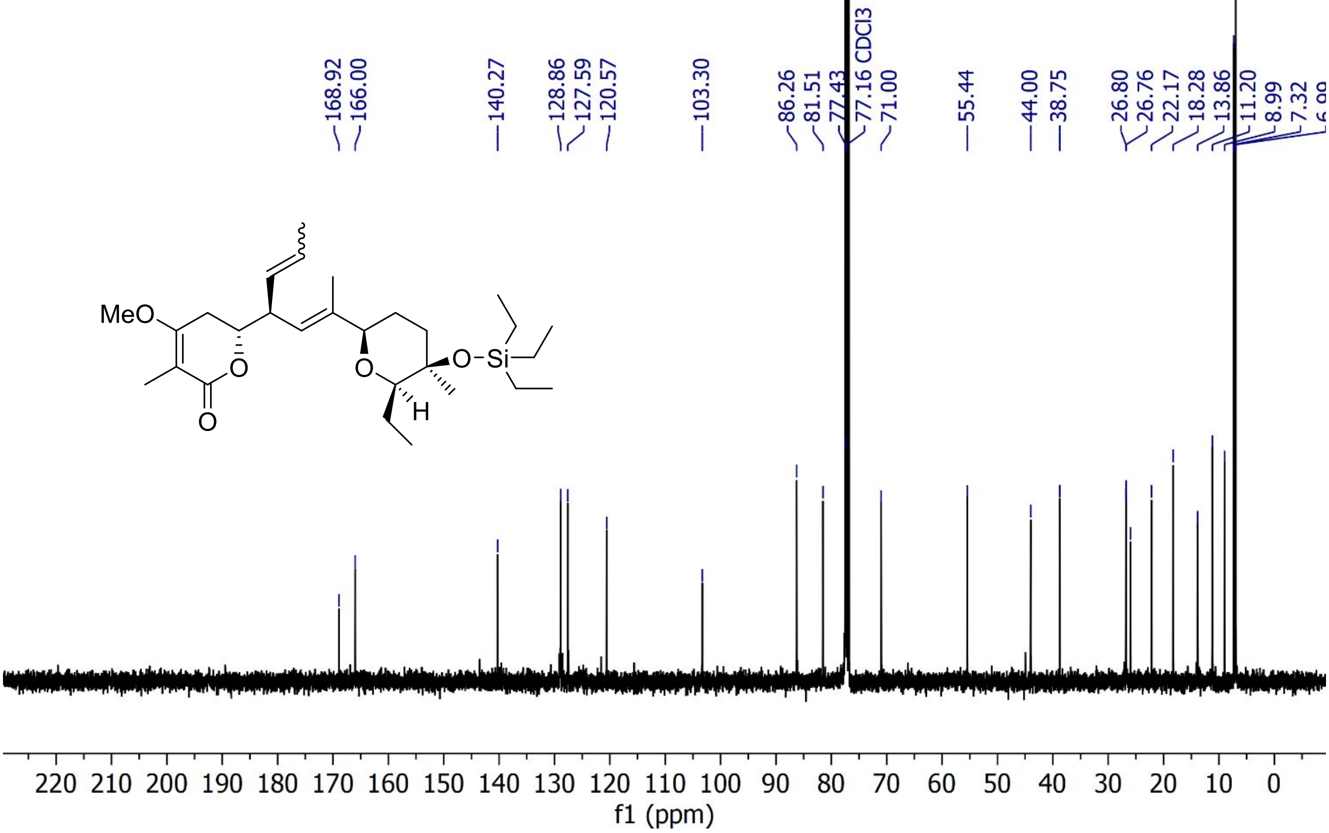

Epi-14-TES-Jerangolid B S13

^{1}H-NMR (400 MHz, MeOH-d_4)

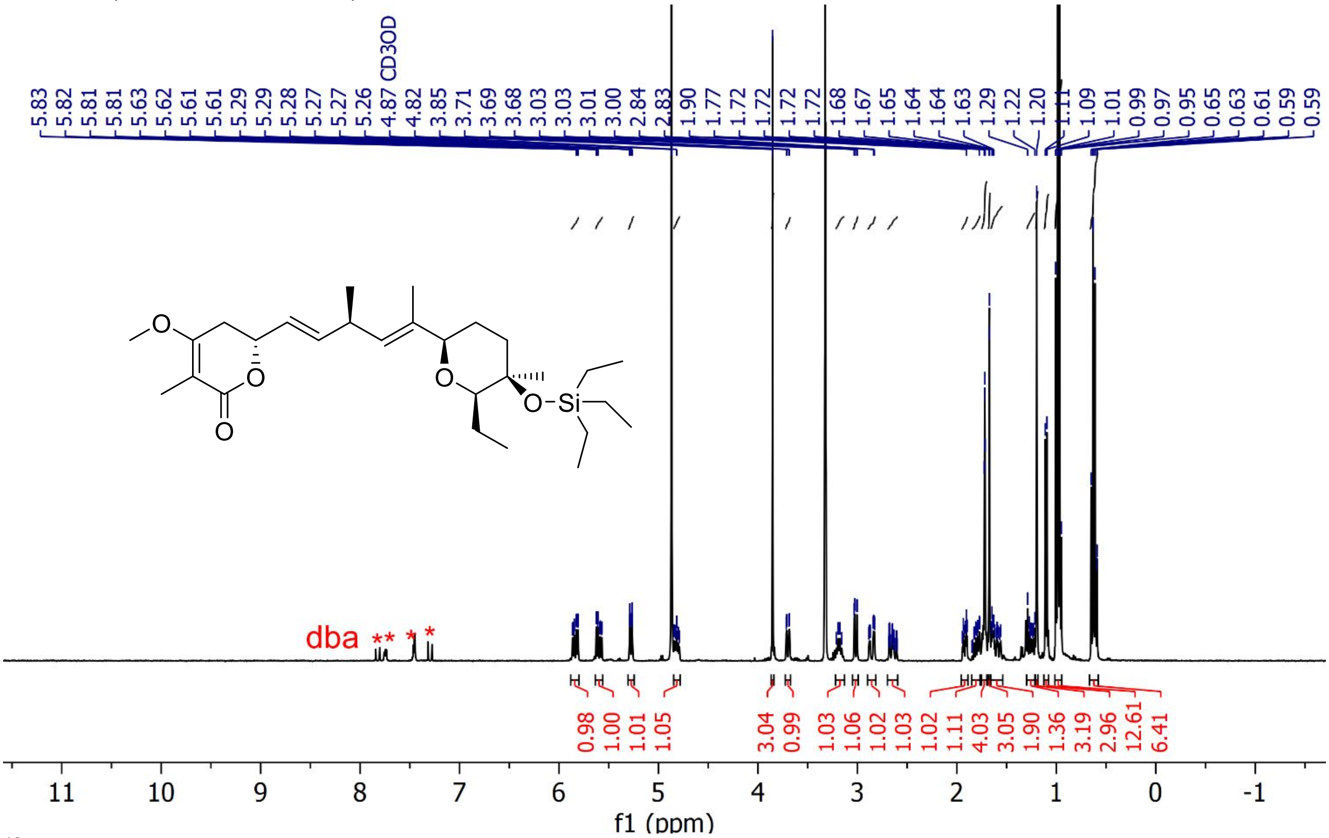

^{13}C-NMR (100 MHz, $CDCl_3$)

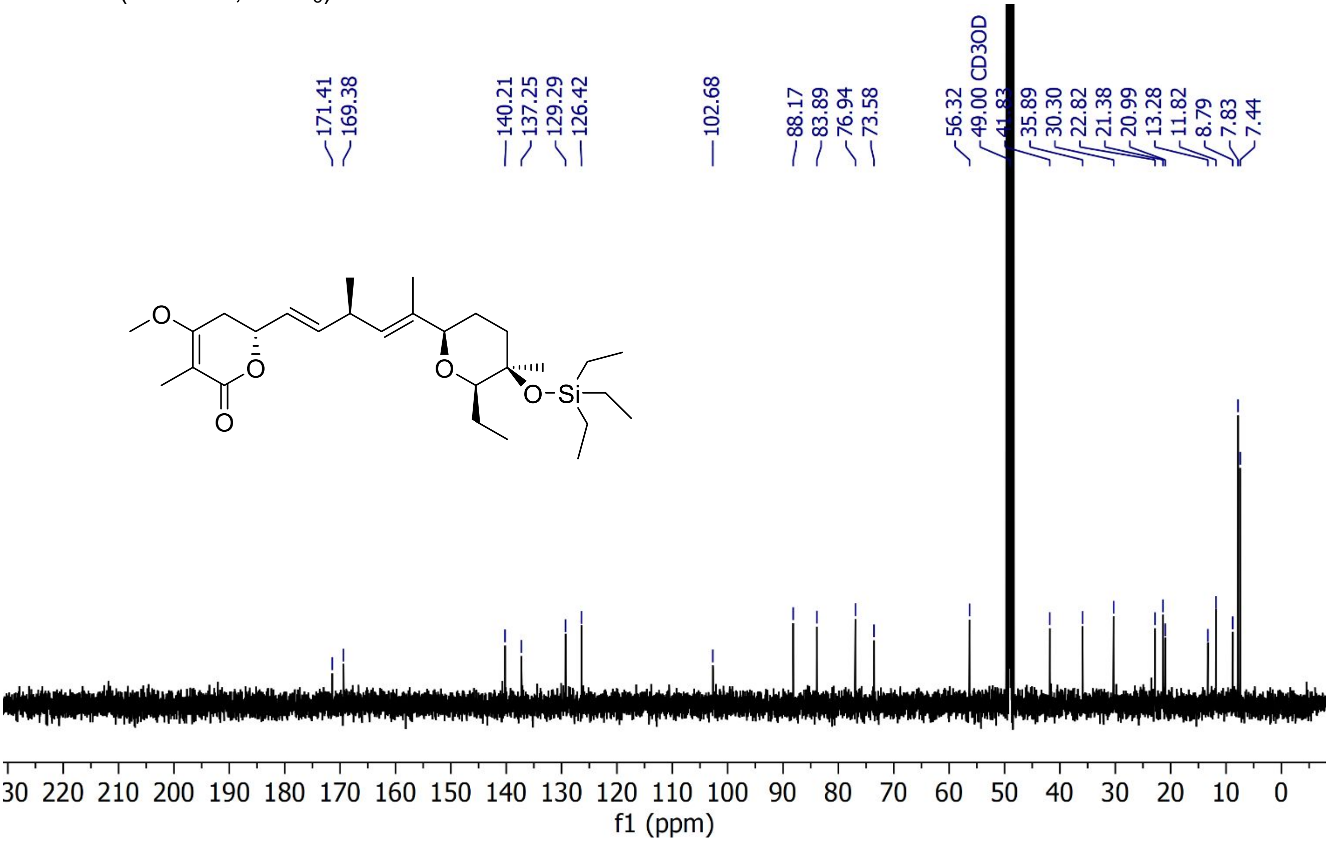

Jerangolid B 1

^{1}H-NMR (400 MHz, MeOH-d_4)

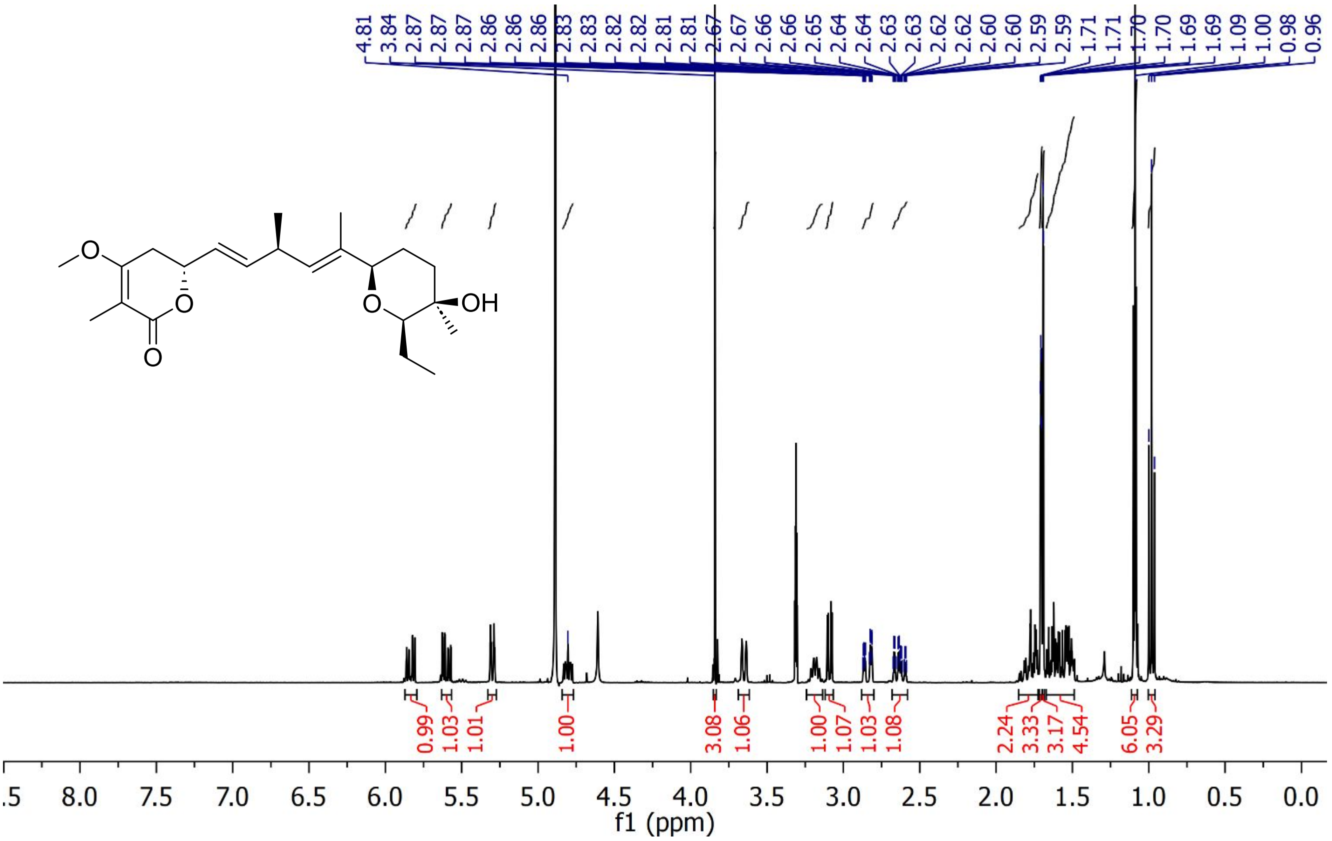

^{13}C-NMR (100 MHz, MeOH-d_4)

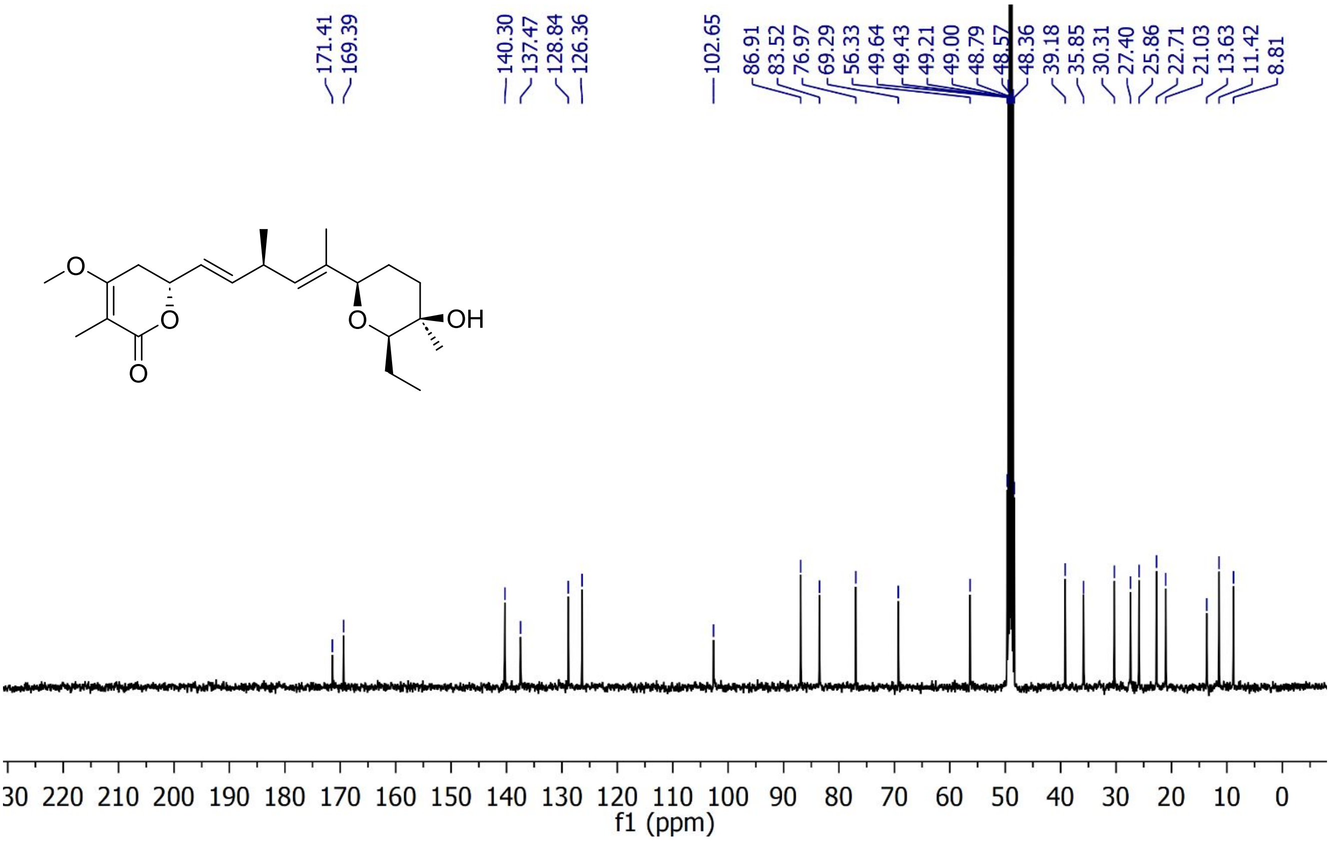

HH-COSY (MeOH-d$_4$)

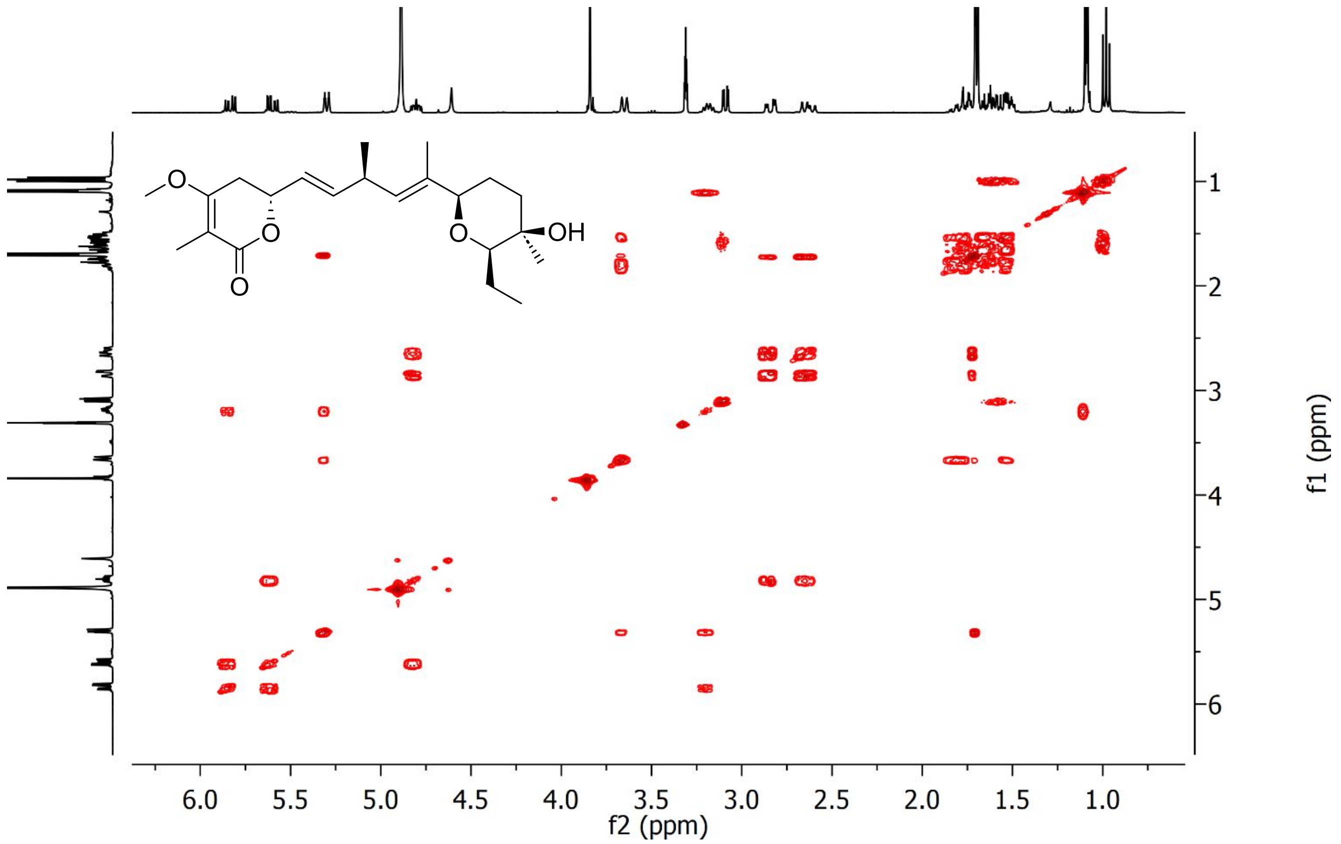

HMBC (MeOH-d$_4$)

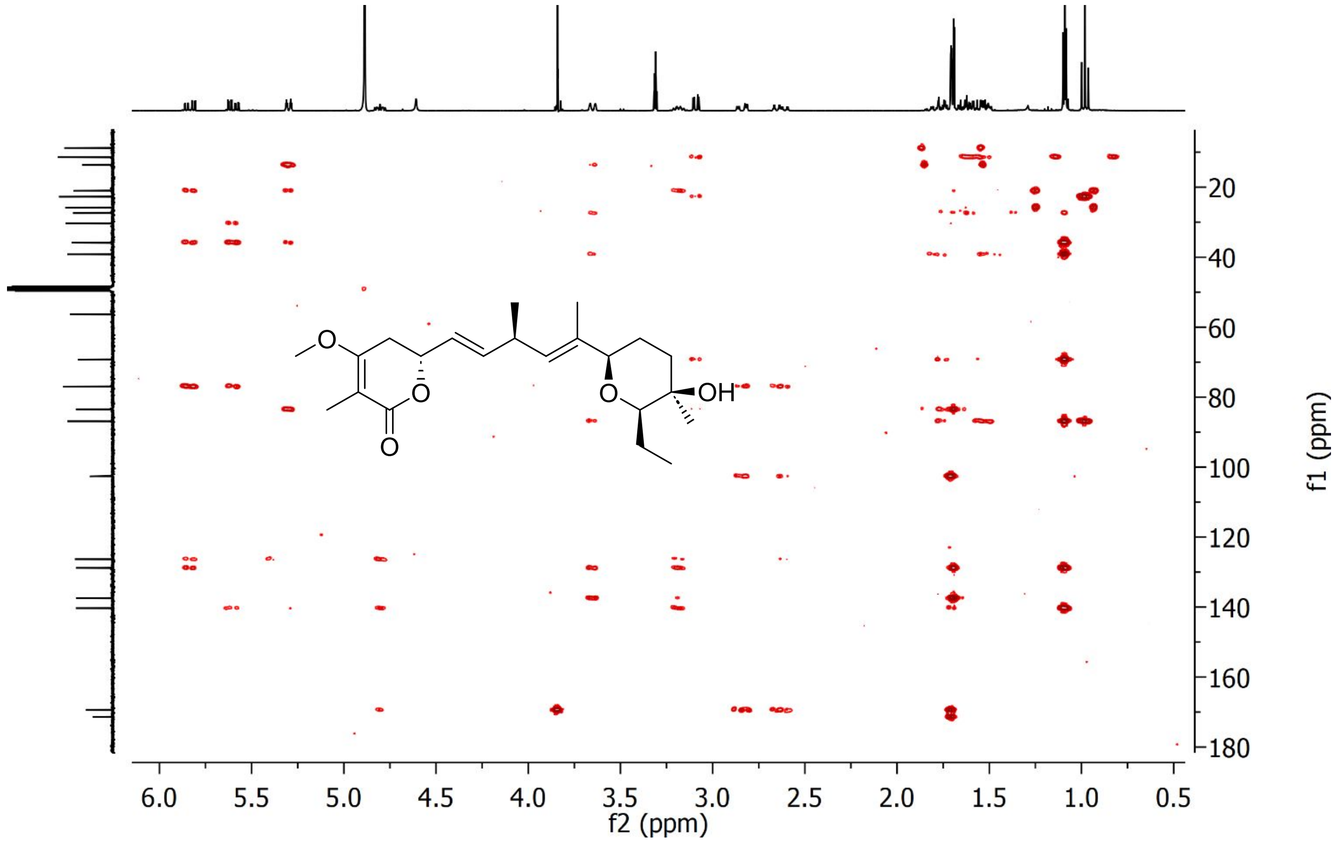

HSQC-DEPT (MeOH-d_4)

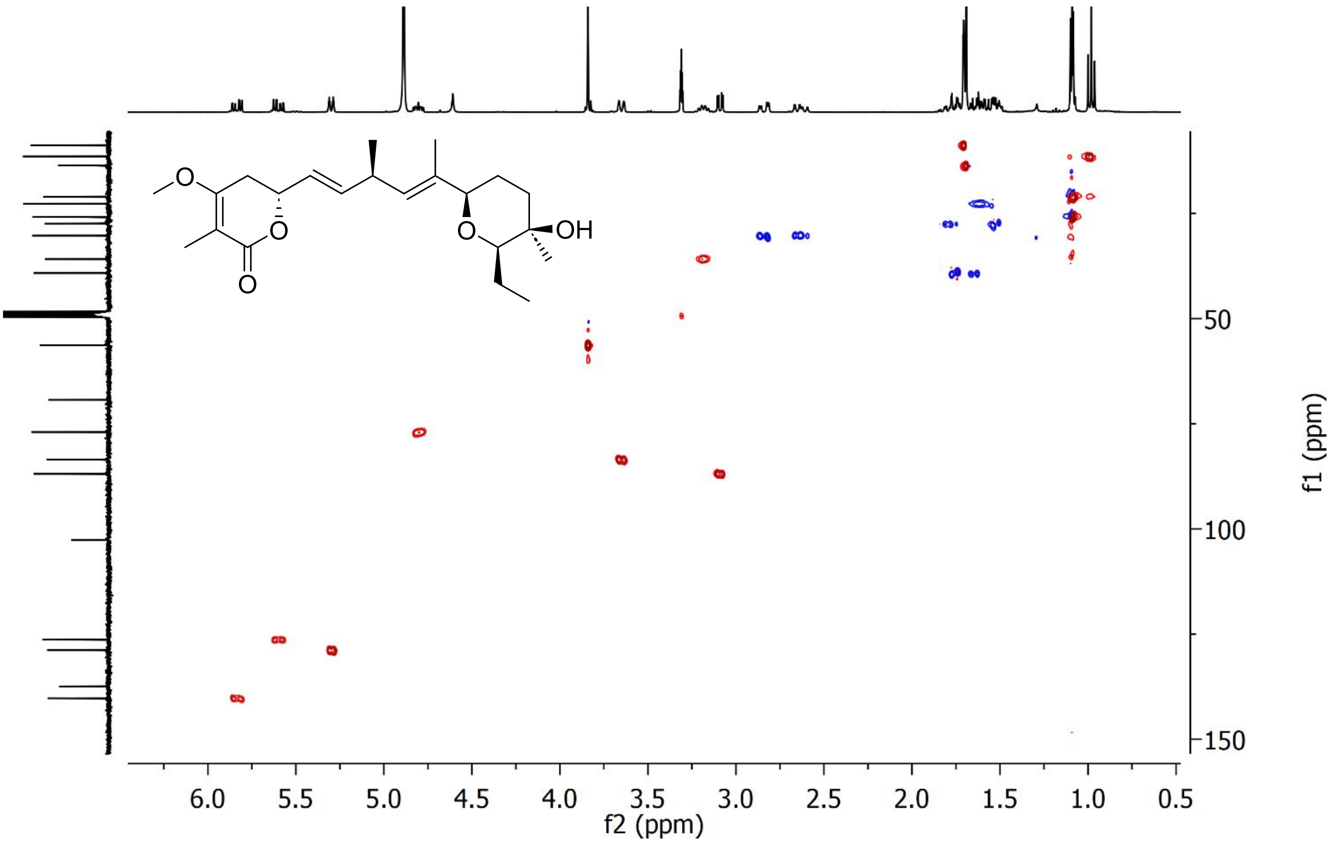

14-S-Jerangolid B S14

^{1}H-NMR (400 MHz, MeOH-d_4)

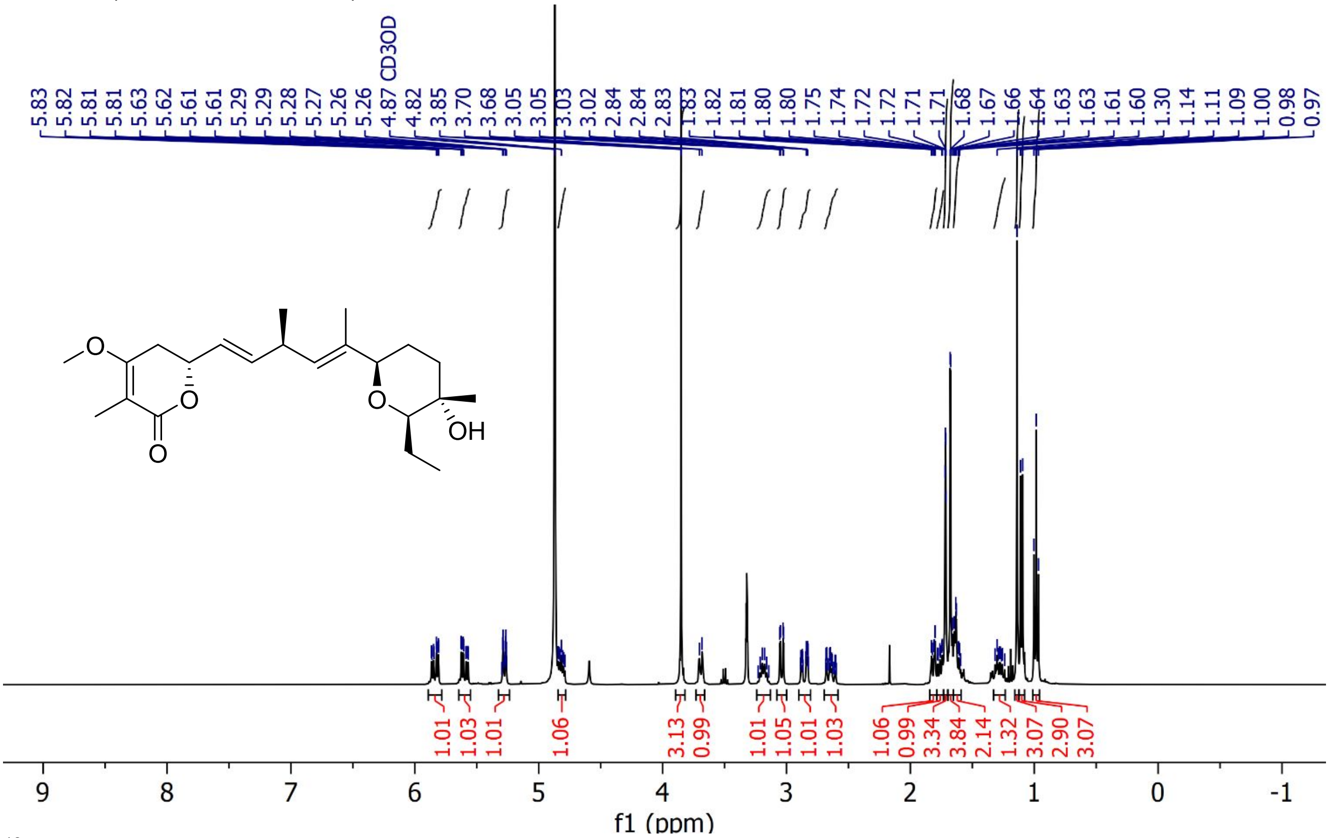

^{13}C-NMR (100 MHz, MeOH-d_4)

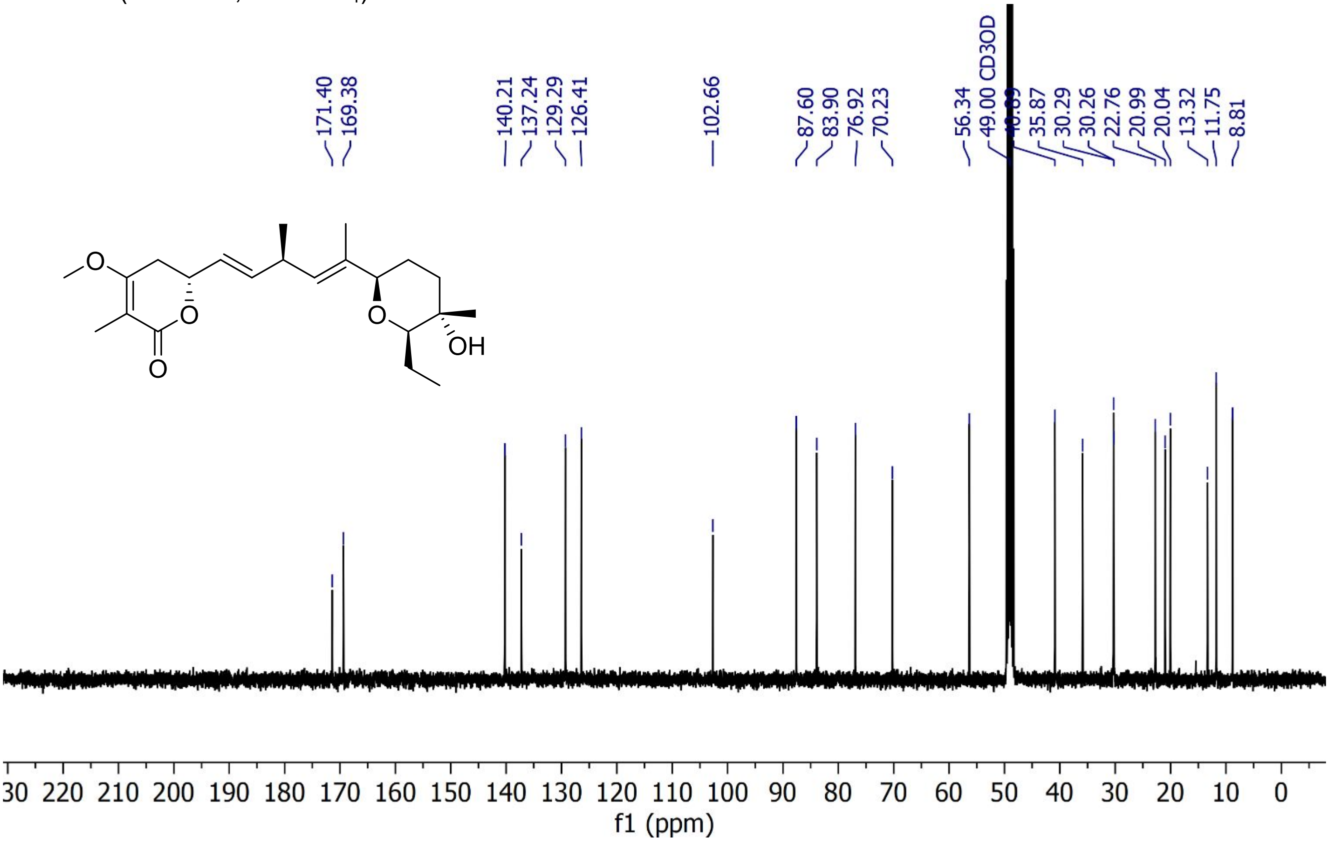

HH-COSY (MeOH-d_4)

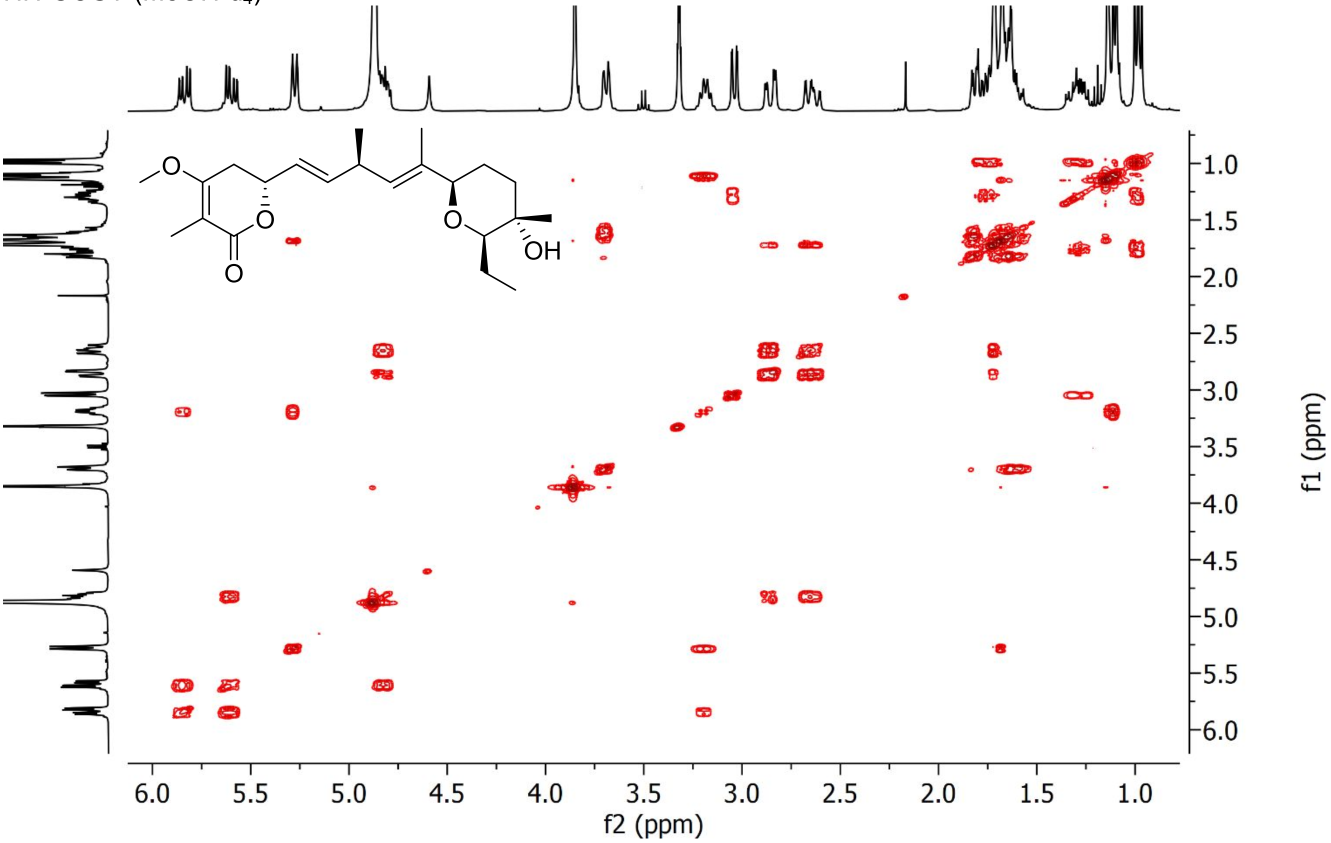

HMBC (MeOH-d_4)

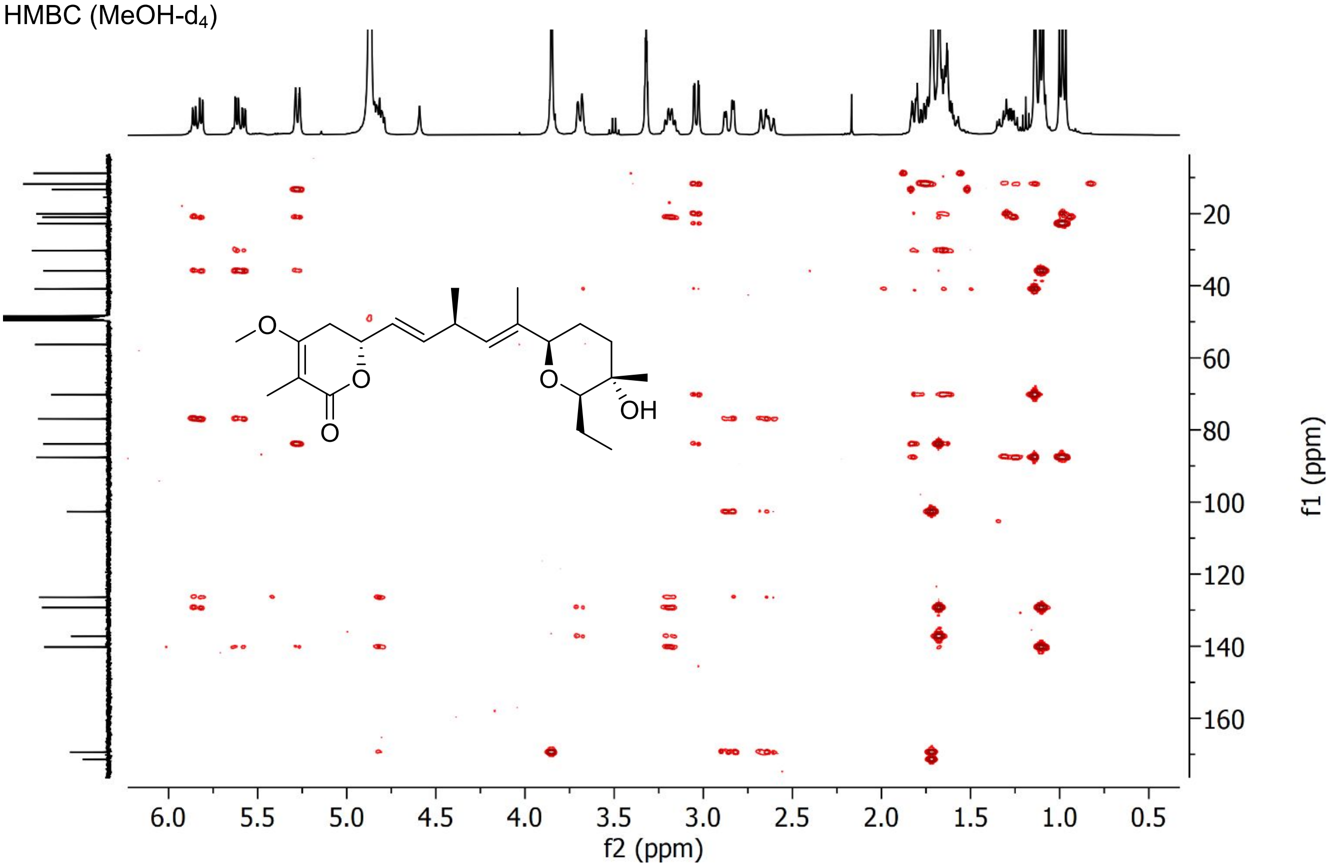

HSQC-DEPT (MeOH-d_4)

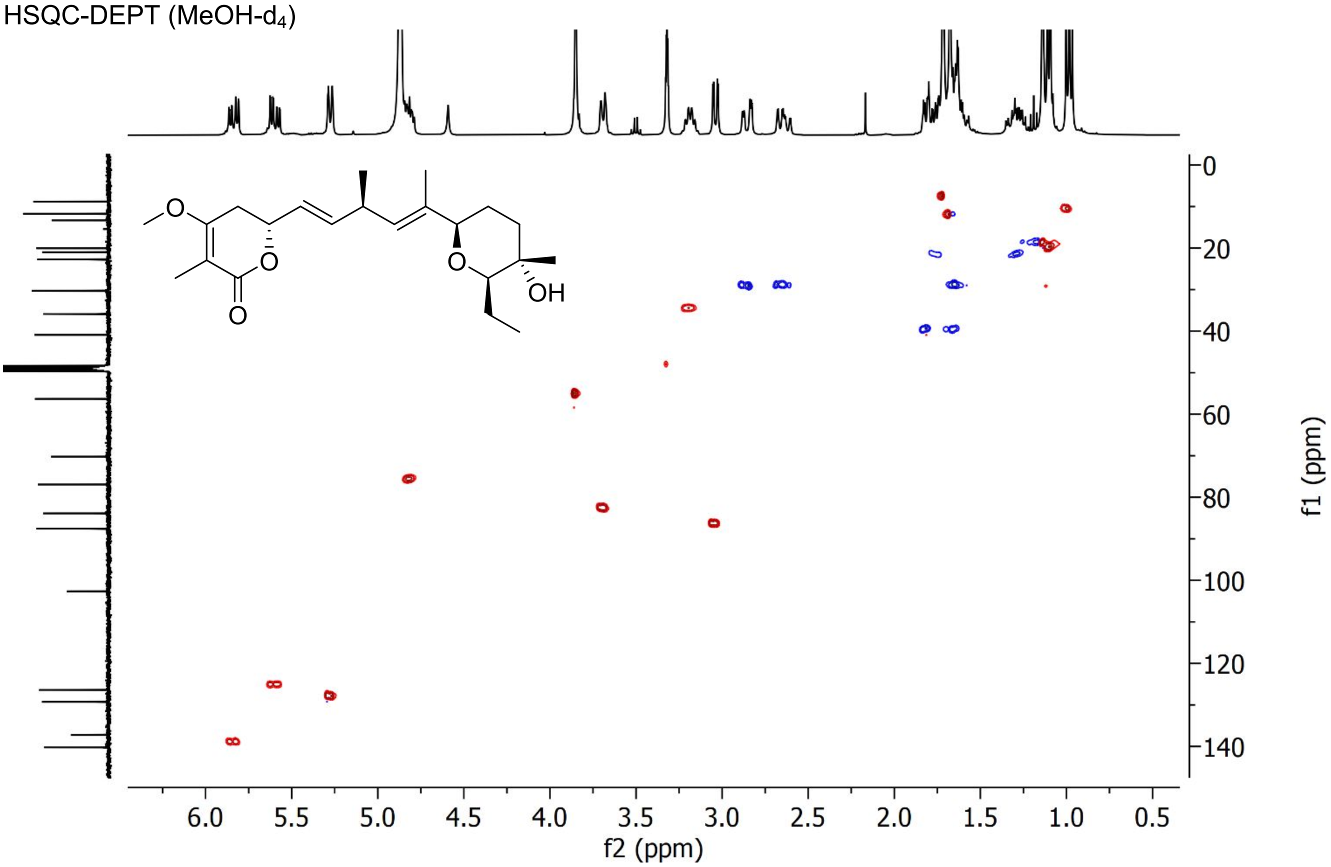

X-Ray crystallographic data

The data set for **S11** was collected using a Bruker X8 Apex diffractometer. Graphite-monochromated $Mo_{K\alpha}$ radiation (λ = 0.71073 Å) was used. Data were collected at 152(2) K and corrected for absorption effects using the multi-scan method. The structure was solved by direct methods using SHELXT[8] and was refined by full matrix least squares calculations on F^2 (SHELXL2018[9]) in the graphical user interface Shelxle[10].

All non H-atoms were located in the electron density maps and refined anisotropically. C-bound H atoms were placed in positions of optimized geometry and treated as riding atoms. Their isotropic displacement parameters were coupled to the corresponding carrier atoms by a factor of 1.2 (CH, CH_2) or 1.5 (CH_3).

Disorder: The $Si(CH_3)_2(C(CH_3)_3)$ unit was split over two positions. Its occupancy factors refined to 0.78 for the major component.

Twinning: The crystal was found to be non-merohedrally twinned. The orientation matrices for the both components were identified using the programm APEX 4 from Bruker. The exact twin matrix identified by the program was found to be (0.99475 -0.06325 -0.01539, 0.03592 -0.99888 -0.00335, 0.02596 0.00477 1.00373). The data were corrected for absorption using twinabs, and the structure was solved using SHELXT with only the non-overlapping reflections of component 1. The structure was refined using the hkfl5 routine with all reflections of component 1 and component 2, resulting in a BASF value of 0.667(1).

***tert*-butyl(((2R,3S,6R)-2-ethyl-6-ethynyl-3-methyltetrahydro-2H-pyran-3-yl)oxy)dimethylsilane S11**

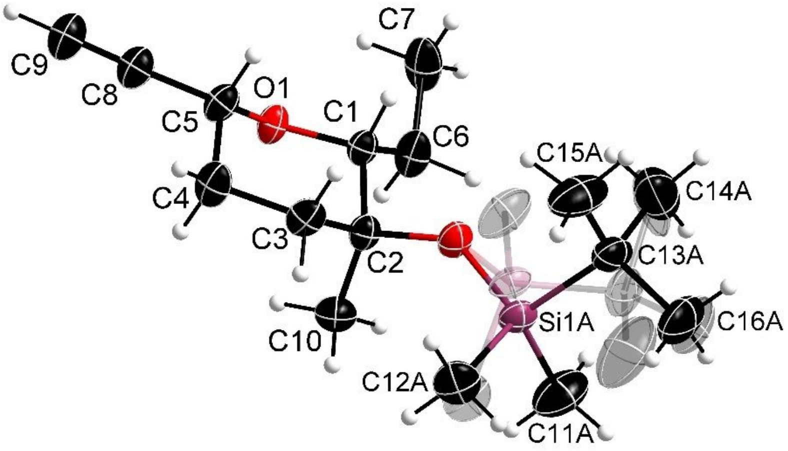

Molecular structure of the pyran **S11**. Displacement ellipsoids are shown at a 50% probability level.

Table S6. Crystal data and structure refinement for pyran **S11**.

Identification code	2464216	
	(sh5146_a_tw)	
Empirical formula	$C_{16}H_{30}O_2Si$	
Formula weight	282.49	
Temperature	152(2) K	
Wavelength	0.71073 Å	
Crystal system	Monoclinic	
Space group	$P2_1$	
Unit cell dimensions	a = 9.8748(4) Å	α = 90°.
	b = 7.3429(3) Å	β = 101.350(2)°.
	c = 12.8549(6) Å	γ = 90°.
Volume	913.88(7) Å^3	
Z	2	
Density (calculated)	1.027 Mg/m^3	
Absorption coefficient	0.126 mm^{-1}	
F(000)	312	
Crystal size	0.200 x 0.200 x 0.020 mm^3	
Theta range for data collection	1.616 to 26.365°.	
Index ranges	-12<=h<=12, -9<=k<=8, -15<=l<=16	
Reflections collected	8070	
Independent reflections	8070 [R(int) = ?]	

Completeness to theta = 25.242°	100.0 %
Absorption correction	Semi-empirical from equivalents
Max. and min. transmission	0.646 and 0.600
Refinement method	Full-matrix least-squares on F^2
Data / restraints / parameters	8070 / 275 / 249
Goodness-of-fit on F^2	0.999
Final R indices [I>2sigma(I)]	R1 = 0.0482, wR2 = 0.0874
R indices (all data)	R1 = 0.0794, wR2 = 0.0991
Absolute structure parameter	0.08(8)
Extinction coefficient	n/a
Largest diff. peak and hole	0.187 and -0.214 e.Å^{-3}

Table S7. Atomic coordinates (x 10^4) and equivalent isotropic displacement parameters (Å^{2}x 10^3) for pyran **S11**. U(eq) is defined as one third of the trace of the orthogonalized Uij tensor.

Atom	X	Y	Z	U(eq)
O(1)	2553(2)	5622(3)	6009(2)	28(1)
O(2)	5769(2)	4022(3)	7620(2)	31(1)
C(1)	3836(3)	4640(5)	6290(3)	26(1)
C(2)	4415(3)	4795(4)	7486(3)	27(1)
C(3)	4551(4)	6819(5)	7781(3)	33(1)
C(4)	3212(4)	7865(5)	7385(3)	37(1)
C(5)	2734(4)	7535(5)	6209(3)	30(1)
C(6)	3561(4)	2724(5)	5882(3)	38(1)
C(7)	3241(4)	2625(6)	4677(3)	51(1)
C(8)	1430(4)	8447(5)	5759(3)	35(1)
C(9)	417(4)	9231(5)	5386(3)	42(1)
C(10)	3537(4)	3785(5)	8140(3)	37(1)
Si(1A)	6957(2)	3316(3)	8620(1)	30(1)
C(11A)	6560(7)	939(8)	8984(5)	60(2)
C(12A)	7094(14)	4809(13)	9820(7)	45(2)
C(13A)	8594(5)	3360(8)	8109(4)	41(1)
C(14A)	8473(11)	2307(16)	7087(6)	71(3)
C(15A)	8975(6)	5327(9)	7888(5)	66(2)
C(16A)	9787(10)	2559(15)	8951(11)	66(3)
Si(1B)	7241(8)	4189(12)	8443(5)	33(2)
C(11B)	8200(20)	6250(30)	8190(20)	57(6)
C(12B)	6910(50)	4160(50)	9820(30)	46(8)
C(13B)	8128(18)	2060(30)	8211(14)	47(4)
C(14B)	8040(40)	1780(60)	7020(20)	72(9)
C(15B)	7320(30)	390(30)	8460(30)	97(9)
C(16B)	9610(30)	1930(60)	8830(40)	63(9)

Table S8. Bond lengths [Å] for pyran **S11**.

Atoms	Bond length [Å]	Atoms	Bond length [Å]
O(1)-C(5)	1.433(4)	C(8)-C(9)	1.172(5)
O(1)-C(1)	1.440(4)	Si(1A)-C(13A)	1.861(5)
O(2)-C(2)	1.432(4)	Si(1A)-C(11A)	1.868(6)
O(2)-Si(1B)	1.625(7)	Si(1A)-C(12A)	1.876(8)
O(2)-Si(1A)	1.644(3)	C(13A)-C(14A)	1.509(9)
C(1)-C(6)	1.507(5)	C(13A)-C(15A)	1.533(8)
C(1)-C(2)	1.536(4)	C(13A)-C(16A)	1.550(9)
C(2)-C(10)	1.515(5)	Si(1B)-C(13B)	1.846(19)
C(2)-C(3)	1.533(5)	Si(1B)-C(11B)	1.849(19)
C(3)-C(4)	1.528(4)	Si(1B)-C(12B)	1.86(3)
C(4)-C(5)	1.512(4)	C(13B)-C(16B)	1.52(2)
C(5)-C(8)	1.466(5)	C(13B)-C(15B)	1.53(2)
C(6)-C(7)	1.521(5)	C(13B)-C(14B)	1.53(2)

Table S9. Bond angles [°] for pyran **S11**.

Atoms	Bond angle [°]	Atoms	Bond angle [°]
C(5)-O(1)-C(1)	111.9(2)	O(2)-Si(1A)-C(12A)	112.6(4)
C(2)-O(2)-Si(1B)	138.0(3)	C(13A)-Si(1A)-C(12A)	110.7(4)
C(2)-O(2)-Si(1A)	136.5(2)	C(11A)-Si(1A)-C(12A)	109.0(4)
O(1)-C(1)-C(6)	106.9(3)	C(14A)-C(13A)-C(15A)	107.6(6)
O(1)-C(1)-C(2)	110.5(3)	C(14A)-C(13A)-C(16A)	109.4(7)
C(6)-C(1)-C(2)	115.0(3)	C(15A)-C(13A)-C(16A)	107.8(5)
O(2)-C(2)-C(10)	111.2(3)	C(14A)-C(13A)-Si(1A)	111.9(5)
O(2)-C(2)-C(3)	108.7(3)	C(15A)-C(13A)-Si(1A)	110.1(4)
C(10)-C(2)-C(3)	111.7(3)	C(16A)-C(13A)-Si(1A)	109.9(6)
O(2)-C(2)-C(1)	104.3(2)	O(2)-Si(1B)-C(13B)	102.9(7)
C(10)-C(2)-C(1)	112.2(3)	O(2)-Si(1B)-C(11B)	111.8(8)
C(3)-C(2)-C(1)	108.5(3)	C(13B)-Si(1B)-C(11B)	112.8(13)
C(4)-C(3)-C(2)	112.1(3)	O(2)-Si(1B)-C(12B)	108.5(16)
C(5)-C(4)-C(3)	109.4(3)	C(13B)-Si(1B)-C(12B)	108.7(16)
O(1)-C(5)-C(8)	107.9(3)	C(11B)-Si(1B)-C(12B)	111.7(13)
O(1)-C(5)-C(4)	109.9(3)	C(16B)-C(13B)-C(15B)	109(2)
C(8)-C(5)-C(4)	113.5(3)	C(16B)-C(13B)-C(14B)	112(3)
C(1)-C(6)-C(7)	112.7(3)	C(15B)-C(13B)-C(14B)	100(2)
C(9)-C(8)-C(5)	177.3(4)	C(16B)-C(13B)-Si(1B)	114(2)
O(2)-Si(1A)-C(13A)	104.7(2)	C(15B)-C(13B)-Si(1B)	111.1(17)
O(2)-Si(1A)-C(11A)	109.8(2)	C(14B)-C(13B)-Si(1B)	109.8(18)
C(13A)-Si(1A)-C(11A)	110.1(3)		

Table S10. Anisotropic displacement parameters ($Å^2 x 10^3$) for pyran **S11**. The anisotropic displacement factor exponent takes the form: $-2p^2[h^2 a^{*2}U^{11} + ... + 2 h k a^* b^* U^{12}]$

Atom	U^{11}	U^{22}	U^{33}	U^{23}	U^{13}	U^{12}
O(1)	22(1)	24(2)	33(2)	-4(1)	-3(1)	1(1)
O(2)	27(1)	36(2)	26(1)	-2(1)	-3(1)	4(1)
C(1)	22(2)	27(2)	28(2)	0(2)	0(2)	1(2)
C(2)	23(2)	27(2)	28(2)	-2(2)	1(2)	0(2)
C(3)	36(2)	31(2)	27(2)	-6(2)	-4(2)	-2(2)
C(4)	41(2)	27(2)	39(2)	-11(2)	-2(2)	2(2)
C(5)	30(2)	23(2)	34(2)	1(2)	-2(2)	-1(2)
C(6)	36(2)	32(2)	40(2)	-13(2)	-7(2)	4(2)
C(7)	45(3)	57(3)	45(3)	-24(2)	-5(2)	14(2)
C(8)	33(2)	27(2)	41(2)	-1(2)	1(2)	-1(2)
C(9)	35(2)	36(2)	52(3)	1(2)	0(2)	3(2)
C(10)	39(2)	37(2)	34(2)	1(2)	8(2)	-3(2)
Si(1A)	27(1)	36(1)	24(1)	3(1)	-2(1)	-3(1)
C(11A)	57(4)	48(4)	71(5)	23(3)	1(3)	-12(3)
C(12A)	43(5)	57(7)	31(4)	-10(4)	-6(3)	-4(5)
C(13A)	31(3)	57(4)	34(3)	14(3)	2(2)	5(3)
C(14A)	57(6)	98(7)	58(5)	-5(4)	15(4)	18(5)
C(15A)	42(4)	82(5)	69(5)	38(4)	2(3)	-16(4)
C(16A)	36(4)	83(8)	77(6)	36(5)	4(4)	14(4)
Si(1B)	34(3)	37(5)	24(3)	4(3)	-5(2)	-3(3)
C(11B)	42(12)	51(13)	66(14)	16(11)	-17(11)	-8(10)
C(12B)	33(13)	47(19)	51(13)	-3(12)	-7(10)	-13(14)
C(13B)	33(8)	54(8)	48(8)	-4(7)	-9(7)	11(7)
C(14B)	54(18)	100(19)	62(11)	-50(12)	13(12)	22(15)
C(15B)	84(17)	49(14)	150(20)	27(14)	-1(16)	0(12)
C(16B)	34(12)	70(20)	78(16)	9(15)	-13(11)	10(13)

Table S11. Hydrogen coordinates (x 10^4) and isotropic displacement parameters ($Å^2 x 10^3$) for pyran **S11**.

Atom	X	Y	Z	U(eq)
H(1)	4515	5209	5905	31
H(3A)	4811	6936	8562	39
H(3B)	5300	7364	7472	39
H(4A)	2493	7454	7771	44
H(4B)	3368	9183	7520	44
H(5)	3463	7990	5834	36

Atom	X	Y	Z	U(eq)
H(6A)	2771	2218	6158	45
H(6B)	4379	1960	6156	45
H(7A)	3149	1347	4453	76
H(7B)	3994	3192	4397	76
H(7C)	2376	3270	4403	76
H(9)	-404	9867	5084	51
H(10A)	2579	4206	7946	55
H(10B)	3890	4019	8895	55
H(10C)	3575	2475	8001	55
H(11A)	6387	179	8345	90
H(11B)	5739	945	9306	90
H(11C)	7347	448	9492	90
H(12A)	7246	6073	9625	68
H(12B)	7871	4404	10368	68
H(12C)	6237	4731	10094	68
H(14A)	7675	2751	6572	106
H(14B)	8350	1010	7224	106
H(14C)	9315	2474	6802	106
H(15A)	9763	5327	7529	98
H(15B)	9221	5992	8560	98
H(15C)	8184	5921	7435	98
H(16A)	10654	2644	8691	99
H(16B)	9591	1279	9079	99
H(16C)	9870	3247	9613	99
H(11D)	8306	6257	7446	85
H(11E)	9119	6240	8653	85
H(11F)	7697	7335	8334	85
H(12D)	6590	5360	9994	68
H(12E)	7768	3852	10315	68
H(12F)	6206	3243	9870	68
H(14D)	8152	2959	6682	108
H(14E)	7144	1257	6700	108
H(14F)	8781	956	6904	108
H(15D)	6533	183	7872	146
H(15E)	6977	604	9113	146
H(15F)	7922	-678	8544	146
H(16D)	10024	3141	8904	95
H(16E)	10141	1123	8459	95
H(16F)	9601	1429	9541	95

Literature

(1) Könning, D.; Hiller, W.; Christmann, M. One-Pot Oxidation/Isomerization of Z-Allylic Alcohols with Oxygen as Stoichiometric Oxidant. *Org. Lett.* **2012**, *14* (20), 5258–5261.
(2) Prantz, K.; Mulzer, J. Synthesis of (Z)-Trisubstituted Olefins by Decarboxylative Grob-Type Fragmentations: Epothilone D, Discodermolide, and Peloruside A. *Chem. Eur. J.* **2010**, *16* (2), 485–506.
(3) Sato, M.; Ogasawara, H.; Oi, K.; Kato, T. Synthesis of 1,3-Dioxin-4-One Derivatives. *Chem. Pharm. Bull.* **1983**, *31* (6), 1896–1901.
(4) Hoye, T. R.; Jeffrey, C. S.; Shao, F. Mosher Ester Analysis for the Determination of Absolute Configuration of Stereogenic (Chiral) Carbinol Carbons. *Nat. Protoc.* **2007**, *2* (10), 2451–2458.
(5) Zhao, M.-X.; Shi, Y. Practical Synthesis of an L -Fructose-Derived Ketone Catalyst for Asymmetric Epoxidation of Olefins. *J. Org. Chem.* **2006**, *71* (14), 5377–5379.
(6) Lenhof, J.; Hutter, M.; Huch, V.; Jauch, J. Towards the Total Synthesis of Jerangolids – Synthesis of an Advanced Intermediate for the Pharmacophore Substructure. *Eur. J. Org. Chem.* **2020**, *36* (36), 5833–5840.
(7) Reichenbach, H.; Höfle, G.; Gerth, K.; Washausen, P. PCT Int. Appl. DE 19607702 **1997**.
(8) Sheldrick, G. M. *SHELXT* – Integrated Space-Group and Crystal-Structure Determination. *Acta Crystallogr. A* **2015**, *71* (1), 3–8.
(9) Sheldrick, G. M. Crystal Structure Refinement with *SHELXL*. *Acta Crystallogr. C* **2015**, *71* (1), 3–8.
(10) Hübschle, C. B.; Sheldrick, G. M.; Dittrich, B. *ShelXle* : A Qt Graphical User Interface for *SHELXL*. *J. Appl. Crystallogr.* **2011**, *44* (6), 1281–1284.
